# Hyperosmotic Stress Induces a Specific Pattern for Stress Granule Formation in Human-Induced Pluripotent Stem Cells

**DOI:** 10.1155/2021/8274936

**Published:** 2021-10-15

**Authors:** Salam Salloum-Asfar, Rudolf Engelke, Hanaa Mousa, Neha Goswami, I. Richard Thompson, Freshteh Palangi, Kamal Kamal, Muna N. Al-Noubi, Frank Schmidt, Sara A. Abdulla, Mohamed M. Emara

**Affiliations:** ^1^Neurological Disorders Research Center, Qatar Biomedical Research Institute (QBRI), Hamad Bin Khalifa University (HBKU), Education City, Qatar Foundation (QF), Doha, Qatar; ^2^Proteomics Core, Weill Cornell Medical College-Qatar, Qatar Foundation, Doha, Qatar; ^3^Basic Medical Sciences Department, College of Medicine, QU Health, Qatar University, Doha, Qatar; ^4^Diabetes Research Center, Qatar Biomedical Research Institute (QBRI), Hamad Bin Khalifa University (HBKU), Education City, Qatar Foundation (QF), Doha, Qatar; ^5^Biomedical and Pharmaceutical Research Unit, QU Health, Qatar University, Doha, Qatar

## Abstract

Stress granules (SGs) are assemblies of selective messenger RNAs (mRNAs), translation factors, and RNA-binding proteins in small untranslated messenger ribonucleoprotein (mRNP) complexes in the cytoplasm. Evidence indicates that different types of cells have shown different mechanisms to respond to stress and the formation of SGs. In the present work, we investigated how human-induced pluripotent stem cells (hiPSCs/IMR90-1) overcome hyperosmotic stress compared to a cell line that does not harbor pluripotent characteristics (SH-SY5Y cell line). Gradient concentrations of NaCl showed a different pattern of SG formation between hiPSCs/IMR90-1 and the nonpluripotent cell line SH-SY5Y. Other pluripotent stem cell lines (hiPSCs/CRTD5 and hESCs/H9 (human embryonic stem cell line)) as well as nonpluripotent cell lines (BHK-21 and MCF-7) were used to confirm this phenomenon. Moreover, the formation of hyperosmotic SGs in hiPSCs/IMR90-1 was independent of eIF2*α* phosphorylation and was associated with low apoptosis levels. In addition, a comprehensive proteomics analysis was performed to identify proteins involved in regulating this specific pattern of hyperosmotic SG formation in hiPSCs/IMR90-1. We found possible implications of microtubule organization on the response to hyperosmotic stress in hiPSCs/IMR90-1. We have also unveiled a reduced expression of tubulin that may protect cells against hyperosmolarity stress while inhibiting SG formation without affecting stem cell self-renewal and pluripotency. Our observations may provide a possible cellular mechanism to better understand SG dynamics in pluripotent stem cells.

## 1. Introduction

The exposure of cells to different external stresses (chemical hypoxia, heat shock, UV, and viral infection) induces the immediate formation of transient ribonucleoprotein (RNP) complexes in the cytoplasm called stress granules (SGs) [1]. These granules are initially nucleated by the aggregation of selected unnecessary messenger RNAs (mRNAs), translation factors, and RNA-binding proteins (RBP) into small mRNP complexes in the cytoplasm. As the recruitment of those complexes increases over time, the granules' size increases to form large structures of about 100–200 nm. Once the stress is relieved, SGs are disassembled and the mRNA is released back to the cytoplasm for translation [2]. Indeed, this scenario reflects the dynamic nature of SGs and explains this reversible process, which stimulates several cellular pathways that can contribute to physiological and/or pathological conditions. It has been well established that the formation of SGs is associated with the inhibition of translation initiation and polysome disassembly [3]. Translation inhibition and SG formation are often coupled to the phosphorylation of eukaryotic translation initiation factor 2 alpha (eIF2*α*) or eukaryotic initiation factor-4A (eIF4A) inhibition [4–6]. However, a recent study argues that localization to SGs does not prevent mRNA translation as commonly believed and that mRNAs can be observed by single-molecule imaging transitioning between the cytoplasm and SGs [7]. Several proposed functions have been associated with SG formation, of which cell survival through mRNA stabilization, sequestration, and protection stand out as the essential functions [8–10].

The first cellular component to be recruited to SGs is G3BP1 (RasGAP SH3 domain-binding protein), which belongs to a family of RNA-binding proteins (BP) and is considered as the seed needed to initiate SG formation when cells are stressed with arsenite [1, 11], hydrogen peroxide (H_2_O_2_) [12], or high temperature [13, 14]. Thus, G3BP is known as a robust SG marker. Moreover, the aggregation of the TIA-1 protein promotes the formation of SGs in a concentration-dependent manner that is inhibited by chaperones and is protease resistant [15]. Besides G3BP and TIA-1, several other markers are recruited to SGs such as TIAR, PABP, eIF4E, eIF4G, eIF3, and P-eIF2 [2]. Microtubules have also been proposed to play a key role in SG formation, as microtubule regulation is crucial for forming cell extensions in many cell types [16]. This hypothesis is because the alteration of the microtubules with nocodazole chemicals blocks the SG assembly [10, 17–19].

Different types of cells have shown different mechanisms to respond to stress and the formation of SGs [20]. Few studies have examined and attempted to identify the SG formation in human-induced pluripotent stem cells (hiPSCs) [13, 21], which can undergo self-renewal and differentiation into specialized cell types [22]. Our group has recently demonstrated that stressors like sodium arsenite and thermal stress induce SG formation in hiPSCs and provoke a downregulation of pluripotency marker expression.

Osmotic homeostasis is essential for the normal function of cells exposed to biophysical factors, including hyperosmolarity or stressful osmotic environments under physiologic or pathological conditions. Hyperosmotic stress is an often overlooked process that potentially contributes to several human diseases. Under physiological conditions, the mammalian kidney's inner medullary kidney exposes cells to high extracellular osmolarity due to the urine concentration. For this reason, renal cells have developed many adaptive strategies to compensate for increased osmolarity. The cytoprotective mechanisms and associated regulatory pathways help cells to confront and hyperosmotic conditions [23]. While the effects of hyperosmolarity have been extensively studied in adult cultured cells, demonstrating changes in cell shape, nutrient transport, proliferation, and growth [24–27], little is known on its effects on the properties and fate of iPS cells in *in vitro* conditions mimicking physiological or pathological conditions [28]. The cells' equilibrium osmolarity is one of the most tightly controlled physiological parameters, regulated by a balance of hydration and solute concentrations [29].

We investigated the response of pluripotent stem cells and differentiated cells towards hyperosmotic stress in the present work. We have performed a comprehensive proteomic analysis, and we have unveiled a surprising result showing that a reduced expression of tubulin could protect cells against hyperosmotic stress while inhibiting SG formation without affecting stem cell self-renewal and pluripotency. Furthermore, our data shed light on the possible implications of microtubule organization in this stress response program. These observations may provide a cellular mechanism to understand better how SGs regulate pluripotent stem cells' survival under stress conditions.

## 2. Materials and Methods

### 2.1. Cell Culture

Human-induced pluripotent stem cell lines (hiPSCs/IMR90-1) were purchased from WiCell Research Institute. For cell attachment, cells were cultured on feeder-independent conditions using the Matrigel matrix (from Corning™). hiPSC colonies were grown in StemFlex™ Medium (from Thermo Fisher Scientific) and maintained in a humidified atmosphere incubator with 5% CO_2_ at 37°C. For cell passage, cell colonies of hiPSCs/IMR90-1 were dissociated to aggregates using a nonenzymatic reagent (ReLeSR; STEMCELL Technologies). The human neuroblastoma SH-SY5Y cell line was obtained from the American Type Culture Collection (ATCC) (http://www.atcc.org). SH-SY5Y were cultured and maintained in Dulbecco's modified Eagle's medium F12 (DMEM F12) (Invitrogen) supplemented with 10% FBS (Sigma-Aldrich) and maintained in an incubator with 5% CO_2_ at 37°C.

### 2.2. Osmotic Stress Treatment Using NaCl

hiPSCs/IMR90-1 and SH-SY5Y were cultured generally 96 h and 24 h, respectively, before treatment with NaCl. When the confluency reached about 80%, cells were exposed to hyperosmotic stress by instantly replacing the medium with the hyperosmotic medium of different concentrations of NaCl: 50 mM, 100 mM, 200 mM, 300 mM, and 400 mM, during the specific incubation period, and maintained in an incubator at 37°C and 5% CO_2_.

### 2.3. Antibodies

Mouse monoclonal antibody of *β*-actin was purchased from Santa Cruz. Mouse monoclonal antibodies to detect the pluripotent markers Lin28a, Nanog, Oct4, and Sox2 were purchased from Cell Signaling. Mouse and rabbit monoclonal antibodies of G3BP were purchased from BD Biosciences. Anti-alpha tubulin antibody (ab7291) and anti-beta-tubulin antibody were purchased from Abcam and Thermo Fischer Scientific, respectively. Anti-mouse and anti-rabbit secondary antibodies conjugated with horseradish peroxidase (HRP) were from Cell Signaling. Alexa Fluor 488, 555, and 647 conjugated secondary antibodies were purchased from Thermo Fisher Scientific.

### 2.4. Immunocytochemistry

In a 24-well plate for hiPSCs/IMR90-1, coverslips were pretreated with Matrigel for 30 minutes and cells were cultured as mentioned above until they reached ~80% colony confluency, generally after about three days of passage. SH-SY5Y were cultured the day before on coverslips. After hyperosmotic treatment with NaCl, we followed the immunocytochemistry procedures that have been described previously [13]. Briefly, the medium was removed and cells were washed with PBS and fixed in 4% paraformaldehyde for 30 minutes at room temperature (RT). Then, cells were washed three times with TBST (TBS with 0.2% Tween), 10 minutes each wash. Fixed cells were then permeabilized using PBST (PBS with 0.2% Triton) for 10 minutes, washed three times with TBST, and incubated for 1 hour in blocking buffer (5% HyClone™ Donor Equine Serum in PBS) at RT. After blocking, cells were incubated for 2 hours at RT or overnight at 4°C with specific primary antibodies and diluted in the blocking buffer; a mouse monoclonal antibody to G3BP to detect stress granules was purchased from BD Biosciences. Mouse and rabbit monoclonal antibodies to detect the pluripotent markers, SOX2, OCT4, NANOG, and LIN28A were from Cell Signaling. Then, cells were washed 3 times with TBST, stained with appropriate anti-mouse and anti-rabbit Alexa Fluor secondary antibodies 488-, 555-, and 647-conjugated (Thermo Fisher) for 1 h at RT, and then washed three times with TBST. Finally, 0.5 *μ*g/ml Hoechst 33258 dye (Molecular Probes; 1 : 30000 dilution in PBS) was added to the cells for 3 minutes to allow nuclear staining. All immunocytochemistry steps were performed with continuous shaking on a microtiter shaker. Coverslips were mounted using Mowiol (poly(vinyl alcohol)) mounting medium, and the cells were observed and photographed with Axio ZEISS fluorescence microscope using 20x, 40x, and 100x objectives.

### 2.5. Stress Granule Quantification

Approximately 1000 cells were counted (distributed over 20 different randomly selected fields within each coverslip). Cells were counted manually, where a cell containing stress granules was identified as the one that has ≥3 distinct and clear granules. Those cells are scored positive cells, whereas others with less than 3 defined granules are scored negative. Moreover, we zoomed the image to be able to distinguish between cells during counting. In addition, dead cells or morphologically differentiated cells were excluded from the count. ImageJ/Fiji software program was further used to confirm the stress granule-positive cell count using the “analyze particles” ImageJ function. In addition, the Analyze Plugin for the Measure and Label function was used. The percentages of cells with SGs were quantified by counting the positive cells over the total number of cells in 20 different independent fields. Data was calculated from three independent experiments and presented as mean ± S.E.M.

### 2.6. Assessments of Cell Survival, Apoptosis, and Death: Annexin V and PI Assay

Treatment with gradient concentrations of NaCl (50 mM, 100 mM, 200 *μ*M, and 400 mM) was performed when cells reached about 80% confluence for 1 hour in an incubator at 37°C and 5% CO_2_. For flow cytometry analysis, cells were harvested and stained with 5 *μ*l Alexa Fluor® 488 annexin V (Thermo Fisher Scientific) in 500 *μ*l specific binding buffer and 1 *μ*l propidium iodide for 10 min at RT in the dark and BD Accuri™ C6 flow cytometer were used (Becton-Dickinson) for running the samples. Data were analyzed using FlowJo software (Tree Star Inc.)

### 2.7. Sample Preparation for Mass Spectrometry

For each experiment and each cell line, we had included 3 technical triplicated per condition (NT, 200 mM and 400 mM of NaCl treatment). Each experiment was repeated 3 independent times. Therefore, 27 samples representing the repetition above were sent for mass spec from each cell line of hiPSCs/IMR90-1 cells. Cells were washed with PBS and lysed with 200 *μ*l 4% SDS, supplemented with Benzonase (stock 250 unit/*μ*l), 1x protease inhibitor (Roche complete EDTA-free protease inhibitor cocktail tablets, GmbH, Germany), and 1x phosphatase inhibitor (Sigma-Aldrich, PhosSTOP, Roche, Chemie GmbH, Germany). After incubation for 40 min at 4°C, cells were sonicated in a water bath for 15 min and cleared by centrifugation at 15000 rpm. Protein concentrations were determined using the Pierce BCA assay kit (Thermo Fisher Scientific, Rockford, IL). According to the manufacturer, a concentration of 10 *μ*g of total protein was used for protein digestion and purification using the PreOmics sample preparation kit (PreOmics GmbH, Planegg, Germany) protocol. Clean peptides were dried in speed-vac and resuspended in 9 *μ*l of LC-loading buffer (mobile phase A), and 1x iRT peptides (Biognosys, Schlieren, Switzerland) were added for chromatography retention time normalization. The number of technical replicates is 3 each.

### 2.8. Mass Spectrometry Measurements

An EASY nLC-1200 (Thermo Scientific) coupled to a Q Exactive HF (Thermo Scientific) was used for mass spectrometric analyses. For data-dependent acquisition, 6.0 *μ*l of peptide solutions was injected in randomized order into the LC system for separation. Further details on instrumentation, LC columns, and analysis parameters can be found in Supplementary Table S1.

### 2.9. Proteomics Data Analysis

FT-MS raw data from hiPSCs/IMR90-1 and SH-SY5Y cells were analyzed separately using MaxQuant v. 1.6.10.43 with the integrated Andromeda database search engine [30, 31]. For peptide identification, enzyme specificity was set to trypsin with a maximum of 2 missed cleavages. Carbamidomethyl cysteine was set as a fixed modification, and oxidized methionine and protein N-acetylation were set as variable modifications. The MS and MS/MS tolerance were specified as 5 ppm and 0.5 Da, respectively. The peptide false discovery rate (FDR) was estimated by a target-decoy search strategy. The required peptide FDR and the required protein FDR were set to 0.01, with the minimum required peptide length of 7 amino acids. Both requantify and match between runs (match time: 0.7 min and alignment time window: 20 min) features were enabled. Protein sequence searches were performed against the reviewed Swiss-Prot *Homo sapiens* database (April 2018 release; 20365 protein sequence entries) appended with sequences of 245 common protein contaminants. Protein quantification was performed using the label-free quantification algorithm MaxLFQ [32] implemented in MaxQuant.

MaxQuant results containing protein identification and normalized label-free protein intensity were further analyzed using R software version 3.6.1 (R Project for Statistical Computing, Vienna, Austria) including standard packages. Statistical analysis and calculation of protein ratios were performed on log_2_-transformed protein intensities using the limma package [33]. Proteins with single-peptide identifications were not considered for statistical analysis. False-discovery rate adjustment of *p* values was done using the Benjamini-Hochberg method. Standardized log-ratios were used for possibilistic fuzzy c-means clustering using the *fcm* function from the ppclust package [34]. Functional overrepresentation analysis of Gene Ontology terms was performed using clusterProfiler [35] comprising semantic similarity filtering (similarity threshold 0.7) to reduce redundancy.

Mass spectrometer raw output files and MaxQuant search results are deposited at MassIVE repository (doi:10.25345/C51487).

### 2.10. Bioinformatics Pathway Analysis Using Ingenuity Pathways Analysis (IPA)

Data were analyzed using IPA (QIAGEN Inc., https://www.qiagenbioinformatics.com/products/ingenuitypathway-analysis). Ingenuity Pathway Analysis (IPA) software (Ingenuity Systems, CA) was used to further investigate the functional aspects of the identified proteins listed in Supplementary Table S2. The putative interactions and new functional networks associated with all identified proteins were analyzed using the software's statistical significance (FDR ≤ 0.05).

### 2.11. Immunoblotting

Protein extracts were collected by lysing the cells using RIPA lysis buffer (Sigma) combined with 1% Halt™ Protease and Phosphatase Inhibitor Cocktail, EDTA-free (100x) (Thermo Fisher Scientific). Protein concentrations were determined using Pierce™ BCA Protein Assay Kit (Thermo Fisher Scientific).

Protein extracts (20 *μ*g) were separated by 10% SDS-PAGE in a reducing condition using 5% *β*-mercaptoethanol, transferred into a nitrocellulose membrane, blocked with 5% nonfat milk in Tris-buffered saline (TBS) containing 0.1% (*v*/*v*) Tween 20, and incubated with primary antibodies overnight at 4°C and HRP-conjugated secondary antibodies for 1 h at room temperature. The proteins were detected using SuperSignal West Femto Maximum Sensitivity Substrate (Thermo Fisher Scientific) and developed with ChemiDoc™ MP Imaging System from Bio-Rad. Housekeeping *β*-actin protein was used as a loading control, and the density of bands on a Western blot image was analyzed using the ImageJ software.

### 2.12. Statistical Analysis

The significance level of all statistical tests was set to a *p* value or FDR-adjusted *p* value. Differences between pairs of data for the same time point were analyzed by either paired *t* test or Mann–Whitney test. All statistical calculations were performed using the GraphPad Software Prism 8 (GraphPad, San Diego, CA, USA).

### 2.13. Data Availability Statement

All data are contained within the manuscript except mass spectrometer raw output files and MaxQuant search results which are deposited at MassIVE repository (doi:10.25345/C51487).

## 3. Results

### 3.1. Gradient Concentrations of NaCl Showed a Different Pattern of SG Formation between hiPSCs/IMR90-1 and SH-SY5Y Cell Lines Lacking Pluripotent Properties

**Figure 1:**
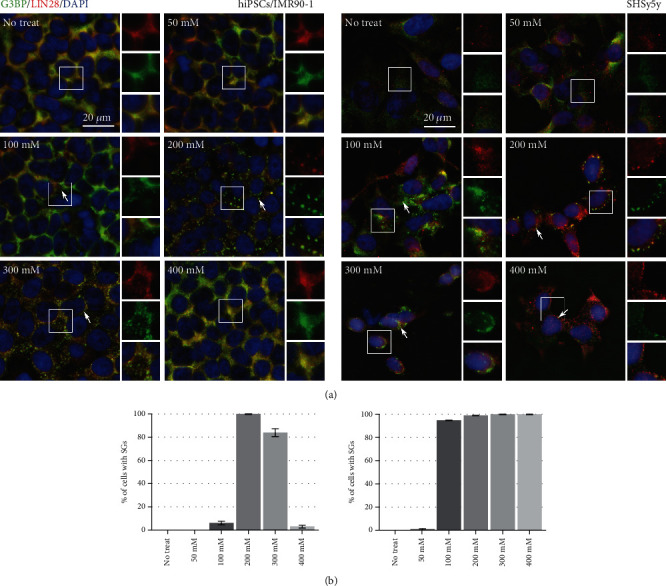
SG assembly in hiPSCs/IMR90-1 and SH-SY5Y under hyperosmolarity stress. (a) Representative fluorescence microscopy images showing nontreated hiPSCs/IMR90-1 and SH-SY5Y cells treated with 50, 100, 200, 300, and 400 mM of sodium chloride stained with the robust SG marker (G3BP (green)). LIN28 is an RNA-binding protein involved in promoting pluripotency. Nucleus is stained in blue (Hoechst). Insets show magnified views of SGs. White arrows indicate SGs. Scale bar indicates 20 *μ*m. (b) Percentage of hiPSCs/IMR90-1 with G3BP-positive SGs after 1 h treatment with the indicated concentrations of sodium chloride (50, 100, 200, 300, and 400 mM). The average percentage of cells with SGs is shown. Error bars indicate the ±standard deviation from 3 independent experiments. Approximately, 1000 cells were counted (distributed over 20 different fields within each coverslip).

To evaluate the effect of osmotic stress on SG formation on pluripotent stem cells (PSCs), we initially determined the effect of hyperosmolarity of NaCl on the commercial hiPSCs/IMR90-1 and compared it to SH-SY5Y that does not have pluripotent characteristics. The cells were cultured in the absence or presence of 50, 100, 200, 300, and 400 mM of NaCl for 1 hour, and SGs were quantified using the SG markers, G3BP and LIN28. LIN28 was also used as a stem cell marker to ensure that the cells still preserved their stemness properties. The results showed SG formation in both types of cells, which increases with increasing concentration. On the other hand, 400 mM treatment of NaCl resulted in an absence of SGs in hiPSCs/IMR90-1 but not SH-SY5Y cells. In addition, under all treatment's conditions, hiPSCs/IMR90-1 maintained their morphology, whereas distinct morphological changes such as noticeable membrane shrinking and cell rounding were observed in SH-SY5Y cells (Figure 1). To ensure that the treated hiPSCs/IMR90-1 maintained pluripotency under all conditions, cells were stained with different pluripotent markers (Nanog, Oct4, and Sox2). All treated cells showed similar expression levels of the three markers, even with a high concentration of NaCl (400 mM) (Supplementary Fig. 1). Moreover, we have used other well-known SG markers, LIN28, TIA-1 and TIAR, to confirm that the composition of these granules in hiPSCs/IMR90-1 is SG per se (Supplementary Fig. 2A, 2B and 2C).

To confirm that the SG assembly and disassembly phenomenon observed with NaCl treatment in hiPSCs/IMR90-1 is caused by hyperosmotic stress, we subjected the cells to sorbitol, which is another physiological stressor known to induce hyperosmolarity. Sorbitol stress was used at concentrations of 200 and 400 mM for a one-hour incubation period. Similar to NaCl treatment, SGs were only formed with the 200 mM concentration of sorbitol but not with the highest concentration (400 mM) (Supplementary Fig. 3). This data indicates that SG assembly in hiPSCs/IMR90-1 depends on the concentration of hyperosmotic stress, a phenomenon that was not seen in SH-SY5Y cells that lack pluripotent properties.

Moreover, to support the claims of differences between stem cell and differentiated cell lines, two different types of pluripotent stem cell lines with uncommon genetic backgrounds, the human-induced pluripotent stem cell (hiPSCs/CRTD5 [36] and human embryonic stem cell line H9, have been treated with NaCl (200 mM and 400 mM) and have shown similar results as the hiPSCs/IMR90-1 cell line. SG formation was only shown at 200 mM of NaCl treatment (Supplementary Fig 4). On the other hand, two different types of nonpluripotent cell lines, BHK-21 and the MCF-7, have been tested and have shown similar results to those observed with SH-SY5Y cell lines that also lack pluripotent characteristics. Both cell lines showed SG formation at 200 and 400 mM of NaCl treatment (Supplementary Fig. 5).

SG formation is known to be formed either dependent [2] or independent on eIF2*α* phosphorylation [12]. It has been previously described that NaCl treatment does not require eIF2*α* phosphorylation to inhibit translation and induces the assembly of poly(A)-positive cytoplasmic foci that compositionally resemble canonical SGs (Kedersha, [9, 20]) in the near-haploid human cell line (HAP1). To confirm if the NaCl granules formed in hiPSCs/IMR90-1 also do not require eIF2*α* phosphorylation, we used immunoblotting analysis to analyze the phosphorylation of eIF2*α* in cells treated with 200 mM (a concentration that induced SG formation) and 400 mM (a concentration that did not induce SG formation) NaCl. No eIF2*α* phosphorylation was detected in nontreated cells or cells incubated with 200 mM NaCl (Supplementary Fig. 6). In contrast, at 400 mM treatment, a significant induction of eIF2*α* phosphorylation was observed (Supplementary Fig. 6). These data indicate that different concentrations of NaCl showed different patterns of SG formation and eIF2*α* phosphorylation in hiPSCs/IMR90-11 and that NaCl-SG formation is independent of eIF2*α* phosphorylation in those cells.

### 3.2. Time Course Experiment Showed No SG Formation in hiPSCs/IMR90-1 under 400 mM of NaCl Treatment

**Figure 2:**
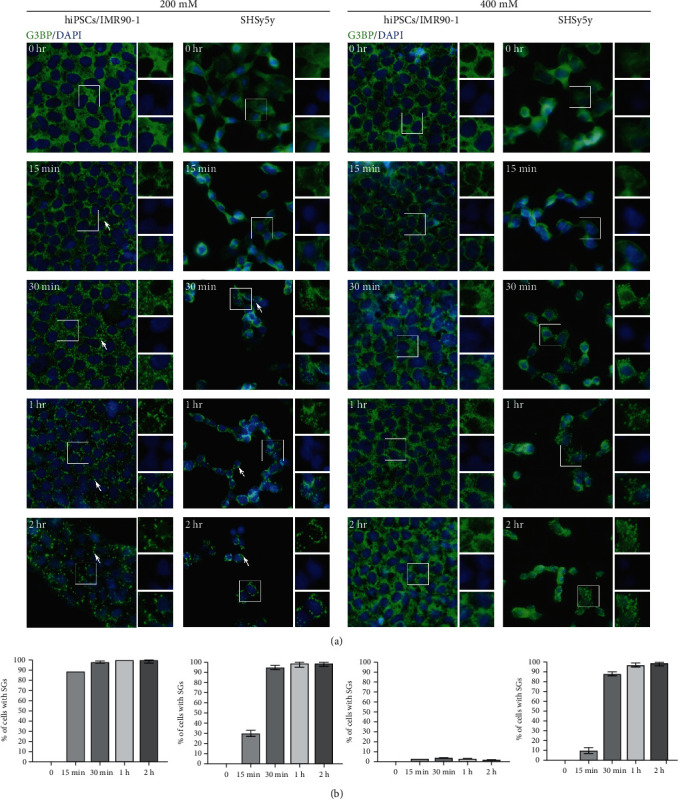
Time-course experiments to ensure that SG assembly in hiPSCs/IMR90-1 and SH-SY5Y is time independent. (a) Representative fluorescence microscopy images showing IMR90-1/hiPSCs and SH-SY5Y treated with 200 and 400 mM of sodium chloride stained with the robust SG marker (G3BP (green)). Nucleus is stained in blue (Hoechst). Insets show magnified views of SGs. White arrows indicate SGs. Scale bar indicates 20 *μ*m. (b) Percentage of hiPSCs/IMR90-1 with G3BP-positive SGs during the time-course treatment with the indicated concentrations of sodium chloride (0, 200, and 400 mM). The average percentage of cells with SGs is shown. Error bars indicate the ±standard deviation from 3 independent experiments. Approximately, 1000 cells were counted (distributed over 20 different fields within each coverslip).

To determine if the absence of SGs in hiPSCs/IMR90-1 under 400 mM NaCl treatment is time dependent, hiPSCs/IMR90-1 and SH-SY5Y cells were left untreated or treated with 200 mM and 400 mM of NaCl at different times (15 min, 30 min, 1 h, and 2 h) and SG formation was detected by fluorescence microscopy using G3BP as a marker. SGs started to appear 30 min after treatment with 200 mM NaCl in both hiPSCs/IMR90-1 and SH-SY5Y cells (Figure 2(a)). As the stress time increased to reach up to two hours, SGs enlarged in size and thus appeared more defined and more precise. The same pattern was seen with SH-SY5Y cells treated with 400 mM of NaCl. In contrast, in hiPSCs/IMR90-1 treated with 400 mM NaCl, no SG formation was detected even after 2 hrs of treatment (Figure 2(b)). These observations suggested that the absence of SGs in hiPSCs/IMR90-1 is time independent; however, it is associated with the elevated concentration of hyperosmotic stress.

### 3.3. hiPSCs/IMR90-1 Induced Low Apoptosis Levels under NaCl Treatment

**Figure 3:**
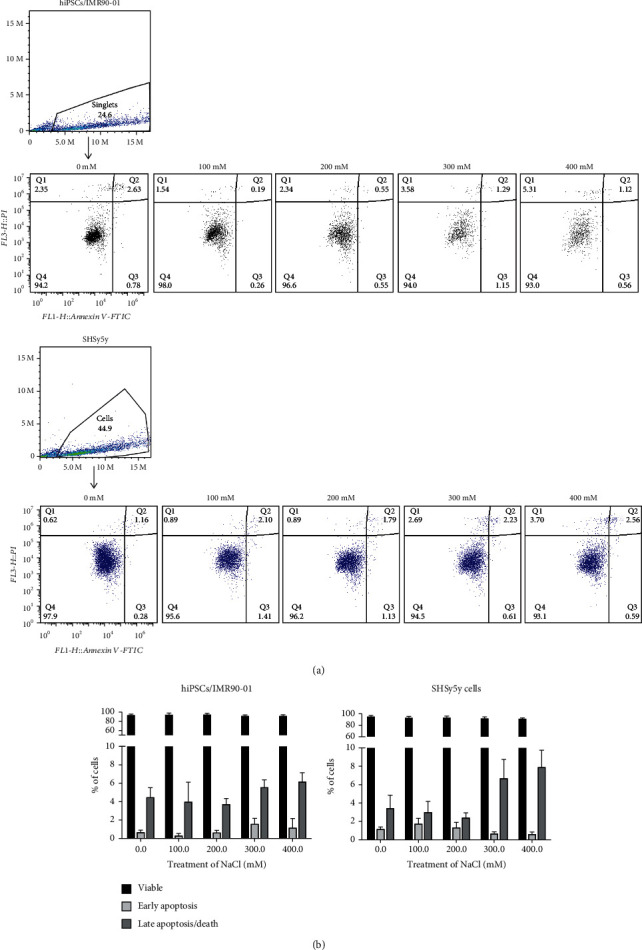
The flow cytometric (FACS) analysis diagram of NaCl for hiPSCs/IMR90-1 and SH-SY5Y. (a) Representative flow cytometric multicolor gating of cells used to analyze the number of live/dead cells and representative profiles of FACS analysis after 1-hour treatment with NaCl. Cells were collected after 1-hour treatment with different concentrations of NaCl, stained with Annexin V and PI, and analyzed by FACS. Cells without NaCl treatment were used as negative controls (*n* = 4 in each group). The diagram can be divided into four regions that are defined as follows: the percentage of necrotic cells (Q1; PI/FITC +/−), the percentage of late apoptotic cells (Q2; PI/FITC +/+), the percentage of viable cells (Q3; PI/FITC −/−), and the percentage of early apoptotic cells (Q4; PI/FITC −/+). (b) Percentages of viable, early, and late apoptosis are presented in the graphs for both types of cells. Results represent four independent experiments with similar results.

To identify whether cell death, apoptosis, or survival signaling pathways are involved in the assembly/disassembly of SGs at different NaCl concentrations, in-house annexin V and propidium iodide (PI) assays were used. We have used both PI and annexin to detect early (EA) and late (LA) stages of apoptosis. In the EA stage, PI cannot stain cells with the intact cell membrane; however, Annexin V can stain phosphatidylserine externalization, an indicator of membrane instability and apoptosis. Hence, cells in early apoptosis are Annexin V positive and PI negative. In contrast, in LA or cell death, the damaged cell membranes can allow Annexin V and PI to enter into cells. In this case, cells are both Annexin V and PI positive. When we compared EA and LA events in hiPSCs/IMR90-1 treated with different NaCl concentrations (100, 200, 300, and 400 mM) with the nontreated condition, we did not find any significant differences in the numbers of apoptotic cells (ranged from 0.26 to 2.30% in EA and 1.73 to 7.2 in LA). In contrast, a gradual increase in LA in SH-SY5Y cells correlates well with increasing NaCl concentrations, where 400 mM of NaCl showed the highest number of dead cells, 9.8% (Figure 3(b)). Surprisingly, we did not observe significant differences in EA or LA between hiPSCs/IMR90-1 and SH-SY5Y (Figures 3(a) and 3(b)). These results indicate that under these conditions of hyperosmotic stress, the apoptotic pathway is not induced in hiPSCs/IMR90-1. To exclude any possibility that apoptosis is taking place at 400 mM NaCl treatment, where no SGs were detected, we employed another approach to further corroborate apoptosis. Western blot analysis was utilized to assess caspase activation in hiPSCs/IMR90-1 by detecting caspase-3 cleavage. Consistent with the flow cytometry results that showed a meager percentage of apoptotic cells, we did not observe a clear activation of caspase 3 in hiPSCs/IMR90-1 under 400 mM of NaCl treatment. However, the 200 mM treatment had only subtle effects on the cleavage of caspase-3 (Supplementary Fig. 7). Although these results are not consistent with the flow cytometry data, the caspase 3 cleavage was subtle compared to the no treatment and the 400 mM of NaCl treatment. Overall, these results indicate that the absence of SGs in hiPSCs/IMR90-1 under 400 mM of NaCl treatment is not due to induction of apoptosis.

### 3.4. Proteomic Analysis Revealed the Presence of Differentially Expressed Microtubule Proteins in hiPSCs/IMR90-1 under Hyperosmotic Stress

Our results showed a specific pattern of hyperosmotic SGs in hiPSCs/IMR90-1, which is different from the other cell lines; therefore, it was of our interest to identify putative proteins that may play a role in producing this specific phenomenon. Mass spectrometry- (MS-) based proteomics was carried out on hiPSCs/IMR90-1 and SH-SY5Y in nontreated cells and cells treated with 200 mM (a condition that showed SGs in both cell types) and 400 mM (a condition that showed SGs only in SH-SY5Y cells) of NaCl. Proteins are differentially illustrated in a volcano plot (Supplementary Fig. 8) in line with the statistical −log^10^*p* value shown in the *y*-axis and the ratio log_2_ fold change in protein-relative abundance as shown in the *x*-axis between nontreated (NT) cells compared to NaCl treatment in hiPSCs/IMR90-1 (Supplementary Fig. 8A) and SH-SY5Y (Supplementary Fig. 8B). A 2-fold (*p* value ≤ 0.05) cutoff was used to sort out statistically significant regulated proteins in both cell lines under these conditions. The total number of quantified proteins in the study were 3729, and in each cutoff region, the number of proteins was indicated in the left and right corners. Only proteins with a log-fold change cutoff (log-FC) smaller than −0.58 or larger than 0.58 (corresponds to 0.67/1.50-fold change) and applying significance cutoff *p* < 0.05, FDR < 0.05, and bonf < 0.05 were considered (Supplementary Table S2). In hiPSCs/IMR90-1, a total of 1166 proteins were quantified with at least one technical replicate in all three conditions (NT, 200 mM, and 400 mM) (Supplementary Table S3). However, in SH-SY5Y, the quantified proteins were accumulated to 860 proteins (Supplementary Table S3). Supplementary Table S4 shows the overlaps of identified proteins of quantitative comparative proteomics in hiPSCs/IMR90-1 and SH-SY5Y.

**Figure 4:**
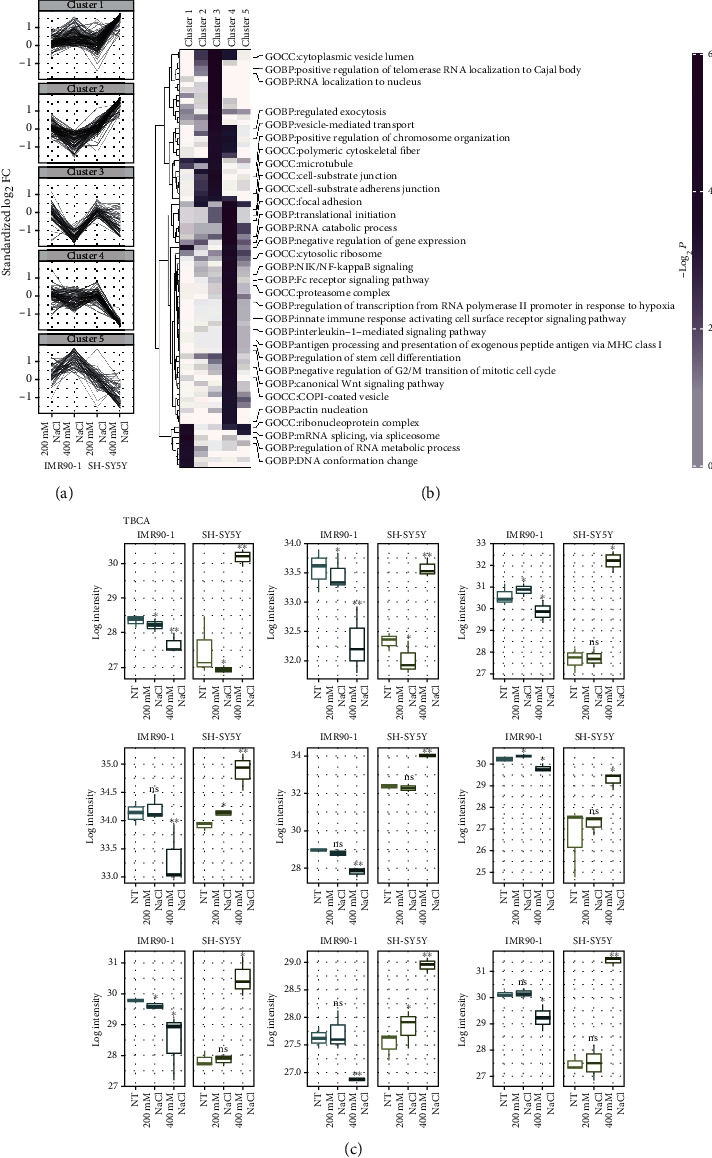
Clustering and functional overrepresentation analysis of differential proteomic data. (a) Fuzzy c-means clustering identifies distinct patterns in protein levels upon NaCl treatment in hiPSCs/IMR90-1 and SH-SY5Y cells. Clustering into 5 clusters was performed to standardized changes in protein levels when compared to untreated cells (*p* < 0.05). Profile plots of the clusters indicate the following general states: 1 and 2, strongly increased expression in 400 mM in hiPSCs/IMR90-1; 3, moderate increase expression in 400 mM in hiPSCs/IMR90-1; and 4 and 5, decreased expressions in 400 mM in hiPSCs/IMR90-1. (b) Heatmap showing Gene Ontology (GO) terms which are statistically overrepresented within clusters as indicated by a high −log^10^*p* value. (c) Selected subtype markers across selected cluster 3 in hiPSCs/IMR90-1 and SH-SY5Y. Selected subtype markers exhibit the subtype-expected pattern (for each pairwise comparison, we used Student *t*-test, ^∗^FDR < 0.05). Microtubule proteins, TBCA, TUBB, STMN1, TUBA1B, TUBB4B, PKBP4, DYNC1H1, MAPRE1, and DPYSL2 were significantly lower in hiPSCs/IMR90-1 under 400 mM of NaCl treatment. NT: no treatment; ns: not significant compared to NT; ^∗^FDR: adjusted *p* value less than 0.05 compared to NT; ^∗∗^FDR: adjusted *p* value less than 0.01 compared to NT.

Secondly, the clustering algorithm, namely, fuzzy c-means, was used to classify and analyze the samples. The fuzzy c-means algorithm resulted in five clusters that best separated the samples. This was evaluated quantitatively using standardized changes in protein levels upon NaCl treatment compared to NT cells (*p* < 0.05) (Figure 4(a)). Profile plots of five selected clusters showing distinct behavior with respect to three states are shown: 1 and 2 indicate strongly increased expression in 400 mM in hiPSCS/IMR90-1, 1 and 3 indicate moderate increase in 400 mM in hiPSCs/IMR90-1, and 4 and 5 indicate decreased expression in 400 mM in hiPSCs/IMR90-1 (Figure 4(a)). To gain more biological insights into these clusters, we performed functional enrichment analysis using Gene Ontology (GO) annotation. This analysis showed that the most statistically overrepresented functional terms are in clusters 3 and 4. However, very few processes are significant in clusters 1, 2, and 5 as indicated in the heat map by a high −log^10^*p* value (Figure 4(b)). Selected subtype markers showed the subtype-expected pattern (for each pairwise comparison, limma^∗^FDR < 0.05 was used). Our results showed a specific expression of microtubule proteins, TBCA, TUBB, STMN1, TUBA1B, TUBB4B, PKBP4, DYNC1H1, MAPRE1, and DPYSL2 that were significantly lower in hiPSCs/IMR90-1 under 400 mM of NaCl treatment that did not show SG formation (Figure 4(c)) compared to those concentrations that induce SGs in either hiPSCs/IMR90-1 or SH-SY5Y cell lines. These results imply the involvement of microtubule proteins in regulating SG formation in hiPSCs/IMR90-1 under hyperosmotic stress.

### 3.5. Pathway Analysis Revealed the Involvement of the Microtubule Cytoskeleton Signaling Pathway in hiPSCs/IMR90-1 under 400 mM NaCl Hyperosmolarity Stress

**Figure 5:**
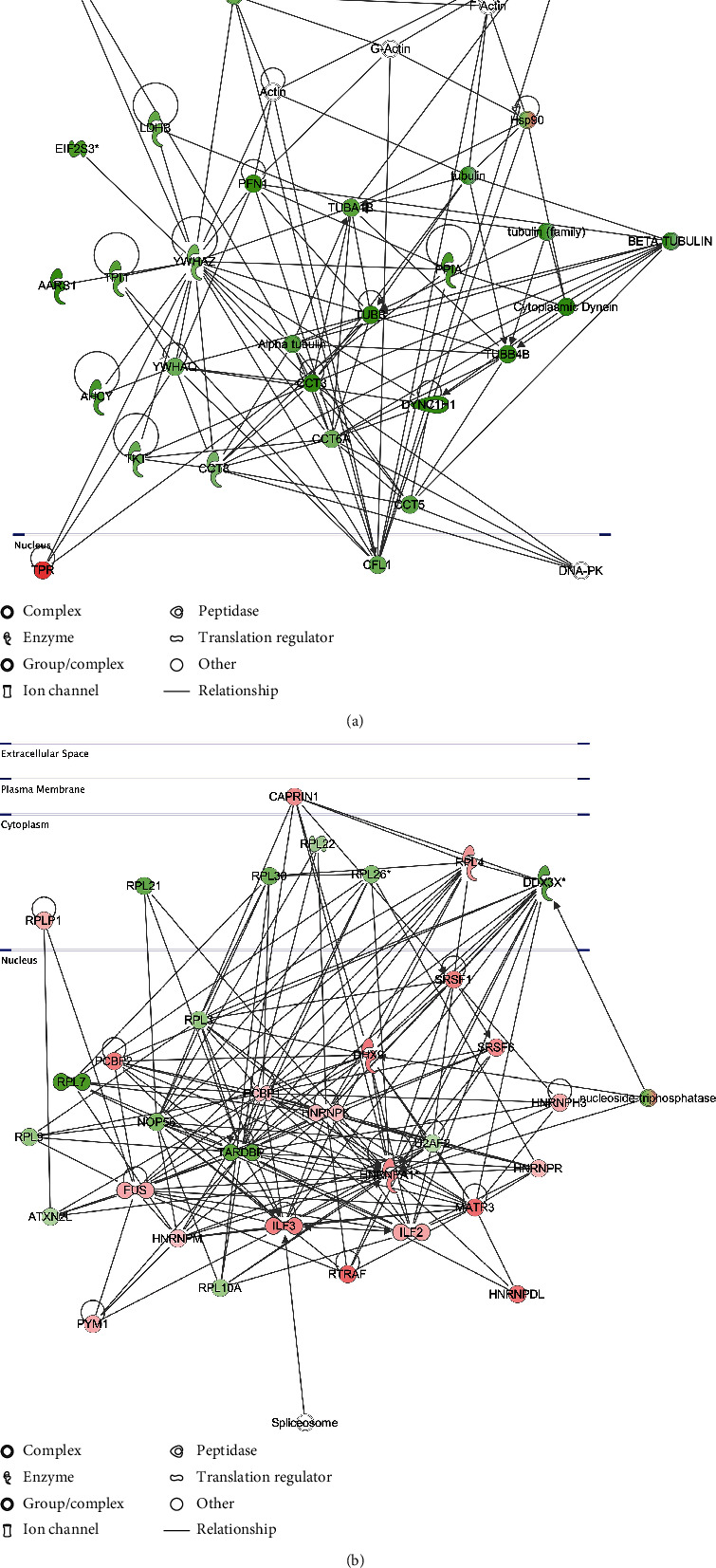
IPA network in hiPSCs/IMR90-1 and SH-SY5Y cells. (a) The top scoring IPA protein network for hiPSCs/IMR90-1 is “cell-to-cell signaling and interaction, cellular assembly and organization” and (b) in SH-SY5Y is “cellular assembly and organization, cell-to-cell signaling and interaction, reproductive system development and function,” both are depicted under the 400 mM NaCl treatment condition. The shapes represent the molecular classes of the proteins. Red represents upregulation, green represents downregulation, and color intensity represents the relative magnitude of change in protein expression. Interactions are indicated by solid lines. The protein interaction networks were generated through the use of IPA software.

We further employed an additional step to explore possible pathways that may be involved in regulating such distinctive proteomic profiles seen in Figure 4. The significantly identified proteins (FDR < 0.05) in hiPSCs/IMR90-1 and SH-SY5Y treated with 400 mM of NaCl were analyzed using QIAGEN Ingenuity Pathway Analysis (IPA). Our analysis revealed the top-scoring IPA protein network in hiPSCs/IMR90-1 as cell-to-cell signaling and interaction, cellular assembly and organization, reproductive system development, and function with a high score (50). This complex protein network is interconnected with tubulin (7 proteins), actin (4 proteins), chaperonin (CCT) (4 proteins), and other cytoskeleton proteins such as CAP1 (cyclase-associated actin cytoskeleton regulatory protein 1) (Supplementary Table S5; Figure 5(a)). On the other hand, the top-scoring IPA protein network in SH-SY5Y was protein synthesis, RNA damage and repair, and RNA posttranscriptional modification with a high score (66) as shown in Supplementary Table S5 and Figure 5(b). This complex protein network is interconnected with subunits of ribosomal proteins (RPL) (9 proteins), heterogeneous nuclear ribonucleoprotein (HNRN) (6 proteins), serine- and arginine-rich splicing (SRS) (2 proteins), and RNA-binding protein (3 proteins) such as FUS (cyclase-associated actin cytoskeleton regulatory protein 1) (Supplementary Table 4; Figure 5(b)). This indicates that the mechanism by which both types of cells survive and their ability to handle the hyperosmolarity stress is variable and different.

### 3.6. Altered Tubulin Expression Was Detected in hiPSCs/IMR90-1 under 400 mM NaCl Hyperosmotic Stress

**Figure 6:**
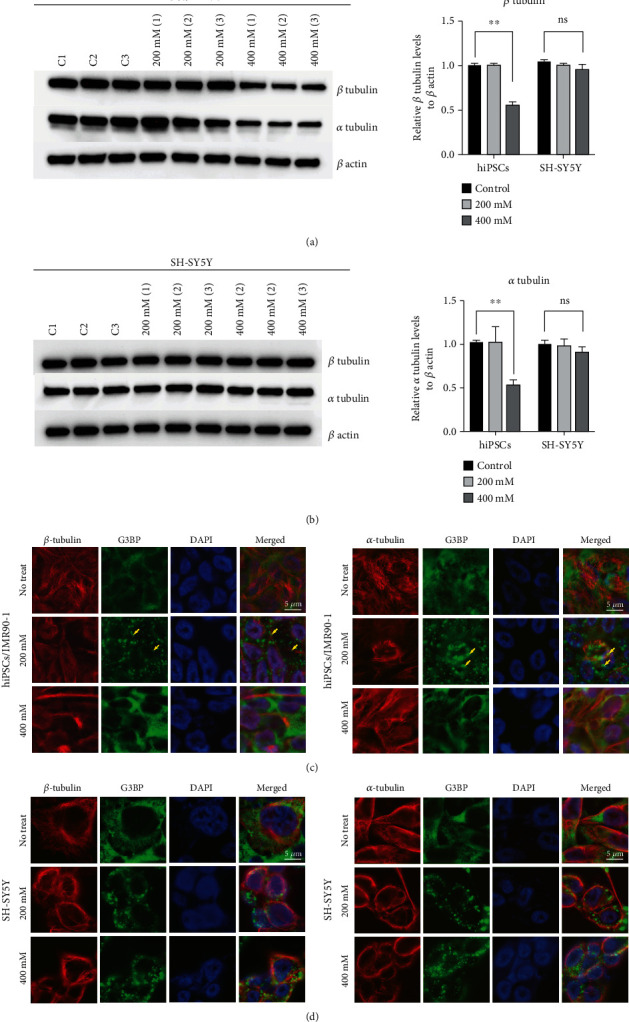
Downregulation of NaCl treatment alters tubulin expression and tubulin network of hiPSCs/IMR90-1. (a) Cells were treated with NaCl for 1 h and then lysed with RIPA buffer. The lysates were separated with SDS-PAGE, transferred onto PVDF membranes, and probed with anti-*α*-tubulin, anti-*β*-tubulin, and anti-*β*-actin antibodies. A representative immunoblot analysis in IMR90/iPSCs and SH-Y5Y cells is shown. (b) Intensity of each band of the immunoblot was measured by the NIH ImageJ program, and the ratios of tubulin and *β*-actin in each treatment was calculated. (c, d) Representative fluorescence microscopy images showing nontreated hiPSCs/IMR90-1 and SH-SY5Y, cells treated with 200 and 400 mM of sodium chloride stained with the robust SG marker (G3BP (green)), *β*-tubulin (c) and *α*-tubulin (d) (red), the largest of the cytoskeletal polymers forming microtubules. Nucleus is stained in blue (Hoechst). Insets show magnified views of SGs and microtubule filaments. Scale bar indicates 5 *μ*m. ns: not significant compared to NT; ^∗∗^*p* value less than 0.01 compared to NT.

Our proteomic analysis as well as IPA data showed a possible involvement of tubulin in regulating SG formation in hiPSCs/IMR90-1 cells (Figures 4 and 5). To validate these results, Western blot experiments were performed to detect the expression levels of *α*-tubulin and *β*-tubulin proteins that were drastically downregulated in hiPSCs/IMR90-1 treated with 400 mM NaCl but not SH-SY5Y cell lines (Figure 4). Protein lysates were prepared after 1 h treatment of NaCl, and WB was performed using *α*-tubulin, *β*-tubulin, and *β*-actin antibodies (Figures 6(a) and 6(b)). Tubulin bands were quantified after normalization to the *β*-actin control bands (Figures 6(a) and 6(b)). In agreement with our proteomic analysis data, there was a significant decrease in the expression of both tubulins in hiPSCs/IMR90-1 treated with 400 mM NaCl compared to that of untreated cells. However, tubulins were nearly unchanged in SH-SY5Y-treated cells.

Since disruption of microtubules has been reported to inhibit SG formation and dynamics in HeLa cells [37–39], it was of our interest to test the effect of NaCl treatment on microtubule organization and link it to SG dynamics. hiPSCs/IMR90-1 and SH-SY5Y cells were treated with 200 mM and 400 mM of NaCl for 1 h and processed for immunofluorescence using *α*-tubulin and *β*-tubulin antibodies. G3BP1 was used as a marker for stress granules and nontreated cells were used as a positive control. The results showed a partial microtubule destabilization after NaCl treatment in hiPSCs/IMR90-1 (Figures 6(c) and 6(d)), where the microtubule cage around the nucleus seemed to lose its intactness compared to the 400 mM condition and the nontreated cells. This data suggests that a high concentration of NaCl causes distorted microtubule framework in hiPSCs and confirms our proteomic analysis results that indicate the possible involvement of microtubules and significant components of tubulin in SG regulation in hiPSCs. To confirm these results, we tested the degree of tubulin stabilization under 400 mM NaCl-treated cells. We treated hyperosmotic stressed cells with taxane drug “paclitaxel,” one of the Taxane family known to stabilize microtubules as an effective agent against cancer [40]. However, the hiPSCs were remarkably sensitive to paclitaxel's cytotoxic effects indicated by the death of all treated cells. Therefore, this is a limitation of our study that might be the basis for further follow-up studies and exploration is needed to support these results.

## 4. Discussion

**Figure 7:**
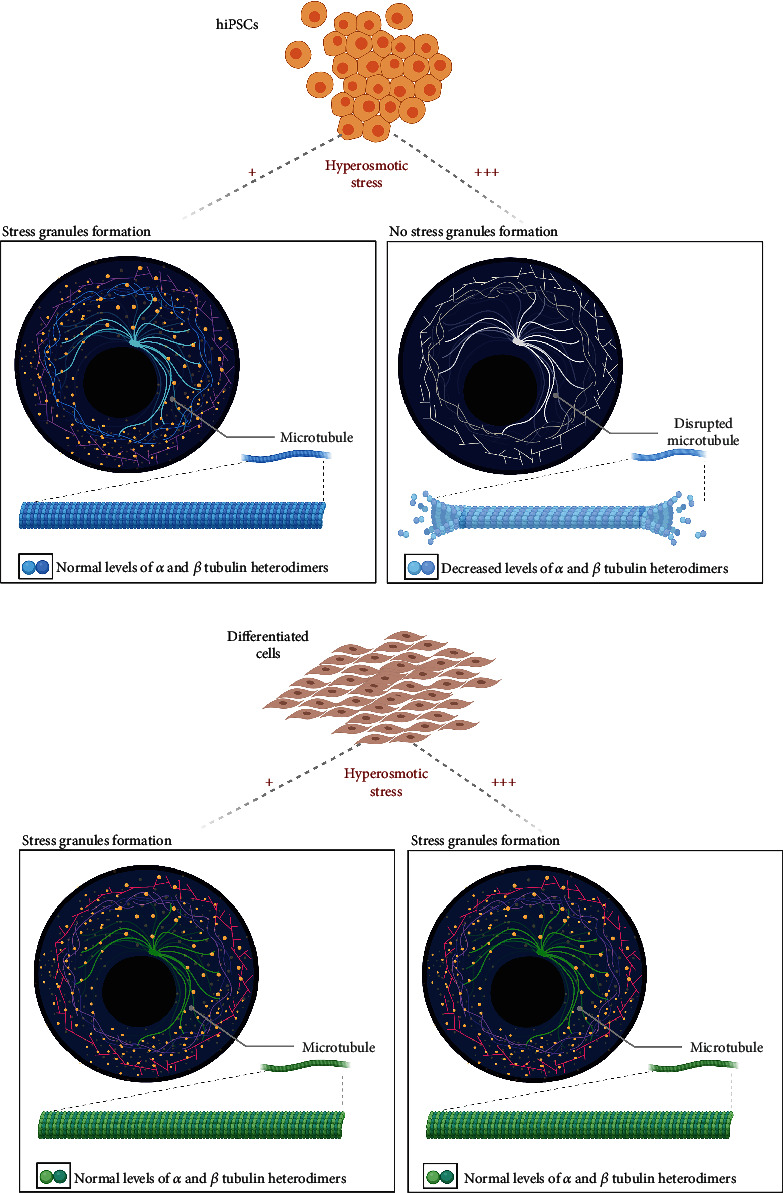
The summary figure of the study. This summarizes a cellular mechanism that controls the assembly and disassembly of SGs induced by hyperosmotic stress in hiPSCs/IMR90-1. Upon gradient concentrations of hyperosmolarity treatment, the effect of increased cell osmolarity differs from one type of cell to another. Under 200 mM of NaCl, hiPSCs/IMR90-1 and SH-SY5Y showed SG formation. However, with a higher concentration, 400 mM, SGs disappeared in hiPSCs/IMR90-1. Reduced expression of tubulin may protect cells against hyperosmolarity stress while inhibiting SG formation without affecting stem cell self-renewal and pluripotency. Possible implications of microtubule organization, dynamic structural cellular components, on the response to hypertonic stress in hiPSCs were found.

This study describes a cellular mechanism that controls the assembly and disassembly of SGs induced by hyperosmotic stress in hiPSCs. Upon gradient concentrations of hyperosmolarity treatment, the effect of increased cell osmolarity differs from one type of cell to another (Figure 7). Under 200 mM of NaCl, hiPSCs/IMR90-1 and SH-S5Y5 showed SG formation. However, with a higher concentration, 400 mM, SGs disappeared in hiPSCs/IMR90-1 but not SH-S5Y5. We have unveiled a reduced expression of tubulin that may protect cells against hyperosmolarity stress while inhibiting SG formation without affecting stem cell self-renewal and pluripotency. We found possible implications of microtubule organization, dynamic structural cellular components on the response to hypertonic stress in hiPSCs/IMR90-1. This and additional events might enhance survival pathways in hiPSCs, which have highly essential survival signaling pathways.

It is well established that SGs are dynamic reversible structures that assemble and disassemble according to the period of stress and type of cell treatment. Notably, the equilibrium for SG existence is balanced with polysomes [9, 39, 41, 42]. Under puromycin treatment, SGs are formed by polysome disassembly, whereas SG dissolution and/or dismantling are stimulated by cycloheximide (CHX) treatment, inhibiting translation elongation, and blocking of the polysome disassembly [43]. Interestingly, hyperosmotic stress was previously shown to stimulate stable SG-like structures that are not disassembled with CHX treatment [20, 44]. However, when cells are pretreated with CHX and then subjected to NaCl osmotic stress, no SGs are assembled (Kedersha, [9]). This indicates that hyperosmotic stress stimulates more stable SG structures compared to canonical SGs. Consistent with this data, we found that SH-SY5Y cells subjected to different concentrations of NaCl form stable SGs up to two hours of treatment even when the cells are apoptotic. However, this was not the case with hiPSCs/IMR90-1, which showed the disappearance of those granules under hyperosmotic stress stimulated by higher concentrations (400 mM) of either NaCl or sorbitol treatment (Figure 1 and Supplementary Figure 3), indicating that those granules behave differently in hiPSCs/IMR90-1 compared to cells harboring no pluripotent characteristics. We have previously shown that hiPSCs/IMR90-1 showed different responses in their ability to form SGs after being subjected to different types of stresses compared to other cell types [13]. Our previous data showed a selective canonical SG formation by SA and HS treatment, but not H_2_O_2_. Here, we reemphasize the same concept but on the concentration levels where hiPSCs could form SGs with low concentrations of NaCl and sorbitol but still survive and combat stress without SG formation under high concentrations of those treatments.

In general, the formation of SGs is mainly based on the aggregation of RNA and proteins to assemble what is known by mRNP complexes that is composed of different markers such as G3BP [9, 14], TIA-1, TIAR, eIF3b, eIF4E, PABP, and LIN28. Stress granule formation differs depending upon the nature of stress and cell type. It has been previously found that the composition and behavior of hyperosmotic SGs are partially different from those known for well-known and established SGs [44]. In this report, hyperosmotic stress granules in hiPSCs/IMR90-1 showed the major molecular components (G3BP, TIA-1, TIAR, and LIN28) as those observed in other types of cells (Supplementary Fig. 2). The formation and composition of iPSC hyperosmotic SGs may be different from other cell types, which affect the dynamics of those granules. If this is true, it would be our interest to dissect the other components of those granules and deeply investigate the mechanism of their accumulation under these conditions. Another crucial aspect of the formation of SGs is their induction dependently or independently of eIF2*α* phosphorylation under stress conditions. The formation of hyperosmotic granules was found to be independent of phosphorylation of eIF2*α* in different types of cell lines [44, 45] and may be formed due to hyperosmotic stress and molecular crowding [45].

Similarly, our data showed that in hiPSCs/IMR90-1 treated with 200 mM of NaCl, SGs are formed independently of eIF2*α* phosphorylation (Supplementary Figure 3). However, at a higher concentration of NaCl (400 *μ*M), marked phosphorylation of eIF2 alpha (Supplementary Figure 6) was observed in hiPSCs/IMR90-1, which coincides with the downregulation of tubulin under these conditions (Figure 6). The association of tubulin downregulation and phosphorylation of eIF2 alpha has been previously described in Leishmania's adaptive response to stress during its development [46]. Moreover, it has been previously shown that skeletal muscle stem cells, which do not induce eIF2*α* phosphorylation, exit quiescence and stimulate differentiation but do not self-renew [47]. hiPSCs/IMR90-1 may develop a specific unknown mechanism regulating self-renewal and differentiation through an alternative induction of SGs and eIF2*α* phosphorylation.

Previous studies have associated mRNP complex assembly and disassembly with microtubule proteins, and hence, SG formation is associated with microtubule cytoskeleton regulation [9, 17, 18, 37–39, 41, 42, 48–52]. Indeed, the requirement of microtubule integrity as an essential part of SG formation was confirmed by using microtubule-targeting drugs (MTDs), which destabilize microtubules and in turn interferes with SG formation and maturation [18, 37, 51, 53]. Microtubules are known as the largest cytoskeletal fibers made of tubulin. Tubulin is a globular protein that consists of two closely related 55 kd polypeptides, *α*-tubulin, and *β*-tubulin. Consistent with this, our proteomic, Western blot, and immunostaining data show microtubule destabilization and a significant decrease in *α*-tubulin and *β*-tubulin expression in hiPSCs/IMR90-1, but not in SH-SY5Y cells, that are treated with a high concentration of NaCl (400 mM) and did not induce SGs (Figures 6 and 7). In agreement with our data, high salt concentration (400 mM) and hyperosmolarity induce microtubule depolymerization in *Arabidopsis* seedlings [54–56]. Significantly, the destabilization of these microtubules is regulated by the propyzamide-hypersensitive 1 (PHS1) kinase that phosphorylates *α*-tubulin [57]. Further investigations are needed to investigate the mechanism of microtubule destabilization and SG inhibition under hyperosmotic stress in hiPSCs.

Another component that has been identified to be a core player in the regulation dynamics of SGs is the motor protein dynein, which binds to microtubules [48, 51, 52, 58]. The knockdown of dynein interferes with SG formation in mammalian cells [52]. The binding capacity of dynein to microtubules is guarded by high levels of ATP [59], which leads to the weak binding ability of the protein to microtubules [60]. Interestingly, hyperosmotic stress and mechanical shear stress were previously reported to induce high ATP levels *in vivo* and *in vitro* [20, 61]. In hiPSCs, high rates of glycolysis are reported, which is associated with high ATP production. Consequently, it is logical to assume that with the specific nature and characteristics of hiPSCs, both microtubules and motor protein dynein would be affected under hyperosmotic stress conditions, and the disruption of one or both would lead to the complete inhibition of SG formation in those cells. Further studies are needed to investigate this point deeply.

Although our data shed light on the possible role of microtubule in regulating SG formation under NaCl treatment in hiPSCs/IMR90-1, we cannot exclude the possibility that other cellular changes might contribute in such regulation. Since damaged mitochondria induce microtubule disassembly and reducing levels of energy metabolism and intracellular material transport [62, 63], we have sorted out all quantified mitochondrial proteins in the proteomic data (Supplementary Fig. 9). The total number of quantified mitochondrial proteins was three: COX7C (cytochrome c oxidase subunit 7C), SHMT2 (serine hydroxymethyltransferase), and ACO2 (aconitate hydratase). The expression of these proteins was significantly increased in hiPSCs/IMR90-1 under the 400 mM treatment of NaCl, which showed no SG formation compared to control and 200 mM treatment. In contrast, in the nonpluripotent SH-SY5Y cells treated with 400 mM NaCl, the expression of these proteins was reduced, specially COX7C that was significantly downregulated. Importantly, those mitochondrial proteins have been found to impair cell proliferation and commit cells to redirect pyruvate to mitochondria, excluding the possibility that microtubule destabilization is regulated by mitochondria damage after the treatment of 400 mM of NaCl in hiPSCs/IMR90-1. However, those proteins may play an important role in regulating stress granule formation and cell survival under these conditions (Supplementary Fig. 9).

Several proteomic investigations have provided insights into the functional protein content implicated in the survival, generation, and/or maintenance of pluripotency [64–66]. With the current study, we understand how hiPSC cell signaling networks differ from the other cellular groups. Ingenuity pathway analysis unveiled that the top-scoring IPA protein network in hiPSCs/IMR90-1 compared to SH-SY5Y is different under 400 mM of NaCl hyperosmolarity stressor. hiPSCs/IMR90-1 are interconnecting tubulin proteins, chaperonin proteins, and other cytoskeleton proteins such as CAP1, whereas SH-SY5Y cells are interconnecting subunits of ribosomal proteins, heterogeneous nuclear ribonucleoprotein, serine- and arginine-rich splicing proteins, and RNA-binding protein such as FUS. This indicates that both types of cells respond to hyperosmolarity stress in various ways to activate survival pathways. The mechanism by which a cell survives and its ability to handle the stress to which it is exposed is unique. On the other hand, intricate signaling networks of protein kinases (PKs) are well known to be involved in regulating cellular events related to cell survival [67]. PK networks represent a significant mechanism by which cellular pluripotency and survival of hiPSCs are regulated [68–70]. To gain a more complete knowledge of cellular processes, we believe that additional phosphoproteome studies are needed to increase our comprehension of the different mechanisms and their fine-tuning involved in hiPSC response to hyperosmolarity stress.

## Data Availability

All data are contained within the manuscript except mass spectrometer raw output files and MaxQuant search results which are deposited at MassIVE repository (doi:10.25345/C51487).

## Supplementary Materials

Supplementary 1 Supplementary Tables—Table S1: LC-MS/MS parameters. Table 2: list of identified proteins—mass spectrometry-based protein quantitation. Table S3: data summary of all quantified proteins in hiPSCs/IMR90-1 and SH-SY5Y. Supplementary Table S4: data summary of overlapped proteins between hiPSCs/IMR90-1 and SH-SY5Y. Supplementary Table S5: top networks in hiPSCs/IMR90-01 and SH-SY5Y.

Supplementary 2 Supplementary Figures—Supplementary Figure 1: pluripotency marker assessment of hiPSCs/IMR90-1 after hyperosmolarity treatment. Supplementary Figure 2: stress granules in iPSCs have the same molecular components as those in other types of cells. Supplementary Figure 3: G3BP stress granule marker in hiPSCs/IMR90-1 after sorbitol treatment. Supplementary Figure 4: two different stem cell lines were treated with NaCl hyperosmolarity treatment (no treatment (control), 200 mM, and 400 mM). Supplementary Figure 5: two different cell lines were treated with NaCl hyperosmolarity treatment (no treatment (control), 200 mM, and 400 mM). Supplementary Figure 6: phosphorylation of eIF2 alpha in both hiPSCs/IMR90-1 and SH-SY5Y after NaCl treatment. Supplementary Figure 7: effects of NaCl on caspase-3 activation by Western blot analysis. Supplementary Figure 8: graphical representation of proteomic data. Supplementary Figure 9: additional mitochondrial quantified proteins.
