# Supplementary material for: Hyperosmotic Stress Induces a Specific Pattern for Stress Granule Formation in Human-Induced Pluripotent Stem Cells
Source: Stem Cells Int. 2021 Oct 15;2021:8274936. doi: 10.1155/2021/8274936 (PMC8538399; doi:10.1155/2021/8274936)
Supplement: Supplementary 1 — Supplementary Tables—Table S1: LC-MS/MS parameters. Table 2: list of identified proteins—mass spectrometry-based protein quantitation. Table S3: data summary of all quantified proteins in hiPSCs/IMR90-1 and SH-SY5Y. Supplementary Table S4: data summary of overlapped proteins between hiPSCs/IMR90-1 and SH-SY5Y. Supplementary Table S5: top networks in hiPSCs/IMR90-01 and SH-SY5Y. [file 8274936.f1.zip › Supplementary Table 2_v2 (1).pdf]

Supplementary Table 2: List of identified proteins - Mass Spectrometry–Based Protein Quantitation:

	UniProt AC/ID	Gene name	Protein name	Majority protein IDs: Fasta headers	Peptides	Sequence coverage [%]	IMR90-1[200mM vs NT] log2 ratio	IMR90-1[200mM vs NT] p-value	IMR90-1[200mM vs NT] FDR	IMR90-1[400mM vs NT] log2 ratio	IMR90-1[400mM vs NT] p-value	IMR90-1[400mM vs NT] FDR	Peptides	Sequence coverage [%]	SHSy5y[200mM vs NT] log2 ratio	SHSy5y[200mM vs NT] p-value	SHSy5y[200mM vs NT] FDR	SHSy5y[400mM vs NT] log2 ratio	SHSy5y[400mM vs NT] p-value	SHSy5y[400mM vs NT] FDR	FCM cluster
1	P0DPI2	GATD3A	Glutamine amid	sp|P0DPI2|GAL3A_HUMAN	3	17.5	-0.01067	0.97378	0.999	0.5892	0.97378	0.99899	4	25	NA	NA	NA	NA	NA	NA	NA
2	P68036	UBE2L3	Ubiquitin-conjug	sp|P68036|UB2L3_HUMAN	2	10.4	-0.072379	0.85246	0.9991	-0.7607	0.06409	0.20018	8	65.6	-0.0669	0.86359	0.9973	-0.221	0.5718	0.6636	NA
3	A0AVT1	UBA6	Ubiquitin-like m	sp|A0AVT1|UBA6_HUMAN	5	5.4	0.5623468	NA	NA	NA	NA	NA	10	11	NA	NA	NA	NA	NA	NA	NA
4	A5YKK6	CNOT1	CCR4-NOT transc	sp|A5YKK6|CNOT1_HUMAN	6	2.9	-0.128986	0.59636	0.9984	0.1993	0.59636	0.99836	8	4	0.31491	NA	NA	NA	NA	NA	NA
5	A6NDG6	PGP	Glycerol-3-phosp	sp|A6NDG6|PGP_HUMAN	2	9	0.0593401	0.88556	0.9991	-0.4489	0.3839	0.593	6	27.4	0.21431	0.62618	0.9973	0.88831	0.053	0.0916	NA
6	A8MPP1	DDX11L8	Putative ATP-de	sp|A8MPP1|D11L8_HUMA	2	2.2	-0.103113	NA	NA	NA	NA	NA	1	1.3	NA	NA	NA	NA	NA	NA	NA
7	Q99613	EIF3C	Eukaryotic trans	sp|Q99613|EIF3C_HUMAN	10	15.4	0.0305203	0.87519	0.9991	-0.2883	0.19671	0.37977	17	25.1	0.02772	0.88657	0.9973	-0.2989	0.1381	0.2057	NA
8	E9PAV3	NACA	Nascent polypep	sp|E9PAV3|NACAM_HUM	6	5.1	0.2180141	0.31103	0.9991	-0.3311	0.13152	0.29758	5	3.4	0.06699	0.7521	0.9973	-0.9268	0.0004	0.0015	4
9	O00116	AGPS	Alkyldihydroxya	sp|O00116|ADAS_HUMAN	3	7	0.0689567	0.75027	0.9984	0.5677	0.75027	0.99836	4	7.8	NA	NA	NA	NA	NA	NA	NA
10	O00139	KIF2A	Kinesin-like prot	sp|O00139|KIF2A_HUMAN	1	1.4	NA	NA	NA	NA	NA	NA	6	7.4	NA	NA	NA	0.40057	NA	NA	NA
11	O00148	DDX39A	ATP-dependent	sp|O00148|DX39A_HUMA	7	17.3	NA	NA	NA	NA	NA	NA	10	26.5	-0.9957	0.07611	0.9991	-1.5615	0.0761	0.9991	NA
12	O00151	PDLIM1	PDZ and LIM dor	sp|O00151|PDLI1_HUMAN	11	53.5	0.0308367	0.9091	0.9987	-0.8939	0.9091	0.99874	NA	NA	NA	NA	NA	NA	NA	NA	NA
13	O00154	ACOT7	Cytosolic acyl co	sp|O00154|BACH_HUMAN	7	24.5	-0.021402	0.91913	0.9991	-0.8517	0.00218	0.02915	8	25.5	0.18385	0.38892	0.9973	-0.6151	0.0094	0.0209	3
14	O00159	MYO1C	Unconventional	sp|O00159|MYO1C_HUMA	1	1.1	NA	NA	NA	NA	NA	NA	4	5.6	0.04983	NA	NA	NA	NA	NA	NA
15	O00161	SNAP23	Synaptosomal-a	sp|O00161|SNP23_HUMAN	4	26.5	-0.184451	0.39312	0.9984	0.2686	0.39312	0.99836	4	25.6	NA	NA	NA	NA	NA	NA	NA
16	O00178	GTPBP1	GTP-binding prot	sp|O00178|GTPB1_HUMAN	2	4.2	0.6726262	NA	NA	NA	NA	NA	3	7.3	-0.0401	NA	NA	NA	NA	NA	NA
17	O00193	SMAP	Small acidic prot	sp|O00193|SMAP_HUMAN	4	26.8	0.2231981	0.57467	0.9991	-0.7326	0.07808	0.22519	5	30.1	-0.0456	0.90819	0.9973	0.27794	0.4858	0.577	NA
18	O00203	AP3B1	AP-3 complex su	sp|O00203|AP3B1_HUMAN	6	5.4	0.039985	0.88096	0.9984	-0.1642	0.88096	0.99836	5	4.8	0.1565	NA	NA	NA	NA	NA	NA
19	O00231	PSMD11	26S proteasome	sp|O00231|PSD11_HUMAN	13	34.6	0.0403358	0.8546	0.9991	-0.3547	0.12067	0.2853	18	47.4	-0.0953	0.66581	0.9973	-0.8156	0.0016	0.0048	4
20	O00232	PSMD12	26S proteasome	sp|O00232|PSD12_HUMAN	13	31.8	-0.09887	0.61093	0.9991	-0.1674	0.39265	0.60102	11	30.5	-0.1836	0.34954	0.9973	-0.8136	0.0006	0.002	4
21	O00264	PGRMC1	Membrane-asso	sp|O00264|PGRC1_HUMAN	6	39.5	-0.093844	0.66702	0.9984	0.2744	0.66702	0.99836	3	15.9	NA	NA	NA	NA	NA	NA	NA
22	O00267	SUPT5H	Transcription el	sp|O00267|SPT5H_HUMAN	6	8.5	-0.431586	NA	NA	NA	NA	NA	10	14.5	-0.0749	NA	NA	NA	NA	NA	NA
23	O00291	HIP1	Huntingtin-inter	sp|O00291|HIP1_HUMAN	1	1	NA	NA	NA	NA	NA	NA	2	1.9	-0.4216	NA	NA	NA	NA	NA	NA
24	O00299	CLIC1	Chloride intrace	sp|O00299|CLIC1_HUMAN	7	41.1	-0.115637	0.64071	0.9991	-0.5719	0.03152	0.12742	11	61	-0.0699	0.77732	0.9973	-0.978	0.0009	0.0031	4
25	O00303	EIF3F	Eukaryotic trans	sp|O00303|EIF3F_HUMAN	10	39.2	-0.593628	0.11019	0.9991	-0.6821	0.06974	0.21129	12	46.5	0.36525	0.31383	0.9973	0.24787	0.4906	0.5797	NA
26	O00410	IPO5	Importin-5	sp|O00410|IPO5_HUMAN	20	24.8	-0.146389	0.52978	0.9991	-0.3941	0.10275	0.26882	29	35.1	-0.1088	0.63952	0.9973	0.60338	0.0174	0.0357	2
27	O00422	SAP18	Histone deacety	sp|O00422|SAP18_HUMAN	4	26.1	0.448963	0.26393	0.9984	0.8126	0.26393	0.99836	5	41.2	0.36161	NA	NA	NA	NA	NA	NA
28	O00425	IGF2BP3	Insulin-like grow	sp|O00425|IF2B3_HUMAN	17	35.8	0.2157527	0.28884	0.9984	0.55	0.28884	0.99836	12	30.1	NA	NA	NA	NA	NA	NA	NA
29	O00429	DNM1L	Dynamin-1-like	sp|O00429|DNM1L_HUMA	3	4.6	NA	NA	NA	NA	NA	NA	12	23.8	0.00071	0.99743	0.9999	0.02414	0.9974	0.9999	NA
30	O00483	NDUFA4	Cytochrome c ox	sp|O00483|NDUA4_HUMA	2	22.2	0.0063321	0.98127	0.999	0.4623	0.98127	0.99899	2	22.2	0.02853	NA	NA	NA	NA	NA	NA
31	O00487	PSMD14	26S proteasome	sp|O00487|PSDE_HUMAN	5	24.8	0.2898981	0.33089	0.9991	0.6663	0.0347	0.13592	8	43.2	0.0384	0.89602	0.9973	-1.3511	0.0002	0.001	5
32	O00505	KPNA3	Importin subunit	sp|O00505|IMA4_HUMAN	6	14.8	0.3612311	0.28521	0.9991	-0.1797	0.62918	0.77581	8	22.6	0.0473	0.88659	0.9973	-0.5211	0.1305	0.1962	NA
33	O00566	MPHOSPH10	U3 small nucleol	sp|O00566|MPP10_HUMA	4	10.3	-0.039822	0.86965	0.9984	0.4688	0.86965	0.99836	5	13.1	NA	NA	NA	NA	NA	NA	NA
34	O00567	NOP56	Nucleolar protei	sp|O00567|NOP56_HUMA	9	21.2	0.0765002	0.79288	0.9991	0.8977	0.0063	0.04962	11	20.4	0.15822	0.5884	0.9973	-0.9917	0.0031	0.0084	5
35	O00571	DDX3X	ATP-dependent	sp|O00571|DDX3X_HUMA	10	19.5	-0.057922	0.78227	0.9991	-0.1701	0.42133	0.61339	18	30.7	-0.0516	0.80532	0.9973	-1.3786	######	######	4
36	O00592	PODXL	Podocalyxin	sp|O00592|PODXL_HUMAN	7	18.6	-0.02306	0.90834	0.9987	0.4651	0.90834	0.99874	NA	NA	NA	NA	NA	NA	NA	NA	NA
37	O00629	KPNA4	Importin subunit	sp|O00629|IMA3_HUMAN	4	10.2	0.1122849	0.76103	0.9991	-0.1051	0.84034	0.91735	4	11.5	-0.3327	0.42507	0.9973	-0.1973	0.6335	0.7163	NA
38	O00767	SCD	Acyl-CoA desatu	sp|O00767|ACOD_HUMAN	5	16.4	-0.03674	0.90083	0.9984	0.8439	0.90083	0.99836	1	3.6	NA	NA	NA	NA	NA	NA	NA
39	O14561	NDUFAB1	Acyl carrier prot	sp|O14561|ACPM_HUMAN	2	15.4	-0.315037	0.28504	0.9991	0.2361	0.45973	0.6439	3	15.4	0.04478	0.86239	0.9973	-0.4341	0.1086	0.1689	NA
40	P47813	EIF1AX	Eukaryotic trans	sp|P47813|IF1AX_HUMAN	3	19.4	0.7805285	0.16742	0.9991	0.1354	0.82061	0.91051	4	31.2	0.22196	0.64999	0.9973	0.15072	0.7823	0.8217	NA
41	O14646	CHD1	Chromodomain-h	sp|O14646|CHD1_HUMAN	3	3.1	0.2243422	0.52307	0.9984	0.1558	0.52307	0.99836	NA	NA	NA	NA	NA	NA	NA	NA	NA
42	O14737	PDCD5	Programmed cel	sp|O14737|PDCD5_HUMA	4	36	0.4146889	0.1885	0.9991	-0.0642	0.83428	0.91706	7	53.6	-0.0396	0.8973	0.9973	0.9864	0.0048	0.012	2
43	O14745	SLC9A3R1	Na(+)/H(+) excha	sp|O14745|NHRF1_HUMA	10	34.9	-0.067809	0.72679	0.9984	-0.1385	0.72679	0.99836	6	20.7	NA	NA	NA	NA	NA	NA	NA
44	O14776	TCERG1	Transcription el	sp|O14776|TCRG1_HUMA	5	6	0.5946413	0.01113	0.9991	0.7794	0.00424	0.03852	6	6.6	0.93458	0.00851	0.7846	0.33323	0.2596	0.3433	1

45	O14818	PSMA7	Proteasome sub	sp|O14818|PSA7_HUMAN	7	37.5	0.2341068	0.26339	0.9991	-0.233	0.26563	0.46617	9	41.5	-0.075	0.71544	0.9973	0.70054	0.0031	0.0084	2
46	O14929	HAT1	Histone acetyltr	sp|O14929|HAT1_HUMAN	4	14.3	0.3388175	NA	NA	NA	NA	NA	4	11.2	-0.1997	0.3837	0.9991	-0.711	0.3837	0.9991	NA
47	P19105	MYL12A	Myosin regulato	sp|P19105|ML12A_HUMAN	3	22.8	-0.311614	0.55686	0.9991	-0.3831	0.5103	0.68136	9	59.1	0.25252	0.59413	0.9973	0.59523	0.2193	0.3042	NA
48	O14964	HGS	Hepatocyte grov	sp|O14964|HGS_HUMAN H	2	2.6	-0.366268	NA	NA	NA	NA	NA	4	6.4	0.13521	0.67887	0.9991	-1.0201	0.6789	0.9991	NA
49	O14974	PPP1R12A	Protein phospha	sp|O14974|MYPT1_HUMA	5	6.1	0.1777187	NA	NA	NA	NA	NA	11	13.6	0.00567	0.98645	0.9991	-0.8464	0.9864	0.9991	NA
50	O14979	HNRNPDL	Heterogeneous	sp|O14979|HNRDL_HUMAN	7	16.9	0.4098684	0.21037	0.9991	0.3604	0.26802	0.46919	9	20.7	-0.5146	0.1623	0.9973	2.1266	######	0.0001	1
51	O14980	XPO1	Exportin-1	sp|O14980|XPO1_HUMAN	15	16.9	0.2251771	0.32763	0.9991	-0.7347	0.00448	0.04022	22	26.2	0.16353	0.47403	0.9973	0.25463	0.2703	0.3554	2
52	O15042	U2SURP	U2 snRNP-associ	sp|O15042|SR140_HUMAN	4	5.4	-0.127157	NA	NA	NA	NA	NA	6	7.8	0.53825	NA	NA	NA	NA	NA	NA
53	O15067	PFAS	Phosphoribosylfo	sp|O15067|PUR4_HUMAN	6	7.5	-0.202859	0.5233	0.9984	-0.3697	0.5233	0.99836	9	10.4	NA	NA	NA	NA	NA	NA	NA
54	O15143	ARPC1B	Actin-related pr	sp|O15143|ARC1B_HUMAN	2	8.3	NA	NA	NA	NA	NA	NA	6	23.4	-0.4862	0.08543	0.9991	-0.2621	0.0854	0.9991	NA
55	O15144	ARPC2	Actin-related pr	sp|O15144|ARPC2_HUMAN	7	24.7	0.1211492	0.48482	0.9991	-0.3264	0.07168	0.21444	10	43	0.12153	0.48347	0.9973	-0.9171	######	0.0003	4
56	O15145	ARPC3	Actin-related pr	sp|O15145|ARPC3_HUMAN	1	7.3	NA	NA	NA	NA	NA	NA	4	35.4	0.75477	NA	NA	NA	NA	NA	NA
57	O15155	BET1	BET1 homolog	sp|O15155|BET1_HUMAN	2	24.6	NA	NA	NA	NA	NA	NA	2	24.6	0.21572	0.5816	0.9991	0.39843	0.5816	0.9991	NA
58	O15212	PFDN6	Prefoldin subunit	sp|O15212|PFD6_HUMAN	3	26.4	0.2872452	0.54796	0.9991	-1.2398	0.0683	0.20962	5	43.4	0.03375	0.93666	0.9973	2.92207	######	######	2
59	O15347	HMGB3	High mobility gr	sp|O15347|HMGB3_HUMA	5	24	-0.18542	0.54707	0.9984	-0.9113	0.54707	0.99836	4	20	NA	NA	NA	NA	NA	NA	NA
60	O15355	PPM1G	Protein phospha	sp|O15355|PPM1G_HUMA	10	28.9	-0.104997	0.70061	0.9991	-0.4056	0.1497	0.32163	18	52	-0.0597	0.82657	0.9973	1.03004	0.0014	0.0043	2
61	O15371	EIF3D	Eukaryotic trans	sp|O15371|EIF3D_HUMAN	9	18.6	-0.129779	0.67035	0.9991	-1.01	0.00377	0.03699	13	27.7	-0.1291	0.67197	0.9973	-0.8741	0.0099	0.0218	3
62	O15372	EIF3H	Eukaryotic trans	sp|O15372|EIF3H_HUMAN	8	30.1	0.2121387	0.47509	0.9991	-0.2283	0.44273	0.62905	10	35.8	-0.0483	0.86971	0.9973	-0.8082	0.013	0.0277	4
63	O15498	YKT6	Synaptobrevin h	sp|O15498|YKT6_HUMAN S	4	22.2	0.0240236	0.95448	0.9991	-0.7739	0.15611	0.33039	8	47.5	0.29603	0.56919	0.9973	2.42322	######	0.0005	2
64	O15511	ARPC5	Actin-related pr	sp|O15511|ARPC5_HUMAN	3	24.5	NA	NA	NA	NA	NA	NA	5	49	0.39458	0.14647	0.9991	-0.5533	0.1465	0.9991	NA
65	O43143	DHX15	Pre-mRNA-splicin	sp|O43143|DHX15_HUMAN	13	19.9	0.1861896	0.40294	0.9991	0.3081	0.22193	0.41407	22	32.6	-0.1468	0.50786	0.9973	0.48028	0.042	0.0744	1
66	O43169	CYB5B	Cytochrome b5 t	sp|O43169|CYB5B_HUMAN	5	35.6	-0.093222	0.74483	0.9984	0.4986	0.74483	0.99836	4	50.7	NA	NA	NA	NA	NA	NA	NA
67	O43172	PRPF4	U4/U6 small nuc	sp|O43172|PRP4_HUMAN	3	6.5	0.1268163	0.57017	0.9984	0.4057	0.57017	0.99836	6	15.5	NA	NA	NA	NA	NA	NA	NA
68	O43175	PHGDH	D-3-phosphoglyc	sp|O43175|SERA_HUMAN	16	37	0.2179407	0.29464	0.9991	-0.6886	0.00341	0.03677	19	45	0.08821	0.66696	0.9973	1.63881	######	######	2
69	O43242	PSMD3	26S proteasome	sp|O43242|PSMD3_HUMA	16	33.7	0.1204179	0.52199	0.9991	0.1108	0.55546	0.71474	16	34.5	-0.0111	0.95243	0.9973	-0.6519	0.0026	0.0073	4
70	O43252	PAPSS1	Bifunctional 3-ph	sp|O43252|PAPS1_HUMAN	2	3.5	NA	NA	NA	NA	NA	NA	11	24.2	-0.2012	0.41524	0.9991	-0.5771	0.4152	0.9991	NA
71	O43264	ZW10	Centromere/kin	sp|O43264|ZW10_HUMAN	2	4.1	NA	NA	NA	0.3369	NA	NA	NA	NA	NA	NA	NA	NA	NA	NA	NA
72	O43324	EEF1E1	Eukaryotic trans	sp|O43324|MCA3_HUMAN	5	26.4	0.3846133	0.13472	0.9984	0.2689	0.13472	0.99836	6	33.9	NA	NA	NA	NA	NA	NA	NA
73	O43390	HNRNPR	Heterogeneous	sp|O43390|HNRPR_HUMAN	23	38.9	0.0690927	0.70729	0.9991	0.5847	0.01089	0.0683	25	42.5	0.04621	0.80144	0.9973	0.98834	######	0.0003	1
74	O43396	TXNL1	Thioredoxin-like	sp|O43396|TXNL1_HUMAN	7	30.1	0.2879908	0.19713	0.9991	-0.0501	0.81783	0.91051	8	36.3	0.18714	0.39494	0.9973	-1.4202	######	######	4
75	O43399	TPD52L2	Tumor protein D	sp|O43399|TPD54_HUMAN	4	29.1	0.1108939	0.64367	0.9984	0.0649	0.64367	0.99836	9	51.5	NA	NA	NA	NA	NA	NA	NA
76	O43491	EPB41L2	Band 4.1-like pro	sp|O43491|E41L2_HUMAN	26	28.8	0.1261575	0.55735	0.9984	1.0912	0.55735	0.99836	2	2.8	NA	NA	NA	NA	NA	NA	NA
77	O43583	DENR	Density-regulate	sp|O43583|DENR_HUMAN	4	23.7	NA	NA	NA	0.5414	NA	NA	7	49	NA	NA	NA	NA	NA	NA	NA
78	O43615	TIMM44	Mitochondrial in	sp|O43615|TIM44_HUMAN	7	18.8	0.2799074	0.15086	0.9984	0.5918	0.15086	0.99836	7	19.9	NA	NA	NA	NA	NA	NA	NA
79	O43660	PLRG1	Pleiotropic regu	sp|O43660|PLRG1_HUMAN	3	6.4	0.7442529	0.18506	0.9984	1.4454	0.18506	0.99836	3	7.8	0.04405	NA	NA	NA	NA	NA	NA
80	O43670	ZNF207	BUB3-interacting	sp|O43670|ZN207_HUMAN	2	5.4	NA	NA	NA	NA	NA	NA	3	7.9	NA	NA	NA	1.69664	NA	NA	NA
81	O43684	BUB3	Mitotic checkpo	sp|O43684|BUB3_HUMAN	5	20.7	0.271462	0.18924	0.9991	0.3294	0.11555	0.28056	6	29	0.12503	0.53681	0.9973	-0.6676	0.0038	0.0099	5
82	O43707	ACTN4	Alpha-actinin-4	sp|O43707|ACTN4_HUMAN	28	39.2	-0.046027	0.88056	0.9991	-0.8923	0.00907	0.06244	45	57.2	-0.0161	0.95813	0.9973	-0.7712	0.0208	0.0415	3
83	O43719	HTATSF1	HIV Tat-specific	sp|O43719|HTSF1_HUMAN	4	7.2	0.4456123	0.21915	0.9984	0.0756	0.21915	0.99836	6	10.7	NA	NA	NA	NA	NA	NA	NA
84	O43765	SGTA	Small glutamine	sp|O43765|SGTA_HUMAN	3	12.8	0.0567004	0.77807	0.9984	0.0827	0.77807	0.99836	7	27.2	NA	NA	NA	1.24055	NA	NA	NA
85	O43768	ENSA	Alpha-endosulfin	sp|O43768|ENSA_HUMAN	3	30.6	0.3188028	0.3595	0.9991	-0.0265	0.93855	0.96022	7	60.3	0.11161	0.74553	0.9973	0.79123	0.0323	0.0597	1
86	O43776	NARS	Asparagine--tRN	sp|O43776|SYNC_HUMAN	11	24.3	-0.188964	0.56107	0.9984	-0.8073	0.56107	0.99836	10	23.2	-0.0499	NA	NA	NA	NA	NA	NA
87	O43795	MYO1B	Unconventional	sp|O43795|MYO1B_HUMA	3	3.3	NA	NA	NA	NA	NA	NA	8	9.8	-0.034	0.96617	0.9991	1.36166	0.9662	0.9991	NA
88	O43852	CALU	Calumenin	sp|O43852|CALU_HUMAN	12	50.2	0.2649089	0.26946	0.9991	0.5196	0.03916	0.1469	17	64.4	0.11095	0.63845	0.9973	-0.2957	0.2199	0.3045	5
89	O43865	AHCYL1	S-adenosylhomo	sp|O43865|SAHH2_HUMA	2	4.3	-0.293612	NA	NA	NA	NA	NA	5	13.6	NA	NA	NA	NA	NA	NA	NA
90	O60216	RAD21	Double-strand-b	sp|O60216|RAD21_HUMAN	5	12	-0.059642	0.85026	0.9984	0.1254	0.85026	0.99836	2	5.7	NA	NA	NA	NA	NA	NA	NA
91	O60264	SMARCA5	SWI/SNF-related	sp|O60264|SMCA5_HUMA	19	22.2	0.1649243	0.40299	0.9991	0.5611	0.00983	0.06319	15	17.5	-0.0527	0.78736	0.9973	-0.2105	0.289	0.3746	5
92	O60271	SPAG9	C-Jun-amino-terr	sp|O60271|JIP4_HUMAN C	3	3.5	NA	NA	NA	NA	NA	NA	9	10.1	-0.4317	NA	NA	NA	NA	NA	NA

93	O60313	OPA1	Dynamin-like 12	sp|O60313|OPA1_HUMAN	2	2.7	0.0911449	0.81198	0.9991	-0.1398	0.75241	0.86792	3	3.9	0.09461	0.79349	0.9973	-2.0625	0.0005	0.0018	4
94	O60341	KDM1A	Lysine-specific hi	sp|O60341|KDM1A_HUMA	6	14.3	0.4521063	0.08611	0.9984	0.6803	0.08611	0.99836	9	13.3	NA	NA	NA	NA	NA	NA	NA
95	O60488	ACSL4	Long-chain-fatty-	sp|O60488|ACSL4_HUMAN	8	14.5	0.0131394	0.95708	0.999	0.3679	0.95708	0.99899	6	11.5	-0.1005	NA	NA	NA	NA	NA	NA
96	O60502	OGA	Protein O-GlcNA	sp|O60502|OGA_HUMAN F	4	5.2	0.0416963	0.81781	0.9984	-0.0065	0.81781	0.99836	2	2.7	NA	NA	NA	NA	NA	NA	NA
97	O60506	SYNCRIP	Heterogeneous	sp|O60506|HNRPQ_HUMA	25	45.7	-0.043179	0.8444	0.9991	-0.2155	0.33416	0.53481	26	52.2	0.12732	0.56457	0.9973	0.28255	0.21	0.2948	NA
98	O60566	BUB1B	Mitotic checkpoi	sp|O60566|BUB1B_HUMAN	3	3.3	-0.087041	0.83222	0.9984	0.8282	0.83222	0.99836	NA	NA	NA	NA	NA	NA	NA	NA	NA
99	O60610	DIAPH1	Protein diaphan	sp|O60610|DIAP1_HUMAN	5	4.8	-0.058727	0.81862	0.9984	0.006	0.81862	0.99836	5	4.9	NA	NA	NA	NA	NA	NA	NA
100	O60664	PLIN3	Perilipin-3	sp|O60664|PLIN3_HUMAN	3	8.3	-0.522162	0.08683	0.9984	-0.868	0.08683	0.99836	8	26.5	NA	NA	NA	2.03141	NA	NA	NA
101	O60684	KPNA6	Importin subunit	sp|O60684|IMA7_HUMAN	2	5.4	NA	NA	NA	NA	NA	NA	9	28.4	0.09468	0.62715	0.9991	0.08749	0.6272	0.9991	NA
102	O60701	UGDH	UDP-glucose 6-d	sp|O60701|UGDH_HUMAN	8	20.9	0.1246908	0.76112	0.9991	-0.1322	0.81961	0.91051	11	27.3	-0.0755	0.85376	0.9973	-1.8086	0.0005	0.0017	4
103	O60716	CTNND1	Catenin delta-1	sp|O60716|CTND1_HUMAN	6	7.4	NA	NA	NA	NA	NA	NA	9	12.6	-0.3299	NA	NA	NA	NA	NA	NA
104	O60749	SNX2	Sorting nexin-2	sp|O60749|SNX2_HUMAN	7	16.4	0.289386	0.43986	0.9991	-0.5146	0.17789	0.35476	11	26.4	0.24868	0.5058	0.9973	-0.0812	0.8269	0.8571	NA
105	O60763	USO1	General vesicula	sp|O60763|USO1_HUMAN	11	17.3	-0.046489	0.76957	0.9984	-0.1701	0.76957	0.99836	6	8	NA	NA	NA	NA	NA	NA	NA
106	O60841	EIF5B	Eukaryotic trans	sp|O60841|IF2P_HUMAN E	10	10.7	0.0338857	0.90056	0.9984	-0.2159	0.90056	0.99836	18	21.3	-0.0947	NA	NA	NA	NA	NA	NA
107	O60869	EDF1	Endothelial diffe	sp|O60869|EDF1_HUMAN	5	35.8	-0.213205	0.71486	0.9984	-0.748	0.71486	0.99836	4	24.3	NA	NA	NA	NA	NA	NA	NA
108	O60884	DNAJA2	DnaJ homolog su	sp|O60884|DNJA2_HUMAN	8	25.7	0.2551244	0.3369	0.9991	0.0808	0.78269	0.88789	10	29.6	-0.0483	0.8536	0.9973	0.48753	0.0772	0.129	NA
109	O60885	BRD4	Bromodomain-co	sp|O60885|BRD4_HUMAN	6	4.8	-1.266953	NA	NA	NA	NA	NA	5	4.6	0.13977	0.66001	0.9991	-0.0758	0.66	0.9991	NA
110	O60925	PFDN1	Prefoldin subunit	sp|O60925|PFD1_HUMAN	4	31.1	-0.0897	0.79898	0.9991	-0.2362	0.63637	0.7779	6	56.6	-0.5752	0.11786	0.9973	1.13965	0.0051	0.0128	2
111	O75083	WDR1	WD repeat-conta	sp|O75083|WDR1_HUMAN	18	42.7	0.0470403	0.86157	0.9991	-1.2908	0.00016	0.00845	11	27.1	0.12427	0.64596	0.9973	-1.159	0.0005	0.0017	3
112	O75190	DNAJB6	DnaJ homolog su	sp|O75190|DNJB6_HUMAN	5	20.2	0.194569	0.37804	0.9984	0.1431	0.37804	0.99836	3	10.4	NA	NA	NA	NA	NA	NA	NA
113	O75223	GGCT	Gamma-glutamy	sp|O75223|GGCT_HUMAN	4	23.4	0.343426	0.3981	0.9984	-0.3863	0.3981	0.99836	6	37.2	NA	NA	NA	NA	NA	NA	NA
114	O75347	TBCA	Tubulin-specific c	sp|O75347|TBCA_HUMAN	8	56.5	-0.13052	0.68505	0.9991	-0.6782	0.04805	0.17027	11	58.3	-0.5728	0.12503	0.9973	2.65722	######	######	2
115	O75348	ATP6V1G1	V-type proton A	sp|O75348|VATG1_HUMA	2	22	NA	NA	NA	0.0998	NA	NA	3	28.8	NA	NA	NA	NA	NA	NA	NA
116	O75367	H2AFY	Core histone ma	sp|O75367|H2AY_HUMAN	4	19.1	0.2585687	0.5553	0.9991	0.6128	0.22015	0.41184	11	39.8	0.42171	0.3406	0.9973	2.68739	######	######	1
117	O75368	SH3BGRL	SH3 domain-bind	sp|O75368|SH3L1_HUMAN	1	8.8	NA	NA	NA	NA	NA	NA	4	46.5	-0.09	NA	NA	NA	NA	NA	NA
118	O75369	FLNB	Filamin-B	sp|O75369|FLNB_HUMAN	64	34.7	0.0718831	0.70238	0.9991	-0.29	0.13584	0.30186	43	23.3	0.25087	0.19316	0.9973	-2.4641	######	######	4
119	O75390	CS	Citrate synthase	sp|O75390|CISY_HUMAN C	6	13.3	-0.175336	0.36321	0.9991	0.4001	0.04827	0.17027	9	20.8	-0.1936	0.31667	0.9973	-0.3436	0.085	0.1409	5
120	O75396	SEC22B	Vesicle-traffickin	sp|O75396|SC22B_HUMAN	1	6.5	NA	NA	NA	NA	NA	NA	7	34	0.26894	0.33692	0.9991	0.72145	0.3369	0.9991	NA
121	O75400	PRPF40A	Pre-mRNA-proce	sp|O75400|PR40A_HUMAN	5	5.3	0.163888	0.79031	0.9984	1.268	0.79031	0.99836	5	4.7	NA	NA	NA	NA	NA	NA	NA
122	O75436	VPS26A	Vacuolar protein	sp|O75436|VP26A_HUMAN	4	15.6	NA	NA	NA	NA	NA	NA	8	33	0.32988	0.2744	0.9991	0.17202	0.2744	0.9991	NA
123	O75475	PSIP1	PC4 and SFRS1-i	sp|O75475|PSIP1_HUMAN	19	40	-0.015337	0.95286	0.9991	0.508	0.06378	0.20018	9	19.6	-0.2563	0.33038	0.9973	0.07635	0.7688	0.8136	NA
124	O75477	ERLIN1	Erlin-1	sp|O75477|ERLN1_HUMAN	6	19.1	0.1976386	0.48993	0.9991	1.2773	0.00416	0.03837	6	20.2	-1.3832	0.00366	0.6565	-0.4307	0.1488	0.2198	5
125	O75487	GPC4	Glypican-4	sp|O75487|GPC4_HUMAN	7	15.1	-0.258991	0.35435	0.9984	0.4062	0.35435	0.99836	NA	NA	NA	NA	NA	NA	NA	NA	NA
126	O75533	SF3B1	Splicing factor 3	sp|O75533|SF3B1_HUMAN	14	14.5	0.0781279	0.66895	0.9991	0.6149	0.00337	0.03677	27	28.5	0.03993	0.82666	0.9973	-0.1178	0.5207	0.6112	5
127	O75534	CSDE1	Cold shock doma	sp|O75534|CSDE1_HUMAN	16	24.6	0.0567411	0.84904	0.9991	-0.1043	0.72681	0.85174	18	23.6	-0.1324	0.65781	0.9973	0.94888	0.0051	0.0127	2
128	O75607	NPM3	Nucleoplasmin-3	sp|O75607|NPM3_HUMAN	3	23.6	0.5992544	NA	NA	NA	NA	NA	2	14.6	NA	NA	NA	NA	NA	NA	NA
129	O75643	SNRNP200	U5 small nuclear	sp|O75643|U520_HUMAN	19	9.9	-0.031705	0.87362	0.9991	0.2788	0.17423	0.35193	27	16.4	0.03994	0.84122	0.9973	0.45663	0.0331	0.061	1
130	O75694	NUP155	Nuclear pore cor	sp|O75694|NU155_HUMAN	5	5.3	0.8225538	0.10093	0.9984	0.7064	0.10093	0.99836	8	8.1	0.08355	NA	NA	NA	NA	NA	NA
131	O75717	WDHD1	WD repeat and	sp|O75717|WDHD1_HUMA	3	3.7	0.1839963	0.33458	0.9984	0.1375	0.33458	0.99836	1	1.6	NA	NA	NA	NA	NA	NA	NA
132	O75787	ATP6AP2	Renin receptor	sp|O75787|RENR_HUMAN	2	5.4	NA	NA	NA	NA	NA	NA	2	7.7	0.11922	NA	NA	NA	NA	NA	NA
133	O75821	EIF3G	Eukaryotic trans	sp|O75821|EIF3G_HUMAN	7	28.8	0.0799303	0.71157	0.9991	-0.1799	0.40937	0.60515	11	36.2	0.12429	0.5665	0.9973	-0.9575	0.0003	0.0013	4
134	O75822	EIF3J	Eukaryotic trans	sp|O75822|EIF3J_HUMAN	7	30.6	0.2842782	0.22803	0.9991	0.0668	0.77234	0.88037	10	48.1	0.21551	0.35621	0.9973	-0.1961	0.4002	0.4952	NA
135	O75832	PSMD10	26S proteasome	sp|O75832|PSD10_HUMAN	3	13.7	0.6857441	NA	NA	NA	NA	NA	NA	NA	NA	NA	NA	NA	NA	NA	NA
136	O75844	ZMPSTE24	CAAX prenyl pro	sp|O75844|FACE1_HUMAN	7	13.9	0.0229702	0.94742	0.999	0.4829	0.94742	0.99899	7	18.9	NA	NA	NA	NA	NA	NA	NA
137	O75874	IDH1	Isocitrate dehyd	sp|O75874|IDHC_HUMAN	20	57.7	-0.083871	0.82982	0.9991	-0.7271	0.07702	0.22519	13	40.3	0.39093	0.37614	0.9973	3.56196	######	######	2
138	O75937	DNAJC8	DnaJ homolog su	sp|O75937|DNJC8_HUMAN	4	23.3	0.3626254	0.33304	0.9991	0.5043	0.18402	0.36444	7	27.7	0.10734	0.77151	0.9973	1.10992	0.0074	0.0171	1
139	O75947	ATP5PD	ATP synthase su	sp|O75947|ATP5H_HUMAN	7	55.9	0.2738739	0.31239	0.9984	0.6344	0.31239	0.99836	8	65.2	NA	NA	NA	NA	NA	NA	NA
140	O75955	FLOT1	Flotillin-1	sp|O75955|FLOT1_HUMAN	2	6.3	NA	NA	NA	NA	NA	NA	4	12.2	-0.3598	NA	NA	NA	NA	NA	NA

141	O75964	ATP5MG	ATP synthase su	sp|O75964|ATP5L_HUMAN	3	32	-0.276163	0.44576	0.9984	0.4752	0.44576	0.99836	3	34	NA	NA	NA	NA	NA	NA	NA
142	O76003	GLRX3	Glutaredoxin-3	sp|O76003|GLRX3_HUMAN	11	43	0.0664878	0.81042	0.9984	-0.181	0.81042	0.99836	15	57.3	NA	NA	NA	2.5511	NA	NA	NA
143	O76021	RSL1D1	Ribosomal L1 do	sp|O76021|RL1D1_HUMAN	4	9.6	-0.630967	0.21524	0.9984	0.2058	0.21524	0.99836	8	19.4	NA	NA	NA	NA	NA	NA	NA
144	O76070	SNCG	Gamma-synuclei	sp|O76070|SYUG_HUMAN	3	33.1	0.1668208	NA	NA	NA	NA	NA	NA	NA	NA	NA	NA	NA	NA	NA	NA
145	O76094	SRP72	Signal recognitio	sp|O76094|SRP72_HUMAN	11	18.3	0.3689523	NA	NA	NA	NA	NA	7	15.5	0.10211	NA	NA	NA	NA	NA	NA
146	O94808	GFPT2	Glutamine--fruct	sp|O94808|GFPT2_HUMAN	5	10	-1.152142	0.04504	0.9984	0.7199	0.04504	0.99836	5	9.8	NA	NA	NA	NA	NA	NA	NA
147	O94826	TOMM70	Mitochondrial in	sp|O94826|TOM70_HUMA	4	6.9	-0.162299	0.71507	0.9991	-0.3594	0.42476	0.6146	14	31.2	0.08717	0.73402	0.9973	0.93217	0.0037	0.0096	2
148	O94905	ERLIN2	Erlin-2	sp|O94905|ERLN2_HUMAN	7	24.5	-0.152241	0.47592	0.9984	0.6605	0.47592	0.99836	5	18.6	NA	NA	NA	NA	NA	NA	NA
149	O94906	PRPF6	Pre-mRNA-proce	sp|O94906|PRP6_HUMAN	2	2.8	NA	NA	NA	NA	NA	NA	7	8.6	0.09813	NA	NA	NA	NA	NA	NA
150	O94925	GLS	Glutaminase kid	sp|O94925|GLSK_HUMAN	2	3.7	NA	NA	NA	NA	NA	NA	6	12.3	0.06018	0.7902	0.9991	0.19486	0.7902	0.9991	NA
151	O94992	HEXIM1	Protein HEXIM1	sp|O94992|HEXI1_HUMAN	1	7	NA	NA	NA	NA	NA	NA	3	15.6	0.28728	0.41691	0.9991	-0.2148	0.4169	0.9991	NA
152	O95104	SCAF4	Splicing factor, a	sp|O95104|SFR15_HUMAN	5	5.8	0.1566584	0.70496	0.9984	1.259	0.70496	0.99836	3	4.2	NA	NA	NA	NA	NA	NA	NA
153	O95163	ELP1	Elongator compl	sp|O95163|ELP1_HUMAN	2	1.9	NA	NA	NA	NA	NA	NA	7	6.5	-0.0907	NA	NA	NA	NA	NA	NA
154	O95197	RTN3	Reticulon-3	sp|O95197|RTN3_HUMAN	2	4.6	0.009762	0.95979	0.999	0.8018	0.95979	0.99899	3	5.6	NA	NA	NA	NA	NA	NA	NA
155	O95202	LETM1	Mitochondrial pr	sp|O95202|LETM1_HUMAN	13	21.1	0.018274	0.9601	0.999	-0.0207	0.9601	0.99899	9	12.9	0.30183	NA	NA	NA	NA	NA	NA
156	O95232	LUC7L3	Luc7-like protein	sp|O95232|LC7L3_HUMAN	2	5.8	NA	NA	NA	NA	NA	NA	7	20.8	0.14772	0.46893	0.9991	0.00248	0.4689	0.9991	NA
157	O95239	KIF4A	Chromosome-ass	sp|O95239|KIF4A_HUMAN	4	3.4	-0.374034	0.38694	0.9984	-0.0854	0.38694	0.99836	7	5.7	0.10051	NA	NA	NA	NA	NA	NA
158	O95292	VAPB	Vesicle-associate	sp|O95292|VAPB_HUMAN	5	26.7	NA	NA	NA	NA	NA	NA	9	45.3	0.38166	0.2693	0.9991	2.13486	0.2693	0.9991	NA
159	O95347	SMC2	Structural maint	sp|O95347|SMC2_HUMAN	16	16.1	0.3366711	0.09297	0.9991	0.3984	0.07716	0.22519	11	10.6	0.10863	0.5691	0.9973	-0.709	0.0181	0.0368	5
160	O95373	IPO7	Importin-7	sp|O95373|IPO7_HUMAN	7	8.4	0.1465205	0.58005	0.9991	0.2152	0.46889	0.64919	16	20.5	-0.1219	0.64484	0.9973	-0.9772	0.0018	0.0052	4
161	O95433	AHSA1	Activator of 90 k	sp|O95433|AHSA1_HUMA	7	34.6	-0.193404	0.57379	0.9991	-0.1013	0.815	0.91051	11	44.4	0.09944	0.74543	0.9973	0.75457	0.0254	0.049	2
162	O95456	PSMG1	Proteasome asse	sp|O95456|PSMG1_HUMA	2	8	0.631064	0.05174	0.9984	0.1397	0.05174	0.99836	2	8	NA	NA	NA	NA	NA	NA	NA
163	O95671	ASMTL	Probable bifunct	sp|O95671|ASML_HUMAN	4	7.7	-0.485979	0.13061	0.9984	-0.8344	0.13061	0.99836	NA	NA	NA	NA	NA	NA	NA	NA	NA
164	O95747	OXSR1	Serine/threonin	sp|O95747|OXSR1_HUMAN	2	4.6	NA	NA	NA	NA	NA	NA	6	12.1	0.3618	0.25953	0.9991	-0.3366	0.2595	0.9991	NA
165	O95782	AP2A1	AP-2 complex su	sp|O95782|AP2A1_HUMAN	7	8.3	0.0459722	0.83205	0.9984	0.1341	0.83205	0.99836	11	13.2	0.20067	NA	NA	NA	NA	NA	NA
166	O95819	MAP4K4	Mitogen-activat	sp|O95819|M4K4_HUMAN	3	3.6	NA	NA	NA	NA	NA	NA	6	6.5	-0.8129	NA	NA	NA	NA	NA	NA
167	O95831	AIFM1	Apoptosis-induci	sp|O95831|AIFM1_HUMAN	16	32.6	0.0820011	0.7534	0.9991	0.6715	0.01847	0.09524	9	17.1	-0.1895	0.47086	0.9973	-0.2563	0.3325	0.4225	5
168	O95864	FADS2	Acyl-CoA 6-desat	sp|O95864|FADS2_HUMAN	5	15.1	-0.148753	0.73846	0.9984	0.5931	0.73846	0.99836	4	11.9	NA	NA	NA	NA	NA	NA	NA
169	O95881	TXNDC12	Thioredoxin dom	sp|O95881|TXD12_HUMAN	4	30.8	0.2241703	0.50922	0.9991	0.0614	0.86799	0.92126	4	29.7	0.1036	0.75879	0.9973	0.27394	0.4218	0.5138	NA
170	O96008	TOMM40	Mitochondrial in	sp|O96008|TOM40_HUMA	6	21.6	-0.390405	0.30712	0.9984	0.6056	0.30712	0.99836	2	6.1	NA	NA	NA	NA	NA	NA	NA
171	O96019	ACTL6A	Actin-like protei	sp|O96019|ACL6A_HUMAN	4	12.6	-0.222544	0.40236	0.9991	0.5604	0.071	0.21331	8	29.4	0.26899	0.314	0.9973	0.18264	0.4903	0.5797	NA
172	P00167	CYB5A	Cytochrome b5	sp|P00167|CYB5_HUMAN	5	49.3	-0.296963	0.46851	0.9991	0.6705	0.17091	0.34711	5	42.5	-0.1945	0.52282	0.9973	-0.4003	0.158	0.2325	NA
173	P00338	LDHA	L-lactate dehydr	sp|P00338|LDHA_HUMAN	13	41.3	-0.11258	0.87051	0.9991	-0.9967	0.16162	0.33626	13	37	-0.2487	0.71918	0.9973	-2.2968	0.0037	0.0097	4
174	P00367	GLUD1	Glutamate dehy	sp|P00367|DHE3_HUMAN	11	23.3	-0.026912	0.88027	0.9991	0.4967	0.01203	0.07061	14	31	-0.0394	0.82564	0.9973	-0.6715	0.0015	0.0044	5
175	P00374	DHFR	Dihydrofolate re	sp|P00374|DYR_HUMAN D	4	24.6	NA	NA	NA	NA	NA	NA	5	31	-0.1792	NA	NA	NA	NA	NA	NA
176	P00387	CYB5R3	NADH-cytochron	sp|P00387|NB5R3_HUMAN	3	13.3	-0.252115	0.37053	0.9984	0.7605	0.37053	0.99836	8	28.9	NA	NA	NA	NA	NA	NA	NA
177	P00390	GSR	Glutathione red	sp|P00390|GSHR_HUMAN	3	8.2	0.0301839	0.88452	0.9991	-0.4177	0.08707	0.24019	6	16.1	0.23975	0.25866	0.9973	0.18701	0.3744	0.4698	NA
178	P00403	MT-CO2	Cytochrome c ox	sp|P00403|COX2_HUMAN	4	24.7	-0.702172	0.18675	0.9984	0.5655	0.18675	0.99836	5	27.8	NA	NA	NA	NA	NA	NA	NA
179	P00441	SOD1	Superoxide dism	sp|P00441|SODC_HUMAN	5	40.3	-0.082309	0.8248	0.9984	-0.5955	0.8248	0.99836	8	69.5	NA	NA	NA	NA	NA	NA	NA
180	P00491	PNP	Purine nucleosid	sp|P00491|PNPH_HUMAN	6	27	0.1197411	0.48763	0.9984	-0.2984	0.48763	0.99836	12	58.5	-0.6365	NA	NA	NA	NA	NA	NA
181	P00492	HPRT1	Hypoxanthine-gu	sp|P00492|HPRT_HUMAN	4	22	-0.038893	NA	NA	NA	NA	NA	5	25.7	NA	NA	NA	NA	NA	NA	NA
182	P00505	GOT2	Aspartate amino	sp|P00505|AATM_HUMAN	9	19.5	0.1645103	0.38431	0.9991	0.3419	0.08125	0.23223	12	32.1	-0.0532	0.77628	0.9973	0.02252	0.9041	0.917	NA
183	P00558	PGK1	Phosphoglycerat	sp|P00558|PGK1_HUMAN	22	59	0.0509379	0.79407	0.9991	-0.6753	0.00277	0.03304	28	80.1	-0.0219	0.91042	0.9973	-0.8222	0.0005	0.0019	3
184	P01111	NRAS	GTPase NRas	sp|P01111|RASN_HUMAN	2	12.2	0.2233319	0.54478	0.9984	0.4146	0.54478	0.99836	2	12.2	NA	NA	NA	NA	NA	NA	NA
185	P10316	HLA-A	HLA class I histo	sp|P10316|1A69_HUMAN	4	16.2	-0.17651	0.55716	0.9984	0.4492	0.55716	0.99836	NA	NA	NA	NA	NA	NA	NA	NA	NA
186	P02545	LMNA	Prelamin-A/C	sp|P02545|LMNA_HUMAN	8	12.2	0.2575312	0.34365	0.9991	0.9011	0.00347	0.03677	35	51.8	0.02049	0.9391	0.9973	-0.148	0.5827	0.6729	5
187	P02786	TFRC	Transferrin rece	sp|P02786|TFR1_HUMAN T	9	13.6	-0.038067	0.87562	0.9991	0.8571	0.00713	0.05274	17	28	0.46379	0.21799	0.9973	2.98067	######	######	1
188	P02787	TF	Serotransferrin	sp|P02787|TRFE_HUMAN S	6	10.6	-0.598464	0.04363	0.9991	0.0581	0.85048	0.91851	17	29.1	-0.3102	0.3221	0.9973	2.29499	######	######	1

189	P02794	FTH1	Ferritin heavy c	sp|P02794|FRIH_HUMAN F	5	28.4	-0.041736	0.9095	0.9991	-0.3584	0.33642	0.536	7	46.4	0.01222	0.97624	0.9973	-0.1769	0.6313	0.715	NA
190	P04075	ALDOA	Fructose-bisphos	sp|P04075|ALDOA_HUMAN	16	60.2	0.0092993	0.97065	0.9991	-1.1475	0.00027	0.00995	29	86.3	0.2353	0.35817	0.9973	0.7049	0.0118	0.0255	2
191	P04080	CSTB	Cystatin-B	sp|P04080|CYTB_HUMAN C	4	56.1	-0.026302	0.91656	0.999	-0.8345	0.91656	0.99899	4	56.1	NA	NA	NA	NA	NA	NA	NA
192	P04179	SOD2	Superoxide dism	sp|P04179|SODM_HUMAN	2	9.5	0.0065781	0.98341	0.999	0.1695	0.98341	0.99899	2	9.9	NA	NA	NA	NA	NA	NA	NA
193	P04181	OAT	Ornithine amino	sp|P04181|OAT_HUMAN O	11	33.9	0.1538625	0.63291	0.9984	0.278	0.63291	0.99836	6	19.8	-0.4626	NA	NA	NA	NA	NA	NA
194	P04216	THY1	Thy-1 membrane	sp|P04216|THY1_HUMAN T	2	15.5	-1.32124	0.05474	0.9984	0.1345	0.05474	0.99836	3	24.2	NA	NA	NA	NA	NA	NA	NA
195	P04350	TUBB4A	Tubulin beta-4A	sp|P04350|TBB4A_HUMAN	13	36.5	-0.606018	NA	NA	NA	NA	NA	20	63.7	NA	NA	NA	NA	NA	NA	NA
196	P04406	GAPDH	Glyceraldehyde-	sp|P04406|G3P_HUMAN G	22	83.6	-0.507128	0.02233	0.9991	-1.33	5.30E-06	0.00188	19	78.5	-0.2114	0.30817	0.9973	0.79327	0.0011	0.0035	2
197	P04792	HSPB1	Heat shock prot	sp|P04792|HSPB1_HUMAN	3	19.5	0.0452162	0.93178	0.9991	0.7637	0.16276	0.33643	11	65.9	0.15594	0.76815	0.9973	1.67205	0.006	0.0144	1
198	P04843	RPN1	Dolichyl-diphosp	sp|P04843|RPN1_HUMAN	22	41.2	-0.366745	0.07319	0.9991	0.2314	0.24416	0.44232	22	45.6	-0.0265	0.89153	0.9973	0.38166	0.0633	0.1073	NA
199	P04844	RPN2	Dolichyl-diphosp	sp|P04844|RPN2_HUMAN	11	24.7	-0.204137	0.47797	0.9991	0.0592	0.8358	0.91731	10	25.7	0.14396	0.61539	0.9973	-0.1248	0.6629	0.7425	NA
200	P04899	GNAI2	Guanine nucleot	sp|P04899|GNAI2_HUMAN	8	26.2	0.1150815	0.75046	0.9991	0.6046	0.10822	0.278	9	32.1	0.21827	0.54801	0.9973	-2.0791	######	0.0001	4
201	Q99878	HIST1H2AJ	Histone H2A typ	sp|Q99878|H2A1J_HUMAN	7	38.3	-0.050322	0.92912	0.999	0.0309	0.92912	0.99899	NA	NA	NA	NA	NA	NA	NA	NA	NA
202	P05023	ATP1A1	Sodium/potassiu	sp|P05023|AT1A1_HUMAN	29	35.1	-0.155844	0.40574	0.9991	0.5155	0.01205	0.07061	28	35.2	0.11991	0.52052	0.9973	-0.3075	0.1112	0.1721	5
203	P05026	ATP1B1	Sodium/potassiu	sp|P05026|AT1B1_HUMAN	3	16.5	0.2166157	0.64022	0.9984	1.1638	0.64022	0.99836	2	8.9	NA	NA	NA	NA	NA	NA	NA
204	P05067	APP	Amyloid-beta pr	sp|P05067|A4_HUMAN Am	4	8.6	-0.299157	0.36695	0.9984	0.4322	0.36695	0.99836	1	2.1	NA	NA	NA	NA	NA	NA	NA
205	P05091	ALDH2	Aldehyde dehyd	sp|P05091|ALDH2_HUMAN	3	8.5	NA	NA	NA	NA	NA	NA	8	23.8	0.10338	0.60158	0.9991	-0.396	0.6016	0.9991	NA
206	P05114	HMGN1	Non-histone chr	sp|P05114|HMGN1_HUMA	4	43	0.5857978	0.14053	0.9984	1.181	0.14053	0.99836	5	47	NA	NA	NA	NA	NA	NA	NA
207	P05141	SLC25A5	ADP/ATP transl	sp|P05141|ADT2_HUMAN	9	30.9	0.4006282	0.17355	0.9991	0.8257	0.00958	0.06292	13	36.9	-0.0573	0.84131	0.9973	-0.161	0.5753	0.6665	5
208	P05186	ALPL	Alkaline phosph	sp|P05186|PPBT_HUMAN A	4	8	-0.091623	0.82359	0.9984	0.2016	0.82359	0.99836	NA	NA	NA	NA	NA	NA	NA	NA	NA
209	P05198	EIF2S1	Eukaryotic trans	sp|P05198|IF2A_HUMAN E	4	12.7	-0.158688	0.59892	0.9991	-0.0014	0.99662	0.99662	10	39.4	-0.8854	0.00885	0.7846	-1.4829	0.0001	0.0006	4
210	P05386	RPLP1	60S acidic ribos	sp|P05386|RLA1_HUMAN 6	4	89.5	-0.110846	0.72858	0.9991	-1.0317	0.00989	0.06319	4	89.5	0.2622	0.41598	0.9973	1.11491	0.0028	0.0077	2
211	P05387	RPLP2	60S acidic ribos	sp|P05387|RLA2_HUMAN 6	5	74.8	0.3040909	0.22276	0.9991	-0.1253	0.60843	0.75813	6	74.8	0.15459	0.52831	0.9973	0.28941	0.2449	0.3295	NA
212	P05388	RPLP0	60S acidic ribos	sp|P05388|RLA0_HUMAN 6	10	43.2	-0.095054	0.77673	0.9991	-0.5384	0.12152	0.2853	13	53.3	0.05038	0.88043	0.9973	-0.4318	0.2083	0.2937	NA
213	P05455	SSB	Lupus La protein	sp|P05455|LA_HUMAN Lup	13	30.9	0.1799018	0.55391	0.9991	0.4373	0.1608	0.33626	17	42.4	0.97745	0.00457	0.6565	2.43286	######	######	1
214	P05556	ITGB1	Integrin beta-1	sp|P05556|ITB1_HUMAN In	8	10.8	-0.278525	0.33691	0.9991	-0.0314	0.91249	0.94965	14	21.4	-0.3744	0.20184	0.9973	-0.9639	0.0034	0.0089	4
215	P06454	PTMA	Prothymosin alp	sp|P06454|PTMA_HUMAN	7	37.8	0.1981903	0.36165	0.9991	-0.1155	0.59193	0.7481	7	37.8	-0.4325	0.05695	0.9973	-0.7087	0.0039	0.0101	4
216	P06493	CDK1	Cyclin-dependen	sp|P06493|CDK1_HUMAN C	8	33	-0.170674	0.45779	0.9991	-0.2065	0.37085	0.57843	14	48.5	-0.2299	0.32054	0.9973	-0.2943	0.2079	0.2937	NA
217	P06576	ATP5F1B	ATP synthase su	sp|P06576|ATPB_HUMAN	21	59.4	-0.139508	0.57136	0.9991	-0.0637	0.79534	0.89461	24	67.7	0.15911	0.51917	0.9973	-1.0448	0.0005	0.0018	4
218	P06730	EIF4E	Eukaryotic trans	sp|P06730|IF4E_HUMAN E	3	18.4	-0.201264	NA	NA	NA	NA	NA	5	26.7	0.00977	0.96684	0.9991	-0.5873	0.9668	0.9991	NA
219	P06733	ENO1	Alpha-enolase	sp|P06733|ENOA_HUMAN	26	72.8	-0.219407	0.24199	0.9991	-0.9954	4.41E-05	0.00447	30	70.5	0.27688	0.14463	0.9973	0.61982	0.0033	0.0089	2
220	P06737	PYGL	Glycogen phosph	sp|P06737|PYGL_HUMAN C	5	8.9	0.0022877	0.99096	0.999	-0.3105	0.99096	0.99899	6	9.6	0.15751	NA	NA	NA	NA	NA	NA
221	P06744	GPI	Glucose-6-phosp	sp|P06744|G6PI_HUMAN G	15	28.5	0.0702974	0.81611	0.9991	-1.1051	0.00182	0.02746	17	36	0.13035	0.66696	0.9973	2.34144	######	######	2
222	P06748	NPM1	Nucleophosmin	sp|P06748|NPM_HUMAN N	13	53.7	0.1923145	0.39265	0.9991	0.404	0.0833	0.23529	14	57.1	0.16656	0.45781	0.9973	0.51516	0.0315	0.0585	1
223	P06753	TPM3	Tropomyosin alp	sp|P06753|TPM3_HUMAN	19	48.4	-0.185646	0.55871	0.9991	-0.6062	0.0686	0.20962	23	55.4	1.01917	0.00463	0.6565	3.33408	######	######	2
224	P07099	EPHX1	Epoxide hydrola	sp|P07099|HYEP_HUMAN	3	7.5	0.118861	0.8071	0.9984	-0.2797	0.8071	0.99836	NA	NA	NA	NA	NA	NA	NA	NA	NA
225	P07108	DBI	Acyl-CoA-binding	sp|P07108|ACBP_HUMAN	3	51.7	-0.052133	0.86285	0.9984	-1.3607	0.86285	0.99836	5	60.9	NA	NA	NA	NA	NA	NA	NA
226	P07195	LDHB	L-lactate dehydr	sp|P07195|LDHB_HUMAN	17	51.8	0.0997277	0.57355	0.9991	-0.7345	0.00068	0.01867	17	59	0.10927	0.58117	0.9973	4.78954	######	######	2
227	P07237	P4HB	Protein disulfide	sp|P07237|PDIA1_HUMAN	23	54.3	-0.030137	0.85527	0.9991	0.6208	0.00146	0.02711	33	68.3	0.06511	0.69409	0.9973	-1.0359	######	######	5
228	P07339	CTSD	Cathepsin D	sp|P07339|CATD_HUMAN	6	16	-0.36317	0.36776	0.9984	-0.0334	0.36776	0.99836	8	26.2	NA	NA	NA	NA	NA	NA	NA
229	P07355	ANXA2	Annexin A2	sp|P07355|ANXA2_HUMAN	13	46.3	-0.145036	0.4544	0.9991	-0.2334	0.235	0.43276	29	75.8	0.19826	0.31008	0.9973	-0.9284	0.0001	0.0006	4
230	P07384	CAPN1	Calpain-1 cataly	sp|P07384|CAN1_HUMAN	5	8	-0.26188	NA	NA	NA	NA	NA	2	3.4	NA	NA	NA	NA	NA	NA	NA
231	P07437	TUBB	Tubulin beta cha	sp|P07437|TBB5_HUMAN T	17	43.9	-0.09246	0.73834	0.9991	-1.2585	0.00027	0.00995	22	64.6	-0.3136	0.26571	0.9973	1.2455	0.0003	0.0012	2
232	P07737	PFN1	Profilin-1	sp|P07737|PROF1_HUMAN	11	91.4	0.0462088	0.85981	0.9991	-1.2131	0.00022	0.00987	11	91.4	0.409	0.13141	0.9973	0.2072	0.4326	0.5252	3
233	P07741	APRT	Adenine phosph	sp|P07741|APT_HUMAN A	2	13.3	-0.018778	0.9368	0.999	-0.985	0.9368	0.99899	6	44.4	NA	NA	NA	NA	NA	NA	NA
234	P07814	EPRS	Bifunctional glut	sp|P07814|SYEP_HUMAN E	28	23.4	0.0686195	0.76127	0.9991	0.153	0.50062	0.67575	39	33	-0.0364	0.8717	0.9973	1.57422	######	######	1
235	P07900	HSP90AA1	Heat shock prot	sp|P07900|HS90A_HUMAN	44	61.1	0.1027725	0.59477	0.9991	-0.6735	0.00256	0.03128	46	65.4	-0.1737	0.37248	0.9973	1.22436	######	######	2
236	P07910	HNRNPC	Heterogeneous	sp|P07910|HNRPC_HUMAN	19	60.1	0.5275696	0.0105	0.9991	0.8062	0.00041	0.0133	16	54.2	0.13311	0.47634	0.9973	0.68358	0.0017	0.005	1

237	P07942	LAMB1	Laminin subunit	sp|P07942|LAMB1_HUMAI	2	1.3	NA	NA	NA	NA	NA	NA	10	8.6	-0.3427	0.21515	0.9991	-0.8864	0.2152	0.9991	NA
238	P07954	FH	Fumarate hydra	sp|P07954|FUMH_HUMAN	9	25.5	-0.226037	0.33246	0.9991	0.5129	0.05991	0.19484	12	30.2	-0.0401	0.86148	0.9973	-0.199	0.3918	0.4865	NA
239	P08133	ANXA6	Annexin A6	sp|P08133|ANXA6_HUMAI	5	10.3	0.3921825	0.35555	0.9991	-1.36	0.00942	0.06292	14	25.7	-0.0404	0.91389	0.9973	-3.0326	######	######	4
240	P08195	SLC3A2	4F2 cell-surface	sp|P08195|4F2_HUMAN 4F	10	23.7	-0.057374	0.84391	0.9991	0.6813	0.03007	0.12709	11	25.7	0.07971	0.78455	0.9973	1.34862	0.0002	0.0009	1
241	P08238	HSP90AB1	Heat shock prot	sp|P08238|HS90B_HUMAN	50	64.1	-0.125823	0.47631	0.9991	-0.6031	0.00292	0.03391	54	69.3	-0.0951	0.58916	0.9973	-0.2896	0.1123	0.1731	3
242	P08243	ASNS	Asparagine synt	sp|P08243|ASNS_HUMAN	5	12.7	0.1805083	NA	NA	NA	NA	NA	7	13.9	-0.5636	NA	NA	NA	NA	NA	NA
243	P08397	HMBS	Porphobilinogen	sp|P08397|HEM3_HUMAN	3	8.3	0.4306566	0.226	0.9984	0.4442	0.226	0.99836	2	8.3	NA	NA	NA	NA	NA	NA	NA
244	P08559	PDHA1	Pyruvate dehyd	sp|P08559|ODPA_HUMAN	6	14.6	-0.291192	0.3381	0.9984	0.328	0.3381	0.99836	3	7.7	NA	NA	NA	NA	NA	NA	NA
245	P08574	CYC1	Cytochrome c1,	sp|P08574|CY1_HUMAN Cy	5	22.2	0.1272632	0.59804	0.9984	0.5379	0.59804	0.99836	4	15.7	NA	NA	NA	NA	NA	NA	NA
246	P08621	SNRNP70	U1 small nuclear	sp|P08621|RU17_HUMAN	4	12.4	0.1600065	0.61862	0.9991	0.3141	0.33366	0.53481	12	27.5	-0.1064	0.74001	0.9973	0.17381	0.5889	0.6768	NA
247	P08670	VIM	Vimentin	sp|P08670|VIME_HUMAN	7	14.6	0.0669774	0.7953	0.9991	0.7059	0.007	0.05274	51	83.9	0.06176	0.78912	0.9973	0.13953	0.5477	0.6397	5
248	P08708	RPS17	40S ribosomal p	sp|P08708|RS17_HUMAN 4	9	62.2	0.2241301	0.52623	0.9991	-0.7214	0.05314	0.18027	8	62.2	0.3075	0.38716	0.9973	-0.0492	0.8888	0.908	NA
249	P08758	ANXA5	Annexin A5	sp|P08758|ANXA5_HUMAI	13	43.8	-0.703192	0.06919	0.9991	-1.5771	0.00046	0.01422	16	53.8	-0.1175	0.74929	0.9973	0.26111	0.4804	0.5723	3
250	P08865	RPSA	40S ribosomal p	sp|P08865|RSSA_HUMAN	12	52.5	0.1770305	0.32893	0.9991	-0.2108	0.24781	0.44594	12	52.5	0.0156	0.93041	0.9973	-1.168	######	######	4
251	P09012	SNRPA	U1 small nuclear	sp|P09012|SNRPA_HUMAN	6	22	-0.272892	0.31494	0.9991	-0.1712	0.52455	0.69224	7	27.7	-0.4049	0.1431	0.9973	0.34362	0.2098	0.2948	NA
252	P09038	FGF2	Fibroblast grow	sp|P09038|FGF2_HUMAN	3	9.7	0.0892984	0.80778	0.9984	0.758	0.80778	0.99836	NA	NA	NA	NA	NA	NA	NA	NA	NA
253	P09211	GSTP1	Glutathione S-tr	sp|P09211|GSTP1_HUMAN	13	70.5	-0.001004	0.99872	0.9993	-1.8167	0.99872	0.99926	9	49	NA	NA	NA	NA	NA	NA	NA
254	P09417	QDPR	Dihydropteridin	sp|P09417|DHPR_HUMAN	3	18.4	-0.052716	NA	NA	NA	NA	NA	6	34.8	0.08425	0.74969	0.9991	0.64599	0.7497	0.9991	NA
255	P09429	HMGB1	High mobility gr	sp|P09429|HMGB1_HUMA	8	32.6	0.1241136	0.68659	0.9991	-0.3275	0.29419	0.49544	12	50.2	-0.121	0.69392	0.9973	1.77134	######	0.0001	2
256	P09493	TPM1	Tropomyosin alp	sp|P09493|TPM1_HUMAN	19	55.6	0.1868024	0.43613	0.9984	0.1366	0.43613	0.99836	13	32.4	NA	NA	NA	NA	NA	NA	NA
257	P09496	CLTA	Clathrin light ch	sp|P09496|CLCA_HUMAN C	5	16.5	-0.080129	0.79363	0.9991	-0.5598	0.18731	0.3689	7	16.5	-0.5714	0.05252	0.9973	0.68683	0.0234	0.0458	2
258	P09622	DLD	Dihydrolipoyl de	sp|P09622|DLDH_HUMAN	8	17.3	0.0764109	0.66062	0.9991	0.5243	0.00721	0.05274	10	29.1	0.26327	0.14251	0.9973	0.43667	0.0209	0.0415	1
259	P09651	HNRNPA1	Heterogeneous	sp|P09651|ROA1_HUMAN	17	40.6	-0.466346	0.23532	0.9991	-0.3883	0.3199	0.51783	19	48.1	0.06532	0.8651	0.9973	1.7231	0.0003	0.0012	2
260	P09661	SNRPA1	U2 small nuclear	sp|P09661|RU2A_HUMAN	3	19.2	NA	NA	NA	NA	NA	NA	6	43.5	-0.2014	0.6932	0.9991	1.83367	0.6932	0.9991	NA
261	P09669	COX6C	Cytochrome c ox	sp|P09669|COX6C_HUMAN	3	29.3	0.6424912	NA	NA	NA	NA	NA	NA	NA	NA	NA	NA	NA	NA	NA	NA
262	P09874	PARP1	Poly [ADP-ribose	sp|P09874|PARP1_HUMAN	32	35.9	0.0055248	0.98124	0.9991	0.212	0.37284	0.57843	32	37	0.14779	0.53191	0.9973	2.31806	######	######	1
263	P09936	UCHL1	Ubiquitin carbox	sp|P09936|UCHL1_HUMAN	12	57.4	0.0757996	0.79284	0.9991	-0.9829	0.00363	0.03699	15	83.4	0.51213	0.22143	0.9973	7.68301	######	######	2
264	P09960	LTA4H	Leukotriene A-4	sp|P09960|LKHA4_HUMAN	13	26.2	-0.046711	0.83382	0.9984	-0.4886	0.83382	0.99836	11	23.2	NA	NA	NA	NA	NA	NA	NA
265	P09972	ALDOC	Fructose-bisphos	sp|P09972|ALDOC_HUMAN	5	25.5	0.2527065	NA	NA	NA	NA	NA	10	36.5	-0.1821	0.80532	0.9991	1.77515	0.8053	0.9991	NA
266	Q71UI9	H2AFV	Histone H2A.V	sp|Q71UI9|H2AV_HUMAN	5	31.2	0.5477235	0.22309	0.9991	0.3309	0.45506	0.64015	6	53.9	0.62535	0.16708	0.9973	2.42658	######	0.0002	1
267	P0CG35	TMSB15B	Thymosin beta-1	sp|P0CG35|TB15B_HUMAN	2	40	0.6709544	0.01493	0.9984	-0.3646	0.01493	0.99836	NA	NA	NA	NA	NA	NA	NA	NA	NA
268	P62987	UBA52	Ubiquitin-60S rib	sp|P62987|RL40_HUMAN U	6	43	-0.004137	0.98076	0.9991	0.2771	0.12011	0.2853	7	47.4	0.16015	0.35703	0.9973	-0.2451	0.1658	0.2429	NA
269	P0DMV9	HSPA1B	Heat shock 70 k	sp|P0DMV9|HS71B_HUMA	17	35.1	-0.132933	0.47877	0.9984	0.2599	0.47877	0.99836	33	68.8	NA	NA	NA	NA	NA	NA	NA
270	P0DN76	U2AF1L5	Splicing factor U	sp|P0DN76|U2AF5_HUMA	2	15	NA	NA	NA	NA	NA	NA	4	25.8	NA	NA	NA	-0.5205	NA	NA	NA
271	P35520	CBS	Cystathionine be	sp|P35520|CBS_HUMAN Cy	9	20.7	0.1552582	0.45484	0.9984	-0.0986	0.45484	0.99836	3	6.2	NA	NA	NA	NA	NA	NA	NA
272	P0DP25	CALM3	Calmodulin-3	sp|P0DP25|CALM3_HUMA	10	70.5	-0.02015	0.93831	0.9991	-0.1763	0.50133	0.67575	12	85.2	0.08331	0.74937	0.9973	0.4446	0.1017	0.1603	NA
273	P10412	HIST1H1E	Histone H1.4	sp|P10412|H14_HUMAN H	6	29.2	NA	NA	NA	NA	NA	NA	9	37	-0.3583	0.21713	0.9991	1.94857	0.2171	0.9991	NA
274	P10515	DLAT	Dihydrolipoyllys	sp|P10515|ODP2_HUMAN	7	12.4	0.4686035	0.06584	0.9984	0.7339	0.06584	0.99836	5	9.6	0.00198	NA	NA	NA	NA	NA	NA
275	P10599	TXN	Thioredoxin	sp|P10599|THIO_HUMAN T	4	38.1	0.2512582	0.39643	0.9984	-0.1019	0.39643	0.99836	5	46.7	NA	NA	NA	NA	NA	NA	NA
276	P10606	COX5B	Cytochrome c ox	sp|P10606|COX5B_HUMAN	4	30.2	0.4048637	0.31198	0.9984	0.4973	0.31198	0.99836	4	30.2	NA	NA	NA	NA	NA	NA	NA
277	P10620	MGST1	Microsomal glut	sp|P10620|MGST1_HUMA	4	42.6	-0.387348	0.25027	0.9984	0.5893	0.25027	0.99836	NA	NA	NA	NA	NA	NA	NA	NA	NA
278	P10768	ESD	S-formylglutathi	sp|P10768|ESTD_HUMAN	5	24.5	0.1721963	NA	NA	NA	NA	NA	7	36.9	NA	NA	NA	NA	NA	NA	NA
279	P10809	HSPD1	60 kDa heat sho	sp|P10809|CH60_HUMAN	34	69.6	-0.100582	0.51497	0.9991	0.3042	0.06091	0.19558	40	79.1	0.0917	0.55233	0.9973	0.77039	0.0001	0.0005	1
280	P11047	LAMC1	Laminin subunit	sp|P11047|LAMC1_HUMAI	5	3.9	NA	NA	NA	NA	NA	NA	13	10.8	0.09007	0.73132	0.9991	-0.0574	0.7313	0.9991	NA
281	P11142	HSPA8	Heat shock cogn	sp|P11142|HSP7C_HUMAN	43	66.1	0.0017076	0.99247	0.9991	-0.1528	0.40376	0.60515	46	77.6	0.03232	0.85831	0.9973	-0.6577	0.0019	0.0055	4
282	P11166	SLC2A1	Solute carrier fa	sp|P11166|GTR1_HUMAN	3	7.7	-0.77599	0.02478	0.9984	0.1789	0.02478	0.99836	3	7.7	NA	NA	NA	NA	NA	NA	NA
283	P11169	SLC2A3	Solute carrier fa	sp|P11169|GTR3_HUMAN	6	13.1	-0.19015	0.31291	0.9984	0.8211	0.31291	0.99836	NA	NA	NA	NA	NA	NA	NA	NA	NA
284	P11177	PDHB	Pyruvate dehyd	sp|P11177|ODPB_HUMAN	6	18.1	0.0110086	0.96394	0.9991	0.3877	0.12597	0.28904	5	17.3	0.4279	0.09384	0.9973	0.33957	0.2238	0.3074	NA

285	P11216	PYGB	Glycogen phospl	sp|P11216|PYGB_HUMAN (	5	9.4	0.0833934	0.86442	0.9991	0.0036	0.99473	0.99662	9	14.4	0.32747	0.50569	0.9973	-2.9092	######	0.0004	4
286	P11279	LAMP1	Lysosome-associ	sp|P11279|LAMP1_HUMAI	3	3.6	-0.043909	0.85567	0.9984	-0.3205	0.85567	0.99836	5	10.3	NA	NA	NA	NA	NA	NA	NA
287	P11310	ACADM	Medium-chain sp	sp|P11310|ACADM_HUMA	5	18.8	-0.529681	NA	NA	NA	NA	NA	5	15.4	NA	NA	NA	NA	NA	NA	NA
288	P11387	TOP1	DNA topoisomer	sp|P11387|TOP1_HUMAN	16	23	0.154543	0.6779	0.9991	0.6117	0.11318	0.27928	17	26.5	0.08357	0.82196	0.9973	4.55437	######	######	1
289	P11388	TOP2A	DNA topoisomer	sp|P11388|TOP2A_HUMAN	37	27.4	0.0994441	0.56305	0.9991	0.6123	0.00216	0.02915	21	17.4	-0.1645	0.34312	0.9973	-2.316	######	######	4
290	P11413	G6PD	Glucose-6-phospl	sp|P11413|G6PD_HUMAN	3	6.4	0.971423	0.10286	0.9991	0.242	0.6697	0.80752	15	38.3	0.2336	0.74439	0.9973	5.26071	######	######	1
291	P11586	MTHFD1	C-1-tetrahydrofo	sp|P11586|C1TC_HUMAN (	29	38.3	0.0571115	0.81624	0.9991	-0.9245	0.00145	0.02711	25	35	0.17357	0.48307	0.9973	1.75245	######	######	2
292	P11766	ADH5	Alcohol dehydro	sp|P11766|ADHX_HUMAN	10	27.3	0.1899945	0.3514	0.9984	-0.5459	0.3514	0.99836	14	32.9	-0.1994	NA	NA	NA	NA	NA	NA
293	P11908	PRPS2	Ribose-phosphat	sp|P11908|PRPS2_HUMAN	4	15.7	NA	NA	NA	NA	NA	NA	5	23.3	0.0424	0.92169	0.9991	-0.7584	0.9217	0.9991	NA
294	P11940	PABPC1	Polyadenylate-b	sp|P11940|PABP1_HUMAN	22	44	-0.039889	0.81738	0.9991	0.0712	0.68077	0.81669	27	51.6	0.07752	0.65435	0.9973	-0.3842	0.0378	0.0685	4
295	P12004	PCNA	Proliferating cell	sp|P12004|PCNA_HUMAN	13	64.8	0.1560578	0.32576	0.9991	-0.1541	0.33174	0.53334	16	74.7	-0.0767	0.62499	0.9973	0.3144	0.0577	0.0991	NA
296	P12081	HARS	Histidine--tRNA l	sp|P12081|SYHC_HUMAN	3	5.9	NA	NA	NA	NA	NA	NA	14	28.5	0.29238	0.39902	0.9991	0.7986	0.399	0.9991	NA
297	P12236	SLC25A6	ADP/ATP translo	sp|P12236|ADT3_HUMAN	9	30.9	-0.134256	0.39522	0.9984	0.2189	0.39522	0.99836	12	45.6	NA	NA	NA	NA	NA	NA	NA
298	P12268	IMPDH2	Inosine-5-monop	sp|P12268|IMDH2_HUMAI	13	30.2	-0.242938	0.19463	0.9991	-0.6414	0.00579	0.04664	15	36.4	-0.0649	0.72215	0.9973	-0.8725	0.0002	0.0008	3
299	P12270	TPR	Nucleoprotein T	sp|P12270|TPR_HUMAN N	29	15.2	0.0324959	0.89953	0.9991	0.8222	0.00496	0.0419	40	21.1	0.13717	0.59558	0.9973	0.45662	0.09	0.1457	1
300	P12277	CKB	Creatine kinase	sp|P12277|KCRB_HUMAN (	15	47.5	0.1023328	0.80999	0.9984	-1.3136	0.80999	0.99836	19	65.4	NA	NA	NA	4.88944	NA	NA	NA
301	P12429	ANXA3	Annexin A3	sp|P12429|ANXA3_HUMAI	6	26	-0.035262	NA	NA	NA	NA	NA	1	2.8	NA	NA	NA	NA	NA	NA	NA
302	P12532	CKMT1A	Creatine kinase	sp|P12532|KCRU_HUMAN (	2	4.6	0.2043584	NA	NA	NA	NA	NA	12	45.6	NA	NA	NA	NA	NA	NA	NA
303	P12814	ACTN1	Alpha-actinin-1	sp|P12814|ACTN1_HUMAN	24	36.8	0.033231	0.85824	0.9991	-0.3143	0.10498	0.27366	38	54.1	-0.0439	0.81373	0.9973	0.12718	0.4972	0.5865	NA
304	P12830	CDH1	Cadherin-1	sp|P12830|CADH1_HUMAI	3	3.6	NA	NA	NA	0.6329	NA	NA	NA	NA	NA	NA	NA	NA	NA	NA	NA
305	P12955	PEPD	Xaa-Pro dipeptid	sp|P12955|PEPD_HUMAN)	4	8.7	0.3817417	0.16415	0.9984	0.0024	0.16415	0.99836	5	14.4	NA	NA	NA	NA	NA	NA	NA
306	P12956	XRCC6	X-ray repair cros	sp|P12956|XRCC6_HUMAN	26	52.9	-0.007406	0.97756	0.999	-0.18	0.97756	0.99899	28	51.6	NA	NA	NA	7.75173	NA	NA	NA
307	P13010	XRCC5	X-ray repair cros	sp|P13010|XRCC5_HUMAN	25	44.5	0.194584	0.22954	0.9984	0.0635	0.22954	0.99836	29	54.6	NA	NA	NA	NA	NA	NA	NA
308	P13073	COX4I1	Cytochrome c ox	sp|P13073|COX41_HUMAN	5	32.5	0.0115615	0.97267	0.999	0.2704	0.97267	0.99899	6	30.2	NA	NA	NA	NA	NA	NA	NA
309	P13639	EEF2	Elongation facto	sp|P13639|EF2_HUMAN El	42	53.8	-0.239822	0.2221	0.9991	-1.1793	1.07E-05	0.00239	49	61.1	0.13877	0.47305	0.9973	-0.28	0.1573	0.2319	3
310	P13667	PDIA4	Protein disulfide	sp|P13667|PDIA4_HUMAN	30	50.4	0.0921677	0.62496	0.9991	0.4008	0.04536	0.16324	28	49.9	-0.116	0.53943	0.9973	0.85714	0.0003	0.0011	1
311	P13674	P4HA1	Prolyl 4-hydroxy	sp|P13674|P4HA1_HUMAN	5	11	-1.37003	0.03042	0.9991	-0.5258	0.372	0.57843	11	27.7	0.02085	0.9595	0.9973	-0.0931	0.8207	0.8532	3
312	P13693	TPT1	Translationally-c	sp|P13693|TCTP_HUMAN T	4	20.9	NA	NA	NA	NA	NA	NA	9	42.4	0.55129	0.10847	0.9991	1.33487	0.1085	0.9991	NA
313	P13797	PLS3	Plastin-3	sp|P13797|PLST_HUMAN P	13	24.3	0.2593288	0.22319	0.9991	-0.6293	0.01448	0.07961	13	26.3	0.33041	0.12615	0.9973	-0.4301	0.0521	0.0906	3
314	P13798	APEH	Acylamino-acid-r	sp|P13798|ACPH_HUMAN	11	18.2	0.0572208	0.76996	0.9984	-0.4799	0.76996	0.99836	4	6.6	NA	NA	NA	NA	NA	NA	NA
315	P13804	ETFA	Electron transfer	sp|P13804|ETFA_HUMAN	9	37.8	-0.125697	0.52997	0.9984	0.1058	0.52997	0.99836	13	56.8	NA	NA	NA	NA	NA	NA	NA
316	P13984	GTF2F2	General transcri	sp|P13984|T2FB_HUMAN (	2	8.8	NA	NA	NA	NA	NA	NA	4	16.9	-0.0559	0.84586	0.9991	0.35061	0.8459	0.9991	NA
317	P14174	MIF	Macrophage mig	sp|P14174|MIF_HUMAN M	2	17.4	-0.411429	0.34062	0.9984	-0.1614	0.34062	0.99836	3	35.7	NA	NA	NA	NA	NA	NA	NA
318	P14314	PRKCSH	Glucosidase 2 su	sp|P14314|GLU2B_HUMAN	11	21	-0.12271	0.72839	0.9991	0.2346	0.50885	0.68136	17	37.9	0.21629	0.54208	0.9973	4.76379	######	######	1
319	P14324	FDPS	Farnesyl pyroph	sp|P14324|FPPS_HUMAN F	5	14.3	-0.295937	0.26857	0.9984	-1.3852	0.26857	0.99836	5	14.3	NA	NA	NA	NA	NA	NA	NA
320	P14550	AKR1A1	Aldo-keto reduc	sp|P14550|AK1A1_HUMAN	7	28	0.0561529	0.79115	0.9991	-0.5566	0.03012	0.12709	11	45.5	0.04337	0.83784	0.9973	-1.234	######	0.0002	4
321	P14618	PKM	Pyruvate kinase	sp|P14618|KPYM_HUMAN	34	65.5	0.0016644	0.9934	0.9991	-1.1104	3.65E-05	0.00432	41	76.5	0.05095	0.80033	0.9973	-0.1096	0.5877	0.6765	3
322	P14625	HSP90B1	Endoplasmin	sp|P14625|ENPL_HUMAN E	42	51.7	-0.128611	0.38569	0.9991	0.5414	0.00168	0.02746	44	58.8	0.0044	0.97602	0.9973	-0.4411	0.0074	0.017	5
323	P14635	CCNB1	G2/mitotic-speci	sp|P14635|CCNB1_HUMAN	2	4.8	0.4332965	NA	NA	NA	NA	NA	1	3	NA	NA	NA	NA	NA	NA	NA
324	P14735	IDE	Insulin-degradin	sp|P14735|IDE_HUMAN In	5	5.6	-0.205008	NA	NA	NA	NA	NA	8	9.2	-0.12	NA	NA	NA	NA	NA	NA
325	P14866	HNRNPL	Heterogeneous	sp|P14866|HNRPL_HUMAN	18	45.7	0.2204447	0.26721	0.9991	0.3619	0.0772	0.22519	19	46.9	0.16256	0.40918	0.9973	0.11611	0.5535	0.6454	NA
326	P14868	DARS	Aspartate--tRNA	sp|P14868|SYDC_HUMAN /	15	34.5	0.1480266	0.45848	0.9991	-0.0126	0.95462	0.96897	18	42.7	0.07239	0.71504	0.9973	0.21666	0.2828	0.3679	NA
327	P15121	AKR1B1	Aldo-keto reduc	sp|P15121|ALDR_HUMAN /	4	13.9	0.3509399	0.31031	0.9991	-0.5841	0.13179	0.29758	8	25.6	0.31887	0.30297	0.9973	-0.5173	0.1044	0.1638	NA
328	P63000	RAC1	Ras-related C3 b	sp|P63000|RAC1_HUMAN	2	10.9	NA	NA	NA	NA	NA	NA	7	43.2	0.17321	0.43634	0.9991	0.87262	0.4363	0.9991	NA
329	P15170	GSPT1	Eukaryotic pept	sp|P15170|ERF3A_HUMAN	8	21.8	0.0908704	0.62314	0.9991	-0.2196	0.24333	0.44232	13	30.7	0.12701	0.49373	0.9973	0.12023	0.5168	0.6086	NA
330	P15311	EZR	Ezrin	sp|P15311|EZRI_HUMAN E	26	44.2	-0.061206	0.76705	0.9991	-0.734	0.00227	0.02957	16	27.6	-0.0427	0.83624	0.9973	-0.8865	0.0005	0.0017	3
331	P15374	UCHL3	Ubiquitin carbox	sp|P15374|UCHL3_HUMAN	5	30	0.7717067	NA	NA	NA	NA	NA	5	31.3	NA	NA	NA	NA	NA	NA	NA
332	P15531	NME1	Nucleoside diphc	sp|P15531|NDKA_HUMAN	8	55.3	0.0001923	0.99938	0.9994	-0.4953	0.05787	0.18907	10	78.9	0.54746	0.03821	0.9973	1.95601	######	######	2

333	P15880	RPS2	40S ribosomal p	sp|P15880|RS2_HUMAN 40	13	48.5	0.001913	0.99369	0.9991	-0.442	0.08156	0.23223	13	47.8	0.24016	0.3279	0.9973	-0.747	0.0062	0.0149	4
334	P15927	RPA2	Replication prot	sp|P15927|RFA2_HUMAN	4	23.7	0.3206292	0.28314	0.9984	0.3332	0.28314	0.99836	4	23.7	0.07096	NA	NA	NA	NA	NA	NA
335	P15954	COX7C	Cytochrome c ox	sp|P15954|COX7C_HUMAN	2	28.6	0.1621164	0.45707	0.9991	0.8081	0.00167	0.02746	2	28.6	-0.0282	0.89627	0.9973	-0.9487	0.0011	0.0036	5
336	P16083	NQO2	Ribosyldihydron	sp|P16083|NQO2_HUMAN	5	34.2	0.1111756	0.71499	0.9984	-0.1541	0.71499	0.99836	NA	NA	NA	NA	NA	NA	NA	NA	NA
337	P16104	H2AFX	Histone H2AX	sp|P16104|H2AX_HUMAN	6	37.8	NA	NA	NA	NA	NA	NA	8	63.6	0.25704	0.234	0.9991	1.34736	0.234	0.9991	NA
338	P16152	CBR1	Carbonyl reduct	sp|P16152|CBR1_HUMAN (	9	51.3	0.1290811	0.80126	0.9991	-1.0026	0.09852	0.26094	14	63.2	0.55225	0.4769	0.9973	3.21039	######	0.0004	2
339	P16401	HIST1H1B	Histone H1.5	sp|P16401|H15_HUMAN H	7	31.9	0.3951639	0.40047	0.9991	0.8281	0.08884	0.24227	8	31.4	-0.4883	0.30157	0.9973	5.03875	######	######	1
340	P10412	HIST1H1E	Histone H1.4	sp|P10412|H14_HUMAN H	6	29.2	NA	NA	NA	NA	NA	NA	9	37	-0.3583	0.21713	0.9991	1.94857	0.2171	0.9991	NA
341	P16403	HIST1H1C	Histone H1.2	sp|P16403|H12_HUMAN H	6	30	0.6184023	0.11797	0.9984	0.8605	0.11797	0.99836	8	34.3	NA	NA	NA	NA	NA	NA	NA
342	P16422	EPCAM	Epithelial cell ad	sp|P16422|EPCAM_HUMA	5	22.3	0.1386749	0.58905	0.9984	0.8398	0.58905	0.99836	NA	NA	NA	NA	NA	NA	NA	NA	NA
343	P16435	POR	NADPH--cytochr	sp|P16435|NCPR_HUMAN	6	9.6	0.1959273	0.47115	0.9984	0.6838	0.47115	0.99836	1	1.9	NA	NA	NA	NA	NA	NA	NA
344	P16615	ATP2A2	Sarcoplasmic/en	sp|P16615|AT2A2_HUMAN	16	19.8	0.0071777	0.97278	0.9991	0.4991	0.0281	0.12299	20	25	0.02543	0.9038	0.9973	-1.4758	######	######	5
345	P16949	STMN1	Stathmin	sp|P16949|STMN1_HUMA	8	51.7	0.2836435	0.44802	0.9991	-0.7212	0.06518	0.20269	9	51.7	0.06649	0.85765	0.9973	4.57461	######	######	2
346	P16989	YBX3	Y-box-binding pr	sp|P16989|YBOX3_HUMAN	8	31.5	0.3428282	0.37679	0.9984	0.7833	0.37679	0.99836	5	13.7	NA	NA	NA	NA	NA	NA	NA
347	P17096	HMGA1	High mobility gr	sp|P17096|HMGA1_HUMA	5	47.7	0.3816649	0.43154	0.9991	1.201	0.02167	0.10155	3	29.9	-0.4534	0.35199	0.9973	0.65969	0.182	0.2633	1
348	P17174	GOT1	Aspartate amino	sp|P17174|AATC_HUMAN	6	20.1	-0.010664	0.9595	0.999	-0.2172	0.9595	0.99899	9	31.2	NA	NA	NA	NA	NA	NA	NA
349	P17302	GJA1	Gap junction alp	sp|P17302|CXA1_HUMAN (	4	16.5	-0.568561	0.08082	0.9984	0.1792	0.08082	0.99836	NA	NA	NA	NA	NA	NA	NA	NA	NA
350	P17480	UBTF	Nucleolar transc	sp|P17480|UBF1_HUMAN	7	9.3	0.3325207	0.18882	0.9991	0.6964	0.0208	0.1003	10	14	0.14898	0.54684	0.9973	-0.2349	0.3463	0.4369	5
351	P17812	CTPS1	CTP synthase 1	sp|P17812|PYRG1_HUMAN	6	11.7	0.1839976	0.52863	0.9991	-0.7709	0.05108	0.17666	11	22.5	-0.1304	0.6167	0.9973	0.22688	0.3882	0.4829	NA
352	P17844	DDX5	Probable ATP-de	sp|P17844|DDX5_HUMAN	18	26.1	-0.059485	0.69164	0.9991	0.0905	0.54761	0.70721	24	40.2	0.07854	0.60117	0.9973	0.26236	0.0935	0.1511	NA
353	P17858	PFKL	ATP-dependent	sp|P17858|PFKAL_HUMAN	1	1.2	NA	NA	NA	NA	NA	NA	4	6.7	-0.1828	NA	NA	NA	NA	NA	NA
354	P17980	PSMC3	26S proteasome	sp|P17980|PRS6A_HUMAN	12	37.4	-0.17005	0.36412	0.9991	-0.2781	0.14588	0.31631	15	46.2	0.04221	0.81961	0.9973	-0.2075	0.2712	0.3554	NA
355	P17987	TCP1	T-complex prote	sp|P17987|TCPA_HUMAN	19	43.3	-0.182054	0.31355	0.9991	-0.5256	0.00828	0.05941	21	52.5	-0.1261	0.48153	0.9973	-0.7181	0.0008	0.0026	3
356	P18031	PTPN1	Tyrosine-protein	sp|P18031|PTN1_HUMAN	2	5.1	-0.161405	0.52208	0.9984	-0.1471	0.52208	0.99836	7	21.4	NA	NA	NA	0.22592	NA	NA	NA
357	P18077	RPL35A	60S ribosomal p	sp|P18077|RL35A_HUMAN	5	39.1	-0.354149	0.32412	0.9991	-0.4191	0.24609	0.44396	6	39.1	0.22751	0.52275	0.9973	-0.7605	0.0439	0.0774	4
358	P18085	ARF4	ADP-ribosylation	sp|P18085|ARF4_HUMAN	4	31.7	0.3106449	0.24395	0.9984	-0.2695	0.24395	0.99836	5	42.8	NA	NA	NA	NA	NA	NA	NA
359	P18124	RPL7	60S ribosomal p	sp|P18124|RL7_HUMAN 60	12	50.8	-0.124321	0.68834	0.9991	0.2454	0.43184	0.6164	8	38.7	-0.2758	0.37817	0.9973	-1.7585	######	0.0002	4
360	P18206	VCL	Vinculin	sp|P18206|VINC_HUMAN V	43	46.1	0.0761284	0.68514	0.9991	-0.5394	0.00975	0.06319	32	35.4	0.03563	0.84918	0.9973	-0.8331	0.0003	0.0013	4
361	P18583	SON	Protein SON	sp|P18583|SON_HUMAN P	14	8.5	-0.320601	0.21335	0.9984	0.002	0.21335	0.99836	12	7	NA	NA	NA	NA	NA	NA	NA
362	P18621	RPL17	60S ribosomal p	sp|P18621|RL17_HUMAN 6	7	42.9	0.3957694	0.22781	0.9991	0.6532	0.05482	0.18165	8	43.5	0.10115	0.75283	0.9973	-0.0407	0.8989	0.9131	NA
363	P18669	PGAM1	Phosphoglycerat	sp|P18669|PGAM1_HUMA	10	58.7	0.1802503	0.46219	0.9991	-0.6167	0.02005	0.09873	16	76	0.11872	0.62656	0.9973	-0.434	0.0882	0.1435	3
364	P18858	LIG1	DNA ligase 1	sp|P18858|DNLI1_HUMAN	9	13.6	0.2761236	0.1372	0.9984	0.0318	0.1372	0.99836	3	4.8	-0.2503	NA	NA	NA	NA	NA	NA
365	P18859	ATP5PF	ATP synthase-co	sp|P18859|ATP5J_HUMAN	3	30.6	-0.28669	0.39732	0.9984	0.2026	0.39732	0.99836	5	51.9	NA	NA	NA	NA	NA	NA	NA
366	P19174	PLCG1	1-phosphatidylin	sp|P19174|PLCG1_HUMAN	2	3	0.1632684	0.58807	0.9984	-0.1546	0.58807	0.99836	6	6.5	NA	NA	NA	NA	NA	NA	NA
367	P19338	NCL	Nucleolin	sp|P19338|NUCL_HUMAN	35	42.7	0.0715936	0.67336	0.9991	0.2623	0.13498	0.30186	34	40.7	0.16659	0.33245	0.9973	0.23332	0.1805	0.2618	NA
368	P19367	HK1	Hexokinase-1	sp|P19367|HXK1_HUMAN	15	17.1	-0.151523	0.581	0.9991	0.2989	0.28276	0.48307	17	20.3	0.2521	0.36245	0.9973	0.34805	0.2138	0.2996	NA
369	P19525	EIF2AK2	Interferon-induc	sp|P19525|E2AK2_HUMAN	2	4	0.0722518	0.86669	0.9984	0.2659	0.86669	0.99836	4	8.2	NA	NA	NA	NA	NA	NA	NA
370	P19623	SRM	Spermidine synt	sp|P19623|SPEE_HUMAN S	7	32.8	-0.146746	0.58014	0.9991	-0.5507	0.15477	0.32873	7	30.8	-0.0078	0.97643	0.9973	-0.9252	0.003	0.0082	4
371	P19784	CSNK2A2	Casein kinase II	sp|P19784|CSK22_HUMAN	3	11.4	-0.089389	0.76018	0.9984	0.3748	0.76018	0.99836	7	30.9	0.38055	NA	NA	NA	NA	NA	NA
372	P20020	ATP2B1	Plasma membra	sp|P20020|AT2B1_HUMAN	3	4.3	NA	NA	NA	NA	NA	NA	14	16.6	0.18055	0.54781	0.9991	-1.9137	0.5478	0.9991	NA
373	P20042	EIF2S2	Eukaryotic trans	sp|P20042|IF2B_HUMAN E	10	31.2	0.3244592	0.16697	0.9991	0.4005	0.09278	0.2501	12	41.7	0.04951	0.82804	0.9973	-1.1227	0.0001	0.0005	5
374	P20073	ANXA7	Annexin A7	sp|P20073|ANXA7_HUMAI	2	5.1	NA	NA	NA	NA	NA	NA	7	15.6	-0.0796	0.81551	0.9991	-0.1165	0.8155	0.9991	NA
375	P20290	BTF3	Transcription fa	sp|P20290|BTF3_HUMAN T	4	30.1	-0.383707	0.32904	0.9991	-0.2882	0.46044	0.6439	7	62.1	0.03852	0.91139	0.9973	-0.8347	0.0266	0.0506	4
376	P20340	RAB6A	Ras-related prot	sp|P20340|RAB6A_HUMAN	5	28.8	-0.319335	0.2995	0.9991	-0.2546	0.40521	0.60515	5	28.8	-0.3117	0.31088	0.9973	-0.6011	0.0604	0.103	NA
377	P20618	PSMB1	Proteasome sub	sp|P20618|PSB1_HUMAN F	3	18.3	0.1250305	NA	NA	NA	NA	NA	7	42.3	0.3342	0.60273	0.9991	-0.2006	0.6027	0.9991	NA
378	P20700	LMNB1	Lamin-B1	sp|P20700|LMNB1_HUMAI	36	59	-0.047077	0.80501	0.9991	0.5249	0.01271	0.07295	38	54.3	0.07188	0.70657	0.9973	0.07857	0.6809	0.7566	5
379	P20962	PTMS	Parathymosin	sp|P20962|PTMS_HUMAN	3	23.5	0.3658066	0.14436	0.9984	-0.0386	0.14436	0.99836	3	22.5	NA	NA	NA	NA	NA	NA	NA
380	P21281	ATP6V1B2	V-type proton A	sp|P21281|VATB2_HUMAN	2	5.1	NA	NA	NA	NA	NA	NA	11	27.4	0.43804	0.21351	0.9991	0.57752	0.2135	0.9991	NA

381	P21291	CSRP1	Cysteine and gly	sp|P21291|CSRP1_HUMAN	2	13	NA	NA	NA	NA	NA	NA	4	29.5	-0.1645	NA	NA	NA	NA	NA	NA
382	P21333	FLNA	Filamin-A	sp|P21333|FLNA_HUMAN	89	45.6	0.0557023	0.70869	0.9991	-0.2561	0.09917	0.26138	84	46.8	0.12606	0.40198	0.9973	-0.1511	0.3173	0.4046	NA
383	P21796	VDAC1	Voltage-depend	sp|P21796|VDAC1_HUMAN	12	49.8	-0.162151	0.49791	0.9991	0.0719	0.76252	0.87339	15	68.2	0.17079	0.47565	0.9973	0.04979	0.8341	0.862	NA
384	P21912	SDHB	Succinate dehyd	sp|P21912|SDHB_HUMAN	2	7.5	NA	NA	NA	NA	NA	NA	5	20	0.39686	0.14624	0.9991	-0.6787	0.1462	0.9991	NA
385	P21926	CD9	CD9 antigen	sp|P21926|CD9_HUMAN C	2	7	-0.186644	0.48993	0.9984	1.0051	0.48993	0.99836	NA	NA	NA	NA	NA	NA	NA	NA	NA
386	P22059	OSBP	Oxysterol-bindin	sp|P22059|OSBP1_HUMAN	1	1.6	NA	NA	NA	NA	NA	NA	2	3.2	-0.0574	0.86586	0.9991	0.12679	0.8659	0.9991	NA
387	P22087	FBL	rRNA 2-O-methy	sp|P22087|FBRL_HUMAN r	6	22.4	-0.033355	0.85712	0.9984	-0.1309	0.85712	0.99836	8	28.7	0.24182	NA	NA	NA	NA	NA	NA
388	P22102	GART	Trifunctional pur	sp|P22102|PUR2_HUMAN	14	18.3	0.339834	0.1356	0.9991	-0.7371	0.00353	0.03677	20	31.2	0.03299	0.88074	0.9973	0.53961	0.0238	0.0463	2
389	P22234	PAICS	Multifunctional	sp|P22234|PUR6_HUMAN	14	37.4	0.2653196	0.2207	0.9991	-0.3054	0.16165	0.33626	19	56	0.26875	0.21505	0.9973	0.61376	0.0093	0.0207	2
390	P22307	SCP2	Non-specific lipid	sp|P22307|NLTP_HUMAN N	2	3.7	0.263415	0.43855	0.9991	0.7487	0.06096	0.19558	7	13.9	0.19128	0.57196	0.9973	-0.0986	0.77	0.8136	NA
391	P22314	UBA1	Ubiquitin-like m	sp|P22314|UBA1_HUMAN	27	35.4	0.1366723	0.56631	0.9991	-0.8572	0.002	0.02835	33	45.1	-0.1131	0.6344	0.9973	0.23115	0.3366	0.4262	3
392	P22392	NME2	Nucleoside dipho	sp|P22392|NDKB_HUMAN	10	66.4	-0.212451	0.55616	0.9991	-1.1536	0.00477	0.04078	11	74.3	-0.4416	0.22927	0.9973	1.22224	0.0032	0.0084	2
393	P22626	HNRNPA2B1	Heterogeneous	sp|P22626|ROA2_HUMAN	18	40.2	0.0505452	0.84303	0.9991	0.1676	0.514	0.68373	24	60.1	0.04866	0.8488	0.9973	1.42401	######	0.0002	1
394	P22695	UQCRC2	Cytochrome b-c1	sp|P22695|QCR2_HUMAN	4	16.1	-0.103463	0.75696	0.9984	-0.0427	0.75696	0.99836	7	23.8	NA	NA	NA	NA	NA	NA	NA
395	P23193	TCEA1	Transcription elo	sp|P23193|TCEA1_HUMAN	5	17.6	-0.341828	0.25483	0.9991	-0.2385	0.47156	0.64919	8	27.6	0.22421	0.4496	0.9973	-0.148	0.6159	0.7032	NA
396	P23229	ITGA6	Integrin alpha-6	sp|P23229|ITA6_HUMAN I	5	6.1	0.3144048	NA	NA	NA	NA	NA	NA	NA	NA	NA	NA	NA	NA	NA	NA
397	P23246	SFPQ	Splicing factor, p	sp|P23246|SFPQ_HUMAN	21	30.1	0.275823	0.21961	0.9991	0.3922	0.08786	0.24124	21	30.1	0.25771	0.25002	0.9973	0.66569	0.007	0.0163	1
398	P23284	PPIB	Peptidyl-prolyl c	sp|P23284|PPIB_HUMAN P	11	47.2	0.1574964	0.53685	0.9991	0.2413	0.34787	0.5493	11	42.1	0.32281	0.21397	0.9973	0.30999	0.2319	0.3168	NA
399	P23378	GLDC	Glycine dehydro	sp|P23378|GCSP_HUMAN	6	6.9	-0.353072	0.08312	0.9984	0.3994	0.08312	0.99836	NA	NA	NA	NA	NA	NA	NA	NA	NA
400	P23381	WARS	Tryptophan--tRN	sp|P23381|SYWC_HUMAN	5	13.8	-0.248101	NA	NA	NA	NA	NA	9	31.2	NA	NA	NA	NA	NA	NA	NA
401	P23396	RPS3	40S ribosomal p	sp|P23396|RS3_HUMAN 40	15	63	0.1722741	0.47669	0.9991	-0.2443	0.31671	0.51612	16	67.9	0.12376	0.60781	0.9973	-0.8269	0.0029	0.008	4
402	P23434	GCSH	Glycine cleavage	sp|P23434|GCSH_HUMAN	2	17.3	-0.234839	0.46237	0.9984	0.2798	0.46237	0.99836	2	17.3	NA	NA	NA	NA	NA	NA	NA
403	P23526	AHCY	Adenosylhomocy	sp|P23526|SAHH_HUMAN	13	35.4	0.0168791	0.94796	0.9991	-0.97	0.00149	0.02711	15	38.9	-0.0442	0.8642	0.9973	2.43998	######	######	2
404	Q16778	HIST2H2BE	Histone H2B typ	sp|Q16778|H2B2E_HUMAN	11	60.3	-0.226468	0.499	0.9984	-0.0579	0.499	0.99836	11	60.3	NA	NA	NA	NA	NA	NA	NA
405	P23528	CFL1	Cofilin-1	sp|P23528|COF1_HUMAN	15	74.7	0.2230755	0.23336	0.9991	-0.6731	0.00175	0.02746	16	76.5	0.06418	0.72634	0.9973	0.99459	######	0.0002	2
406	P23588	EIF4B	Eukaryotic trans	sp|P23588|IF4B_HUMAN E	11	20.6	-0.119681	0.73798	0.9991	-0.4073	0.26352	0.46477	19	39.8	-0.3491	0.33533	0.9973	0.61502	0.0991	0.1576	NA
407	P23921	RRM1	Ribonucleoside-d	sp|P23921|RIR1_HUMAN R	7	9.8	-0.071	0.77766	0.9991	-0.7178	0.02058	0.09992	11	21.3	0.16551	0.5579	0.9973	-0.0326	0.8966	0.9121	3
408	P24534	EEF1B2	Elongation facto	sp|P24534|EF1B_HUMAN E	10	55.6	0.1511701	0.58153	0.9991	-0.8028	0.00857	0.06073	14	70.7	-0.0306	0.91076	0.9973	1.18405	0.0004	0.0016	2
409	P24539	ATP5PB	ATP synthase F(	sp|P24539|AT5F1_HUMAN	6	25.4	-0.209987	0.36381	0.9984	0.336	0.36381	0.99836	5	21.1	NA	NA	NA	NA	NA	NA	NA
410	P24666	ACP1	Low molecular w	sp|P24666|PPAC_HUMAN	4	27.2	0.0134979	0.96293	0.999	-0.5831	0.96293	0.99899	5	43	NA	NA	NA	NA	NA	NA	NA
411	P24928	POLR2A	DNA-directed RN	sp|P24928|RPB1_HUMAN D	6	4.6	-0.046226	0.84635	0.9991	0.1224	0.64744	0.78737	7	5.2	-0.0705	0.76767	0.9973	0.13683	0.6858	0.7593	NA
412	P25205	MCM3	DNA replication	sp|P25205|MCM3_HUMAN	26	42.9	-0.022211	0.91985	0.9991	0.2101	0.34775	0.5493	23	36.1	-0.1669	0.45347	0.9973	0.47026	0.0456	0.0801	1
413	P25398	RPS12	40S ribosomal p	sp|P25398|RS12_HUMAN 4	5	49.2	-0.238356	0.37612	0.9991	-0.912	0.003	0.0343	7	69.7	0.12285	0.6453	0.9973	0.57578	0.0427	0.0755	2
414	P25685	DNAJB1	DnaJ homolog su	sp|P25685|DNJB1_HUMAN	4	13.8	0.4024589	0.16457	0.9991	0.4583	0.23201	0.42949	8	27.6	0.05708	0.8774	0.9973	0.74789	0.0189	0.0383	1
415	P25705	ATP5F1A	ATP synthase su	sp|P25705|ATPA_HUMAN	22	40	-0.328265	0.11947	0.9991	0.1683	0.41179	0.60623	23	45.8	-0.0304	0.88082	0.9973	-0.7844	0.0012	0.0037	4
416	P25786	PSMA1	Proteasome sub	sp|P25786|PSA1_HUMAN	9	33.5	0.1423309	0.6438	0.9991	-0.0172	0.9553	0.96897	12	51.3	-0.1898	0.53858	0.9973	0.10282	0.738	0.7927	NA
417	P25787	PSMA2	Proteasome sub	sp|P25787|PSA2_HUMAN	3	17.9	NA	NA	NA	NA	NA	NA	8	53.4	0.09846	0.71234	0.9991	-0.4619	0.7123	0.9991	NA
418	P25788	PSMA3	Proteasome sub	sp|P25788|PSA3_HUMAN	9	33.3	0.2798898	0.1808	0.9991	0.2125	0.30397	0.50353	10	35.7	-0.1168	0.56758	0.9973	-0.5117	0.0209	0.0415	5
419	P25789	PSMA4	Proteasome sub	sp|P25789|PSA4_HUMAN	7	25.3	0.3490202	0.12106	0.9991	0.0069	0.97475	0.98167	10	41	-0.1606	0.46232	0.9973	-0.35	0.1201	0.1827	NA
420	P26038	MSN	Moesin	sp|P26038|MOES_HUMAN	25	47.3	-0.00143	0.9962	0.9991	-0.5063	0.10603	0.27536	23	36.6	-0.0345	0.90874	0.9973	-2.7566	######	######	4
421	P26196	DDX6	Probable ATP-de	sp|P26196|DDX6_HUMAN	8	21.1	0.1900111	0.46691	0.9991	-0.5023	0.09762	0.2602	10	27.1	-0.3092	0.2431	0.9973	-0.4955	0.0702	0.1186	NA
422	P26368	U2AF2	Splicing factor U	sp|P26368|U2AF2_HUMAN	7	16.8	-0.020256	0.91769	0.9991	0.2823	0.31706	0.51612	12	32.8	-0.0772	0.69443	0.9973	-0.4976	0.0213	0.042	5
423	P26373	RPL13	60S ribosomal p	sp|P26373|RL13_HUMAN 6	7	28.9	0.6496709	0.21789	0.9991	1.2061	0.02985	0.12709	8	36.5	-0.6254	0.23477	0.9973	0.36701	0.4793	0.5721	5
424	P26583	HMGB2	High mobility gr	sp|P26583|HMGB2_HUMA	5	27.3	0.3535729	0.20365	0.9991	0.1691	0.53525	0.70017	6	24.4	-0.0019	0.99436	0.9973	1.05194	0.0011	0.0036	1
425	P26599	PTBP1	Polypyrimidine t	sp|P26599|PTBP1_HUMAN	13	30.7	-0.136538	0.54965	0.9991	0.198	0.38855	0.59757	15	37.5	0.11657	0.60895	0.9973	-0.1097	0.6302	0.7149	NA
426	P26639	TARS	Threonine--tRNA	sp|P26639|SYTC_HUMAN T	13	21.6	-0.005482	0.98129	0.9991	-0.8359	0.00218	0.02915	20	33.7	-0.1351	0.56528	0.9973	1.28198	######	0.0002	2
427	P26640	VARS	Valine--tRNA lig	sp|P26640|SYVC_HUMAN	13	13.4	0.2228925	0.51149	0.9991	-0.1093	0.74624	0.86451	32	35.1	0.12602	0.70923	0.9973	3.88109	######	######	1
428	P26641	EEF1G	Elongation facto	sp|P26641|EF1G_HUMAN	14	27.2	0.0098523	0.96651	0.9991	-0.7569	0.00464	0.04065	22	56.8	-0.0207	0.9298	0.9973	0.51736	0.0394	0.0705	2

429	P27348	YWHAQ	14-3-3 protein th	sp|P27348|1433T_HUMAN	13	48.6	-0.06082	0.75538	0.9991	-0.6467	0.00381	0.03699	17	62.9	0.13338	0.49692	0.9973	0.96196	0.0001	0.0005	2
430	P27635	RPL10	60S ribosomal p	sp|P27635|RL10_HUMAN 6	2	18.7	NA	NA	NA	NA	NA	NA	5	30.4	-0.1484	0.6709	0.9991	-0.2076	0.6709	0.9991	NA
431	P27694	RPA1	Replication prot	sp|P27694|RFA1_HUMAN	7	12.3	0.0216409	0.90087	0.9991	-0.2052	0.30009	0.50005	8	15.3	0.00374	0.98281	0.9973	-1.9648	######	######	4
432	P27695	APEX1	DNA-(apurinic or	sp|P27695|APEX1_HUMAN	8	37.4	0.498416	0.05188	0.9991	-0.1259	0.60341	0.75321	12	57.5	-0.0491	0.83887	0.9973	0.99178	0.0007	0.0024	2
433	P27708	CAD	CAD protein	sp|P27708|PYR1_HUMAN C	17	10.2	0.2296947	0.31023	0.9991	-0.1579	0.48177	0.65688	36	21.6	0.08986	0.68734	0.9973	1.25916	######	0.0002	2
434	P27797	CALR	Calreticulin	sp|P27797|CALR_HUMAN C	27	65.5	-0.132277	0.53483	0.9991	0.3654	0.09863	0.26094	29	80.6	0.03361	0.87399	0.9973	-0.2675	0.2177	0.3032	NA
435	P27816	MAP4	Microtubule-ass	sp|P27816|MAP4_HUMAN	27	28.6	0.124013	0.64352	0.9984	0.2283	0.64352	0.99836	19	21.1	NA	NA	NA	NA	NA	NA	NA
436	P27824	CANX	Calnexin	sp|P27824|CALX_HUMAN C	21	29.6	0.0661227	0.75726	0.9991	0.6735	0.00544	0.04481	16	31.6	0.23159	0.28689	0.9973	1.44746	######	######	1
437	P28066	PSMA5	Proteasome sub	sp|P28066|PSA5_HUMAN	8	45.6	0.1053169	0.61362	0.9991	-0.0134	0.95385	0.96897	9	53.9	-0.1557	0.45751	0.9973	0.15779	0.4517	0.5449	NA
438	P28070	PSMB4	Proteasome sub	sp|P28070|PSB4_HUMAN P	5	38.3	-0.271071	0.36234	0.9991	-0.2142	0.51676	0.68611	7	45.5	-0.408	0.17757	0.9973	-1.3842	0.0002	0.0009	4
439	P28072	PSMB6	Proteasome sub	sp|P28072|PSB6_HUMAN P	3	12.6	0.4385315	0.03875	0.9984	-0.3081	0.03875	0.99836	3	12.6	NA	NA	NA	NA	NA	NA	NA
440	P28074	PSMB5	Proteasome sub	sp|P28074|PSB5_HUMAN P	4	20.9	0.1602595	0.5614	0.9991	-0.2234	0.47039	0.64919	7	35.7	-0.1512	0.62349	0.9973	1.79536	######	######	2
441	P28331	NDUFS1	NADH-ubiquinon	sp|P28331|NDUS1_HUMAN	4	8.4	0.004678	0.99036	0.999	0.9032	0.99036	0.99899	7	13.3	-0.0656	NA	NA	NA	NA	NA	NA
442	P28340	POLD1	DNA polymerase	sp|P28340|DPOD1_HUMAN	2	2.4	-1.084879	0.0162	0.9984	-0.7863	0.0162	0.99836	3	3.6	NA	NA	NA	NA	NA	NA	NA
443	P28482	MAPK1	Mitogen-activat	sp|P28482|MK01_HUMAN	3	10.8	0.6305585	0.03398	0.9984	0.0254	0.03398	0.99836	6	20.8	-0.2591	NA	NA	NA	NA	NA	NA
444	P28838	LAP3	Cytosol aminope	sp|P28838|AMPL_HUMAN	6	14.6	0.2431241	0.35534	0.9991	0.1347	0.71371	0.84477	17	42.2	0.46816	0.08667	0.9973	0.83315	0.0054	0.0133	1
445	P29144	TPP2	Tripeptidyl-pept	sp|P29144|TPP2_HUMAN T	8	9	0.2557984	0.2632	0.9984	-0.3757	0.2632	0.99836	14	15.9	NA	NA	NA	NA	NA	NA	NA
446	P29218	IMPA1	Inositol monoph	sp|P29218|IMPA1_HUMAN	3	13	NA	NA	NA	NA	NA	NA	3	14.8	-0.0332	NA	NA	NA	NA	NA	NA
447	P29373	CRABP2	Cellular retinoic	sp|P29373|RABP2_HUMAN	3	23.2	0.2336716	NA	NA	NA	NA	NA	8	59.4	NA	NA	NA	NA	NA	NA	NA
448	P29401	TKT	Transketolase	sp|P29401|TKT_HUMAN Tr	23	43.8	-0.08993	0.64643	0.9991	-0.6576	0.00344	0.03677	22	44.3	-0.0039	0.98397	0.9973	-0.8007	0.0007	0.0024	3
449	P29692	EEF1D	Elongation facto	sp|P29692|EF1D_HUMAN	10	43.4	-0.006165	0.98264	0.9991	-0.3567	0.21896	0.4107	20	69.8	0.02596	0.92702	0.9973	1.60355	######	0.0002	2
450	P29762	CRABP1	Cellular retinoic	sp|P29762|RABP1_HUMAN	8	65.7	-0.078917	0.73071	0.9984	-0.0455	0.73071	0.99836	8	59.1	NA	NA	NA	NA	NA	NA	NA
451	P29966	MARCKS	Myristoylated a	sp|P29966|MARCS_HUMA	13	69.9	0.2952004	0.31103	0.9984	0.0467	0.31103	0.99836	12	55.4	NA	NA	NA	NA	NA	NA	NA
452	P30040	ERP29	Endoplasmic ret	sp|P30040|ERP29_HUMAN	8	36.4	0.0073549	0.98359	0.9991	0.4729	0.1978	0.38005	10	52.9	-0.2896	0.42282	0.9973	1.95093	######	0.0002	1
453	P30041	PRDX6	Peroxiredoxin-6	sp|P30041|PRDX6_HUMAN	10	53.6	-0.172695	0.53785	0.9991	-0.7424	0.01538	0.08199	17	78.6	-0.4708	0.10514	0.9973	3.95924	######	######	2
454	P30044	PRDX5	Peroxiredoxin-5,	sp|P30044|PRDX5_HUMAN	4	23.8	0.3311664	0.30666	0.9991	-0.4119	0.20745	0.39404	7	47.7	0.03328	0.91685	0.9973	0.55276	0.0969	0.1553	NA
455	P30048	PRDX3	Thioredoxin-dep	sp|P30048|PRDX3_HUMAN	6	25.4	0.1584562	0.64583	0.9991	0.7804	0.03707	0.14131	10	49.2	-0.6318	0.23289	0.9973	3.67403	######	######	1
456	P30050	RPL12	60S ribosomal p	sp|P30050|RL12_HUMAN 6	9	63.6	0.1040017	0.80468	0.9991	-1.0506	0.02162	0.10155	8	58.8	0.27045	0.52247	0.9973	0.66313	0.1282	0.1938	2
457	P30084	ECHS1	Enoyl-CoA hydra	sp|P30084|ECHM_HUMAN	8	37.9	0.1647428	0.57336	0.9984	0.4213	0.57336	0.99836	7	34.5	NA	NA	NA	NA	NA	NA	NA
458	P30085	CMPK1	UMP-CMP kinas	sp|P30085|KCY_HUMAN U	2	13.8	NA	NA	NA	NA	NA	NA	9	47.4	-0.0199	0.95306	0.9991	0.77979	0.9531	0.9991	NA
459	P30086	PEBP1	Phosphatidyleth	sp|P30086|PEBP1_HUMAN	9	66.8	-0.274013	0.56111	0.9991	-1.3675	0.00903	0.06244	10	73.8	-0.3874	0.41373	0.9973	2.84314	######	######	2
460	P30101	PDIA3	Protein disulfide	sp|P30101|PDIA3_HUMAN	22	49.5	-0.099726	0.52716	0.9991	0.2075	0.19712	0.37977	27	55.6	0.10442	0.50815	0.9973	0.67792	0.0004	0.0016	1
461	P30153	PPP2R1A	Serine/threonine	sp|P30153|2AAA_HUMAN	13	26.8	0.0752601	0.71439	0.9991	-0.5681	0.01236	0.07182	20	44	-0.1224	0.55317	0.9973	-0.3694	0.0859	0.1419	3
462	P30154	PPP2R1B	Serine/threonine	sp|P30154|2AAB_HUMAN	2	3.5	NA	NA	NA	NA	NA	NA	8	19.5	0.2284	NA	NA	NA	NA	NA	NA
463	P30419	NMT1	Glycylpeptide N-	sp|P30419|NMT1_HUMAN	5	13.5	-0.066085	0.8343	0.9991	-0.2545	0.57088	0.72797	6	16.3	-0.1367	0.66601	0.9973	-1.6482	0.0001	0.0005	4
464	P30519	HMOX2	Heme oxygenase	sp|P30519|HMOX2_HUMA	2	7.9	NA	NA	NA	NA	NA	NA	7	31	0.16234	NA	NA	NA	NA	NA	NA
465	P30520	ADSS	Adenylosuccinat	sp|P30520|PURA2_HUMAN	4	14.9	-0.117376	0.72485	0.9991	-0.6945	0.13624	0.30186	11	32	0.08054	0.78693	0.9973	-0.2504	0.4064	0.5011	NA
466	P30533	LRPAP1	Alpha-2-macrogl	sp|P30533|AMRP_HUMAN	4	15.1	-0.143374	0.72852	0.9984	0.3899	0.72852	0.99836	5	19	NA	NA	NA	NA	NA	NA	NA
467	P30566	ADSL	Adenylosuccinat	sp|P30566|PUR8_HUMAN A	6	13.4	0.1990802	0.68119	0.9984	-0.4054	0.68119	0.99836	11	32.4	NA	NA	NA	NA	NA	NA	NA
468	P30837	ALDH1B1	Aldehyde dehyd	sp|P30837|AL1B1_HUMAN	3	8.5	-0.148359	0.60909	0.9984	0.2675	0.60909	0.99836	3	8.9	NA	NA	NA	NA	NA	NA	NA
469	P30876	POLR2B	DNA-directed RN	sp|P30876|RPB2_HUMAN D	6	6	-0.100053	0.70786	0.9991	0.0299	0.92922	0.9536	9	10.7	0.22545	0.35195	0.9973	-0.0808	0.735	0.7919	NA
470	P31040	SDHA	Succinate dehyd	sp|P31040|SDHA_HUMAN	1	1.8	NA	NA	NA	NA	NA	NA	7	13.7	-0.1732	NA	NA	NA	NA	NA	NA
471	P31153	MAT2A	S-adenosylmethi	sp|P31153|METK2_HUMAN	10	33.2	0.2092938	0.49692	0.9991	-0.8265	0.0142	0.07895	11	33.2	-0.0941	0.75871	0.9973	-0.5567	0.0828	0.1374	3
472	P31689	DNAJA1	DnaJ homolog su	sp|P31689|DNJA1_HUMAN	8	22.4	0.1701934	0.33263	0.9991	-0.1692	0.38748	0.59722	12	44.3	-0.0464	0.78863	0.9973	0.42046	0.0256	0.0491	2
473	P31930	UQCRC1	Cytochrome b-c1	sp|P31930|QCR1_HUMAN C	4	13.1	-0.217304	0.38628	0.9991	-0.0493	0.8423	0.91735	7	21.2	0.23425	0.29926	0.9973	-0.0827	0.7094	0.7775	NA
474	P31939	ATIC	Bifunctional puri	sp|P31939|PUR9_HUMAN	17	45.4	0.0245886	0.90856	0.9991	-0.7129	0.0083	0.05941	31	75	-0.1989	0.35958	0.9973	0.75384	0.0026	0.0073	2
475	P31942	HNRNPH3	Heterogeneous	sp|P31942|HNRH3_HUMAN	7	27.7	-0.033206	0.92125	0.9991	0.3657	0.28484	0.48465	8	35.3	0.4355	0.20611	0.9973	1.18718	0.0024	0.0067	1
476	P31943	HNRNPH1	Heterogeneous	sp|P31943|HNRH1_HUMAN	11	31.6	-0.074058	0.69196	0.9991	0.0848	0.65044	0.78966	14	48.1	-0.0479	0.79766	0.9973	-0.0438	0.8145	0.848	NA

477	P31946	YWHAB	14-3-3 protein b	sp|P31946|1433B_HUMAN	15	64.6	0.1047426	0.59558	0.9991	-0.4996	0.01981	0.09821	16	65.4	0.08587	0.663	0.9973	1.33532	######	######	2
478	P31948	STIP1	Stress-induced-p	sp|P31948|STIP1_HUMAN	28	49.9	0.1142391	0.54784	0.9991	-0.728	0.0012	0.02575	36	62.2	0.04993	0.79187	0.9973	0.29121	0.1369	0.2047	2
479	P31949	S100A11	Protein S100-A1	sp|P31949|S10AB_HUMAN	4	41	-0.024136	0.90813	0.9987	-0.6594	0.90813	0.99874	7	80	NA	NA	NA	2.91063	NA	NA	NA
480	P32119	PRDX2	Peroxiredoxin-2	sp|P32119|PRDX2_HUMAN	8	52.5	0.0778716	0.78714	0.9991	-0.6686	0.0312	0.12742	12	53.5	0.34147	0.24585	0.9973	1.84232	######	######	2
481	P32969	RPL9	60S ribosomal p	sp|P32969|RL9_HUMAN 60	4	36.5	0.1022963	0.74537	0.9991	0.2283	0.47149	0.64919	8	57.8	-0.0792	0.80137	0.9973	-0.7633	0.0251	0.0486	4
482	P33316	DUT	Deoxyuridine 5-t	sp|P33316|DUT_HUMAN D	4	23.8	0.1217801	0.82134	0.9991	-0.2805	0.64288	0.78451	9	46	0.63197	0.43991	0.9973	3.05261	0.0002	0.0009	2
483	P33981	TTK	Dual specificity p	sp|P33981|TTK_HUMAN D	3	5.6	0.0950067	NA	NA	NA	NA	NA	NA	NA	NA	NA	NA	NA	NA	NA	NA
484	P33992	MCM5	DNA replication	sp|P33992|MCM5_HUMAN	14	25.1	-0.008443	0.98102	0.9991	-0.7835	0.03915	0.1469	13	24.9	0.01215	0.97268	0.9973	-0.277	0.4394	0.5316	3
485	P33993	MCM7	DNA replication	sp|P33993|MCM7_HUMAN	16	23.4	0.4738892	0.19398	0.9991	-0.0242	0.9458	0.96624	17	28.9	0.48699	0.18256	0.9973	0.90086	0.0201	0.0404	1
486	P34896	SHMT1	Serine hydroxym	sp|P34896|GLYC_HUMAN S	2	6.6	NA	NA	NA	NA	NA	NA	5	16.1	0.25769	NA	NA	NA	NA	NA	NA
487	P34897	SHMT2	Serine hydroxym	sp|P34897|GLYM_HUMAN	11	27.8	0.0643378	0.7882	0.9991	0.9452	0.00097	0.02286	17	43.8	-0.2755	0.25899	0.9973	-0.281	0.25	0.3338	5
488	P34932	HSPA4	Heat shock 70 k	sp|P34932|HSP74_HUMAN	38	55.1	0.0564983	0.72211	0.9991	-0.3864	0.02463	0.11264	39	59.5	0.1758	0.27648	0.9973	0.16647	0.3019	0.3875	2
489	P35221	CTNNA1	Catenin alpha-1	sp|P35221|CTNA1_HUMAN	17	27.4	0.1085227	0.5535	0.9984	0.3895	0.5535	0.99836	7	12.3	0.09126	NA	NA	NA	NA	NA	NA
490	P35222	CTNNB1	Catenin beta-1	sp|P35222|CTNB1_HUMAN	7	12.2	0.3502156	0.14772	0.9984	0.7471	0.14772	0.99836	3	4.5	-0.1424	NA	NA	NA	NA	NA	NA
491	P35232	PHB	Prohibitin	sp|P35232|PHB_HUMAN P	12	55.9	0.123977	0.542	0.9991	0.7715	0.00129	0.02619	13	63.2	0.15134	0.45792	0.9973	0.73777	0.0019	0.0054	1
492	P35241	RDX	Radixin	sp|P35241|RADI_HUMAN R	14	23	-0.204473	0.26086	0.9984	-0.0647	0.26086	0.99836	17	30.4	0.18848	NA	NA	NA	NA	NA	NA
493	P35268	RPL22	60S ribosomal p	sp|P35268|RL22_HUMAN 6	3	30.5	0.4634449	0.10156	0.9991	0.1674	0.5396	0.70193	4	50.8	0.25766	0.34882	0.9973	-0.6596	0.0249	0.0482	5
494	P35579	MYH9	Myosin-9	sp|P35579|MYH9_HUMAN	71	42.4	0.0634854	0.67582	0.9991	-0.4588	0.00707	0.05274	80	45.8	0.05025	0.74042	0.9973	-1.1217	######	######	4
495	P35580	MYH10	Myosin-10	sp|P35580|MYH10_HUMA	69	40.4	0.1804269	0.26287	0.9991	-0.1008	0.52591	0.69224	41	26.5	0.18922	0.24121	0.9973	-0.0864	0.5864	0.676	NA
496	P35606	COPB2	Coatomer subun	sp|P35606|COPB2_HUMAN	10	16.9	0.1431392	0.43875	0.9991	-0.1982	0.28778	0.48813	15	23.3	-0.0111	0.95159	0.9973	-1.4918	######	######	4
497	P35611	ADD1	Alpha-adducin	sp|P35611|ADDA_HUMAN	5	9.5	0.3400206	0.09585	0.9984	-0.0106	0.09585	0.99836	NA	NA	NA	NA	NA	NA	NA	NA	NA
498	P35612	ADD2	Beta-adducin	sp|P35612|ADDB_HUMAN	2	3.7	0.6489368	0.33906	0.9984	-0.203	0.33906	0.99836	NA	NA	NA	NA	NA	NA	NA	NA	NA
499	P35613	BSG	Basigin	sp|P35613|BASI_HUMAN B	7	21.8	-0.078333	0.8105	0.9984	0.6329	0.8105	0.99836	4	14.5	NA	NA	NA	NA	NA	NA	NA
500	P35637	FUS	RNA-binding pro	sp|P35637|FUS_HUMAN R	4	8.9	0.039121	0.92318	0.9991	0.1157	0.77582	0.8815	7	17.7	0.22301	0.58423	0.9973	1.21843	0.0075	0.0171	1
501	P35658	NUP214	Nuclear pore co	sp|P35658|NU214_HUMAN	5	2.8	-0.04766	0.90744	0.9987	0.6432	0.90744	0.99874	3	2.2	NA	NA	NA	NA	NA	NA	NA
502	P35659	DEK	Protein DEK	sp|P35659|DEK_HUMAN P	6	18.1	0.1763908	0.62409	0.9991	0.4192	0.25217	0.44979	7	20	-0.3413	0.34794	0.9973	1.42789	0.0009	0.003	1
503	P35998	PSMC2	26S proteasome	sp|P35998|PRS7_HUMAN 2	15	43.6	0.1384189	0.53095	0.9991	0.1798	0.4176	0.61048	18	53.1	0.17588	0.42768	0.9973	-0.2584	0.2491	0.3333	NA
504	P36405	ARL3	ADP-ribosylation	sp|P36405|ARL3_HUMAN A	2	16.5	0.8346962	NA	NA	NA	NA	NA	5	37.9	NA	NA	NA	-1.335	NA	NA	NA
505	P36542	ATP5F1C	ATP synthase su	sp|P36542|ATPG_HUMAN	7	23.2	0.1510699	0.51923	0.9984	0.7145	0.51923	0.99836	10	35.6	-0.2557	NA	NA	NA	NA	NA	NA
506	Q96A05	ATP6V1E2	V-type proton A	sp|Q96A05|VATE2_HUMA	2	9.7	NA	NA	NA	NA	NA	NA	5	23	-0.3579	0.23738	0.9991	0.66514	0.2374	0.9991	NA
507	P36578	RPL4	60S ribosomal p	sp|P36578|RL4_HUMAN 60	6	12.4	0.4631671	0.37292	0.9991	0.3509	0.49743	0.67563	13	35.1	-0.6367	0.22561	0.9973	1.44806	0.0111	0.0241	1
508	P36776	LONP1	Lon protease ho	sp|P36776|LONM_HUMAN	7	8.7	-0.150243	0.4859	0.9991	0.1829	0.39813	0.60515	14	20.1	-0.0118	0.96073	0.9973	0.0744	0.756	0.8049	NA
509	P36871	PGM1	Phosphoglucomu	sp|P36871|PGM1_HUMAN	5	11.4	-0.10419	0.66767	0.9984	-0.5727	0.66767	0.99836	11	25.3	0.15549	NA	NA	NA	NA	NA	NA
510	P36873	PPP1CC	Serine/threonin	sp|P36873|PP1G_HUMAN S	4	15.5	-0.07845	0.83513	0.9984	-0.1839	0.83513	0.99836	12	41.2	NA	NA	NA	NA	NA	NA	NA
511	P36957	DLST	Dihydrolipoyllys	sp|P36957|ODO2_HUMAN	8	19.2	-0.300144	0.22925	0.9991	0.153	0.5328	0.69826	6	16.6	0.02428	0.92899	0.9973	-0.1062	0.6641	0.7426	NA
512	P37108	SRP14	Signal recogniti	sp|P37108|SRP14_HUMAN	5	32.4	0.0620033	0.78133	0.9984	0.2763	0.78133	0.99836	5	33.1	NA	NA	NA	NA	NA	NA	NA
513	P37198	NUP62	Nuclear pore gly	sp|P37198|NUP62_HUMAN	3	7.9	-0.073598	0.72231	0.9984	0.5436	0.72231	0.99836	6	17	-0.1282	NA	NA	NA	NA	NA	NA
514	P37268	FDFT1	Squalene syntha	sp|P37268|FDFT_HUMAN S	7	22.5	-0.308129	0.29907	0.9984	0.0572	0.29907	0.99836	3	10.1	NA	NA	NA	NA	NA	NA	NA
515	P37802	TAGLN2	Transgelin-2	sp|P37802|TAGL2_HUMAN	1	5.5	NA	NA	NA	NA	NA	NA	13	78.4	-0.2071	0.45015	0.9991	-0.3846	0.4501	0.9991	NA
516	P37837	TALDO1	Transaldolase	sp|P37837|TALDO_HUMAN	9	26.7	-0.157189	0.56	0.9991	-0.6689	0.02194	0.10166	12	35	-0.0389	0.88462	0.9973	1.91835	######	######	2
517	P38117	ETFB	Electron transfe	sp|P38117|ETFB_HUMAN E	6	25.5	0.1970262	0.56133	0.9984	1.1393	0.56133	0.99836	9	38	NA	NA	NA	NA	NA	NA	NA
518	P38159	RBMX	RNA-binding mo	sp|P38159|RBMX_HUMAN	12	31.7	0.3731403	0.08292	0.9991	0.7748	0.00141	0.02711	9	22.8	-0.3308	0.12066	0.9973	1.33385	######	######	1
519	P38606	ATP6V1A	V-type proton A	sp|P38606|VATA_HUMAN	2	4.4	NA	NA	NA	NA	NA	NA	12	28	0.25989	0.65579	0.9991	2.9545	0.6558	0.9991	NA
520	P38646	HSPA9	Stress-70 protei	sp|P38646|GRP75_HUMAN	35	56.7	-0.133774	0.47259	0.9991	0.3419	0.07813	0.22519	43	65.4	0.00705	0.96955	0.9973	0.20378	0.2789	0.3641	NA
521	P38919	EIF4A3	Eukaryotic initia	sp|P38919|IF4A3_HUMAN	10	22.6	0.0708638	0.74817	0.9991	0.5056	0.03294	0.12975	12	35	0.05401	0.80655	0.9973	-0.4019	0.0822	0.1368	5
522	P39019	RPS19	40S ribosomal p	sp|P39019|RS19_HUMAN 4	8	49.7	-0.270232	0.43628	0.9991	-0.2379	0.58573	0.74361	10	55.2	0.02282	0.94072	0.9973	1.23647	0.0012	0.0037	2
523	P39023	RPL3	60S ribosomal p	sp|P39023|RL3_HUMAN 60	13	38.7	-0.222155	0.41802	0.9991	0.0541	0.84222	0.91735	15	39.5	-0.3088	0.26469	0.9973	-0.7639	0.0112	0.0244	4
524	P39656	DDOST	Dolichyl-diphosp	sp|P39656|OST48_HUMAN	4	12.7	NA	NA	NA	NA	NA	NA	6	16.4	-0.0244	0.89679	0.9991	-0.1892	0.8968	0.9991	NA

525	P39687	ANP32A	Acidic leucine-ric	sp|P39687|AN32A_HUMAN	7	24.5	0.0329531	0.83915	0.9984	-0.4738	0.83915	0.99836	10	48.2	NA	NA	NA	NA	NA	NA	NA
526	P39748	FEN1	Flap endonuclea	sp|P39748|FEN1_HUMAN	5	18.7	0.4393224	0.21094	0.9991	0.1333	0.69756	0.83261	11	39.5	0.91877	0.02714	0.9973	1.05707	0.0066	0.0154	1
527	P40121	CAPG	Macrophage-cap	sp|P40121|CAPG_HUMAN	2	6.9	-0.050528	0.89362	0.9984	-0.325	0.89362	0.99836	3	11.5	0.15976	NA	NA	NA	NA	NA	NA
528	P40222	TXLNA	Alpha-taxilin	sp|P40222|TXLNA_HUMAN	5	8.6	0.0523124	0.86644	0.9991	0.0506	0.87085	0.92292	10	19	0.44986	0.16074	0.9973	1.08926	0.0025	0.0071	1
529	P40227	CCT6A	T-complex prote	sp|P40227|TCPZ_HUMAN T	16	40.9	-0.036769	0.8455	0.9991	-0.6104	0.00454	0.04022	20	49.2	0.0044	0.9814	0.9973	-0.565	0.0076	0.0173	3
530	P40429	RPL13A	60S ribosomal p	sp|P40429|RL13A_HUMAN	4	20.7	0.1120594	0.79	0.9991	0.4367	0.30676	0.50462	6	30	0.29026	0.49302	0.9973	-0.1563	0.7106	0.7775	NA
531	P40616	ARL1	ADP-ribosylation	sp|P40616|ARL1_HUMAN A	2	13.8	-0.498793	0.14865	0.9991	0.0529	0.89594	0.93506	3	22.7	-0.193	0.41515	0.9973	-0.5283	0.0391	0.0705	4
532	P40763	STAT3	Signal transduce	sp|P40763|STAT3_HUMAN	3	4.8	0.5318916	NA	NA	NA	NA	NA	2	3.6	-0.5436	NA	NA	NA	NA	NA	NA
533	P40925	MDH1	Malate dehydro	sp|P40925|MDHC_HUMAN	11	34.7	0.0556784	0.7793	0.9991	-0.7607	0.00125	0.02602	11	33.8	0.03721	0.85133	0.9973	0.18771	0.3508	0.4418	3
534	P40926	MDH2	Malate dehydro	sp|P40926|MDHM_HUMA	16	55.3	-0.068681	0.78188	0.9991	0.4649	0.07447	0.22092	17	63.6	0.35389	0.16589	0.9973	1.90745	######	######	1
535	P40939	HADHA	Trifunctional enz	sp|P40939|ECHA_HUMAN	8	12.2	0.0827614	0.8182	0.9991	0.4358	0.23675	0.43447	21	41.5	-0.0752	0.85177	0.9973	4.62546	######	######	1
536	P41091	EIF2S3	Eukaryotic trans	sp|P41091|IF2G_HUMAN E	8	23.5	-0.411396	0.15124	0.9991	-0.9594	0.00645	0.04968	16	48.1	-0.0096	0.97243	0.9973	1.26986	0.0003	0.0011	2
537	P41227	NAA10	N-alpha-acetyltr	sp|P41227|NAA10_HUMAN	3	15.3	-0.148443	0.68806	0.9991	-0.2138	0.5983	0.75079	8	44.7	-0.655	0.06229	0.9973	0.31665	0.3447	0.4356	NA
538	P41250	GARS	Glycine--tRNA lig	sp|P41250|GARS_HUMAN	5	7.4	0.3599101	0.13491	0.9991	-0.2762	0.24455	0.44232	17	28.8	-0.1109	0.63451	0.9973	1.4875	######	######	2
539	P41252	IARS	Isoleucine--tRNA	sp|P41252|SYIC_HUMAN Is	17	18.2	0.1871652	0.44779	0.9991	-0.2038	0.40925	0.60515	15	14.2	-0.0923	0.70612	0.9973	-0.1581	0.5202	0.6112	NA
540	P41567	EIF1	Eukaryotic trans	sp|P41567|EIF1_HUMAN E	4	63.7	0.4293899	NA	NA	NA	NA	NA	7	75.2	0.21359	0.49714	0.9991	0.45485	0.4971	0.9991	NA
541	P41743	PRKCI	Protein kinase C	sp|P41743|KPCI_HUMAN P	2	4.9	NA	NA	NA	NA	NA	NA	2	4.9	-0.147	NA	NA	NA	NA	NA	NA
542	P42126	ECI1	Enoyl-CoA delta	sp|P42126|ECI1_HUMAN E	3	12.6	-0.315494	0.39676	0.9984	0.025	0.39676	0.99836	1	5	NA	NA	NA	NA	NA	NA	NA
543	P42166	TMPO	Lamina-associate	sp|P42166|LAP2A_HUMAN	6	13.1	-0.088173	0.69945	0.9991	0.2222	0.33724	0.5361	14	33.7	-0.112	0.68879	0.9973	0.53802	0.0491	0.0857	1
544	P42167	TMPO	Lamina-associate	sp|P42167|LAP2B_HUMAN	5	15.4	0.0895494	0.87336	0.9991	0.566	0.32427	0.52252	12	40.3	-0.0508	0.89214	0.9973	0.91513	0.029	0.0547	1
545	P42224	STAT1	Signal transduce	sp|P42224|STAT1_HUMAN	9	15.1	0.1740597	0.44674	0.9984	0.5596	0.44674	0.99836	5	8.1	NA	NA	NA	NA	NA	NA	NA
546	P42285	MTREX	Exosome RNA he	sp|P42285|MTREX_HUMAN	8	10	-0.200047	0.5433	0.9984	-0.2598	0.5433	0.99836	10	13.5	NA	NA	NA	NA	NA	NA	NA
547	P42677	RPS27	40S ribosomal p	sp|P42677|RS27_HUMAN 4	2	28.6	0.3692037	0.23274	0.9991	0.351	0.25558	0.45188	2	28.6	0.16467	0.58749	0.9973	-0.0139	0.9672	0.9726	NA
548	P42704	LRPPRC	Leucine-rich PPR	sp|P42704|LPPRC_HUMAN	32	27.2	-0.010855	0.9651	0.9991	0.3726	0.14642	0.31651	49	43.6	-0.094	0.70539	0.9973	4.48489	######	######	1
549	P42765	ACAA2	3-ketoacyl-CoA t	sp|P42765|THIM_HUMAN	7	26.7	-0.015889	0.95524	0.999	0.7456	0.95524	0.99899	9	40.8	NA	NA	NA	NA	NA	NA	NA
550	P42766	RPL35	60S ribosomal p	sp|P42766|RL35_HUMAN 6	2	14.6	-0.423866	0.20057	0.9984	-0.5081	0.20057	0.99836	2	14.6	-1.1581	NA	NA	NA	NA	NA	NA
551	P43034	PAFAH1B1	Platelet-activati	sp|P43034|LIS1_HUMAN P	7	19.5	0.1184491	0.58646	0.9991	-0.5544	0.0198	0.09821	7	21.7	0.05202	0.81044	0.9973	-0.7011	0.0099	0.0218	3
552	P43243	MATR3	Matrin-3	sp|P43243|MATR3_HUMA	20	31.5	0.1357172	0.41372	0.9991	0.7016	0.00049	0.0144	19	34.9	-0.2512	0.13964	0.9973	2.29458	######	######	1
553	P43246	MSH2	DNA mismatch r	sp|P43246|MSH2_HUMAN	13	15.6	0.1385051	0.50495	0.9984	-0.0332	0.50495	0.99836	11	13.2	NA	NA	NA	NA	NA	NA	NA
554	P43304	GPD2	Glycerol-3-phosp	sp|P43304|GPDM_HUMAN	3	4.5	NA	NA	NA	NA	NA	NA	10	16.2	0.1262	0.52077	0.9991	0.036	0.5208	0.9991	NA
555	P43307	SSR1	Translocon-asso	sp|P43307|SSRA_HUMAN T	2	6.6	-0.421826	0.27759	0.9991	0.4241	0.31739	0.51612	4	21	-0.3293	0.34088	0.9973	-0.3662	0.2914	0.3763	NA
556	P43487	RANBP1	Ran-specific GTP	sp|P43487|RANG_HUMAN	4	31.3	-0.016266	0.96455	0.9991	-0.5548	0.14293	0.31085	6	47.3	0.01156	0.97481	0.9973	0.81535	0.0377	0.0684	2
557	P43490	NAMPT	Nicotinamide ph	sp|P43490|NAMPT_HUMA	8	21.6	-0.254617	0.25376	0.9991	-0.8671	0.01415	0.07895	9	24.4	0.44316	0.18972	0.9973	-0.327	0.1939	0.2777	3
558	P43686	PSMC4	26S proteasome	sp|P43686|PRS6B_HUMAN	9	29.9	0.0882632	0.69969	0.9991	0.0878	0.70104	0.83535	13	45.2	0.11331	0.62099	0.9973	-0.4237	0.0774	0.1291	NA
559	P45880	VDAC2	Voltage-depende	sp|P45880|VDAC2_HUMAN	12	52	-0.048403	0.82737	0.9991	0.1848	0.40969	0.60515	13	56.1	0.19232	0.39136	0.9973	0.6626	0.0078	0.0175	1
560	P45973	CBX5	Chromobox prot	sp|P45973|CBX5_HUMAN C	10	56	0.1999885	0.56701	0.9991	0.5016	0.16173	0.33626	10	56	0.35958	0.30875	0.9973	1.73011	0.0001	0.0005	1
561	P45974	USP5	Ubiquitin carbox	sp|P45974|UBP5_HUMAN	12	18.3	0.2095625	0.24016	0.9991	-0.5487	0.00555	0.04524	20	30.5	-0.0965	0.58207	0.9973	0.6271	0.0021	0.006	2
562	P46013	MKI67	Proliferation ma	sp|P46013|KI67_HUMAN P	13	7.6	0.2896625	0.28508	0.9984	0.974	0.28508	0.99836	17	8	NA	NA	NA	NA	NA	NA	NA
563	P46060	RANGAP1	Ran GTPase-acti	sp|P46060|RAGP1_HUMAN	6	11.2	-0.33586	0.1966	0.9991	-0.1818	0.47597	0.65273	16	29.5	0.05659	0.84146	0.9973	0.6856	0.0259	0.0496	2
564	P46087	NOP2	Probable 28S rR	sp|P46087|NOP2_HUMAN	5	8.5	NA	NA	NA	NA	NA	NA	11	16.4	-0.2954	0.62526	0.9991	1.7105	0.6253	0.9991	NA
565	P46108	CRK	Adapter molecul	sp|P46108|CRK_HUMAN A	2	8.9	NA	NA	NA	NA	NA	NA	5	25.7	-0.0266	NA	NA	NA	NA	NA	NA
566	P46109	CRKL	Crk-like protein	sp|P46109|CRKL_HUMAN C	4	18.5	-0.146225	NA	NA	NA	NA	NA	7	35.3	0.34057	NA	NA	NA	NA	NA	NA
567	P46776	RPL27A	60S ribosomal p	sp|P46776|RL27A_HUMAN	1	7.4	NA	NA	NA	NA	NA	NA	4	27.7	-0.7485	0.11391	0.9991	2.16563	0.1139	0.9991	NA
568	P46777	RPL5	60S ribosomal p	sp|P46777|RL5_HUMAN 60	11	36.4	0.0116307	0.96247	0.9991	-0.269	0.28505	0.48465	12	40.1	0.02241	0.92776	0.9973	0.00665	0.9785	0.9827	NA
569	P46778	RPL21	60S ribosomal p	sp|P46778|RL21_HUMAN 6	5	29.4	-0.59914	0.26267	0.9991	-0.4651	0.43183	0.6164	4	25	-0.5241	0.32482	0.9973	-1.2865	0.0244	0.0473	4
570	P46779	RPL28	60S ribosomal p	sp|P46779|RL28_HUMAN 6	4	28.5	-0.554309	0.48138	0.9991	0.3286	0.6455	0.78636	4	28.5	-0.4445	0.62302	0.9973	-0.3558	0.5784	0.669	NA
571	P46781	RPS9	40S ribosomal p	sp|P46781|RS9_HUMAN 40	7	30.4	-0.185604	0.46228	0.9991	0.0504	0.84042	0.91735	11	42.3	-0.1327	0.59776	0.9973	0.75112	0.0075	0.0172	1
572	P46782	RPS5	40S ribosomal p	sp|P46782|RS5_HUMAN 40	4	29.9	-0.158909	0.59008	0.9991	-0.0745	0.80002	0.89749	9	49	0.02609	0.92921	0.9973	0.36684	0.2224	0.3061	NA

573	P46783	RPS10	40S ribosomal p	sp|P46783|RS10_HUMAN 4	5	27.9	0.2101839	0.52486	0.9991	0.0553	0.86628	0.92126	8	43	0.47378	0.16197	0.9973	-0.9922	0.0072	0.0167	4
574	P46821	MAP1B	Microtubule-ass	sp|P46821|MAP1B_HUMA	25	16.6	0.1499327	0.61633	0.9991	0.5	0.10743	0.27697	80	47.7	0.07674	0.79701	0.9973	5.28338	######	######	1
575	P46940	IQGAP1	Ras GTPase-activ	sp|P46940|IQGA1_HUMAN	24	20.7	-0.20777	0.51615	0.9991	-0.2856	0.42628	0.61555	33	26.1	0.00692	0.98262	0.9973	-2.7166	######	######	4
576	P46977	STT3A	Dolichyl-diphosp	sp|P46977|STT3A_HUMAN	7	8.7	0.0192125	0.95111	0.9991	0.1998	0.52626	0.69224	8	10.2	0.00904	0.97698	0.9973	-0.9577	0.0067	0.0157	4
577	P47755	CAPZA2	F-actin-capping p	sp|P47755|CAZA2_HUMAN	6	37.1	0.2449797	0.39733	0.9991	0.4602	0.16368	0.33736	9	53.5	0.35729	0.22304	0.9973	-0.6255	0.0416	0.0739	5
578	P47756	CAPZB	F-actin-capping p	sp|P47756|CAPZB_HUMAN	8	39.7	0.2657413	0.26312	0.9991	-0.3316	0.214	0.40353	10	44.4	-0.0752	0.74677	0.9973	0.84944	0.002	0.0057	2
579	P47897	QARS	Glutamine--tRNA	sp|P47897|SYQ_HUMAN G	10	15.9	0.0447027	0.87949	0.9984	0.0611	0.87949	0.99836	11	17.8	0.02305	NA	NA	NA	NA	NA	NA
580	P48047	ATP5PO	ATP synthase su	sp|P48047|ATPO_HUMAN	5	23.9	0.0344474	0.91038	0.9987	0.2618	0.91038	0.99874	3	20.7	NA	NA	NA	NA	NA	NA	NA
581	P48147	PREP	Prolyl endopepti	sp|P48147|PPCE_HUMAN F	7	13.8	-0.162546	0.51584	0.9991	-0.6522	0.03191	0.12783	7	13	-0.233	0.35558	0.9973	-2.0415	######	0.0002	4
582	P48444	ARCN1	Coatomer subun	sp|P48444|COPD_HUMAN	10	21.5	-0.258991	0.38074	0.9991	-0.3454	0.38349	0.593	11	23.1	-0.1293	0.62075	0.9973	-0.7837	0.0172	0.0354	4
583	P48556	PSMD8	26S proteasome	sp|P48556|PSMD8_HUMA	4	14.3	-0.155391	0.51408	0.9991	-0.3424	0.20738	0.39404	6	18.9	-0.1949	0.41506	0.9973	-1.2402	######	0.0004	4
584	P48643	CCT5	T-complex protei	sp|P48643|TCPE_HUMAN T	28	61.4	-0.0554	0.74685	0.9991	-0.7754	0.00028	0.00995	32	74.3	-0.0042	0.98047	0.9973	-0.5377	0.0056	0.0137	3
585	P48681	NES	Nestin	sp|P48681|NEST_HUMAN	22	16.6	0.2481393	0.26499	0.9984	1.0455	0.26499	0.99836	51	43.6	NA	NA	NA	NA	NA	NA	NA
586	P48735	IDH2	Isocitrate dehyd	sp|P48735|IDHP_HUMAN I	3	8.2	NA	NA	NA	NA	NA	NA	12	27.9	-0.0843	0.79192	0.9991	1.19205	0.7919	0.9991	NA
587	P48739	PITPNB	Phosphatidylino	sp|P48739|PIPNB_HUMAN	3	14	0.2494786	0.39596	0.9991	-0.3257	0.32361	0.52252	7	39.1	-0.2291	0.4349	0.9973	2.55538	######	######	2
588	P49006	MARCKSL1	MARCKS-related	sp|P49006|MRP_HUMAN M	7	64.1	0.2974109	0.26009	0.9984	0.0275	0.26009	0.99836	5	68.7	NA	NA	NA	NA	NA	NA	NA
589	P49189	ALDH9A1	4-trimethylamin	sp|P49189|AL9A1_HUMAN	7	15.8	0.0499977	0.78222	0.9984	-0.119	0.78222	0.99836	4	9.9	NA	NA	NA	NA	NA	NA	NA
590	P49207	RPL34	60S ribosomal p	sp|P49207|RL34_HUMAN 6	2	14.5	0.1523998	0.77512	0.9991	-0.3144	0.6318	0.77639	3	20.5	0.51476	0.4375	0.9973	-0.3087	0.6179	0.7043	NA
591	P49257	LMAN1	Protein ERGIC-53	sp|P49257|LMAN1_HUMA	2	4.9	-0.827643	0.06527	0.9991	-0.406	0.42762	0.6158	4	12	0.59224	0.06259	0.9973	-1.3173	0.0079	0.0178	4
592	P49321	NASP	Nuclear autoant	sp|P49321|NASP_HUMAN	52	79.2	0.0952185	0.71659	0.9991	-0.0012	0.99648	0.99662	33	52.3	-0.2325	0.38026	0.9973	3.24391	######	######	1
593	P49327	FASN	Fatty acid synth	sp|P49327|FAS_HUMAN F	60	31	-0.066805	0.82132	0.9991	-1.0819	0.00181	0.02746	77	43.5	0.15342	0.60522	0.9973	5.30125	######	######	2
594	P49368	CCT3	T-complex protei	sp|P49368|TCPG_HUMAN T	23	56.5	-0.025459	0.9471	0.9991	-1.3566	0.00238	0.02957	26	61.1	0.13805	0.71954	0.9973	-1.0259	0.0151	0.0318	3
595	P49411	TUFM	Elongation facto	sp|P49411|EFTU_HUMAN	18	46	-0.068079	0.76893	0.9991	0.1255	0.58945	0.74628	17	45.1	0.0372	0.87236	0.9973	-0.0327	0.8876	0.908	NA
596	P49419	ALDH7A1	Alpha-aminoadip	sp|P49419|AL7A1_HUMAN	12	25.6	0.0294766	0.92216	0.999	0.207	0.92216	0.99899	10	22.6	NA	NA	NA	NA	NA	NA	NA
597	P49458	SRP9	Signal recogniti	sp|P49458|SRP09_HUMAN	7	60.5	0.1382686	0.55718	0.9984	0.0844	0.55718	0.99836	6	62.8	NA	NA	NA	NA	NA	NA	NA
598	P49585	PCYT1A	Choline-phospha	sp|P49585|PCY1A_HUMAN	2	8.4	0.2347976	NA	NA	NA	NA	NA	3	12.5	NA	NA	NA	NA	NA	NA	NA
599	P49588	AARS	Alanine--tRNA li	sp|P49588|SYAC_HUMAN A	21	28.7	0.1981962	0.53026	0.9991	-1.2546	0.00088	0.02141	23	30.4	-0.1382	0.66056	0.9973	-0.9377	0.0077	0.0175	3
600	P49589	CARS	Cysteine--tRNA l	sp|P49589|SYCC_HUMAN C	6	10.2	0.3672257	0.40468	0.9984	-0.208	0.40468	0.99836	5	8	NA	NA	NA	NA	NA	NA	NA
601	P49591	SARS	Serine--tRNA lig	sp|P49591|SYSC_HUMAN S	5	11.7	0.5959404	0.02085	0.9991	-0.1733	0.51303	0.68372	13	28.6	-0.161	0.49721	0.9973	-0.2837	0.2388	0.3235	4
602	P49720	PSMB3	Proteasome sub	sp|P49720|PSB3_HUMAN F	2	13.7	NA	NA	NA	NA	NA	NA	5	37.6	-0.1607	NA	NA	NA	NA	NA	NA
603	P49721	PSMB2	Proteasome sub	sp|P49721|PSB2_HUMAN F	4	28.4	0.3205343	0.46855	0.9991	-0.5559	0.24362	0.44232	5	33.8	-0.319	0.31231	0.9973	0.02686	0.9309	0.9415	NA
604	P49736	MCM2	DNA replication	sp|P49736|MCM2_HUMAN	15	23.7	-0.001712	0.99441	0.9991	-0.5231	0.04477	0.16277	17	23.6	-0.0701	0.7745	0.9973	-0.1143	0.6411	0.7215	3
605	P49755	TMED10	Transmembrane	sp|P49755|TMEDA_HUMA	4	15.5	-0.103702	0.7504	0.9991	0.5749	0.09227	0.24969	6	35.2	0.39561	0.28613	0.9973	0.9508	0.0177	0.0362	1
606	P49756	RBM25	RNA-binding pro	sp|P49756|RBM25_HUMAN	7	10	-0.10609	0.74222	0.9984	0.4015	0.74222	0.99836	8	13.6	0.03742	NA	NA	NA	NA	NA	NA
607	P49790	NUP153	Nuclear pore co	sp|P49790|NU153_HUMAN	5	4.4	-0.257651	0.59671	0.9984	0.1055	0.59671	0.99836	9	8.7	NA	NA	NA	NA	NA	NA	NA
608	P49792	RANBP2	E3 SUMO-protei	sp|P49792|RBP2_HUMAN E	28	12.4	0.3854077	0.05979	0.9984	0.4842	0.05979	0.99836	14	6.2	NA	NA	NA	NA	NA	NA	NA
609	P49903	SEPHS1	Selenide, water	sp|P49903|SPS1_HUMAN S	5	19.6	-0.047454	0.85708	0.9991	0.238	0.37221	0.57843	6	20.7	0.47679	0.08426	0.9973	1.12717	0.0005	0.0017	1
610	P49915	GMPS	GMP synthase [g	sp|P49915|GUAA_HUMAN	15	28.3	-0.100486	0.57826	0.9984	-0.2255	0.57826	0.99836	16	32.8	NA	NA	NA	NA	NA	NA	NA
611	P49916	LIG3	DNA ligase 3	sp|P49916|DNLI3_HUMAN	3	3.4	0.3801107	0.51972	0.9984	0.5134	0.51972	0.99836	6	7.1	NA	NA	NA	NA	NA	NA	NA
612	P50213	IDH3A	Isocitrate dehyd	sp|P50213|IDH3A_HUMAN	1	3.3	NA	NA	NA	NA	NA	NA	7	21	0.09108	0.77134	0.9991	-1.9934	0.7713	0.9991	NA
613	P50395	GDI2	Rab GDP dissocia	sp|P50395|GDIB_HUMAN F	23	60.2	0.1131169	0.5357	0.9991	-0.9322	7.94E-05	0.00704	32	78.9	0.16188	0.37842	0.9973	-0.4394	0.0255	0.049	3
614	P50402	EMD	Emerin	sp|P50402|EMD_HUMAN E	2	10.6	-0.289297	0.19298	0.9984	0.4488	0.19298	0.99836	5	21.7	NA	NA	NA	NA	NA	NA	NA
615	P50454	SERPINH1	Serpin H1	sp|P50454|SERPH_HUMAN	12	35.2	0.18404	0.44998	0.9991	0.2667	0.27818	0.47755	17	50.2	-0.0576	0.81162	0.9973	0.07899	0.744	0.798	NA
616	P50502	ST13	Hsc70-interactin	sp|P50502|F10A1_HUMAN	7	25.7	0.1339408	0.70569	0.9991	-1.2877	0.0019	0.0275	8	25.5	0.04495	0.89895	0.9973	2.02474	######	0.0002	2
617	P50552	VASP	Vasodilator-stim	sp|P50552|VASP_HUMAN	5	17.4	0.8764549	0.01023	0.9984	0.0103	0.01023	0.99836	3	8.9	NA	NA	NA	NA	NA	NA	NA
618	P50570	DNM2	Dynamin-2	sp|P50570|DYN2_HUMAN	2	3.7	NA	NA	NA	NA	NA	NA	3	4.8	0.01037	NA	NA	NA	NA	NA	NA
619	P50579	METAP2	Methionine ami	sp|P50579|MAP2_HUMAN	6	20.3	0.6314839	0.02428	0.9984	0.6875	0.02428	0.99836	6	19	0.01822	NA	NA	NA	NA	NA	NA
620	P50897	PPT1	Palmitoyl-protei	sp|P50897|PPT1_HUMAN F	2	8.8	0.5264991	0.18518	0.9984	1.478	0.18518	0.99836	3	11.1	NA	NA	NA	NA	NA	NA	NA

621	P50914	RPL14	60S ribosomal p	sp|P50914|RL14_HUMAN 6	4	20	0.5069398	0.09673	0.9991	-0.0679	0.86754	0.92126	5	24.7	-0.8569	0.02842	0.9973	0.55014	0.1058	0.1654	2
622	P50990	CCT8	T-complex prote	sp|P50990|TCPQ_HUMAN T	29	56.2	0.0125347	0.94171	0.9991	-0.5429	0.00527	0.04399	35	61.1	-0.123	0.47648	0.9973	-0.272	0.1263	0.1917	3
623	P50991	CCT4	T-complex prote	sp|P50991|TCPD_HUMAN T	19	46.9	0.1929626	0.39177	0.9991	-0.4704	0.04733	0.16946	26	59	0.13705	0.54072	0.9973	-0.3386	0.1418	0.2099	3
624	P50995	ANXA11	Annexin A11	sp|P50995|ANX11_HUMAN	4	10.7	-0.016753	0.93925	0.999	-0.0557	0.93925	0.99899	3	5.5	NA	NA	NA	NA	NA	NA	NA
625	P51114	FXR1	Fragile X menta	sp|P51114|FXR1_HUMAN F	11	26.4	-0.31616	0.14756	0.9991	0.1012	0.63294	0.77639	14	30.1	0.39539	0.07505	0.9973	-0.0668	0.7521	0.8039	NA
626	P51148	RAB5C	Ras-related prot	sp|P51148|RAB5C_HUMAN	5	27.8	-0.077431	0.77577	0.9991	-0.0403	0.89214	0.93506	7	42.1	0.32437	0.19497	0.9973	-0.0733	0.7632	0.81	NA
627	P51149	RAB7A	Ras-related prot	sp|P51149|RAB7A_HUMAN	6	34.8	0.3037135	0.13331	0.9991	0.1643	0.40499	0.60515	9	55.6	0.19945	0.31463	0.9973	0.55874	0.0101	0.0222	1
628	P51532	SMARCA4	Transcription ac	sp|P51532|SMCA4_HUMA	9	7.5	-0.006174	0.9826	0.999	0.7075	0.9826	0.99899	7	5.4	NA	NA	NA	NA	NA	NA	NA
629	P51571	SSR4	Translocon-asso	sp|P51571|SSRD_HUMAN T	3	17.9	NA	NA	NA	NA	NA	NA	5	36.4	0.31947	NA	NA	NA	NA	NA	NA
630	P51572	BCAP31	B-cell receptor-a	sp|P51572|BAP31_HUMAN	9	33.7	-0.277452	0.33388	0.9984	0.6324	0.33388	0.99836	9	29.7	0.22463	NA	NA	NA	NA	NA	NA
631	P51610	HCFC1	Host cell factor 1	sp|P51610|HCFC1_HUMAN	7	5.2	NA	NA	NA	NA	NA	NA	13	9.5	-0.4405	NA	NA	NA	NA	NA	NA
632	P51659	HSD17B4	Peroxisomal mul	sp|P51659|DHB4_HUMAN	19	35.3	-0.132366	0.58444	0.9991	0.202	0.40783	0.60515	10	16.7	0.23217	0.57976	0.9973	1.17283	0.0041	0.0104	1
633	P51665	PSMD7	26S proteasome	sp|P51665|PSMD7_HUMA	5	18.5	0.8045388	0.08075	0.9991	0.905	0.05219	0.17799	10	47.5	0.04509	0.91817	0.9973	-0.755	0.0994	0.1577	NA
634	P51858	HDGF	Hepatoma-deriv	sp|P51858|HDGF_HUMAN	8	43.3	0.4436116	0.10718	0.9991	0.1187	0.65442	0.79314	16	72.5	0.21287	0.42512	0.9973	3.31189	######	######	1
635	P51991	HNRNPA3	Heterogeneous	sp|P51991|ROA3_HUMAN	9	23.8	0.2659204	0.31058	0.9991	0.3765	0.15748	0.33132	18	39.7	0.09325	0.71835	0.9973	-0.1142	0.6589	0.7397	NA
636	P52209	PGD	6-phosphoglucon	sp|P52209|6PGD_HUMAN	14	42	-0.090784	0.67505	0.9991	-0.88	0.00074	0.01946	19	55.3	0.26884	0.22393	0.9973	1.14485	######	0.0003	2
637	P52272	HNRNPM	Heterogeneous	sp|P52272|HNRPM_HUMA	37	54.1	-0.230384	0.16212	0.9991	0.3785	0.02831	0.12313	30	48.1	-0.1603	0.32303	0.9973	0.48752	0.0068	0.0158	1
638	P52292	KPNA2	Importin subunit	sp|P52292|IMA1_HUMAN	11	32.9	-0.172679	0.40201	0.9991	-0.326	0.12347	0.28797	9	24.2	0.17743	0.38948	0.9973	-0.9255	0.0003	0.0011	4
639	P52294	KPNA1	Importin subunit	sp|P52294|IMA5_HUMAN	2	6.3	-0.52781	NA	NA	NA	NA	NA	5	14.3	NA	NA	NA	NA	NA	NA	NA
640	P52306	RAP1GDS1	Rap1 GTPase-GD	sp|P52306|GDS1_HUMAN	1	2.5	NA	NA	NA	NA	NA	NA	7	16.5	-0.0208	0.96296	0.9991	-0.339	0.963	0.9991	NA
641	P52434	POLR2H	DNA-directed RN	sp|P52434|RPAB3_HUMAN	2	14	NA	NA	NA	NA	NA	NA	4	26	0.23451	NA	NA	NA	NA	NA	NA
642	P52565	ARHGDIA	Rho GDP-dissocia	sp|P52565|GDIR1_HUMAN	6	36.8	0.1514804	0.47169	0.9991	-1.0593	9.00E-05	0.00709	10	51.5	-0.0227	0.91336	0.9973	-2.2022	######	######	4
643	P52597	HNRNPF	Heterogeneous	sp|P52597|HNRPF_HUMAN	10	30.4	-0.056185	0.8237	0.9991	0.4188	0.11045	0.27869	12	50.4	0.40066	0.12555	0.9973	0.1085	0.6677	0.7443	NA
644	P52701	MSH6	DNA mismatch r	sp|P52701|MSH6_HUMAN	18	15.1	0.0686909	0.73285	0.9984	-0.1553	0.73285	0.99836	11	10.3	NA	NA	NA	NA	NA	NA	NA
645	P52732	KIF11	Kinesin-like prot	sp|P52732|KIF11_HUMAN	12	13.3	0.1646705	0.39013	0.9984	0.1762	0.39013	0.99836	2	2.1	NA	NA	NA	NA	NA	NA	NA
646	P52788	SMS	Spermine syntha	sp|P52788|SPSY_HUMAN S	10	32	-0.026947	0.91614	0.9991	-0.763	0.00926	0.06252	10	44.3	0.30714	0.42916	0.9973	3.44206	######	######	2
647	P52789	HK2	Hexokinase-2	sp|P52789|HXK2_HUMAN	11	14.6	0.3621986	0.18204	0.9991	0.4911	0.11023	0.27869	19	24.1	0.0414	0.87515	0.9973	-0.3451	0.2023	0.2892	NA
648	P52815	MRPL12	39S ribosomal p	sp|P52815|RM12_HUMAN	5	35.4	-0.001462	0.99765	0.9991	0.8314	0.14838	0.31977	8	52.5	-0.1947	0.69583	0.9973	1.79746	0.0022	0.0061	1
649	P52888	THOP1	Thimet oligopep	sp|P52888|THOP1_HUMAN	4	7.4	NA	NA	NA	NA	NA	NA	10	14.9	-0.1068	0.68721	0.9991	-0.4336	0.6872	0.9991	NA
650	P52907	CAPZA1	F-actin-capping	sp|P52907|CAZA1_HUMAN	7	42.3	0.376226	0.44821	0.9991	0.2602	0.59796	0.75079	12	70.3	-0.2128	0.75962	0.9973	2.64431	0.0016	0.0048	1
651	P52926	HMGA2	High mobility gr	sp|P52926|HMGA2_HUMA	2	31.2	-0.274618	0.57371	0.9984	-0.2031	0.57371	0.99836	1	20.2	NA	NA	NA	NA	NA	NA	NA
652	P52948	NUP98	Nuclear pore cor	sp|P52948|NUP98_HUMAN	6	4.3	0.1730564	0.6399	0.9991	0.9972	0.0262	0.11746	12	8.4	0.29585	0.42702	0.9973	-0.3875	0.3017	0.3875	5
653	P53007	SLC25A1	Tricarboxylate t	sp|P53007|TXTP_HUMAN T	3	10	0.3053519	0.18424	0.9984	0.2937	0.18424	0.99836	1	3.5	NA	NA	NA	NA	NA	NA	NA
654	P53367	ARFIP1	Arfaptin-1	sp|P53367|ARFP1_HUMAN	3	11.8	0.5184125	NA	NA	NA	NA	NA	4	15.8	NA	NA	NA	NA	NA	NA	NA
655	P53396	ACLY	ATP-citrate synt	sp|P53396|ACLY_HUMAN A	24	26.5	0.0377208	0.88391	0.9991	-0.8937	0.0028	0.03304	33	39.3	0.00518	0.98401	0.9973	2.10607	######	######	2
656	P53618	COPB1	Coatomer subun	sp|P53618|COPB_HUMAN	7	11.1	1.1099223	0.02311	0.9991	0.2699	0.66847	0.8074	17	23.5	-0.1263	0.77643	0.9973	-2.3174	0.0001	0.0005	4
657	P53621	COPA	Coatomer subun	sp|P53621|COPA_HUMAN	14	13.3	-0.025374	0.89534	0.9991	-0.5299	0.0129	0.07316	26	27.5	-0.1542	0.42834	0.9973	-1.3868	######	######	4
658	P53634	CTSC	Dipeptidyl pepti	sp|P53634|CATC_HUMAN	2	8.9	1.200703	0.0367	0.9984	1.3136	0.0367	0.99836	4	13.8	NA	NA	NA	NA	NA	NA	NA
659	P53680	AP2S1	AP-2 complex su	sp|P53680|AP2S1_HUMAN	2	13.4	NA	NA	NA	NA	NA	NA	3	21.8	-0.0788	NA	NA	NA	NA	NA	NA
660	P53985	SLC16A1	Monocarboxylat	sp|P53985|MOT1_HUMAN	7	11.6	-0.056716	0.83981	0.9984	0.9555	0.83981	0.99836	5	11.4	NA	NA	NA	NA	NA	NA	NA
661	P53992	SEC24C	Protein transpor	sp|P53992|SC24C_HUMAN	2	3	NA	NA	NA	NA	NA	NA	7	9.2	0.21323	0.44455	0.9991	0.13649	0.4446	0.9991	NA
662	P53999	SUB1	Activated RNA p	sp|P53999|TCP4_HUMAN A	4	27.6	0.9584613	0.06625	0.9984	0.1606	0.06625	0.99836	5	36.2	NA	NA	NA	NA	NA	NA	NA
663	P54105	CLNS1A	Methylosome su	sp|P54105|ICLN_HUMAN M	4	31.6	0.1083739	NA	NA	NA	NA	NA	7	59.5	NA	NA	NA	NA	NA	NA	NA
664	P54136	RARS	Arginine--tRNA l	sp|P54136|SYRC_HUMAN A	13	24.8	0.1388507	0.56561	0.9991	-0.4987	0.05096	0.17666	26	43.3	0.27562	0.26111	0.9973	1.53419	######	######	2
665	P54577	YARS	Tyrosine--tRNA l	sp|P54577|SYYC_HUMAN T	14	26.7	0.0971818	0.68442	0.9991	-0.3544	0.19626	0.37977	20	43.6	0.08896	0.70974	0.9973	-0.5252	0.0404	0.0721	3
666	P54578	USP14	Ubiquitin carbox	sp|P54578|UBP14_HUMAN	12	29.1	0.3143034	0.35277	0.9991	-0.539	0.12003	0.2853	12	30.8	-0.2987	0.3765	0.9973	-0.4712	0.1705	0.2492	NA
667	P54652	HSPA2	Heat shock-relat	sp|P54652|HSP72_HUMAN	18	26.6	0.6947119	NA	NA	NA	NA	NA	NA	NA	NA	NA	NA	NA	NA	NA	NA
668	P54709	ATP1B3	Sodium/potassiu	sp|P54709|AT1B3_HUMAN	6	26.2	0.0479775	0.78967	0.9984	0.4847	0.78967	0.99836	6	24.7	NA	NA	NA	NA	NA	NA	NA

669	P54727	RAD23B	UV excision repa	sp|P54727|RD23B_HUMAN	7	20.5	-0.373569	0.22496	0.9991	-0.1597	0.59708	0.75079	9	26.9	0.08622	0.77466	0.9973	0.00072	0.9981	0.9986	NA
670	P54819	AK2	Adenylate kinas	sp|P54819|KAD2_HUMAN	5	23	-0.173676	0.63817	0.9991	-0.1722	0.70296	0.83624	13	69.9	-0.1332	0.61039	0.9973	-0.754	0.0116	0.0251	4
671	P54886	ALDH18A1	Delta-1-pyrrolin	sp|P54886|P5CS_HUMAN	12	19.2	0.2716137	0.3059	0.9991	0.5901	0.03518	0.13704	15	25.2	-0.079	0.76241	0.9973	0.32722	0.2207	0.305	1
672	P54920	NAPA	Alpha-soluble NS	sp|P54920|SNAA_HUMAN	4	18.3	0.4493976	0.12635	0.9991	0.0367	0.9266	0.9536	7	31.9	-0.3183	0.26915	0.9973	-1.3623	0.0002	0.0009	4
673	P55010	EIF5	Eukaryotic trans	sp|P55010|IF5_HUMAN Eu	9	22.3	0.2173425	0.27943	0.9991	-0.116	0.60004	0.75164	15	40.8	0.25289	0.21124	0.9973	1.06641	######	0.0003	2
674	P55036	PSMD4	26S proteasome	sp|P55036|PSMD4_HUMA	8	29.4	-0.124555	0.4976	0.9991	-0.0374	0.83764	0.91735	9	33.7	0.18883	0.30823	0.9973	-0.2772	0.1417	0.2099	NA
675	P55039	DRG2	Developmentall	sp|P55039|DRG2_HUMAN	2	5.8	NA	NA	NA	NA	NA	NA	2	5.8	-0.0415	NA	NA	NA	NA	NA	NA
676	P55060	CSE1L	Exportin-2	sp|P55060|XPO2_HUMAN	18	23.6	0.1363375	0.43559	0.9991	-0.4063	0.02966	0.12709	28	35.6	0.09144	0.59912	0.9973	-0.123	0.4811	0.5723	3
677	P55072	VCP	Transitional end	sp|P55072|TERA_HUMAN	37	59.6	-0.078134	0.81682	0.9991	-0.9279	0.01276	0.07295	42	66.3	0.07631	0.82101	0.9973	-0.792	0.0294	0.0554	3
678	P55081	MFAP1	Microfibrillar-as	sp|P55081|MFAP1_HUMA	2	4.8	NA	NA	NA	NA	NA	NA	4	14.6	1.12096	0.28661	0.9991	0.97431	0.2866	0.9991	NA
679	P55084	HADHB	Trifunctional enz	sp|P55084|ECHB_HUMAN	3	10.3	NA	NA	NA	NA	NA	NA	11	28.3	0.42224	0.21708	0.9991	0.91673	0.2171	0.9991	NA
680	P55145	MANF	Mesencephalic a	sp|P55145|MANF_HUMAN	2	6	NA	NA	NA	NA	NA	NA	7	41.8	0.18776	0.76164	0.9991	0.67625	0.7616	0.9991	NA
681	P55209	NAP1L1	Nucleosome ass	sp|P55209|NP1L1_HUMAN	7	19.2	0.5696548	0.27838	0.9991	-0.9917	0.06828	0.20962	11	39.4	-0.1855	0.71968	0.9973	-0.3188	0.539	0.6306	NA
682	P55265	ADAR	Double-strandec	sp|P55265|DSRAD_HUMA	14	14.9	0.125546	0.638	0.9984	0.6716	0.638	0.99836	15	16.6	NA	NA	NA	NA	NA	NA	NA
683	P55327	TPD52	Tumor protein D	sp|P55327|TPD52_HUMAN	2	11.6	NA	NA	NA	NA	NA	NA	6	39.7	-0.1279	0.68418	0.9991	0.02449	0.6842	0.9991	NA
684	P55735	SEC13	Protein SEC13 h	sp|P55735|SEC13_HUMAN	3	18	0.1706012	0.37841	0.9991	0.1248	0.62753	0.77546	6	27.6	0.6791	0.00129	0.4588	-0.0497	0.7713	0.8138	5
685	P55769	SNU13	NHP2-like prote	sp|P55769|NH2L1_HUMAN	5	39.8	0.2606126	0.32661	0.9991	0.4114	0.12956	0.29535	6	61.7	-0.1222	0.64177	0.9973	0.9834	0.0015	0.0044	1
686	P55786	NPEPPS	Puromycin-sensi	sp|P55786|PSA_HUMAN P	13	15.3	-0.279507	0.18911	0.9991	-1.2238	1.69E-05	0.00239	9	11.5	-0.1065	0.60863	0.9973	-0.6372	0.0064	0.0152	3
687	P55795	HNRNPH2	Heterogeneous	sp|P55795|HNRH2_HUMA	8	25.8	0.0178363	NA	NA	NA	NA	NA	8	26.3	-0.5958	0.25648	0.9991	-0.6995	0.2565	0.9991	NA
688	P55854	SUMO3	Small ubiquitin-r	sp|P55854|SUMO3_HUMA	2	20.4	0.2621872	0.18296	0.9984	-0.0534	0.18296	0.99836	2	20.4	NA	NA	NA	NA	NA	NA	NA
689	P55884	EIF3B	Eukaryotic trans	sp|P55884|EIF3B_HUMAN	15	21.7	0.0439908	0.84393	0.9991	-0.426	0.07022	0.21187	23	33.7	-0.0972	0.66444	0.9973	-0.5095	0.0338	0.0619	3
690	P56385	ATP5ME	ATP synthase su	sp|P56385|ATP5I_HUMAN	3	31.9	0.8020048	0.1196	0.9984	1.1286	0.1196	0.99836	2	30.4	NA	NA	NA	NA	NA	NA	NA
691	P56537	EIF6	Eukaryotic trans	sp|P56537|IF6_HUMAN Eu	5	30.2	0.0364617	0.88652	0.9991	0.2682	0.35549	0.55885	4	26.9	-0.164	0.60195	0.9973	0.98231	0.0037	0.0097	1
692	P56545	CTBP2	C-terminal-bindi	sp|P56545|CTBP2_HUMAN	4	16.4	0.0845056	0.60798	0.9984	-0.1738	0.60798	0.99836	6	20	NA	NA	NA	NA	NA	NA	NA
693	P56747	CLDN6	Claudin-6	sp|P56747|CLD6_HUMAN	2	8.2	-0.572129	0.19205	0.9984	0.196	0.19205	0.99836	NA	NA	NA	NA	NA	NA	NA	NA	NA
694	P57740	NUP107	Nuclear pore co	sp|P57740|NU107_HUMAN	5	6.1	0.1026805	0.71156	0.9991	0.1099	0.71794	0.84693	6	7.4	0.38967	0.17717	0.9973	-0.9247	0.0202	0.0404	4
695	P58546	MTPN	Myotrophin	sp|P58546|MTPN_HUMAN	3	28.8	0.7193357	NA	NA	NA	NA	NA	4	44.1	0.03291	0.89031	0.9991	0.74665	0.8903	0.9991	NA
696	Q99879	HIST1H2BM	Histone H2B typ	sp|Q99879|H2B1M_HUMA	11	60.3	-0.091418	0.83714	0.9991	-0.04	0.92832	0.9536	11	60.3	0.40245	0.37121	0.9973	-0.3206	0.4743	0.5671	NA
697	P59998	ARPC4	Actin-related pr	sp|P59998|ARPC4_HUMAN	4	22.6	0.0223883	0.91877	0.9991	0.0153	0.95013	0.96788	4	22.6	-0.0966	0.66092	0.9973	-1.3403	######	0.0001	4
698	P60033	CD81	CD81 antigen	sp|P60033|CD81_HUMAN	1	8.5	NA	NA	NA	NA	NA	NA	1	8.5	NA	NA	NA	NA	NA	NA	NA
699	P60059	SEC61G	Protein transpor	sp|P60059|SC61G_HUMAN	3	42.6	-0.357679	0.26388	0.9984	0.3923	0.26388	0.99836	2	36.8	0.07876	NA	NA	NA	NA	NA	NA
700	P60174	TPI1	Triosephosphate	sp|P60174|TPIS_HUMAN T	20	78.3	-0.035813	0.84307	0.9991	-0.8146	0.00029	0.00995	19	77.6	0.00925	0.9592	0.9973	1.53615	######	######	2
701	P60228	EIF3E	Eukaryotic trans	sp|P60228|EIF3E_HUMAN	10	29	-0.001255	0.99563	0.9991	-0.2345	0.31407	0.51427	13	35.1	-0.1727	0.45512	0.9973	-0.2358	0.3115	0.398	NA
702	P60520	GABARAPL2	Gamma-aminobu	sp|P60520|GBRL2_HUMAN	2	22.2	-0.320155	0.3386	0.9984	-0.2032	0.3386	0.99836	3	29.1	NA	NA	NA	NA	NA	NA	NA
703	P60660	MYL6	Myosin light pol	sp|P60660|MYL6_HUMAN	10	64.9	0.2903899	0.24911	0.9991	-0.3397	0.18079	0.35905	10	64.9	0.13699	0.58057	0.9973	0.21844	0.3817	0.4761	NA
704	P60842	EIF4A1	Eukaryotic initia	sp|P60842|IF4A1_HUMAN	20	51.5	0.0209199	0.91323	0.9991	-0.403	0.04851	0.17027	24	63.3	-0.0587	0.75998	0.9973	-0.7565	0.001	0.0032	4
705	P60866	RPS20	40S ribosomal p	sp|P60866|RS20_HUMAN	5	43.7	-0.204923	0.45146	0.9991	-0.4305	0.16668	0.34057	4	37	-0.1116	0.67986	0.9973	0.31593	0.2517	0.3348	NA
706	P60891	PRPS1	Ribose-phosphat	sp|P60891|PRPS1_HUMAN	5	19.8	-0.410076	0.5059	0.9984	0.2283	0.5059	0.99836	5	21.7	NA	NA	NA	NA	NA	NA	NA
707	P60900	PSMA6	Proteasome sub	sp|P60900|PSA6_HUMAN	6	32.1	0.4377611	0.09647	0.9991	0.0365	0.89662	0.93506	8	41.1	0.11605	0.64539	0.9973	-0.9003	0.0023	0.0066	4
708	P60903	S100A10	Protein S100-A1	sp|P60903|S10AA_HUMAN	1	10.3	NA	NA	NA	NA	NA	NA	2	19.6	-0.1863	NA	NA	NA	NA	NA	NA
709	P60953	CDC42	Cell division con	sp|P60953|CDC42_HUMAN	8	38.7	-0.441835	0.07515	0.9991	-0.2853	0.23715	0.43447	11	48.7	0.13585	0.56698	0.9973	-0.0381	0.8717	0.897	NA
710	P60981	DSTN	Destrin	sp|P60981|DEST_HUMAN	8	54.5	0.5595644	0.12579	0.9991	-0.6462	0.11469	0.27942	5	33.3	0.1595	0.65092	0.9973	-0.6897	0.064	0.1083	NA
711	P60983	GMFB	Glia maturation	sp|P60983|GMFB_HUMAN	5	39.4	-0.097831	0.72865	0.9984	-0.0362	0.72865	0.99836	5	27.5	NA	NA	NA	NA	NA	NA	NA
712	P61011	SRP54	Signal recogniti	sp|P61011|SRP54_HUMAN	3	6.2	0.0280609	0.89462	0.9984	-0.0914	0.89462	0.99836	6	15.3	-0.2765	NA	NA	NA	NA	NA	NA
713	P61019	RAB2A	Ras-related prot	sp|P61019|RAB2A_HUMAN	6	34.4	0.0548909	0.80703	0.9991	0.1367	0.54506	0.70555	6	34.4	0.22065	0.33286	0.9973	-1.1508	######	0.0004	4
714	P61026	RAB10	Ras-related prot	sp|P61026|RAB10_HUMAN	4	24.5	0.0660757	0.77466	0.9991	0.2444	0.29735	0.4984	6	33.5	0.08803	0.70317	0.9973	-0.0786	0.7336	0.7917	NA
715	P61081	UBE2M	NEDD8-conjugat	sp|P61081|UBC12_HUMAN	4	21.3	0.2975663	NA	NA	NA	NA	NA	4	20.8	0.97261	0.00992	0.9991	0.96863	0.0099	0.9991	NA
716	P61088	UBE2N	Ubiquitin-conjug	sp|P61088|UBE2N_HUMAN	4	36.8	-0.050143	0.85903	0.9991	-0.7906	0.01151	0.07028	10	80.9	0.27762	0.33235	0.9973	2.2958	######	######	2

717	P61106	RAB14	Ras-related prot	sp|P61106|RAB14_HUMAN	5	32.6	-0.02424	0.92691	0.9991	0.506	0.08812	0.24124	7	42.8	-0.1336	0.47872	0.9973	-0.4437	0.0305	0.0569	5
718	P61158	ACTR3	Actin-related pr	sp|P61158|ARP3_HUMAN	3	6.7	0.0635507	0.91959	0.9991	0.0884	0.88839	0.93452	11	36.1	0.07253	0.86285	0.9973	-0.8044	0.0734	0.1236	NA
719	P61160	ACTR2	Actin-related pr	sp|P61160|ARP2_HUMAN	6	17.8	0.19892	0.49138	0.9991	0.0113	0.96865	0.97692	7	24.1	0.05757	0.84107	0.9973	-1.521	######	0.0003	4
720	P61163	ACTR1A	Alpha-centractin	sp|P61163|ACTZ_HUMAN	4	18.4	0.4668361	NA	NA	NA	NA	NA	6	27.1	-0.0707	0.70052	0.9991	0.06957	0.7005	0.9991	NA
721	P61201	COPS2	COP9 signalosom	sp|P61201|CSN2_HUMAN	6	14.4	0.0829529	0.78482	0.9991	-0.1494	0.72821	0.85198	10	29.3	0.02972	0.92199	0.9973	-0.3867	0.2151	0.3009	NA
722	P84077	ARF1	ADP-ribosylation	sp|P84077|ARF1_HUMAN	4	33.7	0.4305792	0.13536	0.9991	-0.6063	0.04152	0.15175	7	54.7	0.06183	0.82433	0.9973	-1.41	######	0.0004	4
723	P61221	ABCE1	ATP-binding cass	sp|P61221|ABCE1_HUMAN	5	9.3	0.2201624	0.43737	0.9991	-0.1267	0.68718	0.82299	13	27.4	-0.0994	0.72381	0.9973	0.30676	0.2836	0.3683	NA
724	P61224	RAP1B	Ras-related prot	sp|P61224|RAP1B_HUMAN	3	19	-0.046205	0.85969	0.9991	-0.0657	0.85883	0.92074	8	42.9	-0.0873	0.73887	0.9973	-1.1712	0.0004	0.0016	4
725	P61247	RPS3A	40S ribosomal p	sp|P61247|RS3A_HUMAN	13	38.3	0.0693547	0.77903	0.9991	-0.435	0.09212	0.24969	18	61.4	-0.1846	0.4584	0.9973	-0.3775	0.1396	0.2075	NA
726	P61254	RPL26	60S ribosomal p	sp|P61254|RL26_HUMAN 6	6	37.2	0.1749386	0.53461	0.9991	0.5506	0.06279	0.19963	7	37.9	0.01738	0.9505	0.9973	-0.8586	0.0066	0.0154	5
727	P61289	PSME3	Proteasome acti	sp|P61289|PSME3_HUMAN	6	26.8	0.263291	0.23449	0.9991	0.0718	0.76675	0.87681	12	55.9	-0.1489	0.4946	0.9973	0.67693	0.006	0.0145	1
728	P61313	RPL15	60S ribosomal p	sp|P61313|RL15_HUMAN 6	2	11.3	NA	NA	NA	NA	NA	NA	7	33.3	-0.2347	0.67213	0.9991	2.33823	0.6721	0.9991	NA
729	P61326	MAGOH	Protein mago na	sp|P61326|MGN_HUMAN	7	37	NA	NA	NA	NA	NA	NA	7	43.8	0.14221	0.56237	0.9991	0.1082	0.5624	0.9991	NA
730	P61353	RPL27	60S ribosomal p	sp|P61353|RL27_HUMAN 6	5	26.5	-0.180074	0.60178	0.9991	-0.3548	0.30968	0.50825	6	32.4	-0.3589	0.30429	0.9973	-0.0962	0.7797	0.8214	NA
731	P61421	ATP6V0D1	V-type proton A	sp|P61421|VA0D1_HUMAN	2	6.6	NA	NA	NA	NA	NA	NA	3	9.7	-0.2418	NA	NA	NA	NA	NA	NA
732	P61513	RPL37A	60S ribosomal p	sp|P61513|RL37A_HUMAN	3	29.3	-0.005171	0.9846	0.999	-0.0688	0.9846	0.99899	1	19.6	NA	NA	NA	NA	NA	NA	NA
733	P61586	RHOA	Transforming pr	sp|P61586|RHOA_HUMAN	3	18.7	NA	NA	NA	NA	NA	NA	7	51.3	0.19225	0.59048	0.9991	0.6067	0.5905	0.9991	NA
734	P61604	HSPE1	10 kDa heat sho	sp|P61604|CH10_HUMAN	8	68.6	-0.07144	0.81837	0.9991	0.5639	0.08366	0.23536	10	74.5	0.31798	0.31405	0.9973	1.62221	######	0.0003	1
735	P61619	SEC61A1	Protein transpor	sp|P61619|S61A1_HUMAN	3	6.1	-0.111437	0.78045	0.9984	1.5568	0.78045	0.99836	3	6.7	-0.3764	NA	NA	NA	NA	NA	NA
736	P61758	VBP1	Prefoldin subuni	sp|P61758|PFD3_HUMAN	1	6.1	NA	NA	NA	NA	NA	NA	8	46.2	-0.1822	0.61503	0.9991	0.89587	0.615	0.9991	NA
737	P61923	COPZ1	Coatomer subun	sp|P61923|COPZ1_HUMAN	2	13.6	-0.02486	NA	NA	NA	NA	NA	3	22	NA	NA	NA	NA	NA	NA	NA
738	P61956	SUMO2	Small ubiquitin-	sp|P61956|SUMO2_HUMA	2	23.2	-0.525962	0.1171	0.9991	-0.0464	0.88564	0.9344	2	23.2	0.04382	0.89205	0.9973	-0.7126	0.0392	0.0705	4
739	P61970	NUTF2	Nuclear transpo	sp|P61970|NTF2_HUMAN	3	24.4	0.51663	0.13082	0.9991	-0.6108	0.11164	0.27928	5	56.7	-0.0904	0.78376	0.9973	-0.6788	0.0529	0.0916	NA
740	P61978	HNRNPK	Heterogeneous	sp|P61978|HNRPK_HUMAN	26	56.4	-0.088142	0.58293	0.9991	0.1138	0.47971	0.65533	25	64.8	0.05722	0.72075	0.9973	0.91175	######	0.0002	1
741	P61981	YWHAG	14-3-3 protein g	sp|P61981|1433G_HUMAN	16	62.8	0.0369342	0.83901	0.9991	-0.4906	0.01425	0.07895	17	77.3	-0.1246	0.49607	0.9973	1.02849	######	0.0002	2
742	P62070	RRAS2	Ras-related prot	sp|P62070|RRAS2_HUMAN	2	9.3	0.1096402	0.8261	0.9984	-1.132	0.8261	0.99836	NA	NA	NA	NA	NA	NA	NA	NA	NA
743	P62081	RPS7	40S ribosomal p	sp|P62081|RS7_HUMAN 4	11	64.4	-0.005991	0.98311	0.9991	-0.3167	0.27229	0.4737	13	61.3	0.08651	0.76021	0.9973	-0.8842	0.0058	0.014	4
744	P62140	PPP1CB	Serine/threonin	sp|P62140|PP1B_HUMAN	4	15.3	NA	NA	NA	NA	NA	NA	15	55.7	0.37944	0.17134	0.9991	1.09342	0.1713	0.9991	NA
745	P62191	PSMC1	26S proteasome	sp|P62191|PRS4_HUMAN	11	34.3	-0.061063	0.75227	0.9991	-0.4221	0.04097	0.1513	14	44.5	0.23342	0.23717	0.9973	-0.7653	0.0009	0.0031	4
746	P62195	PSMC5	26S proteasome	sp|P62195|PRS8_HUMAN	13	42.9	0.0271766	0.87532	0.9991	-0.0325	0.85114	0.91851	16	53	-0.0214	0.90163	0.9973	-0.4265	0.0233	0.0458	4
747	P62241	RPS8	40S ribosomal p	sp|P62241|RS8_HUMAN 4	6	26.9	0.3219622	0.58077	0.9991	-0.8896	0.13863	0.3043	9	46.2	-0.3249	0.57733	0.9973	-0.1806	0.7559	0.8049	NA
748	P62244	RPS15A	40S ribosomal p	sp|P62244|RS15A_HUMAN	5	30.8	-0.327826	0.47618	0.9991	0.1481	0.77186	0.88037	6	36.9	-0.0081	0.98577	0.9973	1.1043	0.0262	0.0499	1
749	P62249	RPS16	40S ribosomal p	sp|P62249|RS16_HUMAN	10	56.2	0.0432977	0.90913	0.9991	-0.8333	0.04002	0.14855	9	54.8	0.05317	0.88855	0.9973	-0.593	0.1315	0.1972	3
750	P62253	UBE2G1	Ubiquitin-conjug	sp|P62253|UB2G1_HUMAN	1	7.1	NA	NA	NA	NA	NA	NA	2	16.5	0.03902	NA	NA	NA	NA	NA	NA
751	P62258	YWHAE	14-3-3 protein e	sp|P62258|1433E_HUMAN	20	67.5	0.1083765	0.56106	0.9991	-0.5444	0.00867	0.06085	20	75.7	0.03417	0.85388	0.9973	-1.3553	######	######	4
752	P62263	RPS14	40S ribosomal p	sp|P62263|RS14_HUMAN	5	31.8	0.5861178	0.14802	0.9991	-0.5972	0.14098	0.3085	7	39.1	0.55445	0.16977	0.9973	0.47016	0.2405	0.3248	NA
753	P62266	RPS23	40S ribosomal p	sp|P62266|RS23_HUMAN	3	21	0.9939365	0.01274	0.9991	0.2608	0.47355	0.65068	4	28.7	0.26447	0.46741	0.9973	-0.9606	0.0155	0.0325	5
754	P62269	RPS18	40S ribosomal p	sp|P62269|RS18_HUMAN	8	37.5	1.3073385	0.00102	0.7197	0.3248	0.33603	0.536	10	52.6	-0.5089	0.13959	0.9973	-1.2379	0.0016	0.0047	5
755	P62277	RPS13	40S ribosomal p	sp|P62277|RS13_HUMAN	4	26.5	-0.12428	0.70432	0.9991	-0.466	0.16649	0.34057	7	47	-0.0999	0.76003	0.9973	1.24346	0.0013	0.0041	2
756	P62280	RPS11	40S ribosomal p	sp|P62280|RS11_HUMAN	5	22.8	0.1341827	0.74805	0.9991	-0.0545	0.89603	0.93506	9	40.5	-0.2336	0.5772	0.9973	-0.7801	0.0753	0.1262	NA
757	P62306	SNRPF	Small nuclear ri	sp|P62306|RUXF_HUMAN	2	24.4	0.3076112	0.46241	0.9984	0.4901	0.46241	0.99836	2	24.4	-0.0305	NA	NA	NA	NA	NA	NA
758	P62314	SNRPD1	Small nuclear ri	sp|P62314|SMD1_HUMAN	3	43.7	0.4414644	0.27052	0.9991	0.0529	0.89299	0.93506	3	43.7	0.59938	0.14065	0.9973	-0.1928	0.6251	0.7114	NA
759	P62316	SNRPD2	Small nuclear ri	sp|P62316|SMD2_HUMAN	4	39.8	0.6641614	0.02408	0.9991	0.3256	0.24022	0.43895	6	55.9	-0.2361	0.38957	0.9973	-0.6236	0.0326	0.0603	5
760	P62318	SNRPD3	Small nuclear ri	sp|P62318|SMD3_HUMAN	6	54.8	-0.08517	0.71409	0.9991	-0.2963	0.25461	0.45129	6	54.8	-0.0787	0.70516	0.9973	0.26999	0.2061	0.2935	NA
761	P62330	ARF6	ADP-ribosylation	sp|P62330|ARF6_HUMAN	3	24.6	NA	NA	NA	NA	NA	NA	3	24.6	0.42676	0.44725	0.9991	-1.4646	0.4472	0.9991	NA
762	P62333	PSMC6	26S proteasome	sp|P62333|PRS10_HUMAN	11	38.3	0.0219164	0.92258	0.9991	-0.3725	0.11249	0.27928	14	46.3	0.12359	0.58537	0.9973	0.39089	0.097	0.1553	NA
763	P62424	RPL7A	60S ribosomal p	sp|P62424|RL7A_HUMAN	7	24.8	0.3322452	0.51689	0.9991	-0.0984	0.84686	0.91851	9	33.8	0.23397	0.647	0.9973	0.7116	0.1747	0.2549	NA
764	P62491	RAB11A	Ras-related prot	sp|P62491|RB11A_HUMAN	8	37	-0.049649	0.81528	0.9991	-0.0755	0.72279	0.84844	10	49.1	-0.4276	0.05732	0.9973	0.37925	0.0881	0.1435	NA

765	P62495	ETF1	Eukaryotic pept	sp|P62495|ERF1_HUMAN E	13	40	0.0049231	0.97487	0.9991	-0.6523	0.0006	0.01696	13	36.6	0.02397	0.8781	0.9973	-0.89	######	0.0002	3
766	P62633	CNBP	Cellular nucleic a	sp|P62633|CNBP_HUMAN C	3	15.3	NA	NA	NA	NA	NA	NA	7	42.4	-0.4188	0.34481	0.9991	-0.201	0.3448	0.9991	NA
767	P62701	RPS4X	40S ribosomal p	sp|P62701|RS4X_HUMAN 4	14	52.1	0.0234207	0.92123	0.9991	-0.4067	0.09999	0.26258	16	55.1	-0.0649	0.78434	0.9973	-0.7743	0.0042	0.0108	4
768	P67775	PPP2CA	Serine/threonine	sp|P67775|PP2AA_HUMAN	2	8.4	NA	NA	NA	NA	NA	NA	8	41.7	-0.1955	0.6693	0.9991	-0.8153	0.6693	0.9991	NA
769	P62750	RPL23A	60S ribosomal p	sp|P62750|RL23A_HUMAN	7	43.6	0.1503742	0.61001	0.9991	0.079	0.78801	0.88965	8	45.5	0.08767	0.76556	0.9973	0.13874	0.6377	0.7188	NA
770	P62753	RPS6	40S ribosomal p	sp|P62753|RS6_HUMAN 40	6	20.9	0.2233786	0.62809	0.9991	-0.2859	0.53626	0.7002	8	25.7	-0.1022	0.82414	0.9973	-0.142	0.7577	0.8054	NA
771	P62760	VSNL1	Visinin-like prot	sp|P62760|VISL1_HUMAN	7	37.2	0.3141829	0.2759	0.9984	0.1292	0.2759	0.99836	NA	NA	NA	NA	NA	NA	NA	NA	NA
772	P62805	HIST1H4A	Histone H4	sp|P62805|H4_HUMAN His	11	60.2	-0.400551	0.321	0.9991	0.0196	0.96071	0.97306	11	63.1	0.36278	0.36744	0.9973	0.43561	0.2818	0.3673	NA
773	P62820	RAB1A	Ras-related prot	sp|P62820|RAB1A_HUMAN	10	58	-0.13273	0.59447	0.9991	0.3864	0.13312	0.29964	11	62	0.15269	0.54083	0.9973	-0.3891	0.1306	0.1962	NA
774	P62826	RAN	GTP-binding nuc	sp|P62826|RAN_HUMAN G	7	30.1	0.3011265	0.18408	0.9991	-0.4477	0.05545	0.18284	9	44.4	0.15898	0.4744	0.9973	0.0784	0.7226	0.7823	NA
775	P62829	RPL23	60S ribosomal p	sp|P62829|RL23_HUMAN 6	3	18.6	-0.949202	0.00514	0.9991	-0.4513	0.18549	0.36632	7	62.9	0.05657	0.84852	0.9973	-0.127	0.6687	0.7443	3
776	P62841	RPS15	40S ribosomal p	sp|P62841|RS15_HUMAN 4	1	8.3	NA	NA	NA	NA	NA	NA	5	57.9	-0.0076	0.98959	0.9973	-0.5297	0.3683	0.463	NA
777	P62847	RPS24	40S ribosomal p	sp|P62847|RS24_HUMAN 4	5	39.8	0.2350329	0.47485	0.9991	-0.1784	0.58628	0.74361	4	33.8	0.16739	0.60938	0.9973	-1.0682	0.0042	0.0107	4
778	P62851	RPS25	40S ribosomal p	sp|P62851|RS25_HUMAN 4	3	22.4	-0.099456	0.78478	0.9991	-0.6561	0.12134	0.2853	4	31.2	-0.0897	0.80546	0.9973	1.89214	######	0.0004	2
779	P62854	RPS26	40S ribosomal p	sp|P62854|RS26_HUMAN 4	1	13	NA	NA	NA	NA	NA	NA	3	27	-0.9001	0.025	0.9991	-0.0055	0.025	0.9991	NA
780	P62857	RPS28	40S ribosomal p	sp|P62857|RS28_HUMAN 4	5	56.5	0.1080976	0.84909	0.9991	0.2289	0.74251	0.8616	5	56.5	0.14152	0.7251	0.9973	1.74708	0.0008	0.0025	1
781	P62873	GNB1	Guanine nucleot	sp|P62873|GBB1_HUMAN G	8	29.4	0.0578879	0.81869	0.9991	0.2175	0.39418	0.60102	10	32.6	-0.0618	0.80659	0.9973	-0.6448	0.0193	0.0391	5
782	P62875	POLR2L	DNA-directed RN	sp|P62875|RPAB5_HUMAN	2	29.9	NA	NA	NA	NA	NA	NA	2	29.9	0.07296	0.87616	0.9991	0.98381	0.8762	0.9991	NA
783	P62879	GNB2	Guanine nucleot	sp|P62879|GBB2_HUMAN G	5	19.4	NA	NA	NA	NA	NA	NA	9	36.2	-0.0123	0.96571	0.9991	0.63996	0.9657	0.9991	NA
784	P62888	RPL30	60S ribosomal p	sp|P62888|RL30_HUMAN 6	6	55.7	0.0674135	0.87236	0.9991	-0.0325	0.93817	0.96022	7	66.1	0.24112	0.56738	0.9973	-1.1297	0.0145	0.0306	4
785	P62899	RPL31	60S ribosomal p	sp|P62899|RL31_HUMAN 6	8	47.2	0.2884424	0.40472	0.9991	0.1422	0.6788	0.81572	6	39.2	0.05667	0.8686	0.9973	-0.2905	0.4015	0.4959	NA
786	P62906	RPL10A	60S ribosomal p	sp|P62906|RL10A_HUMAN	8	42.4	0.1759507	0.5227	0.9991	-0.6452	0.0289	0.12495	10	48.4	-0.0089	0.97393	0.9973	-0.7227	0.0161	0.0338	3
787	P62910	RPL32	60S ribosomal p	sp|P62910|RL32_HUMAN 6	2	17	NA	NA	NA	NA	NA	NA	3	22.2	0.11699	0.85491	0.9991	0.50252	0.8549	0.9991	NA
788	P62913	RPL11	60S ribosomal p	sp|P62913|RL11_HUMAN 6	4	20.8	0.0655392	0.80367	0.9991	0.0457	0.8623	0.92074	5	28.7	-0.0668	0.79996	0.9973	-0.0632	0.8106	0.8465	NA
789	P62917	RPL8	60S ribosomal p	sp|P62917|RL8_HUMAN 60	2	9.3	0.3764509	0.4217	0.9991	0.1199	0.79619	0.89461	5	18.7	-0.4697	0.31879	0.9973	0.06718	0.8849	0.9079	NA
790	P62937	PPIA	Peptidyl-prolyl c	sp|P62937|PPIA_HUMAN P	12	73.9	0.2432771	0.3669	0.9991	-0.9432	0.00234	0.02957	13	74.5	-0.1703	0.52486	0.9973	2.02207	######	######	2
791	P62942	FKBP1A	Peptidyl-prolyl c	sp|P62942|FKB1A_HUMAN	2	29.6	NA	NA	NA	NA	NA	NA	6	64.8	0.00608	0.99024	0.9992	0.74819	0.9902	0.9992	NA
792	P62995	TRA2B	Transformer-2 p	sp|P62995|TRA2B_HUMAN	5	19.8	-0.733889	0.12252	0.9991	0.8837	0.06889	0.20962	3	13.9	0.00599	0.98655	0.9973	-0.185	0.6049	0.6929	NA
793	P63010	AP2B1	AP-2 complex su	sp|P63010|AP2B1_HUMAN	12	14.6	-0.005087	0.98003	0.9991	0.2817	0.1779	0.35476	21	25	-0.4135	0.05502	0.9973	-0.2172	0.2934	0.3782	NA
794	Q15836	VAMP3	Vesicle-associate	sp|Q15836|VAMP3_HUMA	1	24	NA	NA	NA	NA	NA	NA	3	40	-0.066	0.73781	0.9991	-1.5783	0.7378	0.9991	NA
795	P63092	GNAS	Guanine nucleot	sp|P63092|GNAS2_HUMAN	2	6.1	NA	NA	NA	NA	NA	NA	12	16.5	-0.0191	0.93922	0.9991	2.08617	0.9392	0.9991	NA
796	P63098	PPP3R1	Calcineurin subu	sp|P63098|CANB1_HUMAN	1	7.1	NA	NA	NA	NA	NA	NA	5	32.9	-0.1992	0.40765	0.9991	0.16838	0.4077	0.9991	NA
797	P63104	YWHAZ	14-3-3 protein ze	sp|P63104|1433Z_HUMAN	18	68.6	0.2219987	0.20023	0.9991	-0.6213	0.00174	0.02746	21	72.2	-0.0947	0.57694	0.9973	0.39276	0.0309	0.0575	2
798	P63151	PPP2R2A	Serine/threonine	sp|P63151|2ABA_HUMAN	3	8.7	NA	NA	NA	NA	NA	NA	7	21.5	0.10953	0.64474	0.9991	-0.338	0.6447	0.9991	NA
799	P63165	SUMO1	Small ubiquitin-r	sp|P63165|SUMO1_HUMA	4	35.6	0.4277881	0.36315	0.9991	0.7689	0.11296	0.27928	4	36.6	0.65667	0.32485	0.9973	3.03838	######	######	1
800	P63167	DYNLL1	Dynein light cha	sp|P63167|DYL1_HUMAN D	2	37.1	-0.11552	0.83393	0.9991	0.1762	0.77497	0.8815	3	44.9	-0.2912	0.59854	0.9973	-1.3931	0.021	0.0415	4
801	P63173	RPL38	60S ribosomal p	sp|P63173|RL38_HUMAN 6	2	28.6	-0.090247	0.84551	0.9991	-0.4468	0.34275	0.54364	3	38.6	0.15783	0.73367	0.9973	-0.8309	0.0611	0.1039	NA
802	P63208	SKP1	S-phase kinase-a	sp|P63208|SKP1_HUMAN S	7	60.7	-0.451928	0.10071	0.9991	-0.3359	0.21397	0.40353	10	65.6	-0.3612	0.18303	0.9973	-0.107	0.6859	0.7593	NA
803	P63220	RPS21	40S ribosomal p	sp|P63220|RS21_HUMAN 4	4	48.2	0.0357224	0.8963	0.9991	-0.0848	0.75743	0.87178	6	67.5	0.62568	0.03367	0.9973	0.75788	0.0124	0.0266	2
804	P63241	EIF5A	Eukaryotic trans	sp|P63241|IF5A1_HUMAN	7	69.5	0.2089738	0.3078	0.9991	-0.464	0.03245	0.12854	13	88.3	0.15636	0.44218	0.9973	0.15244	0.4535	0.545	3
805	P63244	RACK1	Receptor of activ	sp|P63244|RACK1_HUMAN	15	49.2	-0.058547	0.71051	0.9991	-0.7218	0.00025	0.00995	18	62.1	0.13106	0.41005	0.9973	-0.5531	0.0025	0.007	3
806	P63261	ACTG1	Actin, cytoplasm	sp|P63261|ACTG_HUMAN	29	88.8	-0.204962	0.30324	0.9991	-0.4316	0.03942	0.1471	31	88.3	-0.0845	0.66704	0.9973	-0.6202	0.0053	0.0131	3
807	P63279	UBE2I	SUMO-conjugati	sp|P63279|UBC9_HUMAN S	2	13.9	0.5010419	0.21995	0.9991	-1.0383	0.03126	0.12742	5	46.8	0.67983	0.10273	0.9973	2.90912	######	######	2
808	P67809	YBX1	Nuclease-sensitiv	sp|P67809|YBOX1_HUMAN	14	54	0.305585	0.20772	0.9991	0.3841	0.11827	0.28521	14	54	-0.0156	0.94727	0.9973	0.25466	0.2902	0.3754	NA
809	P67870	CSNK2B	Casein kinase II s	sp|P67870|CSK2B_HUMAN	3	21.9	0.3616235	0.24702	0.9991	0.6182	0.11654	0.28201	5	29.3	0.34232	0.17152	0.9973	-0.18	0.4571	0.5484	NA
810	P67936	TPM4	Tropomyosin alp	sp|P67936|TPM4_HUMAN	25	77.4	0.2895723	0.23768	0.9991	0.1256	0.60227	0.7531	30	80.2	0.06249	0.79469	0.9973	0.07128	0.7667	0.8125	NA
811	P68133	ACTA1	Actin, alpha ske	sp|P68133|ACTS_HUMAN A	17	38.7	-0.463123	0.1921	0.9991	-0.1178	0.76036	0.87233	14	32.4	0.175	0.61349	0.9973	0.04704	0.8916	0.9082	NA
812	P68104	EEF1A1	Elongation facto	sp|P68104|EF1A1_HUMAN	30	61.7	-0.152417	0.43746	0.9991	-0.9618	0.00012	0.00755	28	72.5	0.00427	0.98246	0.9973	-0.2412	0.2255	0.3087	3

813	P68363	TUBA1B	Tubulin alpha-1B	sp|P68363|TBA1B_HUMAN	17	45.7	0.0822906	0.73751	0.9991	-0.8057	0.00407	0.03837	26	66.1	0.22368	0.36758	0.9973	0.97935	0.0009	0.0029	2
814	P68366	TUBA4A	Tubulin alpha-4A	sp|P68366|TBA4A_HUMAN	14	36.8	0.1447371	NA	NA	NA	NA	NA	22	57.8	0.20123	NA	NA	NA	NA	NA	NA
815	P68371	TUBB4B	Tubulin beta-4B	sp|P68371|TBB4B_HUMAN	17	41.6	-0.189852	0.29231	0.9991	-1.2091	3.00E-06	0.00188	23	68.3	-0.0856	0.63034	0.9973	1.67607	######	######	2
816	P68400	CSNK2A1	Casein kinase II	sp|P68400|CSK21_HUMAN	2	7.2	-0.70289	NA	NA	NA	NA	NA	7	27.4	0.11216	0.71479	0.9991	-0.1714	0.7148	0.9991	NA
817	P69849	NOMO3	Nodal modulato	sp|P69849|NOMO3_HUMA	12	13.5	0.0806778	0.77697	0.9991	0.4178	0.15486	0.32873	9	9.3	-0.0468	0.86926	0.9973	-1.0974	0.0012	0.0037	5
818	P78344	EIF4G2	Eukaryotic trans	sp|P78344|IF4G2_HUMAN	7	7.2	0.3357318	0.16386	0.9991	-0.3888	0.24893	0.44682	16	18.5	0.06676	0.77464	0.9973	-0.4815	0.0534	0.0921	NA
819	P78347	GTF2I	General transcri	sp|P78347|GTF2I_HUMAN	15	18.8	-0.044007	0.83228	0.9984	0.3702	0.83228	0.99836	14	18.8	NA	NA	NA	NA	NA	NA	NA
820	P78371	CCT2	T-complex prote	sp|P78371|TCPB_HUMAN T	24	62.8	0.1015651	0.59093	0.9991	-0.4382	0.03066	0.12742	28	69.9	0.00721	0.96944	0.9973	-0.5912	0.0056	0.0136	3
821	P78527	PRKDC	DNA-dependent	sp|P78527|PRKDC_HUMAN	55	15.1	-0.033761	0.93992	0.9991	0.0784	0.86109	0.92074	68	20.7	-0.4901	0.37829	0.9973	5.84147	######	######	1
822	P80723	BASP1	Brain acid solubl	sp|P80723|BASP1_HUMAN	14	76.7	0.3595211	0.25605	0.9984	0.3116	0.25605	0.99836	12	68.3	NA	NA	NA	NA	NA	NA	NA
823	P82650	MRPS22	28S ribosomal p	sp|P82650|RT22_HUMAN 2	4	10.8	0.2537301	0.34242	0.9984	0.5372	0.34242	0.99836	5	14.4	NA	NA	NA	NA	NA	NA	NA
824	P82970	HMGN5	High mobility gr	sp|P82970|HMGN5_HUMA	6	27.7	0.2954159	0.32623	0.9984	0.5923	0.32623	0.99836	2	8.2	NA	NA	NA	NA	NA	NA	NA
825	P82979	SARNP	SAP domain-con	sp|P82979|SARNP_HUMAN	7	34.3	0.1519241	0.61561	0.9991	0.3352	0.27501	0.47673	7	35.7	0.1249	0.67938	0.9973	1.67861	######	0.0002	1
826	P83916	CBX1	Chromobox prote	sp|P83916|CBX1_HUMAN C	6	41.6	0.3682383	0.10108	0.9991	0.2219	0.4674	0.64919	4	30.3	-0.1647	0.44608	0.9973	-1.2521	######	0.0002	4
827	P84085	ARF5	ADP-ribosylation	sp|P84085|ARF5_HUMAN A	4	31.7	0.2683051	0.49602	0.9991	0.1168	0.80692	0.9038	7	56.1	0.0502	0.8555	0.9973	-1.0481	0.0197	0.0397	4
828	P84090	ERH	Enhancer of rudi	sp|P84090|ERH_HUMAN E	3	27.9	-0.096773	0.72746	0.9984	-0.1323	0.72746	0.99836	4	44.2	NA	NA	NA	2.81819	NA	NA	NA
829	P84098	RPL19	60S ribosomal p	sp|P84098|RL19_HUMAN 6	1	8.7	NA	NA	NA	NA	NA	NA	4	21.9	-2.1766	0.00251	0.9991	0.74138	0.0025	0.9991	NA
830	P84103	SRSF3	Serine/arginine-	sp|P84103|SRSF3_HUMAN	5	35.4	0.1168877	0.71021	0.9991	1.0441	0.00374	0.03699	6	35.4	-0.1131	0.71909	0.9973	2.34088	######	######	1
831	P99999	CYCS	Cytochrome c	sp|P99999|CYC_HUMAN Cy	3	27.6	-0.448914	0.2236	0.9984	-1.2782	0.2236	0.99836	3	30.5	NA	NA	NA	NA	NA	NA	NA
832	Q00059	TFAM	Transcription fac	sp|Q00059|TFAM_HUMAN	2	6.1	0.9521459	0.01515	0.9984	0.0059	0.01515	0.99836	5	19.9	NA	NA	NA	NA	NA	NA	NA
833	Q00169	PITPNA	Phosphatidylino	sp|Q00169|PIPNA_HUMAN	1	7	NA	NA	NA	NA	NA	NA	7	39.6	0.05429	NA	NA	NA	NA	NA	NA
834	Q00325	SLC25A3	Phosphate carri	sp|Q00325|MPCP_HUMAN	6	15.2	-0.46251	0.11023	0.9991	0.1601	0.56677	0.72534	4	13	-0.0134	0.96149	0.9973	-0.4684	0.1061	0.1654	NA
835	Q00610	CLTC	Clathrin heavy c	sp|Q00610|CLH1_HUMAN	29	22.9	-0.036898	0.88653	0.9991	-0.1957	0.45309	0.63992	58	46.4	-0.0415	0.87251	0.9973	-0.2293	0.3808	0.4761	NA
836	Q00688	FKBP3	Peptidyl-prolyl c	sp|Q00688|FKBP3_HUMAN	5	25	0.023262	0.95402	0.9991	-0.3114	0.44431	0.63003	10	50	0.21766	0.59119	0.9973	2.76258	######	######	2
837	Q00765	REEP5	Receptor expres	sp|Q00765|REEP5_HUMAN	3	14.8	-0.288587	0.3214	0.9984	0.9898	0.3214	0.99836	2	10.6	NA	NA	NA	NA	NA	NA	NA
838	Q00839	HNRNPU	Heterogeneous	sp|Q00839|HNRPU_HUMA	22	34.4	-0.115917	0.56825	0.9991	0.322	0.12482	0.2883	19	28.5	0.03571	0.85984	0.9973	-0.3473	0.0998	0.1579	NA
839	Q01082	SPTBN1	Spectrin beta ch	sp|Q01082|SPTB2_HUMAN	50	28.6	0.0322471	0.85664	0.9991	0.297	0.10988	0.27869	72	41.8	-0.0815	0.64894	0.9973	-0.5779	0.0045	0.0114	5
840	Q01085	TIAL1	Nucleolysin TIAR	sp|Q01085|TIAR_HUMAN N	3	7.2	-0.222604	0.38023	0.9991	-0.016	0.96377	0.97378	5	16.3	-0.2622	0.48795	0.9973	0.79436	0.0411	0.0732	1
841	Q01105	SET	Protein SET	sp|Q01105|SET_HUMAN P	5	24.1	0.325981	0.18646	0.9991	-0.2684	0.27259	0.4737	7	33.8	-0.0521	0.82839	0.9973	1.16687	0.0001	0.0006	2
842	Q01130	SRSF2	Serine/arginine-	sp|Q01130|SRSF2_HUMAN	5	22.2	0.2018965	0.56964	0.9991	-0.0072	0.98381	0.98799	4	21.7	-0.1121	0.75136	0.9973	-0.6356	0.0861	0.1419	NA
843	Q01469	FABP5	Fatty acid-bindi	sp|Q01469|FABP5_HUMAN	7	49.6	0.0940588	0.70259	0.9984	-1.4995	0.70259	0.99836	8	67.4	NA	NA	NA	NA	NA	NA	NA
844	Q01518	CAP1	Adenylyl cyclase	sp|Q01518|CAP1_HUMAN	16	39.2	-0.002034	0.99446	0.9991	-1.3717	0.0002	0.00956	20	54.3	0.16448	0.57618	0.9973	-0.0923	0.7529	0.8039	3
845	Q01581	HMGCS1	Hydroxymethylg	sp|Q01581|HMCS1_HUMA	15	37.9	-0.054675	0.74777	0.9984	-0.7169	0.74777	0.99836	5	11.2	NA	NA	NA	NA	NA	NA	NA
846	Q01628	IFITM3	Interferon-induc	sp|Q01628|IFM3_HUMAN	3	48.9	-0.433914	0.06258	0.9984	-0.0999	0.06258	0.99836	NA	NA	NA	NA	NA	NA	NA	NA	NA
847	Q01780	EXOSC10	Exosome compor	sp|Q01780|EXOSX_HUMAN	6	8.2	-0.074097	0.75966	0.9984	0.3207	0.75966	0.99836	2	2.8	NA	NA	NA	NA	NA	NA	NA
848	Q01813	PFKP	ATP-dependent	sp|Q01813|PFKAP_HUMAN	5	9.8	-0.532998	NA	NA	NA	NA	NA	9	14.8	NA	NA	NA	NA	NA	NA	NA
849	Q01844	EWSR1	RNA-binding pro	sp|Q01844|EWS_HUMAN R	4	9	0.1342201	0.63843	0.9991	-0.0758	0.79032	0.89084	4	9	-0.0028	0.99202	0.9973	1.19192	0.0006	0.002	2
850	Q01995	TAGLN	Transgelin	sp|Q01995|TAGL_HUMAN	4	21.4	-0.084502	0.72852	0.9984	-0.8513	0.72852	0.99836	NA	NA	NA	NA	NA	NA	NA	NA	NA
851	Q02218	OGDH	2-oxoglutarate d	sp|Q02218|ODO1_HUMAN	7	8.7	-0.213413	0.38325	0.9991	0.1612	0.50762	0.68136	16	20.9	-0.0242	0.90217	0.9973	-1.0787	######	0.0004	4
852	Q02543	RPL18A	60S ribosomal p	sp|Q02543|RL18A_HUMAN	5	27.3	0.0770135	0.90742	0.9991	0.3222	0.62781	0.77546	6	34.1	0.16271	0.80605	0.9973	0.8549	0.2079	0.2937	NA
853	Q02790	FKBP4	Peptidyl-prolyl c	sp|Q02790|FKBP4_HUMAN	20	51.6	0.1290141	0.80614	0.9991	-0.4203	0.4282	0.6158	22	54.5	0.60729	0.25719	0.9973	2.6247	0.0001	0.0005	2
854	Q02818	NUCB1	Nucleobindin-1	sp|Q02818|NUCB1_HUMAI	6	17.8	-0.244747	0.55845	0.9991	0.1029	0.82502	0.91397	18	51.4	0.7939	0.07093	0.9973	3.24773	######	######	1
855	Q02878	RPL6	60S ribosomal p	sp|Q02878|RL6_HUMAN 60	8	21.2	0.058634	0.91464	0.9991	0.7299	0.19382	0.37903	12	31.9	-0.1322	0.80922	0.9973	1.28821	0.0291	0.0549	1
856	Q02880	TOP2B	DNA topoisomer	sp|Q02880|TOP2B_HUMAN	17	11.8	0.0131019	0.95179	0.999	0.0026	0.95179	0.99899	11	8.4	NA	NA	NA	NA	NA	NA	NA
857	Q02952	AKAP12	A-kinase anchor	sp|Q02952|AKA12_HUMAI	29	26	0.2392011	0.39562	0.9984	0.4099	0.39562	0.99836	18	15.6	NA	NA	NA	NA	NA	NA	NA
858	Q02978	SLC25A11	Mitochondrial 2-	sp|Q02978|M2OM_HUMA	3	12.7	-0.273694	0.45901	0.9984	0.0254	0.45901	0.99836	2	10.2	NA	NA	NA	NA	NA	NA	NA
859	Q03252	LMNB2	Lamin-B2	sp|Q03252|LMNB2_HUMA	13	27.3	-0.008133	0.97081	0.999	0.7395	0.97081	0.99899	14	21	NA	NA	NA	0.65824	NA	NA	NA
860	Q03701	CEBPZ	CCAAT/enhance	sp|Q03701|CEBPZ_HUMAN	6	6.8	0.1747354	0.64939	0.9991	0.7233	0.07708	0.22519	8	9.7	0.52584	0.31602	0.9973	1.04314	0.0162	0.0339	1

861	Q04637	EIF4G1	Eukaryotic trans	sp|Q04637|IF4G1_HUMAN	24	18.1	0.0910313	0.57415	0.9991	0.1883	0.25241	0.44979	27	19.9	-0.1471	0.36753	0.9973	-1.1763	######	######	4
862	Q04760	GLO1	Lactoylglutathio	sp|Q04760|LGUL_HUMAN	2	20.1	0.2624325	0.54987	0.9984	0.0108	0.54987	0.99836	9	52.2	NA	NA	NA	NA	NA	NA	NA
863	Q04837	SSBP1	Single-stranded	sp|Q04837|SSBP_HUMAN	5	43.9	0.2064509	0.47569	0.9984	1.0357	0.47569	0.99836	4	37.8	NA	NA	NA	NA	NA	NA	NA
864	Q04917	YWHAH	14-3-3 protein e	sp|Q04917|1433F_HUMAN	14	56.1	0.0011327	0.99492	0.9991	-0.3735	0.04849	0.17027	15	60.2	-0.1369	0.44571	0.9973	-1.3294	######	######	4
865	Q05086	UBE3A	Ubiquitin-protei	sp|Q05086|UBE3A_HUMAN	1	1.6	NA	NA	NA	NA	NA	NA	2	3.7	0.24062	0.42214	0.9991	-2.2011	0.4221	0.9991	NA
866	Q05519	SRSF11	Serine/arginine-	sp|Q05519|SRS11_HUMAN	3	7.9	0.5833983	NA	NA	NA	NA	NA	4	13.6	0.20936	0.77829	0.9991	1.667	0.7783	0.9991	NA
867	Q05682	CALD1	Caldesmon	sp|Q05682|CALD1_HUMAN	12	20.6	0.0733744	0.81563	0.9984	0.2076	0.81563	0.99836	10	16	NA	NA	NA	1.8144	NA	NA	NA
868	Q06210	GFPT1	Glutamine--fruct	sp|Q06210|GFPT1_HUMAN	10	19	0.219781	0.33072	0.9991	-0.3906	0.22594	0.41936	18	35.2	0.26155	0.25029	0.9973	-0.5258	0.03	0.0561	3
869	Q06265	EXOSC9	Exosome comple	sp|Q06265|EXOS9_HUMAN	2	4.8	0.2757244	0.168	0.9984	0.203	0.168	0.99836	2	5	NA	NA	NA	NA	NA	NA	NA
870	Q06323	PSME1	Proteasome acti	sp|Q06323|PSME1_HUMA	4	18.1	0.1450276	0.54942	0.9991	-0.444	0.11393	0.27928	16	68.3	0.01356	0.95511	0.9973	-0.7617	0.0056	0.0137	4
871	Q06830	PRDX1	Peroxiredoxin-1	sp|Q06830|PRDX1_HUMAN	13	60.3	-0.109596	0.65446	0.9991	-0.9529	0.00107	0.02437	15	74.4	0.20095	0.41526	0.9973	1.80077	######	######	2
872	Q07020	RPL18	60S ribosomal p	sp|Q07020|RL18_HUMAN 6	4	22.9	1.1359789	0.00301	0.899	-0.0057	0.00301	0.89902	5	30.9	NA	NA	NA	NA	NA	NA	NA
873	Q07021	C1QBP	Complement cor	sp|Q07021|C1QBP_HUMAN	6	39.7	-0.037337	0.89975	0.9984	0.0691	0.89975	0.99836	8	51.8	NA	NA	NA	NA	NA	NA	NA
874	Q07065	CKAP4	Cytoskeleton-as	sp|Q07065|CKAP4_HUMAN	14	27.4	-0.12222	0.71828	0.9991	0.7849	0.03117	0.12742	27	51.8	-0.0446	0.89507	0.9973	1.42828	0.0005	0.0019	1
875	Q07157	TJP1	Tight junction pr	sp|Q07157|ZO1_HUMAN T	10	6.9	0.1756238	0.46417	0.9984	0.425	0.46417	0.99836	2	1.6	0.33909	NA	NA	NA	NA	NA	NA
876	Q07666	KHDRBS1	KH domain-conta	sp|Q07666|KHDR1_HUMAN	9	26.2	0.0661784	0.81757	0.9991	0.5407	0.07309	0.21774	8	17.2	0.47008	0.11492	0.9973	-0.058	0.8398	0.8667	NA
877	Q07866	KLC1	Kinesin light cha	sp|Q07866|KLC1_HUMAN K	6	12.6	NA	NA	NA	NA	NA	NA	22	43.3	0.11005	0.82533	0.9991	4.56383	0.8253	0.9991	NA
878	Q07955	SRSF1	Serine/arginine-	sp|Q07955|SRSF1_HUMAN	8	31.5	-0.093461	0.7973	0.9991	0.7765	0.04513	0.16324	12	47.2	0.4689	0.20836	0.9973	1.88075	######	0.0004	1
879	Q07960	ARHGAP1	Rho GTPase-acti	sp|Q07960|RHG01_HUMAN	6	16.9	0.3906131	0.26225	0.9984	0.2131	0.26225	0.99836	4	10.3	0.39437	NA	NA	NA	NA	NA	NA
880	Q08211	DHX9	ATP-dependent	sp|Q08211|DHX9_HUMAN	30	31.5	-0.124185	0.63079	0.9991	0.1668	0.51989	0.6877	34	36.4	0.52875	0.05304	0.9973	2.05441	######	######	1
881	Q08257	CRYZ	Quinone oxidore	sp|Q08257|QOR_HUMAN Q	3	12.2	0.1591592	0.49248	0.9984	0.1447	0.49248	0.99836	4	18.8	NA	NA	NA	NA	NA	NA	NA
882	Q08431	MFGE8	Lactadherin	sp|Q08431|MFGM_HUMA	3	9.6	NA	NA	NA	-0.8714	NA	NA	NA	NA	NA	NA	NA	NA	NA	NA	NA
883	Q08752	PPID	Peptidyl-prolyl c	sp|Q08752|PPID_HUMAN P	9	29.2	0.0327766	0.88563	0.9991	-0.9146	0.01165	0.07028	10	32.7	-0.2041	0.37687	0.9973	-0.6075	0.0165	0.0343	3
884	Q08945	SSRP1	FACT complex su	sp|Q08945|SSRP1_HUMAN	10	18.5	-0.107509	0.57449	0.9991	0.1614	0.40221	0.60515	11	20.7	0.0687	0.71898	0.9973	0.31532	0.1119	0.1728	NA
885	Q08J23	NSUN2	tRNA (cytosine(3	sp|Q08J23|NSUN2_HUMAN	7	12.3	-0.014928	0.95836	0.9991	-0.2283	0.47025	0.64919	10	15.4	-0.1758	0.49539	0.9973	0.10995	0.6683	0.7443	NA
886	Q09028	RBBP4	Histone-binding	sp|Q09028|RBBP4_HUMAN	11	26.8	0.3805896	0.09519	0.9991	0.3191	0.15662	0.33049	17	69.4	0.05346	0.80662	0.9973	-0.0704	0.7474	0.8004	NA
887	Q09161	NCBP1	Nuclear cap-bind	sp|Q09161|NCBP1_HUMAN	2	2.7	-0.144851	0.38638	0.9984	0.0581	0.38638	0.99836	4	5.1	NA	NA	NA	NA	NA	NA	NA
888	Q0VF96	CGNL1	Cingulin-like pro	sp|Q0VF96|CGNL1_HUMAN	5	5.1	0.2449277	0.50549	0.9984	0.6828	0.50549	0.99836	NA	NA	NA	NA	NA	NA	NA	NA	NA
889	Q10570	CPSF1	Cleavage and po	sp|Q10570|CPSF1_HUMAN	3	2.6	-0.091942	0.65917	0.9984	0.5107	0.65917	0.99836	4	3.5	0.72745	NA	NA	NA	NA	NA	NA
890	O43896	KIF1C	Kinesin-like prot	sp|O43896|KIF1C_HUMAN	1	1	NA	NA	NA	NA	NA	NA	1	1.2	NA	NA	NA	NA	NA	NA	NA
891	Q12788	TBL3	Transducin beta-	sp|Q12788|TBL3_HUMAN T	4	6.7	0.2000647	0.54499	0.9984	-0.0408	0.54499	0.99836	9	18.3	-0.004	NA	NA	NA	NA	NA	NA
892	Q12824	SMARCB1	SWI/SNF-related	sp|Q12824|SNF5_HUMAN	3	11.4	-0.016547	0.92664	0.999	0.6079	0.92664	0.99899	2	8.1	NA	NA	NA	NA	NA	NA	NA
893	Q12849	GRSF1	G-rich sequence	sp|Q12849|GRSF1_HUMAN	8	27.9	-0.179537	0.3539	0.9984	0.2776	0.3539	0.99836	7	26.5	NA	NA	NA	NA	NA	NA	NA
894	Q12874	SF3A3	Splicing factor 3	sp|Q12874|SF3A3_HUMAN	7	19.6	-0.303359	0.26776	0.9991	-0.1263	0.67434	0.81173	15	36.9	-0.2913	0.28642	0.9973	-0.2036	0.4519	0.5449	NA
895	Q12888	TP53BP1	TP53-binding pro	sp|Q12888|TP53B_HUMAN	15	10.7	0.3619341	0.22839	0.9991	0.8442	0.01168	0.07028	19	14.2	-0.7461	0.15658	0.9973	0.4348	0.3022	0.3875	1
896	Q12904	AIMP1	Aminoacyl tRNA	sp|Q12904|AIMP1_HUMAN	6	26.6	0.0044577	NA	NA	NA	NA	NA	7	35.6	-0.0891	0.68234	0.9991	0.03922	0.6823	0.9991	NA
897	Q12905	ILF2	Interleukin enha	sp|Q12905|ILF2_HUMAN In	15	56.2	0.0685505	0.81158	0.9991	0.2611	0.36946	0.57825	13	51.3	-0.007	0.98056	0.9973	1.28773	0.0003	0.0012	1
898	Q12906	ILF3	Interleukin enha	sp|Q12906|ILF3_HUMAN In	33	45	-0.047772	0.78515	0.9991	0.192	0.28153	0.48213	26	35.1	0.09713	0.58078	0.9973	1.88406	######	######	1
899	Q12907	LMAN2	Vesicular integr	sp|Q12907|LMAN2_HUMA	2	5.6	-0.557981	NA	NA	NA	NA	NA	11	39.3	NA	NA	NA	NA	NA	NA	NA
900	Q12931	TRAP1	Heat shock prot	sp|Q12931|TRAP1_HUMAN	10	18.3	0.2795567	0.41184	0.9991	0.2572	0.44947	0.63607	21	36.5	-0.0868	0.79695	0.9973	2.19671	######	######	1
901	Q12996	CSTF3	Cleavage stimul	sp|Q12996|CSTF3_HUMAN	5	8.2	0.0312475	0.87041	0.9984	0.1483	0.87041	0.99836	7	12.3	NA	NA	NA	NA	NA	NA	NA
902	Q13011	ECH1	Delta(3,5)-Delta	sp|Q13011|ECH1_HUMAN	6	25.3	-0.132423	0.60021	0.9984	-0.0671	0.60021	0.99836	7	26.5	NA	NA	NA	NA	NA	NA	NA
903	Q13042	CDC16	Cell division cycl	sp|Q13042|CDC16_HUMAN	2	4.4	-0.351262	NA	NA	NA	NA	NA	2	3.9	-0.0197	NA	NA	NA	NA	NA	NA
904	Q13045	FLII	Protein flightles	sp|Q13045|FLII_HUMAN Pr	4	4	NA	NA	NA	NA	NA	NA	4	3.8	0.26321	NA	NA	NA	NA	NA	NA
905	Q13123	IK	Protein Red	sp|Q13123|RED_HUMAN P	6	12.2	-0.19486	0.38769	0.9991	0.811	0.01978	0.09821	7	13.5	-0.396	0.09123	0.9973	-0.6661	0.0085	0.0191	5
906	Q13126	MTAP	S-methyl-5-thioa	sp|Q13126|MTAP_HUMAN	5	21.6	NA	NA	NA	NA	NA	NA	9	38.2	-0.1289	0.62576	0.9991	-0.8417	0.6258	0.9991	NA
907	Q13148	TARDBP	TAR DNA-bindin	sp|Q13148|TADBP_HUMAN	8	18.1	-0.065114	0.77517	0.9991	0.593	0.01743	0.09153	7	18.4	0.01504	0.94732	0.9973	-1.4071	######	######	5
908	Q13151	HNRNPA0	Heterogeneous	sp|Q13151|ROA0_HUMAN	5	15.7	0.5177723	0.12087	0.9984	0.7243	0.12087	0.99836	7	36.1	NA	NA	NA	NA	NA	NA	NA

909	Q13153	PAK1	Serine/threonin	sp|Q13153|PAK1_HUMAN	8	18	0.1392688	NA	NA	NA	NA	NA	6	15.6	NA	NA	NA	NA	NA	NA	NA
910	Q13162	PRDX4	Peroxiredoxin-4	sp|Q13162|PRDX4_HUMAN	8	33.6	-0.023437	0.95186	0.9991	0.0397	0.91859	0.95217	13	52.8	-0.2147	0.58206	0.9973	1.75742	0.0003	0.0011	1
911	Q13177	PAK2	Serine/threonin	sp|Q13177|PAK2_HUMAN	10	26.3	-0.107827	NA	NA	NA	NA	NA	16	39.5	0.09095	0.65742	0.9991	0.78389	0.6574	0.9991	NA
912	Q13185	CBX3	Chromobox prot	sp|Q13185|CBX3_HUMAN	9	48.6	0.1789043	0.5353	0.9991	-0.1136	0.69273	0.82824	9	48.6	0.12456	0.66503	0.9973	-0.2282	0.4308	0.524	NA
913	Q13188	STK3	Serine/threonin	sp|Q13188|STK3_HUMAN	2	8.4	1.1521147	NA	NA	NA	NA	NA	NA	NA	NA	NA	NA	NA	NA	NA	NA
914	Q13200	PSMD2	26S proteasome	sp|Q13200|PSMD2_HUMA	19	22.2	0.0072026	0.9722	0.9991	0.4306	0.05001	0.17466	25	33	-0.2795	0.18809	0.9973	0.04613	0.8235	0.8548	NA
915	Q13242	SRSF9	Serine/arginine-	sp|Q13242|SRSF9_HUMAN	5	24.9	-0.069844	0.80363	0.9984	0.4864	0.80363	0.99836	9	46.6	NA	NA	NA	NA	NA	NA	NA
916	Q13247	SRSF6	Serine/arginine-	sp|Q13247|SRSF6_HUMAN	7	14.5	-0.127659	0.7319	0.9991	0.4615	0.22539	0.41936	6	15.4	-0.0384	0.91782	0.9973	1.70861	0.0002	0.001	1
917	Q13263	TRIM28	Transcription int	sp|Q13263|TIF1B_HUMAN	28	50.5	-0.106005	0.55094	0.9991	0.1502	0.40059	0.60515	21	40.8	-0.3267	0.07855	0.9973	-0.8839	0.0001	0.0005	4
918	Q13283	G3BP1	Ras GTPase-activ	sp|Q13283|G3BP1_HUMAN	11	30.3	0.1517213	0.54425	0.9991	0.149	0.55142	0.71084	13	36.7	-0.202	0.42152	0.9973	1.01884	0.0007	0.0024	1
919	Q13310	PABPC4	Polyadenylate-b	sp|Q13310|PABP4_HUMAN	14	24.7	0.1676896	0.52317	0.9991	0.1465	0.57631	0.73358	17	31.8	0.05838	0.82313	0.9973	0.3642	0.1752	0.2551	NA
920	Q13330	MTA1	Metastasis-asso	sp|Q13330|MTA1_HUMAN	3	5.2	0.5105294	NA	NA	NA	NA	NA	6	11.2	NA	NA	NA	NA	NA	NA	NA
921	Q13347	EIF3I	Eukaryotic trans	sp|Q13347|EIF3I_HUMAN	6	23.1	-0.162894	0.51917	0.9991	-0.6041	0.02624	0.11746	13	50.5	-0.0092	0.97072	0.9973	-0.3958	0.1286	0.194	3
922	Q13404	UBE2V1	Ubiquitin-conjug	sp|Q13404|UB2V1_HUMAN	5	44.2	0.0439752	0.88119	0.9984	-0.9865	0.88119	0.99836	6	46.9	NA	NA	NA	NA	NA	NA	NA
923	Q13405	MRPL49	39S ribosomal p	sp|Q13405|RM49_HUMAN	3	23.5	1.4964326	NA	NA	NA	NA	NA	3	23.5	NA	NA	NA	NA	NA	NA	NA
924	Q13409	DYNC1I2	Cytoplasmic dyn	sp|Q13409|DC1I2_HUMAN	5	11.8	0.4167876	0.0893	0.9984	0.3072	0.0893	0.99836	7	18	NA	NA	NA	NA	NA	NA	NA
925	Q13428	TCOF1	Treacle protein	sp|Q13428|TCOF_HUMAN	11	10.1	0.4657567	0.1471	0.9984	0.6228	0.1471	0.99836	16	13.8	NA	NA	NA	NA	NA	NA	NA
926	Q13435	SF3B2	Splicing factor 3	sp|Q13435|SF3B2_HUMAN	12	19.1	0.1938218	0.66843	0.9991	0.4772	0.2985	0.49915	25	34.6	0.44789	0.3282	0.9973	1.91635	0.0005	0.0018	1
927	Q13442	PDAP1	28 kDa heat- an	sp|Q13442|HAP28_HUMA	6	29.8	0.199307	0.38445	0.9991	-0.1873	0.4631	0.64633	6	29.8	0.37302	0.11405	0.9973	1.58157	######	######	2
928	Q13451	FKBP5	Peptidyl-prolyl c	sp|Q13451|FKBP5_HUMAN	6	17.1	0.4217714	0.33563	0.9984	1.9375	0.33563	0.99836	6	18.4	NA	NA	NA	NA	NA	NA	NA
929	Q13509	TUBB3	Tubulin beta-3 c	sp|Q13509|TBB3_HUMAN	11	28.4	0.3638608	0.34649	0.9984	-0.8424	0.34649	0.99836	20	60.4	NA	NA	NA	NA	NA	NA	NA
930	Q13526	PIN1	Peptidyl-prolyl c	sp|Q13526|PIN1_HUMAN	1	6.1	NA	NA	NA	NA	NA	NA	5	39.3	#######	0.99993	0.9999	0.75766	0.9999	0.9999	NA
931	Q13547	HDAC1	Histone deacety	sp|Q13547|HDAC1_HUMA	5	9.5	NA	NA	NA	NA	NA	NA	7	17	0.27187	NA	NA	NA	NA	NA	NA
932	Q13561	DCTN2	Dynactin subuni	sp|Q13561|DCTN2_HUMA	6	22.2	0.6321923	0.07653	0.9991	0.4427	0.36031	0.56518	15	45.9	0.24004	0.48033	0.9973	0.56407	0.1101	0.1709	NA
933	Q13564	NAE1	NEDD8-activatin	sp|Q13564|ULA1_HUMAN	5	12.9	-0.29101	0.44275	0.9984	-0.1097	0.44275	0.99836	6	16.3	NA	NA	NA	NA	NA	NA	NA
934	Q13576	IQGAP2	Ras GTPase-activ	sp|Q13576|IQGA2_HUMAN	6	4.4	NA	NA	NA	NA	NA	NA	4	2.4	0.59196	NA	NA	NA	NA	NA	NA
935	Q13596	SNX1	Sorting nexin-1	sp|Q13596|SNX1_HUMAN	3	6.1	NA	NA	NA	NA	NA	NA	10	22.2	0.01237	0.9714	0.9991	-0.1231	0.9714	0.9991	NA
936	Q13601	KRR1	KRR1 small subu	sp|Q13601|KRR1_HUMAN	2	7.3	0.2779487	0.60926	0.9984	0.2857	0.60926	0.99836	3	9.7	NA	NA	NA	NA	NA	NA	NA
937	Q13616	CUL1	Cullin-1	sp|Q13616|CUL1_HUMAN	2	3.6	NA	NA	NA	NA	NA	NA	5	10.4	-0.0543	NA	NA	NA	NA	NA	NA
938	Q13813	SPTAN1	Spectrin alpha c	sp|Q13813|SPTN1_HUMAN	54	27.5	-0.09847	0.66926	0.9991	-0.1956	0.40011	0.60515	97	47.5	-0.149	0.51949	0.9973	0.25418	0.2777	0.3633	NA
939	Q13838	DDX39B	Spliceosome RN	sp|Q13838|DX39B_HUMAN	11	33.6	0.0007171	0.99726	0.9991	-0.1804	0.39285	0.60102	11	29	0.2445	0.25123	0.9973	-0.4885	0.03	0.0561	4
940	Q13907	IDI1	Isopentenyl-diph	sp|Q13907|IDI1_HUMAN	4	22.9	0.4133857	NA	NA	NA	NA	NA	3	18.5	NA	NA	NA	NA	NA	NA	NA
941	Q14008	CKAP5	Cytoskeleton-as	sp|Q14008|CKAP5_HUMAN	14	7.7	-0.04768	0.87928	0.9991	-0.1722	0.625	0.77546	20	11.7	0.28337	0.3729	0.9973	0.59088	0.0745	0.1251	NA
942	Q14019	COTL1	Coactosin-like p	sp|Q14019|COTL1_HUMAN	3	19	0.0694378	0.81491	0.9984	-0.3992	0.81491	0.99836	3	30.3	NA	NA	NA	NA	NA	NA	NA
943	Q14103	HNRNPD	Heterogeneous	sp|Q14103|HNRPD_HUMA	11	29.9	-0.078784	0.76268	0.9991	-0.2762	0.29729	0.4984	16	36.3	0.14823	0.57133	0.9973	1.94437	######	######	2
944	Q14126	DSG2	Desmoglein-2	sp|Q14126|DSG2_HUMAN	8	11.2	-0.261114	0.35476	0.9984	0.6343	0.35476	0.99836	NA	NA	NA	NA	NA	NA	NA	NA	NA
945	Q14151	SAFB2	Scaffold attachm	sp|Q14151|SAFB2_HUMAN	10	16.8	-0.072456	0.77612	0.9984	0.7329	0.77612	0.99836	11	16.5	NA	NA	NA	NA	NA	NA	NA
946	Q14152	EIF3A	Eukaryotic trans	sp|Q14152|EIF3A_HUMAN	19	16.6	0.439351	0.15025	0.9991	-0.738	0.02177	0.10155	32	23.4	0.01431	0.96139	0.9973	-0.9348	0.0053	0.0131	3
947	Q14157	UBAP2L	Ubiquitin-associ	sp|Q14157|UBP2L_HUMAN	11	15.8	0.182275	0.6662	0.9991	-0.1553	0.71296	0.84477	15	24.4	0.48329	0.31309	0.9973	3.5279	######	######	1
948	Q14165	MLEC	Malectin	sp|Q14165|MLEC_HUMAN	8	32.2	-0.23514	0.33335	0.9991	0.6048	0.02055	0.09992	5	21.9	0.06917	0.77303	0.9973	-0.7025	0.0087	0.0194	5
949	Q14166	TTLL12	Tubulin--tyrosine	sp|Q14166|TTL12_HUMAN	7	13.8	0.3728489	0.386	0.9991	-0.0897	0.85028	0.91851	15	33.4	0.83994	0.06232	0.9973	-0.1424	0.7379	0.7927	NA
950	Q14194	CRMP1	Dihydropyrimidi	sp|Q14194|DPYL1_HUMAN	11	28.1	0.1507691	0.51375	0.9984	-0.6232	0.51375	0.99836	9	20.3	NA	NA	NA	0.53052	NA	NA	NA
951	Q14195	DPYSL3	Dihydropyrimidi	sp|Q14195|DPYL3_HUMAN	24	63	0.1837758	0.57142	0.9984	-0.9973	0.57142	0.99836	18	47.5	NA	NA	NA	NA	NA	NA	NA
952	Q14203	DCTN1	Dynactin subuni	sp|Q14203|DCTN1_HUMA	5	5.5	NA	NA	NA	NA	NA	NA	17	17.3	-0.0801	NA	NA	NA	NA	NA	NA
953	Q14204	DYNC1H1	Cytoplasmic dyn	sp|Q14204|DYHC1_HUMA	47	13.9	-0.157343	0.69665	0.9991	-1.3382	0.00376	0.03699	93	25.8	0.00058	0.99885	0.9997	2.67447	######	######	2
954	Q14240	EIF4A2	Eukaryotic initia	sp|Q14240|IF4A2_HUMAN	13	31.4	0.4764216	NA	NA	NA	NA	NA	12	31.7	NA	NA	NA	NA	NA	NA	NA
955	Q14247	CTTN	Src substrate co	sp|Q14247|SRC8_HUMAN	10	27.6	-0.003551	0.99018	0.9991	0.3371	0.25249	0.44979	20	44.5	-0.3113	0.28929	0.9973	0.89346	0.0061	0.0147	1
956	Q14254	FLOT2	Flotillin-2	sp|Q14254|FLOT2_HUMAN	6	16.1	-0.626563	0.09252	0.9991	-0.1565	0.75285	0.86792	6	15.2	0.22975	0.51605	0.9973	-1.4449	0.0113	0.0245	4

957	Q14257	RCN2	Reticulocalbin-2	sp|Q14257|RCN2_HUMAN	9	36.6	-0.121049	0.55281	0.9984	0.3241	0.55281	0.99836	10	46.4	NA	NA	NA	NA	NA	NA	NA
958	Q14318	FKBP8	Peptidyl-prolyl c	sp|Q14318|FKBP8_HUMAN	5	17.2	0.2596829	0.50824	0.9984	0.1896	0.50824	0.99836	2	7.8	NA	NA	NA	NA	NA	NA	NA
959	Q14320	FAM50A	Protein FAM50A	sp|Q14320|FA50A_HUMAN	1	2.4	NA	NA	NA	NA	NA	NA	6	23	0.44304	0.1787	0.9991	0.59333	0.1787	0.9991	NA
960	Q14444	CAPRIN1	Caprin-1	sp|Q14444|CAPR1_HUMAN	12	23	-0.04543	0.85785	0.9991	0.333	0.20058	0.38435	12	19.9	-0.1281	0.61473	0.9973	1.60914	######	######	1
961	Q14498	RBM39	RNA-binding pro	sp|Q14498|RBM39_HUMA	5	12.1	-0.067702	0.82431	0.9991	0.4695	0.13772	0.30325	10	24.9	0.15805	0.56416	0.9973	0.16192	0.5548	0.6459	NA
962	Q14534	SQLE	Squalene monoo	sp|Q14534|ERG1_HUMAN	3	8.7	-0.256881	0.42736	0.9984	0.8294	0.42736	0.99836	NA	NA	NA	NA	NA	NA	NA	NA	NA
963	Q14566	MCM6	DNA replication	sp|Q14566|MCM6_HUMAN	18	29.4	0.0055739	0.979	0.9991	0.2853	0.18981	0.37279	17	24.8	0.0259	0.9027	0.9973	0.29309	0.1786	0.2595	NA
964	Q14671	PUM1	Pumilio homolog	sp|Q14671|PUM1_HUMAN	4	3.9	-0.117559	NA	NA	NA	NA	NA	4	5.1	0.06182	NA	NA	NA	NA	NA	NA
965	Q14677	CLINT1	Clathrin interact	sp|Q14677|EPN4_HUMAN	9	20	-0.001344	0.99636	0.9991	0.0382	0.89681	0.93506	8	17	-0.119	0.68655	0.9973	0.33594	0.2629	0.3465	NA
966	Q14683	SMC1A	Structural maint	sp|Q14683|SMC1A_HUMA	21	17.7	0.0437894	0.87133	0.9991	0.4306	0.12484	0.2883	16	13.4	-0.0036	0.98944	0.9973	-0.0969	0.7205	0.7823	NA
967	Q14684	RRP1B	Ribosomal RNA p	sp|Q14684|RRP1B_HUMAN	7	9.9	0.0776669	0.71362	0.9984	-0.2126	0.71362	0.99836	8	12.5	NA	NA	NA	NA	NA	NA	NA
968	Q14690	PDCD11	Protein RRP5 ho	sp|Q14690|RRP5_HUMAN	18	11.2	0.3171799	0.17179	0.9984	0.4636	0.17179	0.99836	8	4.8	NA	NA	NA	NA	NA	NA	NA
969	Q14692	BMS1	Ribosome bioger	sp|Q14692|BMS1_HUMAN	4	4.4	-0.309282	0.49201	0.9984	-0.3803	0.49201	0.99836	2	2.2	NA	NA	NA	NA	NA	NA	NA
970	Q14694	USP10	Ubiquitin carbox	sp|Q14694|UBP10_HUMAN	3	4.9	-0.302778	0.29629	0.9984	-0.487	0.29629	0.99836	7	13	NA	NA	NA	NA	NA	NA	NA
971	Q14696	MESD	LRP chaperone M	sp|Q14696|MESD_HUMAN	5	25.6	-0.32963	0.29691	0.9984	-0.4879	0.29691	0.99836	3	14.5	NA	NA	NA	NA	NA	NA	NA
972	Q14697	GANAB	Neutral alpha-gl	sp|Q14697|GANAB_HUMA	8	13	-0.154473	0.63691	0.9991	0.7547	0.05222	0.17799	17	23.1	-0.2234	0.49653	0.9973	1.38359	0.0006	0.002	1
973	Q14739	LBR	Delta(14)-sterol	sp|Q14739|LBR_HUMAN D	5	7.3	-0.341589	0.14151	0.9984	0.4408	0.14151	0.99836	2	3.1	NA	NA	NA	NA	NA	NA	NA
974	Q14839	CHD4	Chromodomain-h	sp|Q14839|CHD4_HUMAN	17	11.4	0.0177576	0.96415	0.9991	0.6906	0.09455	0.25391	21	13.8	0.27307	0.4927	0.9973	-0.3255	0.415	0.5082	NA
975	Q14847	LASP1	LIM and SH3 dor	sp|Q14847|LASP1_HUMAN	8	32.2	0.2254465	0.70308	0.9991	-0.4768	0.42381	0.61448	7	29.9	-0.0636	0.91416	0.9973	-1.4131	0.0269	0.0509	4
976	Q14914	PTGR1	Prostaglandin re	sp|Q14914|PTGR1_HUMAN	7	26.7	0.4531984	0.04262	0.9984	0.3568	0.04262	0.99836	1	7	NA	NA	NA	NA	NA	NA	NA
977	Q14974	KPNB1	Importin subunit	sp|Q14974|IMB1_HUMAN	8	14.7	-0.248329	0.39168	0.9991	-0.5874	0.05345	0.18047	23	38.6	-0.1485	0.60576	0.9973	-0.3921	0.1833	0.2642	NA
978	Q14978	NOLC1	Nucleolar and co	sp|Q14978|NOLC1_HUMAN	5	6.3	-0.314258	0.54481	0.9991	-0.0536	0.91731	0.95217	12	16.7	-1.021	0.03955	0.9973	3.92872	######	######	1
979	Q14980	NUMA1	Nuclear mitotic a	sp|Q14980|NUMA1_HUMA	14	8	-0.023764	0.93547	0.9991	0.1574	0.63291	0.77639	40	24.3	0.99616	0.02903	0.9973	2.96514	######	######	1
980	Q14C86	GAPVD1	GTPase-activatin	sp|Q14C86|GAPD1_HUMA	1	0.8	NA	NA	NA	NA	NA	NA	5	3.9	-0.0299	0.91749	0.9991	-0.1911	0.9175	0.9991	NA
981	Q15003	NCAPH	Condensin comp	sp|Q15003|CND2_HUMAN	6	9.9	-0.125225	0.6219	0.9984	0.6543	0.6219	0.99836	4	6.2	NA	NA	NA	NA	NA	NA	NA
982	Q15005	SPCS2	Signal peptidase	sp|Q15005|SPCS2_HUMAN	4	23.9	0.1429662	0.72925	0.9984	1.2624	0.72925	0.99836	2	12.4	NA	NA	NA	NA	NA	NA	NA
983	Q15019	Sep-02	Septin-2	sp|Q15019|SEPT2_HUMAN	7	20.2	0.1179827	0.68514	0.9991	-0.5047	0.09611	0.25715	12	47.1	-0.302	0.30614	0.9973	2.04035	######	######	2
984	Q15020	SART3	Squamous cell ca	sp|Q15020|SART3_HUMAN	3	5.2	0.3986332	0.19501	0.9984	0.6012	0.19501	0.99836	7	8.7	NA	NA	NA	NA	NA	NA	NA
985	Q15029	EFTUD2	116 kDa U5 sma	sp|Q15029|U5S1_HUMAN	14	18	0.1904116	0.46305	0.9991	0.4093	0.12539	0.28864	25	32.5	-0.2773	0.28961	0.9973	0.41757	0.1184	0.1806	NA
986	Q15046	KARS	Lysine--tRNA liga	sp|Q15046|SYK_HUMAN Ly	12	26.5	-0.113762	0.70708	0.9991	0.0695	0.81825	0.91051	22	40.2	0.43873	0.15925	0.9973	1.51951	######	0.0005	1
987	Q15050	RRS1	Ribosome bioger	sp|Q15050|RRS1_HUMAN	3	11.5	0.1169961	NA	NA	NA	NA	NA	3	14	NA	NA	NA	NA	NA	NA	NA
988	Q15056	EIF4H	Eukaryotic trans	sp|Q15056|IF4H_HUMAN E	5	29.8	0.4320008	0.48654	0.9991	-0.5794	0.50897	0.68136	8	46.8	0.63435	0.31163	0.9973	3.60983	######	0.0002	2
989	Q15075	EEA1	Early endosome	sp|Q15075|EEA1_HUMAN	5	4.2	NA	NA	NA	NA	NA	NA	10	8.1	0.0808	0.91926	0.9991	1.07434	0.9193	0.9991	NA
990	Q15084	PDIA6	Protein disulfide	sp|Q15084|PDIA6_HUMAN	9	27.7	-0.272313	0.1463	0.9984	0.2798	0.1463	0.99836	13	40.7	NA	NA	NA	3.10619	NA	NA	NA
991	Q15125	EBP	3-beta-hydroxyst	sp|Q15125|EBP_HUMAN 3-	1	5.2	NA	NA	NA	NA	NA	NA	1	5.2	NA	NA	NA	NA	NA	NA	NA
992	Q15181	PPA1	Inorganic pyroph	sp|Q15181|IPYR_HUMAN I	12	49.1	0.041155	0.80636	0.9991	-0.5068	0.01473	0.08033	14	61.6	-0.1074	0.52484	0.9973	0.64876	0.0013	0.0039	2
993	Q15185	PTGES3	Prostaglandin E	sp|Q15185|TEBP_HUMAN	4	28.8	-0.103485	0.70937	0.9991	-0.4113	0.30293	0.503	5	36.9	0.04419	0.87331	0.9973	1.41678	0.0001	0.0005	2
994	Q15233	NONO	Non-POU domain	sp|Q15233|NONO_HUMAN	18	44.8	0.2007395	0.38941	0.9991	0.3788	0.11423	0.27928	20	48.2	-0.0529	0.8185	0.9973	0.4118	0.0881	0.1435	NA
995	Q15257	PTPA	Serine/threonine	sp|Q15257|PTPA_HUMAN	3	18.2	0.461486	NA	NA	NA	NA	NA	2	7.3	NA	NA	NA	NA	NA	NA	NA
996	Q15286	RAB35	Ras-related prot	sp|Q15286|RAB35_HUMAN	2	11.9	-0.141464	0.65516	0.9984	-0.4691	0.65516	0.99836	2	12.9	NA	NA	NA	NA	NA	NA	NA
997	Q15287	RNPS1	RNA-binding pro	sp|Q15287|RNPS1_HUMAN	3	9.8	0.2009932	0.49497	0.9991	0.6681	0.05483	0.18165	4	13.8	0.22687	0.44208	0.9973	0.23102	0.4339	0.5259	NA
998	Q15293	RCN1	Reticulocalbin-1	sp|Q15293|RCN1_HUMAN	6	26.3	0.0808911	0.79868	0.9991	0.4996	0.12851	0.29391	11	49.2	-0.2035	0.5233	0.9973	-0.0005	0.9986	0.9986	NA
999	Q15363	TMED2	Transmembrane	sp|Q15363|TMED2_HUMA	4	24.9	0.0194197	0.94341	0.9991	0.365	0.1946	0.37903	4	22.9	-0.227	0.36032	0.9973	-0.2815	0.2601	0.3433	NA
1000	Q15365	PCBP1	Poly(rC)-binding	sp|Q15365|PCBP1_HUMAN	13	47.8	-0.316283	0.2127	0.9991	-0.5969	0.02602	0.11746	13	59.6	0.28973	0.25183	0.9973	0.91438	0.0017	0.005	2
1001	Q15366	PCBP2	Poly(rC)-binding	sp|Q15366|PCBP2_HUMAN	12	41.6	-0.651205	0.26454	0.9991	0.6534	0.42355	0.61448	10	40	-0.5257	0.36412	0.9973	1.98451	0.0032	0.0084	1
1002	Q15369	ELOC	Elongin-C	sp|Q15369|ELOC_HUMAN	3	32.1	0.5206093	0.05061	0.9991	-0.4552	0.20785	0.39404	3	41.1	0.03009	0.90358	0.9973	-0.1212	0.6269	0.7123	NA
1003	Q15370	ELOB	Elongin-B	sp|Q15370|ELOB_HUMAN	3	21.2	-0.159094	0.64673	0.9991	-0.1688	0.627	0.77546	6	37.3	-0.0116	0.97317	0.9973	-0.4086	0.2475	0.3323	NA
1004	Q15382	RHEB	GTP-binding prot	sp|Q15382|RHEB_HUMAN	2	13	0.111777	NA	NA	NA	NA	NA	2	13	-0.0215	NA	NA	NA	NA	NA	NA

1005	Q15392	DHCR24	Delta(24)-sterol	sp|Q15392|DHC24_HUMAN	4	10.3	-0.109577	0.60921	0.9984	0.1738	0.60921	0.99836	2	4.3	NA	NA	NA	NA	NA	NA	NA
1006	Q15393	SF3B3	Splicing factor 3	sp|Q15393|SF3B3_HUMAN	21	22.2	-0.108682	0.66295	0.9991	-0.2145	0.39376	0.60102	22	25.4	-0.0318	0.89837	0.9973	-0.782	0.0055	0.0136	4
1007	Q15417	CNN3	Calponin-3	sp|Q15417|CNN3_HUMAN	5	20.4	0.190301	0.41546	0.9991	0.0256	0.92113	0.9534	8	31.9	0.33176	0.16469	0.9973	0.31751	0.1825	0.2635	NA
1008	Q15424	SAFB	Scaffold attachm	sp|Q15424|SAFB1_HUMAN	8	14.4	0.4371981	0.16679	0.9984	0.3144	0.16679	0.99836	13	18.6	NA	NA	NA	NA	NA	NA	NA
1009	Q15427	SF3B4	Splicing factor 3	sp|Q15427|SF3B4_HUMAN	5	12.5	0.0473112	0.89329	0.9991	0.6384	0.08424	0.23606	6	19.3	0.15531	0.66058	0.9973	0.79006	0.0367	0.0669	1
1010	Q15437	SEC23B	Protein transpor	sp|Q15437|SC23B_HUMAN	3	4.6	NA	NA	NA	NA	NA	NA	8	13.4	0.10498	NA	NA	NA	NA	NA	NA
1011	Q15459	SF3A1	Splicing factor 3	sp|Q15459|SF3A1_HUMAN	20	31.5	0.0889171	0.70016	0.9991	0.3733	0.11898	0.2853	22	35.3	-0.0714	0.757	0.9973	-0.5247	0.0341	0.0622	5
1012	Q15631	TSN	Translin	sp|Q15631|TSN_HUMAN T	2	11.8	0.1457934	0.48629	0.9984	-0.2392	0.48629	0.99836	4	25	NA	NA	NA	NA	NA	NA	NA
1013	Q15637	SF1	Splicing factor 1	sp|Q15637|SF01_HUMAN	6	13.3	-0.311048	0.32159	0.9991	0.342	0.27727	0.47715	9	19.1	-0.2395	0.4426	0.9973	0.02509	0.9353	0.9446	NA
1014	Q15645	TRIP13	Pachytene check	sp|Q15645|PCH2_HUMAN	2	5.8	0.3757318	0.2585	0.9991	0.0731	0.84464	0.91848	8	17.8	0.24071	0.27652	0.9973	-0.0953	0.6593	0.7397	NA
1015	Q15651	HMGN3	High mobility gr	sp|Q15651|HMGN3_HUMA	2	20.2	0.4698831	0.19157	0.9984	0.5893	0.19157	0.99836	NA	NA	NA	NA	NA	NA	NA	NA	NA
1016	Q15691	MAPRE1	Microtubule-ass	sp|Q15691|MARE1_HUMA	6	23.9	0.0937037	0.6869	0.9991	-0.7599	0.00917	0.06248	10	53.7	0.31301	0.18971	0.9973	1.46073	######	######	2
1017	Q15717	ELAVL1	ELAV-like protei	sp|Q15717|ELAV1_HUMAN	8	34	-0.246069	0.25227	0.9991	0.3906	0.07752	0.22519	9	36.8	-0.2539	0.23805	0.9973	-0.3329	0.1275	0.1931	NA
1018	Q15738	NSDHL	Sterol-4-alpha-ca	sp|Q15738|NSDHL_HUMAN	5	16.4	0.0379774	0.90672	0.9987	0.7166	0.90672	0.99874	2	5.4	NA	NA	NA	NA	NA	NA	NA
1019	Q15758	SLC1A5	Neutral amino a	sp|Q15758|AAAT_HUMAN	6	13.5	0.2830417	0.32705	0.9984	1.1212	0.32705	0.99836	3	7.8	NA	NA	NA	NA	NA	NA	NA
1020	Q15785	TOMM34	Mitochondrial in	sp|Q15785|TOM34_HUMA	2	7.8	NA	NA	NA	NA	NA	NA	11	45.3	NA	NA	NA	2.88456	NA	NA	NA
1021	Q15819	UBE2V2	Ubiquitin-conjug	sp|Q15819|UB2V2_HUMAN	5	46.9	0.0170702	0.96079	0.9991	-0.8045	0.05201	0.17799	7	58.6	0.24661	0.48123	0.9973	0.18702	0.5919	0.6791	NA
1022	Q16181	Sep-07	Septin-7	sp|Q16181|SEPT7_HUMAN	7	16.5	0.0381557	0.88007	0.9991	-0.6078	0.02634	0.11746	9	22.4	0.21039	0.41028	0.9973	0.7149	0.0109	0.0238	2
1023	Q16186	ADRM1	Proteasomal ubi	sp|Q16186|ADRM1_HUMA	4	13.3	-0.004496	0.98672	0.999	0.3287	0.98672	0.99899	3	10.6	NA	NA	NA	NA	NA	NA	NA
1024	Q16204	CCDC6	Coiled-coil doma	sp|Q16204|CCDC6_HUMAN	3	8	0.5962828	NA	NA	NA	NA	NA	NA	NA	NA	NA	NA	NA	NA	NA	NA
1025	Q16531	DDB1	DNA damage-bin	sp|Q16531|DDB1_HUMAN	11	11	-0.204687	0.4591	0.9991	-0.5002	0.08205	0.23269	18	18.2	0.02549	0.92591	0.9973	0.22297	0.4207	0.5134	NA
1026	Q16543	CDC37	Hsp90 co-chaper	sp|Q16543|CDC37_HUMAN	10	28.6	0.0409187	0.85545	0.9991	-0.4045	0.0856	0.238	11	32	-0.0503	0.82282	0.9973	1.92146	######	######	2
1027	Q16555	DPYSL2	Dihydropyrimidi	sp|Q16555|DPYL2_HUMAN	18	47.7	0.0332001	0.91606	0.9991	-0.906	0.01922	0.09731	24	63.1	0.02724	0.93108	0.9973	3.9178	######	######	2
1028	Q16576	RBBP7	Histone-binding	sp|Q16576|RBBP7_HUMAN	13	34.8	-0.108989	0.69743	0.9991	-0.133	0.63556	0.7779	14	46.6	-0.0962	0.73144	0.9973	-2.3772	######	######	4
1029	Q16629	SRSF7	Serine/arginine-	sp|Q16629|SRSF7_HUMAN	3	17.6	0.1858831	0.55629	0.9991	1.0131	0.03544	0.13729	8	30.3	0.27418	0.38888	0.9973	1.99692	######	######	1
1030	Q16630	CPSF6	Cleavage and po	sp|Q16630|CPSF6_HUMAN	6	15.2	0.1151043	0.66831	0.9991	0.2032	0.50067	0.67575	6	17.1	0.11786	0.66091	0.9973	0.34729	0.2068	0.2937	NA
1031	Q16643	DBN1	Drebrin	sp|Q16643|DREB_HUMAN	14	30.4	-0.26456	0.34602	0.9991	0.1238	0.65581	0.79346	21	41.9	-0.0491	0.8592	0.9973	3.23017	######	######	1
1032	Q16658	FSCN1	Fascin	sp|Q16658|FSCN1_HUMAN	15	40.8	-0.316359	0.10572	0.9984	-1.1529	0.10572	0.99836	22	58.8	NA	NA	NA	NA	NA	NA	NA
1033	Q16718	NDUFA5	NADH dehydrog	sp|Q16718|NDUA5_HUMA	2	20.7	-0.19447	NA	NA	NA	NA	NA	3	43.1	NA	NA	NA	NA	NA	NA	NA
1034	Q16740	CLPP	ATP-dependent	sp|Q16740|CLPP_HUMAN	2	10.8	NA	NA	NA	NA	NA	NA	4	21.7	0.17711	0.52406	0.9991	-1.9302	0.5241	0.9991	NA
1035	Q16799	RTN1	Reticulon-1	sp|Q16799|RTN1_HUMAN	2	2.1	0.4116702	0.48428	0.9984	0.368	0.48428	0.99836	2	2.1	NA	NA	NA	NA	NA	NA	NA
1036	Q16822	PCK2	Phosphoenolpyr	sp|Q16822|PCKGM_HUMA	6	10.9	-0.070014	0.79917	0.9984	0.577	0.79917	0.99836	4	7.7	NA	NA	NA	NA	NA	NA	NA
1037	Q16836	HADH	Hydroxyacyl-coe	sp|Q16836|HCDH_HUMAN	4	17.8	-0.144904	0.61372	0.9984	0.646	0.61372	0.99836	5	25.8	NA	NA	NA	NA	NA	NA	NA
1038	Q16850	CYP51A1	Lanosterol 14-al	sp|Q16850|CP51A_HUMAN	7	15.9	-0.855577	0.24905	0.9984	0.1668	0.24905	0.99836	2	5.8	NA	NA	NA	NA	NA	NA	NA
1039	Q16851	UGP2	UTP--glucose-1-p	sp|Q16851|UGPA_HUMAN	26	56.5	0.0354249	0.92032	0.999	-1.1382	0.92032	0.99899	4	10.8	NA	NA	NA	NA	NA	NA	NA
1040	Q16891	IMMT	MICOS complex	sp|Q16891|MIC60_HUMAN	18	27.4	0.0625108	0.87268	0.9991	0.752	0.06749	0.20896	20	32.5	-0.3338	0.39732	0.9973	1.25751	0.0047	0.0117	1
1041	Q1KMD3	HNRNPUL2	Heterogeneous	sp|Q1KMD3|HNRL2_HUMA	11	16.7	0.1357958	0.66051	0.9991	1.0946	0.0023	0.02957	11	18.9	-0.0036	0.99069	0.9973	-0.6445	0.0493	0.0859	5
1042	Q29RF7	PDS5A	Sister chromatid	sp|Q29RF7|PDS5A_HUMAN	3	3.2	0.3923993	NA	NA	NA	NA	NA	6	6.3	NA	NA	NA	NA	NA	NA	NA
1043	Q2NL82	TSR1	Pre-rRNA-proces	sp|Q2NL82|TSR1_HUMAN	3	4.7	NA	NA	NA	NA	NA	NA	3	5	-0.2425	0.30927	0.9991	-1.1586	0.3093	0.9991	NA
1044	Q2Q1W2	TRIM71	E3 ubiquitin-pro	sp|Q2Q1W2|LIN41_HUMAN	5	5.6	-0.069658	0.81622	0.9984	0.3195	0.81622	0.99836	NA	NA	NA	NA	NA	NA	NA	NA	NA
1045	Q32MZ4	LRRFIP1	Leucine-rich repe	sp|Q32MZ4|LRRF1_HUMAN	8	11.4	0.1795928	0.56093	0.9991	-0.4907	0.1247	0.2883	11	16.8	-0.2973	0.39236	0.9973	-0.0505	0.8832	0.9075	NA
1046	Q4VC31	CCDC58	Coiled-coil doma	sp|Q4VC31|CCD58_HUMAN	2	16.7	-0.438771	0.4364	0.9991	-0.7148	0.21566	0.40558	4	34	-0.145	0.65268	0.9973	0.89062	0.0168	0.0348	2
1047	Q52LJ0	FAM98B	Protein FAM98B	sp|Q52LJ0|FA98B_HUMAN	3	13	0.2286627	0.22849	0.9984	0.0298	0.22849	0.99836	4	17.9	NA	NA	NA	NA	NA	NA	NA
1048	Q53GQ0	HSD17B12	Very-long-chain	sp|Q53GQ0|DHB12_HUMA	5	14.4	-0.297819	0.18312	0.9984	0.2663	0.18312	0.99836	NA	NA	NA	NA	NA	NA	NA	NA	NA
1049	Q53GS9	USP39	U4/U6.U5 tri-snR	sp|Q53GS9|SNUT2_HUMA	2	6	NA	NA	NA	NA	NA	NA	4	9.9	0.20854	0.43849	0.9991	0.74611	0.4385	0.9991	NA
1050	Q58FF8	HSP90AB2P	Putative heat sh	sp|Q58FF8|H90B2_HUMAN	11	22.8	0.7224213	0.21179	0.9991	-0.5207	0.41385	0.60623	9	21.8	0.66359	0.24952	0.9973	1.57174	0.0123	0.0265	2
1051	Q5BKZ1	ZNF326	DBIRD complex s	sp|Q5BKZ1|ZN326_HUMAN	4	11.7	0.1692036	NA	NA	NA	NA	NA	4	7.7	NA	NA	NA	NA	NA	NA	NA
1052	Q5H9R7	PPP6R3	Serine/threonine	sp|Q5H9R7|PP6R3_HUMAN	4	5.6	0.1521736	0.55916	0.9984	-0.0968	0.55916	0.99836	6	9.3	-0.3465	NA	NA	NA	NA	NA	NA

1053	Q5JSH3	WDR44	WD repeat-cont	sp|Q5JSH3|WDR44_HUMA	6	8.9	-0.388867	0.25647	0.9984	-0.5473	0.25647	0.99836	1	1.9	NA	NA	NA	NA	NA	NA	NA
1054	Q5JTH9	RRP12	RRP12-like prote	sp|Q5JTH9|RRP12_HUMAN	2	2.1	NA	NA	NA	NA	NA	NA	10	10.1	0.71444	0.07415	0.9991	0.03573	0.0741	0.9991	NA
1055	Q5T0U0	CCDC122	Coiled-coil doma	sp|Q5T0U0|CC122_HUMAN	2	7	-1.109108	0.00795	0.9984	-0.1213	0.00795	0.99836	NA	NA	NA	NA	NA	NA	NA	NA	NA
1056	Q5T4S7	UBR4	E3 ubiquitin-pro	sp|Q5T4S7|UBR4_HUMAN	7	1.5	0.0624791	0.81489	0.9984	-0.0707	0.81489	0.99836	7	1.8	NA	NA	NA	NA	NA	NA	NA
1057	Q5T7N2	L1TD1	LINE-1 type tran	sp|Q5T7N2|LITD1_HUMAN	41	45	-0.098373	0.51776	0.9984	0.6554	0.51776	0.99836	NA	NA	NA	NA	NA	NA	NA	NA	NA
1058	Q5TFE4	NT5DC1	5-nucleotidase d	sp|Q5TFE4|NT5D1_HUMAN	5	16.5	-0.181305	0.57274	0.9984	-0.8355	0.57274	0.99836	2	4	NA	NA	NA	NA	NA	NA	NA
1059	Q5UIP0	RIF1	Telomere-associ	sp|Q5UIP0|RIF1_HUMAN T	19	11	0.148608	0.61416	0.9984	0.1333	0.61416	0.99836	5	2.6	NA	NA	NA	NA	NA	NA	NA
1060	Q5VYK3	ECPAS	Proteasome ada	sp|Q5VYK3|ECM29_HUMA	13	8.5	-0.274595	0.26251	0.9984	-0.3365	0.26251	0.99836	17	12.1	0.01922	NA	NA	NA	NA	NA	NA
1061	Q6DD88	ATL3	Atlastin-3	sp|Q6DD88|ATLA3_HUMA	1	3.5	NA	NA	NA	NA	NA	NA	4	11.1	0.25124	0.359	0.9991	-1.5202	0.359	0.9991	NA
1062	Q6FI81	CIAPIN1	Anamorsin	sp|Q6FI81|CPIN1_HUMAN	4	15.4	0.3834536	0.40764	0.9984	-0.4394	0.40764	0.99836	8	37.2	NA	NA	NA	NA	NA	NA	NA
1063	Q6IAA8	LAMTOR1	Ragulator compl	sp|Q6IAA8|LTOR1_HUMAN	3	29.8	-0.253438	0.25051	0.9984	0.2047	0.25051	0.99836	3	31.1	NA	NA	NA	NA	NA	NA	NA
1064	Q6IN85	PPP4R3A	Serine/threonin	sp|Q6IN85|P4R3A_HUMAN	5	6.6	0.0707004	NA	NA	NA	NA	NA	3	3.8	NA	NA	NA	NA	NA	NA	NA
1065	Q6NVY1	HIBCH	3-hydroxyisobut	sp|Q6NVY1|HIBCH_HUMAN	7	21.2	0.084149	0.70018	0.9984	0.4201	0.70018	0.99836	4	12.2	NA	NA	NA	NA	NA	NA	NA
1066	Q6P2Q9	PRPF8	Pre-mRNA-proce	sp|Q6P2Q9|PRP8_HUMAN	25	13.1	-0.050321	0.75073	0.9991	0.1503	0.34869	0.54937	31	18.5	0.03025	0.8484	0.9973	0.00093	0.9953	0.9981	NA
1067	Q6P474	PDXDC2P	Putative pyridox	sp|Q6P474|PDXD2_HUMAN	1	1	NA	NA	NA	NA	NA	NA	1	2.8	NA	NA	NA	NA	NA	NA	NA
1068	Q6PKG0	LARP1	La-related prote	sp|Q6PKG0|LARP1_HUMAN	8	6.8	-0.614685	0.06064	0.9991	-0.4292	0.17813	0.35476	12	16.3	-0.4564	0.15362	0.9973	-0.4224	0.1847	0.2657	NA
1069	Q6UB35	MTHFD1L	Monofunctional	sp|Q6UB35|C1TM_HUMAN	7	9.6	0.0626317	0.77847	0.9984	0.1376	0.77847	0.99836	4	4.7	-0.0011	NA	NA	NA	NA	NA	NA
1070	Q6UN15	FIP1L1	Pre-mRNA 3-end	sp|Q6UN15|FIP1_HUMAN	3	8.8	NA	NA	NA	NA	NA	NA	4	8.8	0.12923	0.67659	0.9991	0.51143	0.6766	0.9991	NA
1071	Q6UW68	TMEM205	Transmembrane	sp|Q6UW68|TM205_HUMA	3	22.2	-0.226647	0.53693	0.9984	-0.0239	0.53693	0.99836	2	15.9	NA	NA	NA	NA	NA	NA	NA
1072	Q71DI3	HIST2H3A	Histone H3.2	sp|Q71DI3|H32_HUMAN H	8	53.7	0.2881617	0.54107	0.9991	0.7242	0.1358	0.30186	5	42.6	0.35821	0.44882	0.9973	1.96513	0.0006	0.002	1
1073	Q71UM5	RPS27L	40S ribosomal p	sp|Q71UM5|RS27L_HUMA	2	28.6	NA	NA	NA	NA	NA	NA	3	33.3	0.27746	0.62037	0.9991	-0.3171	0.6204	0.9991	NA
1074	Q7KZ85	SUPT6H	Transcription el	sp|Q7KZ85|SPT6H_HUMAN	2	2.1	NA	NA	NA	NA	NA	NA	7	5.7	0.06902	NA	NA	NA	NA	NA	NA
1075	Q7KZF4	SND1	Staphylococcal n	sp|Q7KZF4|SND1_HUMAN	24	31.1	0.0494389	0.826	0.9991	-0.3213	0.16551	0.34014	32	44.2	0.03156	0.88834	0.9973	-0.8389	0.0016	0.0046	4
1076	Q7L014	DDX46	Probable ATP-de	sp|Q7L014|DDX46_HUMAN	6	7.1	-0.11332	NA	NA	NA	NA	NA	13	14.5	0.47819	0.06432	0.9991	0.89742	0.0643	0.9991	NA
1077	Q7L0Y3	TRMT10C	tRNA methyltra	sp|Q7L0Y3|TM10C_HUMAN	4	10.2	NA	NA	NA	1.2248	NA	NA	2	5.5	NA	NA	NA	NA	NA	NA	NA
1078	Q7L190	DPPA4	Developmental	sp|Q7L190|DPPA4_HUMAN	2	12.5	-0.194929	0.70624	0.9984	0.0902	0.70624	0.99836	NA	NA	NA	NA	NA	NA	NA	NA	NA
1079	Q7L1Q6	BZW1	Basic leucine zip	sp|Q7L1Q6|BZW1_HUMAN	8	17.2	-0.012326	0.96086	0.9991	-0.6893	0.02444	0.11253	11	27.4	-0.4436	0.09233	0.9973	-1.0128	0.0009	0.003	3
1080	Q7L2H7	EIF3M	Eukaryotic trans	sp|Q7L2H7|EIF3M_HUMAN	4	14.7	-0.169253	0.52545	0.9991	-0.2162	0.56584	0.72534	15	54.3	-0.0272	0.91808	0.9973	-1.454	######	0.0003	4
1081	Q7L311	ARMCX2	Armadillo repea	sp|Q7L311|ARMX2_HUMA	3	17.1	-0.119327	NA	NA	NA	NA	NA	NA	NA	NA	NA	NA	NA	NA	NA	NA
1082	Q9H8S9	MOB1A	MOB kinase acti	sp|Q9H8S9|MOB1A_HUMA	2	11.6	0.101086	NA	NA	NA	NA	NA	3	16.7	0.20128	NA	NA	NA	NA	NA	NA
1083	Q7Z2W4	ZC3HAV1	Zinc finger CCCH	sp|Q7Z2W4|ZCCHV_HUMA	2	3	NA	NA	NA	-0.1164	NA	NA	5	8.4	NA	NA	NA	NA	NA	NA	NA
1084	Q7Z3K3	POGZ	Pogo transposab	sp|Q7Z3K3|POGZ_HUMAN	4	3.6	0.0933125	0.79243	0.9984	0.6792	0.79243	0.99836	3	3.4	NA	NA	NA	NA	NA	NA	NA
1085	Q7Z417	NUFIP2	Nuclear fragile X	sp|Q7Z417|NUFP2_HUMAN	5	9.5	NA	NA	NA	NA	NA	NA	8	17.6	0.12099	NA	NA	NA	NA	NA	NA
1086	Q7Z4V5	HDGFL2	Hepatoma-deriv	sp|Q7Z4V5|HDGR2_HUMA	2	4.8	0.3222367	0.40206	0.9984	0.4464	0.40206	0.99836	4	7.3	NA	NA	NA	NA	NA	NA	NA
1087	Q7Z6Z7	HUWE1	E3 ubiquitin-pro	sp|Q7Z6Z7|HUWE1_HUMA	8	2.1	NA	NA	NA	NA	NA	NA	6	1.9	-0.1802	NA	NA	NA	NA	NA	NA
1088	Q7Z739	YTHDF3	YTH domain-con	sp|Q7Z739|YTHD3_HUMAN	5	9.6	NA	NA	NA	NA	NA	NA	10	20.2	0.3495	0.2258	0.9991	0.49999	0.2258	0.9991	NA
1089	Q7Z7K6	CENPV	Centromere pro	sp|Q7Z7K6|CENPV_HUMAN	3	14.9	0.2477644	0.39442	0.9984	0.3781	0.39442	0.99836	1	4.7	NA	NA	NA	NA	NA	NA	NA
1090	Q86U42	PABPN1	Polyadenylate-b	sp|Q86U42|PABP2_HUMAN	6	24.8	-0.003382	0.9918	0.9991	0.8793	0.01509	0.08154	6	27.1	-0.079	0.81039	0.9973	-0.0462	0.8882	0.908	5
1091	Q86UP2	KTN1	Kinectin	sp|Q86UP2|KTN1_HUMAN	10	8	0.2342649	0.89203	0.9991	0.1875	0.91349	0.94965	43	37.4	-0.2824	0.80016	0.9973	2.07569	0.0866	0.1424	NA
1092	Q86UV5	USP48	Ubiquitin carbox	sp|Q86UV5|UBP48_HUMA	2	2.8	-0.091231	NA	NA	NA	NA	NA	3	4	NA	NA	NA	NA	NA	NA	NA
1093	Q86V81	ALYREF	THO complex sul	sp|Q86V81|THOC4_HUMA	6	26.5	-0.225128	0.47662	0.9991	-0.0084	0.97859	0.98415	9	43.2	0.14426	0.6468	0.9973	0.36152	0.2588	0.343	NA
1094	Q86VP6	CAND1	Cullin-associated	sp|Q86VP6|CAND1_HUMA	18	18	0.643557	0.01657	0.9991	-0.6841	0.0117	0.07028	24	27.9	-0.1026	0.67587	0.9973	-0.1706	0.4891	0.5797	3
1095	Q86X55	CARM1	Histone-arginine	sp|Q86X55|CARM1_HUMA	5	9.7	-0.312755	0.25195	0.9991	-0.0338	0.92848	0.9536	5	9.9	0.2118	0.43163	0.9973	-0.5176	0.0991	0.1576	NA
1096	Q86XP3	DDX42	ATP-dependent	sp|Q86XP3|DDX42_HUMAN	10	13.1	-0.069757	0.67891	0.9984	-0.0322	0.67891	0.99836	12	15.2	0.17225	NA	NA	NA	NA	NA	NA
1097	Q86YP4	GATAD2A	Transcriptional r	sp|Q86YP4|P66A_HUMAN	4	6.8	0.5827001	0.26955	0.9984	-0.3423	0.26955	0.99836	NA	NA	NA	NA	NA	NA	NA	NA	NA
1098	Q8IVW6	ARID3B	AT-rich interacti	sp|Q8IVW6|ARI3B_HUMAN	3	7.3	NA	NA	NA	-0.0488	NA	NA	NA	NA	NA	NA	NA	NA	NA	NA	NA
1099	Q8IWA0	WDR75	WD repeat-cont	sp|Q8IWA0|WDR75_HUMA	3	5.1	-0.041325	0.91968	0.999	1.3029	0.91968	0.99899	4	7.5	NA	NA	NA	NA	NA	NA	NA
1100	Q8IWS0	PHF6	PHD finger prote	sp|Q8IWS0|PHF6_HUMAN	4	15.1	0.1944294	0.43792	0.9984	0.3682	0.43792	0.99836	3	7.7	NA	NA	NA	NA	NA	NA	NA

1101	Q8IX12	CCAR1	Cell division cycl	sp|Q8IX12|CCAR1_HUMAN	8	9	-0.278869	0.4132	0.9984	0.8446	0.4132	0.99836	5	5.1	NA	NA	NA	NA	NA	NA	NA
1102	Q8IY81	FTSJ3	pre-rRNA 2-O-rib	sp|Q8IY81|SPB1_HUMAN p	4	6	-0.61926	0.30665	0.9991	0.1286	0.82762	0.91541	7	12.6	-0.0201	0.98245	0.9973	0.30049	0.6884	0.7602	NA
1103	Q8IYB3	SRRM1	Serine/arginine	sp|Q8IYB3|SRRM1_HUMAN	2	2.8	0.413414	0.67421	0.9984	0.8774	0.67421	0.99836	4	7.9	NA	NA	NA	NA	NA	NA	NA
1104	Q8IZL8	PELP1	Proline-, glutami	sp|Q8IZL8|PELP1_HUMAN	1	2.2	NA	NA	NA	NA	NA	NA	NA	NA	NA	NA	NA	NA	NA	NA	NA
1105	Q8N0W4	NLGN4X	Neuroligin-4, X-l	sp|Q8N0W4|NLGNX_HUMA	3	5.6	-0.244938	0.45613	0.9984	0.5045	0.45613	0.99836	NA	NA	NA	NA	NA	NA	NA	NA	NA
1106	Q8N163	CCAR2	Cell cycle and ap	sp|Q8N163|CCAR2_HUMAN	18	26.2	0.0499372	0.87706	0.9991	1.1052	0.00352	0.03677	16	24.5	1.084	0.01416	0.9973	4.04964	######	######	1
1107	Q8N1F7	NUP93	Nuclear pore cor	sp|Q8N1F7|NUP93_HUMA	11	15.5	-0.183647	0.44992	0.9991	0.1757	0.46948	0.64919	15	23.4	-0.5641	0.02993	0.9973	-0.0104	0.9655	0.9723	5
1108	Q8N1G4	LRRC47	Leucine-rich repe	sp|Q8N1G4|LRC47_HUMAN	8	17.7	0.0879878	0.69502	0.9984	-0.4532	0.69502	0.99836	10	22.3	NA	NA	NA	NA	NA	NA	NA
1109	Q8N474	SFRP1	Secreted frizzled	sp|Q8N474|SFRP1_HUMAN	1	5.1	NA	NA	NA	NA	NA	NA	NA	NA	NA	NA	NA	NA	NA	NA	NA
1110	Q8N5K1	CISD2	CDGSH iron-sulfu	sp|Q8N5K1|CISD2_HUMAN	3	22.2	0.3093172	0.53815	0.9991	1.4579	0.01651	0.08736	5	41.5	0.38267	0.39737	0.9973	0.60725	0.1874	0.269	1
1111	Q8N7H5	PAF1	RNA polymerase	sp|Q8N7H5|PAF1_HUMAN	4	9.4	NA	NA	NA	NA	NA	NA	7	16.8	NA	NA	NA	0.57411	NA	NA	NA
1112	Q8N8S7	ENAH	Protein enabled	sp|Q8N8S7|ENAH_HUMAN	6	13.5	0.3950243	0.22414	0.9984	-0.0095	0.22414	0.99836	4	6.3	NA	NA	NA	NA	NA	NA	NA
1113	Q8NBS9	TXNDC5	Thioredoxin dom	sp|Q8NBS9|TXND5_HUMA	2	7.2	-0.302779	NA	NA	NA	NA	NA	15	39.6	NA	NA	NA	3.99162	NA	NA	NA
1114	Q8NC51	SERBP1	Plasminogen act	sp|Q8NC51|PAIRB_HUMAN	14	42.6	0.1647344	0.60423	0.9991	-0.3333	0.30045	0.50005	20	55.4	0.09489	0.76464	0.9973	0.80273	0.0201	0.0404	2
1115	Q8NE71	ABCF1	ATP-binding cass	sp|Q8NE71|ABCF1_HUMAN	13	20.1	0.6389884	0.05272	0.9984	0.2193	0.05272	0.99836	10	15	NA	NA	NA	NA	NA	NA	NA
1116	Q8NF37	LPCAT1	Lysophosphatidy	sp|Q8NF37|PCAT1_HUMAN	3	7.1	0.068342	0.73909	0.9984	0.4875	0.73909	0.99836	3	6.2	NA	NA	NA	NA	NA	NA	NA
1117	Q8NFW8	CMAS	N-acylneuramina	sp|Q8NFW8|NEUA_HUMA	3	6.2	NA	NA	NA	NA	NA	NA	3	8.1	-0.1432	NA	NA	NA	NA	NA	NA
1118	Q8NI22	MCFD2	Multiple coagula	sp|Q8NI22|MCFD2_HUMA	3	44.5	-0.054158	NA	NA	NA	NA	NA	3	44.5	NA	NA	NA	NA	NA	NA	NA
1119	Q8NI27	THOC2	THO complex sul	sp|Q8NI27|THOC2_HUMAN	2	1.6	NA	NA	NA	NA	NA	NA	2	1.3	0.42158	NA	NA	NA	NA	NA	NA
1120	Q8NI36	WDR36	WD repeat-conta	sp|Q8NI36|WDR36_HUMA	5	5.4	0.220614	0.49932	0.9984	0.5288	0.49932	0.99836	4	4.5	NA	NA	NA	NA	NA	NA	NA
1121	Q8TAT6	NPLOC4	Nuclear protein	sp|Q8TAT6|NPL4_HUMAN	2	3.9	NA	NA	NA	0.3596	NA	NA	6	12.3	-0.1255	NA	NA	NA	NA	NA	NA
1122	Q8TC07	TBC1D15	TBC1 domain fan	sp|Q8TC07|TBC15_HUMAN	2	3.9	NA	NA	NA	NA	NA	NA	5	7.5	0.06893	NA	NA	NA	NA	NA	NA
1123	Q8TC12	RDH11	Retinol dehydro	sp|Q8TC12|RDH11_HUMAN	5	22.3	0.1446656	0.55879	0.9984	0.122	0.55879	0.99836	NA	NA	NA	NA	NA	NA	NA	NA	NA
1124	Q8TCJ2	STT3B	Dolichyl-diphosp	sp|Q8TCJ2|STT3B_HUMAN	4	4.6	0.4331901	0.09034	0.9984	0.5084	0.09034	0.99836	1	1.1	NA	NA	NA	NA	NA	NA	NA
1125	Q8TCS8	PNPT1	Polyribonucleoti	sp|Q8TCS8|PNPT1_HUMAN	5	8.6	0.2143271	0.4911	0.9984	0.7947	0.4911	0.99836	4	6.9	0.23367	NA	NA	NA	NA	NA	NA
1126	Q8TCT9	HM13	Minor histocomp	sp|Q8TCT9|HM13_HUMAN	7	19.4	0.0727457	0.80008	0.9991	0.6487	0.03688	0.14131	6	17.5	0.23668	0.46494	0.9973	0.38265	0.2444	0.3295	1
1127	Q8TEM1	NUP210	Nuclear pore me	sp|Q8TEM1|PO210_HUMA	5	3.4	0.1959572	0.4819	0.9984	0.7993	0.4819	0.99836	NA	NA	NA	NA	NA	NA	NA	NA	NA
1128	Q8TEQ6	GEMIN5	Gem-associated	sp|Q8TEQ6|GEMI5_HUMA	5	6.2	-0.433896	0.1617	0.9984	0.1696	0.1617	0.99836	6	5.2	NA	NA	NA	NA	NA	NA	NA
1129	Q8WUM0	NUP133	Nuclear pore cor	sp|Q8WUM0|NU133_HUM	7	6.5	-0.088616	0.71686	0.9984	0.43	0.71686	0.99836	3	3.3	NA	NA	NA	NA	NA	NA	NA
1130	Q8WUM4	PDCD6IP	Programmed cel	sp|Q8WUM4|PDC6I_HUMA	8	9.6	0.4035597	0.1308	0.9991	-0.3902	0.29127	0.49286	16	21.4	-0.1346	0.60126	0.9973	-0.3216	0.2219	0.306	NA
1131	Q8WVJ2	NUDCD2	NudC domain-co	sp|Q8WVJ2|NUDC2_HUMA	2	17.8	0.3624544	NA	NA	NA	NA	NA	4	36.3	0.26563	0.38339	0.9991	-0.8673	0.3834	0.9991	NA
1132	Q8WVM8	SCFD1	Sec1 family dom	sp|Q8WVM8|SCFD1_HUM	3	7.6	0.1023887	0.80343	0.9984	0.1288	0.80343	0.99836	4	10.4	NA	NA	NA	NA	NA	NA	NA
1133	Q8WVV9	HNRNPLL	Heterogeneous	sp|Q8WVV9|HNRLL_HUMA	1	3.3	NA	NA	NA	NA	NA	NA	7	18.8	0.0984	NA	NA	NA	NA	NA	NA
1134	Q8WVX9	FAR1	Fatty acyl-CoA r	sp|Q8WVX9|FACR1_HUMA	5	12.8	0.0030047	0.99316	0.999	0.317	0.99316	0.99899	4	10.3	0.09668	NA	NA	NA	NA	NA	NA
1135	Q8WW12	PCNP	PEST proteolytic	sp|Q8WW12|PCNP_HUMA	6	48.3	0.3034876	0.18293	0.9984	0.1028	0.18293	0.99836	6	36	-0.0581	NA	NA	NA	NA	NA	NA
1136	Q8WWM7	ATXN2L	Ataxin-2-like pro	sp|Q8WWM7|ATX2L_HUM	9	10	0.0093924	0.9651	0.9991	0.3179	0.15181	0.32517	8	9.6	-0.0366	0.86454	0.9973	-0.5582	0.0176	0.036	5
1137	Q8WWY3	PRPF31	U4/U6 small nuc	sp|Q8WWY3|PRP31_HUMA	3	6.8	0.4517631	0.27998	0.9984	0.8487	0.27998	0.99836	5	11.6	-0.7102	NA	NA	NA	NA	NA	NA
1138	Q8WXF1	PSPC1	Paraspeckle com	sp|Q8WXF1|PSPC1_HUMA	5	11.7	0.2225453	0.51745	0.9984	0.1346	0.51745	0.99836	7	18.5	NA	NA	NA	NA	NA	NA	NA
1139	Q8WY07	SLC7A3	Cationic amino a	sp|Q8WY07|CTR3_HUMAN	3	6.8	-0.006525	0.97136	0.999	0.2926	0.97136	0.99899	NA	NA	NA	NA	NA	NA	NA	NA	NA
1140	Q92499	DDX1	ATP-dependent	sp|Q92499|DDX1_HUMAN	15	25.1	-0.066794	0.721	0.9991	-0.3711	0.06021	0.19493	21	37.2	0.37503	0.05782	0.9973	0.22006	0.2483	0.3328	NA
1141	Q92520	FAM3C	Protein FAM3C	sp|Q92520|FAM3C_HUMA	2	11.9	0.6761153	0.14658	0.9984	1.2269	0.14658	0.99836	3	16.3	NA	NA	NA	NA	NA	NA	NA
1142	Q92522	H1FX	Histone H1x	sp|Q92522|H1X_HUMAN H	2	14.1	-0.08079	0.82676	0.9984	0.6639	0.82676	0.99836	2	12.7	NA	NA	NA	NA	NA	NA	NA
1143	Q92598	HSPH1	Heat shock prot	sp|Q92598|HS105_HUMAN	26	39.5	0.2663297	0.20369	0.9991	-0.6171	0.00722	0.05274	29	41.6	0.0261	0.89834	0.9973	0.07323	0.7205	0.7823	3
1144	Q92616	GCN1	eIF-2-alpha kina	sp|Q92616|GCN1_HUMAN	8	3.7	0.1785148	0.6232	0.9991	0.5194	0.25377	0.45093	39	20.2	-0.1797	0.48647	0.9973	1.66716	######	0.0001	1
1145	Q92621	NUP205	Nuclear pore cor	sp|Q92621|NU205_HUMAN	8	4.4	0.4819365	0.32566	0.9984	0.9978	0.32566	0.99836	8	5.2	0.24984	NA	NA	NA	NA	NA	NA
1146	Q92667	AKAP1	A-kinase anchor	sp|Q92667|AKAP1_HUMAN	4	6.5	0.1130997	0.64627	0.9984	0.785	0.64627	0.99836	2	3.7	NA	NA	NA	NA	NA	NA	NA
1147	Q92734	TFG	Protein TFG	sp|Q92734|TFG_HUMAN P	4	20	-0.324989	0.19486	0.9991	-1.347	0.00014	0.00831	5	22	-0.0051	0.98325	0.9973	0.25494	0.3041	0.3891	3
1148	Q92769	HDAC2	Histone deacety	sp|Q92769|HDAC2_HUMA	7	15	0.0616423	0.80384	0.9984	0.6369	0.80384	0.99836	5	9.4	NA	NA	NA	NA	NA	NA	NA

1149	Q92804	TAF15	TATA-binding pr	sp|Q92804|RBP56_HUMAN	4	7.3	0.7167508	0.05342	0.9984	1.2236	0.05342	0.99836	7	17.6	NA	NA	NA	NA	NA	NA	NA
1150	Q92833	JARID2	Protein Jumonji	sp|Q92833|JARD2_HUMAN	2	2.5	-0.390013	NA	NA	NA	NA	NA	NA	NA	NA	NA	NA	NA	NA	NA	NA
1151	Q92841	DDX17	Probable ATP-de	sp|Q92841|DDX17_HUMAI	15	21.5	0.139857	0.42767	0.9991	0.2909	0.10959	0.27869	24	36.1	0.17198	0.33177	0.9973	1.61606	######	######	1
1152	Q92878	RAD50	DNA repair prot	sp|Q92878|RAD50_HUMAI	7	5.6	0.1067855	0.67441	0.9984	0.3567	0.67441	0.99836	3	2.7	NA	NA	NA	NA	NA	NA	NA
1153	Q92879	CELF1	CUGBP Elav-like	sp|Q92879|CELF1_HUMAN	4	11.5	-0.200492	0.43096	0.9984	0.1338	0.43096	0.99836	2	6.2	NA	NA	NA	NA	NA	NA	NA
1154	Q92900	UPF1	Regulator of nor	sp|Q92900|RENT1_HUMAN	9	10.2	-0.007291	0.97217	0.9991	-0.3092	0.30468	0.50354	20	21.3	0.14428	0.49358	0.9973	-0.2418	0.2582	0.3428	NA
1155	Q92905	COPS5	COP9 signalosom	sp|Q92905|CSN5_HUMAN	3	12.3	0.2638797	NA	NA	NA	NA	NA	7	24.9	-0.087	0.66528	0.9991	-0.6863	0.6653	0.9991	NA
1156	Q92922	SMARCC1	SWI/SNF comple	sp|Q92922|SMRC1_HUMA	13	15.3	0.1754525	0.39533	0.9984	0.4633	0.39533	0.99836	11	13	NA	NA	NA	NA	NA	NA	NA
1157	Q92945	KHSRP	Far upstream el	sp|Q92945|FUBP2_HUMAN	14	26.7	-0.016496	0.94759	0.9991	-0.0365	0.88448	0.9344	24	42.5	-0.217	0.3925	0.9973	1.19541	0.0002	0.0007	1
1158	Q92973	TNPO1	Transportin-1	sp|Q92973|TNPO1_HUMAI	7	9.1	0.0716112	0.74821	0.9991	0.0792	0.75086	0.86792	16	23.6	0.06506	0.77048	0.9973	0.473	0.0471	0.0824	1
1159	Q92990	GLMN	Glomulin	sp|Q92990|GLMN_HUMAN	2	3.5	0.286584	NA	NA	NA	NA	NA	NA	NA	NA	NA	NA	NA	NA	NA	NA
1160	Q93008	USP9X	Probable ubiquit	sp|Q93008|USP9X_HUMAN	11	5.8	0.3550563	0.1272	0.9984	-0.112	0.1272	0.99836	5	2.8	NA	NA	NA	NA	NA	NA	NA
1161	Q93009	USP7	Ubiquitin carbox	sp|Q93009|UBP7_HUMAN	5	5.4	-0.156501	0.63886	0.9991	0.2011	0.62273	0.77459	10	10.3	-0.1286	0.75192	0.9973	-0.1292	0.6978	0.7683	NA
1162	Q969G3	SMARCE1	SWI/SNF-related	sp|Q969G3|SMCE1_HUMA	7	17.5	-0.45758	0.2926	0.9984	0.8651	0.2926	0.99836	5	14.4	NA	NA	NA	NA	NA	NA	NA
1163	Q969H8	MYDGF	Myeloid-derived	sp|Q969H8|MYDGF_HUMA	2	13.9	NA	NA	NA	0.9232	NA	NA	2	12.1	NA	NA	NA	NA	NA	NA	NA
1164	Q969T9	WBP2	WW domain-bind	sp|Q969T9|WBP2_HUMAN	3	24.5	NA	NA	NA	NA	NA	NA	2	19.2	-0.327	NA	NA	NA	NA	NA	NA
1165	Q969X6	UTP4	U3 small nucleol	sp|Q969X6|UTP4_HUMAN	3	5	-1.206601	0.01016	0.9984	0.4182	0.01016	0.99836	3	5.5	NA	NA	NA	NA	NA	NA	NA
1166	Q969Z0	TBRG4	FAST kinase dor	sp|Q969Z0|FAKD4_HUMAI	4	6.7	-0.360156	0.28143	0.9984	0.1112	0.28143	0.99836	8	19.3	NA	NA	NA	NA	NA	NA	NA
1167	Q96A26	FAM162A	Protein FAM162	sp|Q96A26|F162A_HUMAI	3	18.8	0.0321062	0.91818	0.999	0.701	0.91818	0.99899	2	18.2	NA	NA	NA	NA	NA	NA	NA
1168	Q96A33	CCDC47	Coiled-coil doma	sp|Q96A33|CCD47_HUMAI	6	14.5	NA	NA	NA	NA	NA	NA	3	9.3	0.22936	0.43575	0.9991	-0.1034	0.4357	0.9991	NA
1169	Q96A49	SYAP1	Synapse-associat	sp|Q96A49|SYAP1_HUMAI	3	9.7	0.053723	0.88291	0.9984	0.5215	0.88291	0.99836	3	10.5	NA	NA	NA	NA	NA	NA	NA
1170	P61326	MAGOH	Protein mago na	sp|P61326|MGN_HUMAN	7	37	0.0270424	0.92053	0.9991	0.5472	0.05675	0.18627	7	43.8	0.14221	0.60134	0.9973	0.1082	0.6905	0.7613	NA
1171	Q96AC1	FERMT2	Fermitin family	sp|Q96AC1|FERM2_HUMA	6	11.6	NA	NA	NA	NA	NA	NA	8	15.6	0.14268	0.59943	0.9991	0.29353	0.5994	0.9991	NA
1172	Q96AE4	FUBP1	Far upstream el	sp|Q96AE4|FUBP1_HUMAI	15	26.9	-0.451096	0.23421	0.9991	-0.5185	0.17537	0.35323	23	41.3	0.23319	0.71526	0.9973	4.93671	######	######	2
1173	Q96AG4	LRRC59	Leucine-rich repe	sp|Q96AG4|LRC59_HUMAN	5	18.9	0.5937334	0.02437	0.9991	0.6231	0.01899	0.09686	12	40.7	0.28786	0.24636	0.9973	-0.2092	0.395	0.4896	5
1174	Q96AY3	FKBP10	Peptidyl-prolyl c	sp|Q96AY3|FKB10_HUMAN	7	13.6	-0.536043	0.0735	0.9984	0.3525	0.0735	0.99836	12	29.2	NA	NA	NA	NA	NA	NA	NA
1175	Q96CW1	AP2M1	AP-2 complex su	sp|Q96CW1|AP2M1_HUMA	3	7.4	0.0392761	0.83247	0.9984	-0.0411	0.83247	0.99836	4	10.8	0.13586	NA	NA	NA	NA	NA	NA
1176	Q96DI7	SNRNP40	U5 small nuclear	sp|Q96DI7|SNR40_HUMAN	2	10.1	NA	NA	NA	NA	NA	NA	3	17.4	0.0478	NA	NA	NA	NA	NA	NA
1177	Q96FW1	OTUB1	Ubiquitin thioes	sp|Q96FW1|OTUB1_HUMA	5	19.9	0.2039683	0.57103	0.9991	-0.1693	0.73862	0.85849	10	51.7	-0.2424	0.50174	0.9973	2.63606	######	######	2
1178	Q96HE7	ERO1A	ERO1-like protei	sp|Q96HE7|ERO1A_HUMA	1	3	NA	NA	NA	NA	NA	NA	8	18.8	0.3126	0.49867	0.9991	0.69396	0.4987	0.9991	NA
1179	Q96HS1	PGAM5	Serine/threonin	sp|Q96HS1|PGAM5_HUMA	3	11.8	-0.187895	0.4913	0.9984	0.4622	0.4913	0.99836	1	3.5	NA	NA	NA	NA	NA	NA	NA
1180	Q96I24	FUBP3	Far upstream el	sp|Q96I24|FUBP3_HUMAN	6	11.5	0.4131889	0.38526	0.9984	0.2576	0.38526	0.99836	8	18.2	NA	NA	NA	NA	NA	NA	NA
1181	Q96I25	RBM17	Splicing factor 4	sp|Q96I25|SPF45_HUMAN	2	7.2	0.1177614	0.76166	0.9991	0.2539	0.41338	0.60623	5	16.7	0.12784	0.64227	0.9973	-0.1001	0.7157	0.7806	NA
1182	Q96IZ0	PAWR	PRKC apoptosis \	sp|Q96IZ0|PAWR_HUMAN	4	22.4	-0.153095	NA	NA	NA	NA	NA	NA	NA	NA	NA	NA	NA	NA	NA	NA
1183	Q96J01	THOC3	THO complex sul	sp|Q96J01|THOC3_HUMAN	2	4.8	NA	NA	NA	0.505	NA	NA	1	6	NA	NA	NA	NA	NA	NA	NA
1184	Q96JJ7	TMX3	Protein disulfide	sp|Q96JJ7|TMX3_HUMAN	3	7	NA	NA	NA	NA	NA	NA	2	4.2	-0.1268	NA	NA	NA	NA	NA	NA
1185	Q96K17	BTF3L4	Transcription fa	sp|Q96K17|BT3L4_HUMAN	4	38.6	0.0362559	0.87725	0.9984	-0.3463	0.87725	0.99836	4	38.6	NA	NA	NA	NA	NA	NA	NA
1186	Q96KP4	CNDP2	Cytosolic non-sp	sp|Q96KP4|CNDP2_HUMAI	13	37.5	0.1764716	0.51327	0.9984	-1.268	0.51327	0.99836	4	13.3	-0.3784	NA	NA	NA	NA	NA	NA
1187	Q96P16	RPRD1A	Regulation of nu	sp|Q96P16|RPR1A_HUMAN	3	14.1	0.1422392	0.63058	0.9984	-0.1562	0.63058	0.99836	1	3.8	NA	NA	NA	NA	NA	NA	NA
1188	Q96P70	IPO9	Importin-9	sp|Q96P70|IPO9_HUMAN I	8	12.1	-0.220036	0.42933	0.9991	-0.5148	0.10952	0.27869	8	11.9	0.08575	0.75598	0.9973	0.22581	0.4176	0.5104	NA
1189	Q96PK6	RBM14	RNA-binding pro	sp|Q96PK6|RBM14_HUMA	2	2.8	NA	NA	NA	NA	NA	NA	9	14.8	-0.2856	0.37265	0.9991	0.50893	0.3727	0.9991	NA
1190	Q96QD8	SLC38A2	Sodium-coupled	sp|Q96QD8|S38A2_HUMA	2	7.3	-0.352205	0.24295	0.9984	0.2593	0.24295	0.99836	1	4.2	NA	NA	NA	NA	NA	NA	NA
1191	Q96QK1	VPS35	Vacuolar protein	sp|Q96QK1|VPS35_HUMAI	4	7.2	0.1154853	0.55385	0.9991	-0.5623	0.01843	0.09524	11	19.3	0.04891	0.8011	0.9973	-0.2179	0.2709	0.3554	3
1192	Q96RE7	NACC1	Nucleus accumbe	sp|Q96RE7|NACC1_HUMAI	5	14	0.2150432	0.52613	0.9984	0.7002	0.52613	0.99836	1	2.5	NA	NA	NA	NA	NA	NA	NA
1193	Q96RP9	GFM1	Elongation facto	sp|Q96RP9|EFGM_HUMAN	5	7.3	0.2416325	0.63483	0.9984	0.5503	0.63483	0.99836	6	10.9	NA	NA	NA	NA	NA	NA	NA
1194	Q96RS6	NUDCD1	NudC domain-co	sp|Q96RS6|NUDC1_HUMA	4	9.3	0.1191745	0.57979	0.9984	0.0873	0.57979	0.99836	3	6.7	NA	NA	NA	NA	NA	NA	NA
1195	Q96SQ9	CYP2S1	Cytochrome P45	sp|Q96SQ9|CP2S1_HUMAN	5	11.7	0.2294864	0.21315	0.9984	0.6132	0.21315	0.99836	NA	NA	NA	NA	NA	NA	NA	NA	NA
1196	Q96TA1	FAM129B	Niban-like prote	sp|Q96TA1|NIBL1_HUMAN	2	3.6	NA	NA	NA	NA	NA	NA	3	4.3	-0.5765	NA	NA	NA	NA	NA	NA

1197	Q99417	MYCBP	c-Myc-binding pr	sp|Q99417|MYCBP_HUMA	1	10.7	NA	NA	NA	NA	NA	NA	4	55.3	0.16551	0.78868	0.9991	0.00902	0.7887	0.9991	NA
1198	Q99426	TBCB	Tubulin-folding c	sp|Q99426|TBCB_HUMAN T	7	32	-0.893884	0.03457	0.9984	-1.3226	0.03457	0.99836	6	27	NA	NA	NA	NA	NA	NA	NA
1199	Q99436	PSMB7	Proteasome sub	sp|Q99436|PSB7_HUMAN	4	17.7	0.0397289	0.83325	0.9991	-0.2091	0.27693	0.47715	6	31.4	-0.0727	0.70057	0.9973	-1.1535	######	0.0003	4
1200	Q99439	CNN2	Calponin-2	sp|Q99439|CNN2_HUMAN	5	17.2	-0.237688	0.21714	0.9984	-0.4055	0.21714	0.99836	6	23.3	NA	NA	NA	NA	NA	NA	NA
1201	Q99459	CDC5L	Cell division cycl	sp|Q99459|CDC5L_HUMAN	8	17.3	0.1668139	0.38819	0.9991	0.4562	0.02725	0.12063	13	22.6	0.08994	0.63901	0.9973	-0.4216	0.0393	0.0705	5
1202	Q99460	PSMD1	26S proteasome	sp|Q99460|PSMD1_HUMA	21	31.1	-0.036131	0.84299	0.9991	-0.1446	0.43222	0.6164	23	34.1	-0.1189	0.51703	0.9973	-0.0626	0.7318	0.7909	NA
1203	Q99471	PFDN5	Prefoldin subunit	sp|Q99471|PFD5_HUMAN	3	26.6	NA	NA	NA	NA	NA	NA	6	60.4	-0.242	NA	NA	NA	NA	NA	NA
1204	Q99497	PARK7	Protein/nucleic a	sp|Q99497|PARK7_HUMAN	10	55	0.3623403	0.48157	0.9991	-0.7257	0.16803	0.34234	15	76.7	-0.7703	0.14486	0.9973	4.0875	######	######	2
1205	Q99536	VAT1	Synaptic vesicle	sp|Q99536|VAT1_HUMAN	8	31.3	0.0713482	0.72702	0.9991	-0.1262	0.5385	0.70183	7	26.5	0.32255	0.12752	0.9973	-0.0733	0.7199	0.7823	NA
1206	Q99543	DNAJC2	DnaJ homolog su	sp|Q99543|DNJC2_HUMAN	4	7.2	-0.261967	NA	NA	NA	NA	NA	3	5.6	NA	NA	NA	NA	NA	NA	NA
1207	Q99584	S100A13	Protein S100-A1	sp|Q99584|S10AD_HUMAN	4	40.8	0.2059173	0.30262	0.9984	-0.5218	0.30262	0.99836	5	48	NA	NA	NA	NA	NA	NA	NA
1208	Q99615	DNAJC7	DnaJ homolog su	sp|Q99615|DNJC7_HUMAN	9	18	0.2285936	0.45004	0.9991	0.1098	0.71491	0.84478	9	21.5	-0.1617	0.59153	0.9973	-1.2291	0.0007	0.0024	4
1209	Q99623	PHB2	Prohibitin-2	sp|Q99623|PHB2_HUMAN	13	44.1	0.1807574	0.5078	0.9991	1.0549	0.0011	0.02437	14	51.8	-0.0019	0.99453	0.9973	0.92268	0.0032	0.0084	1
1210	Q99714	HSD17B10	3-hydroxyacyl-Co	sp|Q99714|HCD2_HUMAN	3	19.2	-0.028379	0.90412	0.9991	0.8237	0.02147	0.10155	8	50.6	-0.68	0.02035	0.9973	1.06139	0.0007	0.0024	1
1211	Q99729	HNRNPAB	Heterogeneous	sp|Q99729|ROAA_HUMAN	8	22.6	-0.039623	0.89929	0.9991	0.7001	0.03864	0.14651	9	25	-2.1319	0.00022	0.1542	1.31441	0.0007	0.0024	1
1212	Q99733	NAP1L4	Nucleosome asse	sp|Q99733|NP1L4_HUMAN	9	31.5	-0.019484	0.91631	0.999	-0.7898	0.91631	0.99899	11	36.5	NA	NA	NA	NA	NA	NA	NA
1213	Q99747	NAPG	Gamma-soluble	sp|Q99747|SNAG_HUMAN	1	3.2	NA	NA	NA	NA	NA	NA	6	22.1	0.04934	0.80947	0.9991	-0.6856	0.8095	0.9991	NA
1214	Q99798	ACO2	Aconitate hydra	sp|Q99798|ACON_HUMAN	15	28.1	0.0654693	0.77402	0.9991	0.7492	0.00405	0.03837	15	26.9	0.17285	0.45187	0.9973	-0.2039	0.3766	0.4717	5
1215	Q99808	SLC29A1	Equilibrative nu	sp|Q99808|S29A1_HUMAN	2	6.6	0.0017254	0.99454	0.999	1.0218	0.99454	0.99899	NA	NA	NA	NA	NA	NA	NA	NA	NA
1216	Q99832	CCT7	T-complex prote	sp|Q99832|TCPH_HUMAN	20	47.7	0.0463774	0.84132	0.9991	-0.4541	0.06336	0.20018	24	57.1	#######	0.9997	0.9997	-0.378	0.1164	0.1782	NA
1217	Q99848	EBNA1BP2	Probable rRNA-p	sp|Q99848|EBP2_HUMAN	1	2.9	NA	NA	NA	NA	NA	NA	8	27.1	NA	NA	NA	2.42736	NA	NA	NA
1218	Q99873	PRMT1	Protein arginine	sp|Q99873|ANM1_HUMAN	16	47.2	0.2275069	0.35544	0.9991	-0.6254	0.01854	0.09524	13	40.7	-0.1565	0.52207	0.9973	-0.8853	0.0019	0.0054	3
1219	Q99961	SH3GL1	Endophilin-A2	sp|Q99961|SH3G1_HUMAN	4	13.9	-0.203203	NA	NA	NA	NA	NA	12	42.9	-0.261	0.66258	0.9991	1.33415	0.6626	0.9991	NA
1220	Q9BPW8	NIPSNAP1	Protein NipSnap	sp|Q9BPW8|NIPS1_HUMAN	2	6.3	-0.425874	NA	NA	NA	NA	NA	5	24.6	NA	NA	NA	NA	NA	NA	NA
1221	Q9BQE3	TUBA1C	Tubulin alpha-1C	sp|Q9BQE3|TBA1C_HUMAN	17	45.4	0.5433191	0.19242	0.9991	-0.4075	0.42986	0.6164	26	65.9	0.46715	0.20911	0.9973	-0.1337	0.7117	0.7775	NA
1222	Q9BQI0	AIF1L	Allograft inflamm	sp|Q9BQI0|AIF1L_HUMAN	5	38	0.0805194	0.77987	0.9984	0.1942	0.77987	0.99836	NA	NA	NA	NA	NA	NA	NA	NA	NA
1223	Q9BRA2	TXNDC17	Thioredoxin dom	sp|Q9BRA2|TXD17_HUMAN	4	33.3	0.6906899	0.25283	0.9984	0.7568	0.25283	0.99836	4	37.4	NA	NA	NA	NA	NA	NA	NA
1224	Q9BRK5	SDF4	45 kDa calcium-b	sp|Q9BRK5|CAB45_HUMAN	3	13.8	NA	NA	NA	NA	NA	NA	5	15.2	0.21153	0.60427	0.9991	0.40203	0.6043	0.9991	NA
1225	Q9BRP8	PYM1	Partner of Y14 a	sp|Q9BRP8|PYM1_HUMAN	5	33.8	-0.227622	0.56137	0.9991	0.0836	0.83027	0.9155	6	40.2	-0.011	0.98166	0.9973	1.2145	0.0129	0.0275	1
1226	Q9BRX8	PRXL2A	Peroxiredoxin-lik	sp|Q9BRX8|PXL2A_HUMAN	4	20.1	0.4684302	0.32529	0.9984	0.4077	0.32529	0.99836	4	20.1	NA	NA	NA	NA	NA	NA	NA
1227	Q9BSJ8	ESYT1	Extended synapt	sp|Q9BSJ8|ESYT1_HUMAN	7	9.4	-0.367492	0.11722	0.9984	0.0959	0.11722	0.99836	8	10.7	NA	NA	NA	-0.5125	NA	NA	NA
1228	Q9BT09	CNPY3	Protein canopy	sp|Q9BT09|CNPY3_HUMAN	3	14	-0.352693	0.40913	0.9984	-0.4169	0.40913	0.99836	4	19.8	NA	NA	NA	NA	NA	NA	NA
1229	Q9BT78	COPS4	COP9 signalosom	sp|Q9BT78|CSN4_HUMAN	4	13.5	0.3037698	0.34395	0.9991	-0.272	0.45946	0.6439	13	43.8	-0.1343	0.52561	0.9973	0.17412	0.4134	0.5071	NA
1230	Q9BTT0	ANP32E	Acidic leucine-ric	sp|Q9BTT0|AN32E_HUMAN	4	29.1	0.407071	NA	NA	NA	NA	NA	7	40.3	-0.1631	0.72873	0.9991	2.25718	0.7287	0.9991	NA
1231	Q9BUF5	TUBB6	Tubulin beta-6 c	sp|Q9BUF5|TBB6_HUMAN	8	21.5	NA	NA	NA	NA	NA	NA	17	50.4	NA	NA	NA	-0.8677	NA	NA	NA
1232	Q9BUJ2	HNRNPUL1	Heterogeneous	sp|Q9BUJ2|HNRL1_HUMAN	7	10	-0.166797	0.52113	0.9991	0.426	0.113	0.27928	8	11.3	0.55257	0.04486	0.9973	0.31081	0.2391	0.3235	1
1233	Q9BUL8	PDCD10	Programmed cel	sp|Q9BUL8|PDC10_HUMAN	2	17	0.0628339	NA	NA	NA	NA	NA	2	11.8	NA	NA	NA	NA	NA	NA	NA
1234	Q9BUQ8	DDX23	Probable ATP-de	sp|Q9BUQ8|DDX23_HUMA	3	5.5	NA	NA	NA	NA	NA	NA	8	12.4	-0.1695	NA	NA	NA	NA	NA	NA
1235	Q9BV38	WDR18	WD repeat-cont	sp|Q9BV38|WDR18_HUMA	2	4.9	-0.987723	0.07638	0.9984	-0.1668	0.07638	0.99836	NA	NA	NA	NA	NA	NA	NA	NA	NA
1236	Q9BV40	VAMP8	Vesicle-associate	sp|Q9BV40|VAMP8_HUMA	2	22	NA	NA	NA	NA	NA	NA	2	22	0.37951	NA	NA	NA	NA	NA	NA
1237	Q9BVA1	TUBB2B	Tubulin beta-2B	sp|Q9BVA1|TBB2B_HUMAN	14	39.1	-0.025757	0.92245	0.999	-1.1142	0.92245	0.99899	22	71.7	NA	NA	NA	NA	NA	NA	NA
1238	Q9BVG4	PBDC1	Protein PBDC1	sp|Q9BVG4|PBDC1_HUMA	7	30.5	0.0294416	NA	NA	NA	NA	NA	4	20.6	0.3939	NA	NA	NA	NA	NA	NA
1239	Q9BW27	NUP85	Nuclear pore cor	sp|Q9BW27|NUP85_HUMA	2	3.2	-0.148411	0.71954	0.9984	0.5194	0.71954	0.99836	3	6.1	NA	NA	NA	NA	NA	NA	NA
1240	Q9BWD1	ACAT2	Acetyl-CoA acet	sp|Q9BWD1|THIC_HUMAN	6	22.7	-0.10872	0.55354	0.9984	-0.2321	0.55354	0.99836	NA	NA	NA	NA	NA	NA	NA	NA	NA
1241	Q9BWF3	RBM4	RNA-binding pro	sp|Q9BWF3|RBM4_HUMAN	7	25.8	-0.084428	0.75078	0.9984	0.6208	0.75078	0.99836	11	34.6	NA	NA	NA	2.5035	NA	NA	NA
1242	Q9BWU0	SLC4A1AP	Kanadaptin	sp|Q9BWU0|NADAP_HUM	3	5.8	-0.06404	NA	NA	NA	NA	NA	1	1.1	NA	NA	NA	NA	NA	NA	NA
1243	Q9BXJ9	NAA15	N-alpha-acetyltr	sp|Q9BXJ9|NAA15_HUMAN	14	16.6	0.275592	0.29552	0.9984	-0.0731	0.29552	0.99836	16	22.2	NA	NA	NA	NA	NA	NA	NA
1244	Q9BXP5	SRRT	Serrate RNA effe	sp|Q9BXP5|SRRT_HUMAN	10	13.1	0.0011983	0.99726	0.9991	0.5325	0.14188	0.30951	13	18.9	0.18983	0.54616	0.9973	-0.406	0.2062	0.2935	NA

1245	Q9BY44	EIF2A	Eukaryotic trans	sp|Q9BY44|EIF2A_HUMAN	5	12.3	-0.048683	0.81019	0.9984	-0.296	0.81019	0.99836	10	25.5	NA	NA	NA	NA	NA	NA	NA
1246	Q9BYD6	MRPL1	39S ribosomal pı	sp|Q9BYD6|RM01_HUMAN	4	20.6	0.8998424	0.01195	0.9984	0.8195	0.01195	0.99836	4	20.6	NA	NA	NA	NA	NA	NA	NA
1247	Q9BYG3	NIFK	MKI67 FHA dom	sp|Q9BYG3|MK67I_HUMAI	5	25.3	-0.075029	0.85373	0.9984	0.2118	0.85373	0.99836	4	20.1	NA	NA	NA	NA	NA	NA	NA
1248	Q9BYT8	NLN	Neurolysin, mitc	sp|Q9BYT8|NEUL_HUMAN	7	12.4	0.3859671	0.04775	0.9984	0.0753	0.04775	0.99836	9	15.1	NA	NA	NA	NA	NA	NA	NA
1249	Q9BZE4	GTPBP4	Nucleolar GTP-bi	sp|Q9BZE4|NOG1_HUMAN	5	6.6	0.0667243	0.76588	0.9984	0.3158	0.76588	0.99836	9	16.1	0.13874	NA	NA	NA	NA	NA	NA
1250	Q9BZX2	UCK2	Uridine-cytidine	sp|Q9BZX2|UCK2_HUMAN	2	8.4	NA	NA	NA	NA	NA	NA	5	33	-0.157	NA	NA	NA	NA	NA	NA
1251	Q9BZZ5	API5	Apoptosis inhibit	sp|Q9BZZ5|API5_HUMAN A	6	10.7	0.0493187	0.83891	0.9991	0.0122	0.96416	0.97378	10	23.5	0.24834	0.31393	0.9973	0.12298	0.6135	0.7016	NA
1252	Q9C005	DPY30	Protein dpy-30 h	sp|Q9C005|DPY30_HUMAN	2	36.4	-0.023023	0.91853	0.999	0.5702	0.91853	0.99899	4	74.7	NA	NA	NA	NA	NA	NA	NA
1253	Q9C0C9	UBE2O	(E3-independent	sp|Q9C0C9|UBE2O_HUMAI	7	7.3	0.3194222	NA	NA	NA	NA	NA	4	4.3	NA	NA	NA	NA	NA	NA	NA
1254	Q9GZL7	WDR12	Ribosome biogeı	sp|Q9GZL7|WDR12_HUMA	4	11.3	-0.087022	0.79345	0.9991	-0.2826	0.52888	0.6944	8	25.8	-0.073	0.80586	0.9973	0.53834	0.0869	0.1427	NA
1255	Q9GZR7	DDX24	ATP-dependent	sp|Q9GZR7|DDX24_HUMAI	4	5.9	-0.011918	0.982	0.999	0.3602	0.982	0.99899	6	8.3	NA	NA	NA	NA	NA	NA	NA
1256	Q9GZS3	WDR61	WD repeat-conta	sp|Q9GZS3|WDR61_HUMA	3	14.8	NA	NA	NA	NA	NA	NA	8	46.6	0.14684	0.55705	0.9991	0.39535	0.557	0.9991	NA
1257	Q9GZT3	SLIRP	SRA stem-loop-ir	sp|Q9GZT3|SLIRP_HUMAN	2	22	0.2029317	0.62207	0.9984	0.5265	0.62207	0.99836	8	69.7	NA	NA	NA	NA	NA	NA	NA
1258	Q9GZZ1	NAA50	N-alpha-acetyltr	sp|Q9GZZ1|NAA50_HUMA	3	17.8	0.0318623	0.9027	0.9991	-0.6031	0.03163	0.12742	7	47.9	0.14329	0.58417	0.9973	1.06896	0.0007	0.0024	2
1259	Q9H0A0	NAT10	RNA cytidine ace	sp|Q9H0A0|NAT10_HUMA	7	8.6	0.1327551	0.65647	0.9984	0.5977	0.65647	0.99836	8	12.5	NA	NA	NA	NA	NA	NA	NA
1260	Q9H0D6	XRN2	5-3 exoribonucle	sp|Q9H0D6|XRN2_HUMAN	6	8.4	-0.419695	0.18225	0.9984	0.4386	0.18225	0.99836	7	9.8	NA	NA	NA	NA	NA	NA	NA
1261	Q9H0S4	DDX47	Probable ATP-de	sp|Q9H0S4|DDX47_HUMAI	2	5.3	NA	NA	NA	NA	NA	NA	4	10.3	-1.209	0.0157	0.9991	-0.921	0.0157	0.9991	NA
1262	Q9H0U4	RAB1B	Ras-related prot	sp|Q9H0U4|RAB1B_HUMAI	7	48.3	-0.017143	0.96045	0.999	0.4514	0.96045	0.99899	10	58.7	NA	NA	NA	NA	NA	NA	NA
1263	Q9H1E3	NUCKS1	Nuclear ubiquitc	sp|Q9H1E3|NUCKS_HUMAI	10	47.3	0.5826035	0.1389	0.9991	-0.2182	0.56419	0.72466	3	14.4	-0.0332	0.95934	0.9973	1.62726	0.0085	0.0191	2
1264	Q9H223	EHD4	EH domain-conta	sp|Q9H223|EHD4_HUMAN	2	4.8	NA	NA	NA	NA	NA	NA	8	17	-0.2757	0.28943	0.9991	-1.4089	0.2894	0.9991	NA
1265	Q9H2U2	PPA2	Inorganic pyroph	sp|Q9H2U2|IPYR2_HUMAN	3	12.3	0.0664253	0.77111	0.9984	0.1515	0.77111	0.99836	7	29.3	NA	NA	NA	NA	NA	NA	NA
1266	Q9H307	PNN	Pinin	sp|Q9H307|PININ_HUMAN	10	14.8	0.563627	0.17324	0.9984	1.5058	0.17324	0.99836	7	9.8	NA	NA	NA	NA	NA	NA	NA
1267	Q9H3K6	BOLA2	BolA-like proteir	sp|Q9H3K6|BOLA2_HUMAI	2	29.1	-0.044878	0.88102	0.9984	-0.4237	0.88102	0.99836	5	69.8	NA	NA	NA	NA	NA	NA	NA
1268	Q9H3N1	TMX1	Thioredoxin-rela	sp|Q9H3N1|TMX1_HUMAN	6	17.9	0.1897915	0.52877	0.9984	0.6494	0.52877	0.99836	3	12.1	NA	NA	NA	NA	NA	NA	NA
1269	Q9H444	CHMP4B	Charged multive	sp|Q9H444|CHM4B_HUMA	6	30.4	-0.078797	0.86691	0.9991	-0.9329	0.12329	0.28797	6	37.9	-0.0768	0.84156	0.9973	0.6386	0.1145	0.1757	NA
1270	Q9H4A4	RNPEP	Aminopeptidase	sp|Q9H4A4|AMPB_HUMAI	3	6	0.0130021	NA	NA	NA	NA	NA	10	20.8	NA	NA	NA	NA	NA	NA	NA
1271	Q9H4M9	EHD1	EH domain-conta	sp|Q9H4M9|EHD1_HUMAI	1	2.1	NA	NA	NA	NA	NA	NA	8	22.3	-0.2344	0.4652	0.9991	-0.3626	0.4652	0.9991	NA
1272	Q9H583	HEATR1	HEAT repeat-cor	sp|Q9H583|HEAT1_HUMA	6	3.7	-0.071316	0.7329	0.9984	0.1862	0.7329	0.99836	7	3.7	NA	NA	NA	0.22617	NA	NA	NA
1273	Q9H6T3	RPAP3	RNA polymerase	sp|Q9H6T3|RPAP3_HUMAI	5	9.8	0.0423898	0.89621	0.9984	-0.1405	0.89621	0.99836	4	8	NA	NA	NA	NA	NA	NA	NA
1274	Q9H6Z4	RANBP3	Ran-binding prot	sp|Q9H6Z4|RANB3_HUMA	3	6.3	-0.018604	0.93592	0.999	-0.6431	0.93592	0.99899	4	9.5	NA	NA	NA	NA	NA	NA	NA
1275	Q9H7Z7	PTGES2	Prostaglandin E	sp|Q9H7Z7|PGES2_HUMAI	3	6.4	NA	NA	NA	0.4841	NA	NA	2	7.4	NA	NA	NA	NA	NA	NA	NA
1276	Q9H8Y8	GORASP2	Golgi reassembly	sp|Q9H8Y8|GORS2_HUMA	4	10.8	-0.329845	0.21242	0.9984	-0.6932	0.21242	0.99836	4	12.2	NA	NA	NA	NA	NA	NA	NA
1277	Q9H910	JPT2	Jupiter microtub	sp|Q9H910|JUPI2_HUMAN	7	48.9	0.3338711	0.456	0.9984	-0.569	0.456	0.99836	6	41.6	NA	NA	NA	NA	NA	NA	NA
1278	Q9H9B4	SFXN1	Sideroflexin-1	sp|Q9H9B4|SFXN1_HUMAI	3	12.7	0.1874228	0.3366	0.9984	0.4357	0.3366	0.99836	3	13	NA	NA	NA	NA	NA	NA	NA
1279	Q9H9Z2	LIN28A	Protein lin-28 hc	sp|Q9H9Z2|LN28A_HUMAI	12	58.4	0.0513984	0.76893	0.9984	-0.2414	0.76893	0.99836	NA	NA	NA	NA	NA	NA	NA	NA	NA
1280	Q9HAV4	XPO5	Exportin-5	sp|Q9HAV4|XPO5_HUMAN	7	8.7	-0.174203	0.33416	0.9984	-0.1273	0.33416	0.99836	16	19.5	NA	NA	NA	NA	NA	NA	NA
1281	Q9HAV7	GRPEL1	GrpE protein hoı	sp|Q9HAV7|GRPE1_HUMA	3	20.3	0.2842111	0.27504	0.9984	0.9215	0.27504	0.99836	5	28.6	0.25439	NA	NA	NA	NA	NA	NA
1282	Q9HB71	CACYBP	Calcyclin-binding	sp|Q9HB71|CYBP_HUMAN	11	39	0.0499102	0.8011	0.9991	-0.3637	0.08006	0.22981	13	56.1	0.07822	0.69341	0.9973	0.91325	0.0002	0.001	2
1283	Q9HC07	TMEM165	Transmembrane	sp|Q9HC07|TM165_HUMA	3	19.4	0.2195471	0.46457	0.9984	0.473	0.46457	0.99836	1	5.6	NA	NA	NA	NA	NA	NA	NA
1284	Q9HC38	GLOD4	Glyoxalase doma	sp|Q9HC38|GLOD4_HUMA	5	16.9	0.1746621	0.53803	0.9984	-0.2731	0.53803	0.99836	5	17.3	NA	NA	NA	NA	NA	NA	NA
1285	Q9HCE1	MOV10	Helicase MOV-1(	sp|Q9HCE1|MOV10_HUMA	2	2.8	0.1136047	0.78263	0.9984	0.1374	0.78263	0.99836	NA	NA	NA	NA	NA	NA	NA	NA	NA
1286	Q9HCN8	SDF2L1	Stromal cell-deri	sp|Q9HCN8|SDF2L_HUMAI	1	5.4	NA	NA	NA	NA	NA	NA	5	27.1	0.48174	NA	NA	NA	NA	NA	NA
1287	Q9HD45	TM9SF3	Transmembrane	sp|Q9HD45|TM9S3_HUMA	3	5.8	-1.043374	0.04896	0.9991	1.172	0.03119	0.12742	3	7.5	0.11053	0.71498	0.9973	0.07785	0.7966	0.8355	5
1288	Q9NP72	RAB18	Ras-related prot	sp|Q9NP72|RAB18_HUMAI	5	32	0.387441	0.13475	0.9991	-0.1616	0.51986	0.6877	4	27.2	0.02959	0.89452	0.9973	-0.3793	0.104	0.1635	NA
1289	Q9NP97	DYNLRB1	Dynein light cha	sp|Q9NP97|DLRB1_HUMAN	4	57.3	0.5463025	NA	NA	NA	NA	NA	4	59.4	NA	NA	NA	NA	NA	NA	NA
1290	Q9NPA8	ENY2	Transcription an	sp|Q9NPA8|ENY2_HUMAN	1	16.8	NA	NA	NA	NA	NA	NA	1	16.8	NA	NA	NA	NA	NA	NA	NA
1291	Q9NPJ3	ACOT13	Acyl-coenzyme A	sp|Q9NPJ3|ACO13_HUMAI	3	30.7	0.4240398	0.20041	0.9984	0.9338	0.20041	0.99836	3	25	NA	NA	NA	NA	NA	NA	NA
1292	Q9NQC3	RTN4	Reticulon-4	sp|Q9NQC3|RTN4_HUMAN	11	14.5	-0.157925	0.39688	0.9984	0.3172	0.39688	0.99836	7	8.6	NA	NA	NA	0.28559	NA	NA	NA

1293	Q9NQP4	PFDN4	Prefoldin subunit	sp|Q9NQP4|PFD4_HUMAN	2	20.1	-0.131648	NA	NA	NA	NA	NA	2	20.1	NA	NA	NA	NA	NA	NA	NA
1294	Q9NQW6	ANLN	Anillin	sp|Q9NQW6|ANLN_HUMA	2	2.7	-0.206792	0.64071	0.9984	0.8984	0.64071	0.99836	2	3.2	NA	NA	NA	NA	NA	NA	NA
1295	Q9NQW7	XPNPEP1	Xaa-Pro aminope	sp|Q9NQW7|XPP1_HUMAN	1	1.8	NA	NA	NA	NA	NA	NA	6	11.9	-0.0229	0.89706	0.9991	-0.4138	0.8971	0.9991	NA
1296	Q9NR30	DDX21	Nucleolar RNA h	sp|Q9NR30|DDX21_HUMA	27	37.8	0.0908945	0.82686	0.9991	0.9905	0.02739	0.12063	27	43	-0.2239	0.59144	0.9973	2.28611	######	0.0002	1
1297	Q9NR31	SAR1A	GTP-binding prot	sp|Q9NR31|SAR1A_HUMA	4	26.8	0.1287896	0.59325	0.9991	-0.8872	0.00417	0.03837	6	42.9	0.11216	0.64142	0.9973	-0.6358	0.0164	0.0343	3
1298	Q9NR45	NANS	Sialic acid syntha	sp|Q9NR45|SIAS_HUMAN	4	14.5	0.2111359	NA	NA	NA	NA	NA	3	12.8	-0.1174	0.70191	0.9991	-1.3135	0.7019	0.9991	NA
1299	Q9NR50	EIF2B3	Translation initia	sp|Q9NR50|EI2BG_HUMAN	4	12.6	NA	NA	NA	NA	NA	NA	7	22.6	-0.04	NA	NA	NA	NA	NA	NA
1300	Q9NRF8	CTPS2	CTP synthase 2	sp|Q9NRF8|PYRG2_HUMAN	3	5.6	-0.313466	NA	NA	NA	NA	NA	5	9.9	NA	NA	NA	NA	NA	NA	NA
1301	Q9NRV9	HEBP1	Heme-binding pr	sp|Q9NRV9|HEBP1_HUMA	1	11.1	NA	NA	NA	NA	NA	NA	2	19	-0.0594	NA	NA	NA	NA	NA	NA
1302	Q9NRZ9	HELLS	Lymphoid-specifi	sp|Q9NRZ9|HELLS_HUMAN	6	7.2	0.2950048	0.25493	0.9984	0.6218	0.25493	0.99836	NA	NA	NA	NA	NA	NA	NA	NA	NA
1303	Q9NS69	TOMM22	Mitochondrial in	sp|Q9NS69|TOM22_HUMA	4	52.1	0.0007118	0.99692	0.999	0.5523	0.99692	0.99899	2	16.2	NA	NA	NA	NA	NA	NA	NA
1304	Q9NSD9	FARSB	Phenylalanine--t	sp|Q9NSD9|SYFB_HUMAN	8	12.9	-0.033947	0.91447	0.9991	0.1539	0.73118	0.85314	12	23.1	-0.0186	0.95302	0.9973	0.78753	0.0234	0.0458	1
1305	Q9NSE4	IARS2	Isoleucine--tRNA	sp|Q9NSE4|SYIM_HUMAN	4	6.2	-0.093703	0.71101	0.9984	0.9706	0.71101	0.99836	5	7.4	-0.2907	NA	NA	NA	NA	NA	NA
1306	Q9NT62	ATG3	Ubiquitin-like-co	sp|Q9NT62|ATG3_HUMAN	2	7.3	-0.092533	NA	NA	NA	NA	NA	2	7.3	NA	NA	NA	NA	NA	NA	NA
1307	Q9NTJ3	SMC4	Structural maint	sp|Q9NTJ3|SMC4_HUMAN	19	16.5	-0.066605	0.80275	0.9984	0.3618	0.80275	0.99836	5	4.6	NA	NA	NA	NA	NA	NA	NA
1308	Q9NTJ5	SACM1L	Phosphatidylinos	sp|Q9NTJ5|SAC1_HUMAN	6	10.4	-0.396494	0.36397	0.9984	0.4419	0.36397	0.99836	NA	NA	NA	NA	NA	NA	NA	NA	NA
1309	Q9NTK5	OLA1	Obg-like ATPase	sp|Q9NTK5|OLA1_HUMAN	8	20.7	-0.056454	0.7743	0.9991	-0.7904	0.00083	0.02112	13	31.8	0.0587	0.76561	0.9973	-0.3962	0.0572	0.0984	3
1310	Q9NU22	MDN1	Midasin	sp|Q9NU22|MDN1_HUMA	14	3.5	0.12779	0.74939	0.9984	1.024	0.74939	0.99836	3	0.7	0.07994	NA	NA	NA	NA	NA	NA
1311	Q9NUQ3	TXLNG	Gamma-taxilin	sp|Q9NUQ3|TXLNG_HUMA	6	13.8	0.2478033	0.23917	0.9984	-0.358	0.23917	0.99836	2	3.6	NA	NA	NA	NA	NA	NA	NA
1312	Q9NUQ9	FAM49B	Protein FAM49B	sp|Q9NUQ9|FA49B_HUMA	5	19.8	0.390761	NA	NA	NA	NA	NA	7	28.1	0.26613	0.31655	0.9991	-0.3978	0.3165	0.9991	NA
1313	Q9NUU7	DDX19A	ATP-dependent	sp|Q9NUU7|DD19A_HUMA	7	16.7	0.403169	NA	NA	NA	NA	NA	8	20.3	-0.2489	0.3926	0.9991	-0.0358	0.3926	0.9991	NA
1314	Q9NVA2	Sep-11	Septin-11	sp|Q9NVA2|SEP11_HUMA	7	17	0.0176473	NA	NA	NA	NA	NA	12	35.4	0.48768	0.11965	0.9991	1.81375	0.1196	0.9991	NA
1315	Q9NVI7	ATAD3A	ATPase family A	sp|Q9NVI7|ATD3A_HUMA	4	8.7	-0.253409	0.35172	0.9984	0.4013	0.35172	0.99836	5	7.4	NA	NA	NA	NA	NA	NA	NA
1316	Q9NVP1	DDX18	ATP-dependent	sp|Q9NVP1|DDX18_HUMA	7	11.5	0.1326176	0.64201	0.9991	0.5859	0.05406	0.18164	12	24.3	0.04086	0.90657	0.9973	0.31052	0.3364	0.4262	NA
1317	Q9NVU7	SDAD1	Protein SDA1 ho	sp|Q9NVU7|SDA1_HUMAN	3	4.9	0.1489324	0.71862	0.9984	0.0869	0.71862	0.99836	3	4.5	0.3607	NA	NA	NA	NA	NA	NA
1318	Q9NWH9	SLTM	SAFB-like transc	sp|Q9NWH9|SLTM_HUMA	5	6.4	0.2573606	0.50282	0.9984	0.9943	0.50282	0.99836	2	3.2	NA	NA	NA	NA	NA	NA	NA
1319	Q9NWV4	CZIB	CXXC motif conta	sp|Q9NWV4|CZIB_HUMAN	2	16.9	0.0971704	NA	NA	NA	NA	NA	NA	NA	NA	NA	NA	NA	NA	NA	NA
1320	Q9NX55	HYPK	Huntingtin-inter	sp|Q9NX55|HYPK_HUMAN	3	14.7	0.5517683	NA	NA	NA	NA	NA	4	33.3	NA	NA	NA	NA	NA	NA	NA
1321	Q9NX58	LYAR	Cell growth-regu	sp|Q9NX58|LYAR_HUMAN	2	7.4	-0.289102	0.38244	0.9984	1.9279	0.38244	0.99836	2	9.2	NA	NA	NA	NA	NA	NA	NA
1322	Q9NXG2	THUMPD1	THUMP domain-	sp|Q9NXG2|THUM1_HUMA	5	20.1	0.600099	0.08331	0.9984	0.2886	0.08331	0.99836	1	2.3	NA	NA	NA	NA	NA	NA	NA
1323	Q9NY33	DPP3	Dipeptidyl pepti	sp|Q9NY33|DPP3_HUMAN	6	10.2	-0.289388	NA	NA	NA	NA	NA	8	16.3	0.17776	0.67696	0.9991	-1.0968	0.677	0.9991	NA
1324	Q9NYF8	BCLAF1	Bcl-2-associated	sp|Q9NYF8|BCLF1_HUMAN	7	9	0.1489329	0.76785	0.9991	0.8461	0.10708	0.27697	10	12.9	-0.3327	0.51193	0.9973	0.61081	0.2358	0.3208	NA
1325	Q9NYK5	MRPL39	39S ribosomal pr	sp|Q9NYK5|RM39_HUMAN	4	12.1	-0.396182	0.34522	0.9984	-0.2024	0.34522	0.99836	4	12.4	NA	NA	NA	NA	NA	NA	NA
1326	Q9NYU2	UGGT1	UDP-glucose:glyc	sp|Q9NYU2|UGGG1_HUMA	7	5.9	-0.038585	0.91261	0.9991	0.2751	0.48868	0.66502	14	11.6	0.17982	0.76817	0.9973	1.30057	0.0211	0.0417	1
1327	Q9NZ01	TECR	Very-long-chain	sp|Q9NZ01|TECR_HUMAN	3	9.1	0.3078784	0.33749	0.9984	0.6573	0.33749	0.99836	2	6.8	NA	NA	NA	NA	NA	NA	NA
1328	Q9NZ45	CISD1	CDGSH iron-sulfu	sp|Q9NZ45|CISD1_HUMAN	3	34.3	0.3178425	0.22534	0.9984	1.0661	0.22534	0.99836	2	25.9	NA	NA	NA	NA	NA	NA	NA
1329	Q9NZB2	FAM120A	Constitutive coa	sp|Q9NZB2|F120A_HUMAN	3	3.5	NA	NA	NA	NA	NA	NA	5	4.4	-0.3966	NA	NA	NA	NA	NA	NA
1330	Q9NZL9	MAT2B	Methionine ade	sp|Q9NZL9|MAT2B_HUMA	4	18	NA	NA	NA	-0.6856	NA	NA	4	12.9	0.15174	0.6432	0.9991	0.88234	0.6432	0.9991	NA
1331	Q9P016	THYN1	Thymocyte nucle	sp|Q9P016|THYN1_HUMAN	6	25.3	0.3824536	0.04638	0.9984	0.2971	0.04638	0.99836	4	16.9	NA	NA	NA	NA	NA	NA	NA
1332	Q9P0L0	VAPA	Vesicle-associate	sp|Q9P0L0|VAPA_HUMAN	6	30.5	0.0489447	0.78855	0.9984	0.4991	0.78855	0.99836	7	35.3	NA	NA	NA	NA	NA	NA	NA
1333	Q9P0V9	Sep-10	Septin-10	sp|Q9P0V9|SEP10_HUMAN	5	15.6	0.2632029	NA	NA	NA	NA	NA	NA	NA	NA	NA	NA	NA	NA	NA	NA
1334	Q9P1F3	ABRACL	Costars family pr	sp|Q9P1F3|ABRAL_HUMAN	4	70.4	0.759263	NA	NA	NA	NA	NA	3	65.4	NA	NA	NA	NA	NA	NA	NA
1335	Q9P258	RCC2	Protein RCC2	sp|Q9P258|RCC2_HUMAN	13	37.9	-0.081703	0.77804	0.9991	-0.0505	0.86159	0.92074	13	33.5	0.35466	0.23105	0.9973	1.47236	######	0.0004	1
1336	Q9P287	BCCIP	BRCA2 and CDKN	sp|Q9P287|BCCIP_HUMAN	2	9.9	0.0507612	0.87576	0.9984	-0.0912	0.87576	0.99836	3	14.3	NA	NA	NA	NA	NA	NA	NA
1337	Q9P289	STK26	Serine/threonine	sp|Q9P289|STK26_HUMAN	5	14.4	0.0275134	0.88793	0.9984	-0.294	0.88793	0.99836	4	12.7	NA	NA	NA	NA	NA	NA	NA
1338	Q9P2E9	RRBP1	Ribosome-bindin	sp|Q9P2E9|RRBP1_HUMAN	30	37.4	0.0787725	0.7234	0.9984	0.4077	0.7234	0.99836	16	22.8	-0.2541	NA	NA	NA	NA	NA	NA
1339	Q9P2J5	LARS	Leucine--tRNA lig	sp|Q9P2J5|SYLC_HUMAN L	24	22.1	0.1223943	0.72814	0.9991	-0.0618	0.86059	0.92074	24	24.4	0.41127	0.25171	0.9973	0.97165	0.0125	0.0266	1
1340	Q9P2R7	SUCLA2	Succinate--CoA li	sp|Q9P2R7|SUCB1_HUMAN	4	8	-0.190161	0.72471	0.9991	0.9158	0.05438	0.18165	9	23.1	0.09394	0.79388	0.9973	-0.5924	0.1173	0.1793	NA

1341	Q9UBB4	ATXN10	Ataxin-10	sp|Q9UBB4|ATX10_HUMAI	3	8.2	-0.6824	0.27301	0.9991	-0.0533	0.92939	0.9536	11	28.8	-0.1312	0.78984	0.9973	1.72642	0.0031	0.0084	1
1342	Q9UBC3	DNMT3B	DNA (cytosine-5	sp|Q9UBC3|DNM3B_HUMA	12	17.8	0.0589872	0.72008	0.9984	0.566	0.72008	0.99836	NA	NA	NA	NA	NA	NA	NA	NA	NA
1343	Q9UBE0	SAE1	SUMO-activating	sp|Q9UBE0|SAE1_HUMAN	8	24.6	0.3476976	0.11639	0.9991	0.0767	0.71912	0.84693	9	32.1	0.50276	0.02878	0.9973	0.13235	0.5367	0.629	1
1344	Q9UBF2	COPG2	Coatomer subun	sp|Q9UBF2|COPG2_HUMA	4	5.9	-0.335143	NA	NA	NA	NA	NA	5	7.1	NA	NA	NA	NA	NA	NA	NA
1345	Q9UBQ0	VPS29	Vacuolar protei	sp|Q9UBQ0|VPS29_HUMA	1	7.1	NA	NA	NA	NA	NA	NA	3	19.8	0.67295	0.1203	0.9991	0.7035	0.1203	0.9991	NA
1346	Q9UBQ5	EIF3K	Eukaryotic trans	sp|Q9UBQ5|EIF3K_HUMAN	4	22.5	0.3924531	0.21687	0.9991	-0.085	0.78424	0.88823	5	27.5	0.2973	0.3447	0.9973	1.16061	0.0015	0.0046	1
1347	Q9UBS4	DNAJB11	DnaJ homolog su	sp|Q9UBS4|DJB11_HUMAN	3	10.9	NA	NA	NA	NA	NA	NA	9	32.1	-0.4294	0.14321	0.9991	-0.5721	0.1432	0.9991	NA
1348	Q9UBT2	UBA2	SUMO-activating	sp|Q9UBT2|SAE2_HUMAN	7	13.4	0.3276778	0.17342	0.9991	0.1337	0.56917	0.7271	12	28.4	0.26318	0.26941	0.9973	0.08312	0.7227	0.7823	NA
1349	Q9UDR5	AASS	Alpha-aminoadip	sp|Q9UDR5|AASS_HUMAN	12	18.1	0.0647776	0.69258	0.9984	0.5598	0.69258	0.99836	NA	NA	NA	NA	NA	NA	NA	NA	NA
1350	Q9UDY2	TJP2	Tight junction pr	sp|Q9UDY2|ZO2_HUMAN T	6	6.2	-0.02156	NA	NA	NA	NA	NA	3	2.8	NA	NA	NA	NA	NA	NA	NA
1351	Q9UG63	ABCF2	ATP-binding cass	sp|Q9UG63|ABCF2_HUMA	1	1.8	NA	NA	NA	NA	NA	NA	7	13	-0.0074	0.97668	0.9991	-0.3467	0.9767	0.9991	NA
1352	Q9UHB6	LIMA1	LIM domain and	sp|Q9UHB6|LIMA1_HUMA	4	5.8	NA	NA	NA	NA	NA	NA	7	10.9	NA	NA	NA	-0.3672	NA	NA	NA
1353	Q9UHB9	SRP68	Signal recogniti	sp|Q9UHB9|SRP68_HUMAI	8	17.5	0.0407962	0.89834	0.9991	-0.2828	0.43296	0.6164	9	18.3	0.01835	0.95417	0.9973	-0.5285	0.1126	0.1732	NA
1354	Q9UHD1	CHORDC1	Cysteine and his	sp|Q9UHD1|CHRD1_HUMA	7	25.9	0.3023245	NA	NA	NA	NA	NA	7	28.6	0.76547	0.03208	0.9991	1.62391	0.0321	0.9991	NA
1355	Q9UHD8	Sep-09	Septin-9	sp|Q9UHD8|SEPT9_HUMA	12	23.7	0.2198836	0.39599	0.9991	-0.3412	0.19452	0.37903	17	39.9	0.00891	0.97225	0.9973	-0.0459	0.8578	0.8839	NA
1356	Q9UHV9	PFDN2	Prefoldin subuni	sp|Q9UHV9|PFD2_HUMAN	4	31.2	0.5872601	NA	NA	NA	NA	NA	6	47.4	-0.1733	0.68881	0.9991	0.57498	0.6888	0.9991	NA
1357	Q9UHX1	PUF60	Poly(U)-binding-s	sp|Q9UHX1|PUF60_HUMA	11	30.4	0.0030259	0.98823	0.9991	0.5927	0.00955	0.06292	13	32.7	0.02506	0.90278	0.9973	-0.3561	0.0966	0.1553	5
1358	Q9UJQ4	SALL4	Sal-like protein 4	sp|Q9UJQ4|SALL4_HUMAN	7	9.4	0.6290107	0.07964	0.9984	1.0391	0.07964	0.99836	NA	NA	NA	NA	NA	NA	NA	NA	NA
1359	Q9UJS0	SLC25A13	Calcium-binding	sp|Q9UJS0|CMC2_HUMAN	9	16.4	-0.139445	0.61888	0.9991	0.4481	0.16246	0.33643	8	14.5	0.30798	0.48099	0.9973	1.51648	0.0012	0.0037	1
1360	Q9UJZ1	STOML2	Stomatin-like pr	sp|Q9UJZ1|STML2_HUMAI	9	42.1	0.2343504	0.36712	0.9984	0.7677	0.36712	0.99836	5	22.5	0.04067	NA	NA	NA	NA	NA	NA
1361	Q9UK76	JPT1	Jupiter microtub	sp|Q9UK76|JUPI1_HUMAN	3	39	-0.112505	0.72969	0.9991	-0.7198	0.13506	0.30186	8	68.8	0.24173	0.54645	0.9973	3.79967	######	######	2
1362	Q9UKD2	MRTO4	mRNA turnover	sp|Q9UKD2|MRT4_HUMAN	4	15.5	0.1325284	0.72041	0.9991	0.5715	0.13725	0.30314	4	22.2	-0.3162	0.23789	0.9973	-0.4685	0.0889	0.1442	NA
1363	Q9UKF6	CPSF3	Cleavage and po	sp|Q9UKF6|CPSF3_HUMAN	8	13	-0.054866	0.81783	0.9984	0.4182	0.81783	0.99836	6	10.2	-0.0109	NA	NA	NA	NA	NA	NA
1364	Q9UKK9	NUDT5	ADP-sugar pyrop	sp|Q9UKK9|NUDT5_HUMA	8	38.8	-0.213425	0.41192	0.9991	-0.8259	0.01037	0.06563	11	48.4	-0.7897	0.00685	0.7846	0.87214	0.0035	0.0092	2
1365	Q9UKM9	RALY	RNA-binding pro	sp|Q9UKM9|RALY_HUMAN	5	15.7	-0.172323	0.37972	0.9984	0.1169	0.37972	0.99836	6	20.6	NA	NA	NA	NA	NA	NA	NA
1366	Q9UKV3	ACIN1	Apoptotic chrom	sp|Q9UKV3|ACINU_HUMA	13	11.7	0.0119722	0.96868	0.9991	0.8141	0.01518	0.08154	8	5.6	0.27004	0.38149	0.9973	0.6497	0.0456	0.0801	1
1367	Q9UKX7	NUP50	Nuclear pore co	sp|Q9UKX7|NUP50_HUMA	6	16.2	-0.081708	0.85708	0.9984	-0.2542	0.85708	0.99836	9	26.9	NA	NA	NA	NA	NA	NA	NA
1368	Q9UKY7	CDV3	Protein CDV3 ho	sp|Q9UKY7|CDV3_HUMAN	2	8.5	NA	NA	NA	NA	NA	NA	6	24.4	-0.0796	0.82406	0.9991	0.17052	0.8241	0.9991	NA
1369	Q9UL46	PSME2	Proteasome acti	sp|Q9UL46|PSME2_HUMA	2	7.5	NA	NA	NA	NA	NA	NA	12	58.2	-0.5407	0.23265	0.9991	-1.4986	0.2326	0.9991	NA
1370	Q9ULV4	CORO1C	Coronin-1C	sp|Q9ULV4|COR1C_HUMAI	5	9.5	-0.070093	0.77166	0.9991	-0.0494	0.85485	0.92074	5	15.2	0.35898	0.19643	0.9973	0.32951	0.2336	0.3185	NA
1371	Q9UMS4	PRPF19	Pre-mRNA-proce	sp|Q9UMS4|PRP19_HUMA	6	14.9	0.2299387	0.41019	0.9991	0.1483	0.59299	0.7481	8	22.8	-0.1552	0.57621	0.9973	0.24438	0.3821	0.4761	NA
1372	Q9UN86	G3BP2	Ras GTPase-activ	sp|Q9UN86|G3BP2_HUMA	4	10.8	0.6588838	0.13814	0.9991	-0.1439	0.73782	0.85849	13	34	-0.0927	0.82903	0.9973	-0.047	0.9129	0.9246	NA
1373	Q9UNF0	PACSIN2	Protein kinase C	sp|Q9UNF0|PACN2_HUMA	8	19.8	0.0430615	0.82767	0.9991	-0.0426	0.82965	0.9155	5	13.6	0.01782	0.95207	0.9973	-0.281	0.217	0.3029	NA
1374	Q9UNF1	MAGED2	Melanoma-assoc	sp|Q9UNF1|MAGD2_HUM	10	24.9	0.168554	0.60094	0.9984	1.0841	0.60094	0.99836	3	7.6	NA	NA	NA	NA	NA	NA	NA
1375	Q9UNM6	PSMD13	26S proteasome	sp|Q9UNM6|PSD13_HUMA	8	22.6	0.0318109	0.864	0.9991	-0.5777	0.00593	0.04727	11	36.2	0.04232	0.81982	0.9973	0.04005	0.8293	0.8584	3
1376	Q9UNN5	FAF1	FAS-associated f	sp|Q9UNN5|FAF1_HUMAN	2	3.8	NA	NA	NA	NA	NA	NA	4	7.5	0.13115	0.66719	0.9991	0.60791	0.6672	0.9991	NA
1377	Q9UNS2	COPS3	COP9 signalosom	sp|Q9UNS2|CSN3_HUMAN	1	3.5	NA	NA	NA	NA	NA	NA	6	21	-0.2757	NA	NA	NA	NA	NA	NA
1378	Q9UNX4	WDR3	WD repeat-conta	sp|Q9UNX4|WDR3_HUMAI	4	5.7	-0.066117	0.83289	0.9984	0.7541	0.83289	0.99836	4	5.3	NA	NA	NA	NA	NA	NA	NA
1379	Q9UNZ2	NSFL1C	NSFL1 cofactor p	sp|Q9UNZ2|NSF1C_HUMA	7	25.9	0.3013779	0.29371	0.9991	-0.5195	0.11394	0.27928	8	29.2	-0.3116	0.27817	0.9973	-0.2349	0.4098	0.5044	NA
1380	Q9UPN3	MACF1	Microtubule-acti	sp|Q9UPN3|MACF1_HUMA	4	0.7	-0.155603	0.53774	0.9984	0.1649	0.53774	0.99836	10	2	NA	NA	NA	NA	NA	NA	NA
1381	Q9UQ35	SRRM2	Serine/arginine	sp|Q9UQ35|SRRM2_HUMA	4	2.8	0.0332732	0.8892	0.9984	1.0429	0.8892	0.99836	16	8.9	NA	NA	NA	NA	NA	NA	NA
1382	Q9UQ80	PA2G4	Proliferation-ass	sp|Q9UQ80|PA2G4_HUMA	14	43.1	0.2251901	0.26956	0.9991	0.1672	0.40825	0.60515	22	71.3	0.03123	0.87601	0.9973	-0.649	0.0045	0.0114	4
1383	Q9UQE7	SMC3	Structural maint	sp|Q9UQE7|SMC3_HUMAN	16	14.5	0.0445743	0.859	0.9991	0.3479	0.17766	0.35476	16	16.1	0.08743	0.72788	0.9973	-0.2469	0.332	0.4225	NA
1384	Q9Y224	RTRAF	RNA transcriptio	sp|Q9Y224|RTRAF_HUMAN	4	16.8	0.050625	0.90114	0.9991	0.272	0.54534	0.70555	12	57.4	0.69958	0.19047	0.9973	2.47596	######	######	1
1385	Q9Y230	RUVBL2	RuvB-like 2	sp|Q9Y230|RUVB2_HUMAI	11	30	0.2092625	0.26159	0.9991	-0.1534	0.40626	0.60515	16	43.8	-0.1412	0.44384	0.9973	0.73102	0.0009	0.0029	2
1386	Q9Y262	EIF3L	Eukaryotic trans	sp|Q9Y262|EIF3L_HUMAN	12	25	-0.350771	0.07366	0.9991	-0.4315	0.03157	0.12742	23	50	0.02297	0.90192	0.9973	-0.0883	0.6367	0.7188	3
1387	Q9Y263	PLAA	Phospholipase A	sp|Q9Y263|PLAP_HUMAN	6	9.8	-0.080457	0.67111	0.9984	-0.057	0.67111	0.99836	4	7.7	NA	NA	NA	NA	NA	NA	NA
1388	Q9Y265	RUVBL1	RuvB-like 1	sp|Q9Y265|RUVB1_HUMAI	12	34.2	0.0993665	0.59793	0.9991	-0.1549	0.41381	0.60623	18	57.2	0.02638	0.8882	0.9973	0.97298	######	0.0004	2

1389	Q9Y266	NUDC	Nuclear migratio	sp|Q9Y266|NUDC_HUMAN	16	51.4	0.1912736	0.34894	0.9991	-0.3614	0.0868	0.24019	18	51.4	-0.1294	0.52311	0.9973	0.46042	0.0335	0.0615	2
1390	Q9Y277	VDAC3	Voltage-depend	sp|Q9Y277|VDAC3_HUMA	6	31.8	-0.257004	0.23804	0.9984	0.4282	0.23804	0.99836	6	32.9	-0.0094	NA	NA	NA	NA	NA	NA
1391	Q9Y285	FARSA	Phenylalanine--t	sp|Q9Y285|SYFA_HUMAN	6	14	0.4749002	0.10062	0.9984	0.1742	0.10062	0.99836	8	21.5	NA	NA	NA	NA	NA	NA	NA
1392	Q9Y295	DRG1	Developmentally	sp|Q9Y295|DRG1_HUMAN	4	17.2	0.0063915	0.97514	0.9991	-0.0441	0.84775	0.91851	7	31.6	0.37366	0.08338	0.9973	0.08302	0.6864	0.7593	NA
1393	Q9Y2B0	CNPY2	Protein canopy h	sp|Q9Y2B0|CNPY2_HUMAN	2	15.9	NA	NA	NA	NA	NA	NA	8	53.8	0.0207	0.9742	0.9991	1.47698	0.9742	0.9991	NA
1394	Q9Y2L1	DIS3	Exosome comple	sp|Q9Y2L1|RRP44_HUMAN	5	6.5	-0.363307	NA	NA	NA	NA	NA	7	11.5	NA	NA	NA	NA	NA	NA	NA
1395	Q9Y2V2	CARHSP1	Calcium-regulate	sp|Q9Y2V2|CHSP1_HUMAN	4	40.1	-0.162038	0.7242	0.9991	-0.3156	0.54056	0.70193	3	34.7	0.15232	0.76658	0.9973	1.67733	0.0048	0.012	2
1396	Q9Y2W1	THRAP3	Thyroid hormone	sp|Q9Y2W1|TR150_HUMA	15	18.1	-0.042556	0.89897	0.9991	0.8378	0.02164	0.10155	14	18.1	0.11109	0.74067	0.9973	1.2118	0.002	0.0057	1
1397	Q9Y2W2	WBP11	WW domain-bind	sp|Q9Y2W2|WBP11_HUMA	4	8	-0.035621	0.89848	0.9991	0.2682	0.47843	0.65484	6	11.1	-0.0925	0.74096	0.9973	-0.011	0.9648	0.9723	NA
1398	Q9Y2X3	NOP58	Nucleolar protei	sp|Q9Y2X3|NOP58_HUMAN	11	27.2	0.2776327	0.36938	0.9991	1.1194	0.0018	0.02746	12	29.7	0.02891	0.92459	0.9973	-0.6133	0.0579	0.0992	5
1399	Q9Y2X9	ZNF281	Zinc finger prote	sp|Q9Y2X9|ZN281_HUMAN	5	8.6	NA	NA	NA	0.5678	NA	NA	NA	NA	NA	NA	NA	NA	NA	NA	NA
1400	Q9Y2Z0	SUGT1	Protein SGT1 ho	sp|Q9Y2Z0|SGT1_HUMAN	8	29.9	0.1948777	0.36053	0.9991	-0.2334	0.27633	0.47715	14	46.8	0.05329	0.82072	0.9973	0.35495	0.1061	0.1654	NA
1401	Q9Y305	ACOT9	Acyl-coenzyme A	sp|Q9Y305|ACOT9_HUMAN	3	7.3	-0.304645	0.21686	0.9984	0.282	0.21686	0.99836	NA	NA	NA	NA	NA	NA	NA	NA	NA
1402	Q9Y333	LSM2	U6 snRNA-associ	sp|Q9Y333|LSM2_HUMAN	3	32.6	0.3818189	0.20429	0.9984	0.113	0.20429	0.99836	2	31.6	NA	NA	NA	NA	NA	NA	NA
1403	Q9Y383	LUC7L2	Putative RNA-bi	sp|Q9Y383|LC7L2_HUMAN	4	13.3	0.6693444	0.13681	0.9991	0.5627	0.20594	0.39356	9	26.5	0.06757	0.82516	0.9973	0.53137	0.0981	0.1566	NA
1404	Q9Y3B4	SF3B6	Splicing factor 3	sp|Q9Y3B4|SF3B6_HUMAN	4	34.4	-0.454054	0.43204	0.9984	0.2222	0.43204	0.99836	4	34.4	NA	NA	NA	0.90841	NA	NA	NA
1405	Q9Y3C6	PPIL1	Peptidyl-prolyl c	sp|Q9Y3C6|PPIL1_HUMAN	2	18.7	-0.543021	NA	NA	NA	NA	NA	3	25.3	0.31854	0.1771	0.9991	0.88692	0.1771	0.9991	NA
1406	Q9Y3D9	MRPS23	28S ribosomal p	sp|Q9Y3D9|RT23_HUMAN	3	17.4	-0.880761	0.26526	0.9984	0.851	0.26526	0.99836	5	30.5	NA	NA	NA	NA	NA	NA	NA
1407	Q9Y3F4	STRAP	Serine-threonine	sp|Q9Y3F4|STRAP_HUMAN	12	46.9	0.4492951	0.24685	0.9991	-0.8559	0.03577	0.13782	14	53.4	-0.0946	0.80353	0.9973	0.44566	0.2505	0.3339	2
1408	Q9Y3I0	RTCB	tRNA-splicing lig	sp|Q9Y3I0|RTCB_HUMAN t	12	33.5	0.1174597	0.5791	0.9991	-0.0136	0.94867	0.96777	17	43	0.09317	0.65937	0.9973	-0.1743	0.413	0.5071	NA
1409	Q9Y3T9	NOC2L	Nucleolar comple	sp|Q9Y3T9|NOC2L_HUMAN	3	4.4	0.1423093	NA	NA	NA	NA	NA	5	5.9	NA	NA	NA	NA	NA	NA	NA
1410	Q9Y3Y2	CHTOP	Chromatin targe	sp|Q9Y3Y2|CHTOP_HUMAN	3	18.5	0.6107747	0.11243	0.9984	0.6817	0.11243	0.99836	2	13.3	-0.2084	NA	NA	NA	NA	NA	NA
1411	Q9Y467	SALL2	Sal-like protein	sp|Q9Y467|SALL2_HUMAN	6	12	-0.040524	0.87012	0.9984	1.2641	0.87012	0.99836	NA	NA	NA	NA	NA	NA	NA	NA	NA
1412	Q9Y490	TLN1	Talin-1	sp|Q9Y490|TLN1_HUMAN T	21	10.7	0.1377982	0.61825	0.9991	-1.0219	0.00163	0.02746	40	23.3	0.08593	0.75542	0.9973	-2.0711	######	######	4
1413	Q9Y4L1	HYOU1	Hypoxia up-regu	sp|Q9Y4L1|HYOU1_HUMAN	24	33.8	0.1616135	0.43243	0.9991	0.3383	0.11097	0.279	23	34.9	-0.0555	0.78585	0.9973	-2.0943	######	######	4
1414	Q9Y4W2	LAS1L	Ribosomal bioge	sp|Q9Y4W2|LAS1L_HUMAN	3	6.3	-1.818271	NA	NA	NA	NA	NA	NA	NA	NA	NA	NA	NA	NA	NA	NA
1415	Q9Y4W6	AFG3L2	AFG3-like protei	sp|Q9Y4W6|AFG32_HUMA	6	8.3	-0.561934	0.13579	0.9984	0.3043	0.13579	0.99836	3	6.1	NA	NA	NA	NA	NA	NA	NA
1416	Q9Y4Z0	LSM4	U6 snRNA-associ	sp|Q9Y4Z0|LSM4_HUMAN	2	14.4	0.5718934	0.34865	0.9991	0.1417	0.83262	0.91666	3	21.6	0.53408	0.55515	0.9973	0.213	0.8017	0.8396	NA
1417	Q9Y512	SAMM50	Sorting and asse	sp|Q9Y512|SAM50_HUMA	5	12.2	-0.181307	0.44588	0.9984	0.4019	0.44588	0.99836	2	6.6	NA	NA	NA	NA	NA	NA	NA
1418	Q9Y520	PRRC2C	Protein PRRC2C	sp|Q9Y520|PRC2C_HUMAN	14	6.5	-0.017763	0.95984	0.9991	0.1327	0.70737	0.84008	12	5.8	0.10239	0.79536	0.9973	0.09769	0.8046	0.8413	NA
1419	Q9Y5A9	YTHDF2	YTH domain-con	sp|Q9Y5A9|YTHD2_HUMA	6	11.4	0.2281611	0.47729	0.9991	0.2718	0.39868	0.60515	9	19	-0.1988	0.5351	0.9973	-0.0759	0.8119	0.8465	NA
1420	Q9Y5B9	SUPT16H	FACT complex su	sp|Q9Y5B9|SP16H_HUMAN	18	19.7	0.0144997	0.94411	0.9991	0.0292	0.88775	0.93452	15	17.2	-0.0073	0.97179	0.9973	0.54214	0.0168	0.0348	1
1421	Q9Y5J9	TIMM8B	Mitochondrial im	sp|Q9Y5J9|TIM8B_HUMAN	2	25.3	-0.464544	0.27556	0.9984	-0.0892	0.27556	0.99836	3	33.7	NA	NA	NA	NA	NA	NA	NA
1422	Q9Y5K5	UCHL5	Ubiquitin carbox	sp|Q9Y5K5|UCHL5_HUMAN	1	3.3	NA	NA	NA	NA	NA	NA	6	20.7	-0.2418	0.28394	0.9991	0.39709	0.2839	0.9991	NA
1423	Q9Y5K6	CD2AP	CD2-associated p	sp|Q9Y5K6|CD2AP_HUMAN	3	6.7	0.8149763	NA	NA	NA	NA	NA	4	6.6	NA	NA	NA	NA	NA	NA	NA
1424	Q9Y5L0	TNPO3	Transportin-3	sp|Q9Y5L0|TNPO3_HUMAN	2	2.2	-0.073818	NA	NA	NA	NA	NA	3	4.8	NA	NA	NA	NA	NA	NA	NA
1425	Q9Y5L4	TIMM13	Mitochondrial im	sp|Q9Y5L4|TIM13_HUMAN	2	28.4	-0.957385	0.00976	0.9984	-1.1631	0.00976	0.99836	4	53.7	NA	NA	NA	NA	NA	NA	NA
1426	Q9Y5M8	SRPRB	Signal recognitio	sp|Q9Y5M8|SRPRB_HUMA	5	22.1	0.7849735	NA	NA	NA	NA	NA	6	28.8	NA	NA	NA	NA	NA	NA	NA
1427	Q9Y5S9	RBM8A	RNA-binding pro	sp|Q9Y5S9|RBM8A_HUMA	4	36.8	0.4338247	0.05478	0.9991	0.384	0.08537	0.238	5	50.6	0.06018	0.77776	0.9973	0.08034	0.7066	0.7767	NA
1428	Q9Y605	MRFAP1	MORF4 family-as	sp|Q9Y605|MOFA1_HUMA	2	28.3	0.1223033	0.76664	0.9984	0.3327	0.76664	0.99836	2	26.8	NA	NA	NA	NA	NA	NA	NA
1429	Q9Y617	PSAT1	Phosphoserine a	sp|Q9Y617|SERC_HUMAN	7	20.3	0.547107	0.09932	0.9991	-0.1218	0.73161	0.85314	7	22.4	0.04577	0.88523	0.9973	-0.5543	0.0953	0.1536	NA
1430	Q9Y678	COPG1	Coatomer subun	sp|Q9Y678|COPG1_HUMAN	4	5.4	NA	NA	NA	NA	NA	NA	18	27.9	0.10975	0.71579	0.9991	-1.3445	0.7158	0.9991	NA
1431	Q9Y696	CLIC4	Chloride intrace	sp|Q9Y696|CLIC4_HUMAN	10	42.3	0.0718756	0.6948	0.9991	-0.8729	0.00017	0.00845	15	64.8	0.10059	0.5838	0.9973	-0.8129	0.0003	0.0013	3
1432	Q9Y6A4	CFAP20	Cilia- and flagella	sp|Q9Y6A4|CFA20_HUMAN	2	10.4	NA	NA	NA	NA	NA	NA	2	10.4	1.33913	0.01513	0.9991	1.32015	0.0151	0.9991	NA
1433	Q9Y6A5	TACC3	Transforming ac	sp|Q9Y6A5|TACC3_HUMAN	6	9.4	0.069217	0.77586	0.9984	0.6446	0.77586	0.99836	5	8.5	NA	NA	NA	NA	NA	NA	NA
1434	Q9Y6A9	SPCS1	Signal peptidase	sp|Q9Y6A9|SPCS1_HUMAN	2	26.5	0.0109733	0.95593	0.999	0.217	0.95593	0.99899	2	26.5	NA	NA	NA	NA	NA	NA	NA
1435	Q9Y6C9	MTCH2	Mitochondrial ca	sp|Q9Y6C9|MTCH2_HUMA	5	23.8	-0.017472	0.93766	0.999	0.7484	0.93766	0.99899	3	16.8	0.02829	NA	NA	NA	NA	NA	NA
1436	Q9Y6E2	BZW2	Basic leucine zip	sp|Q9Y6E2|BZW2_HUMAN	5	9.3	0.3444674	0.36146	0.9991	-0.0916	0.78757	0.88965	8	15	-0.2941	0.34054	0.9973	-0.3665	0.2391	0.3235	NA

1437	Q9Y6K1	DNMT3A	DNA (cytosine-5	sp|Q9Y6K1|DNM3A_HUMA	6	8.9	-0.222284	0.30541	0.9984	0.2803	0.30541	0.99836	2	3.4	NA	NA	NA	NA	NA	NA	NA
1438	Q9Y6M1	IGF2BP2	Insulin-like grow	sp|Q9Y6M1|IF2B2_HUMAN	8	17	0.5693228	0.16174	0.9984	0.965	0.16174	0.99836	6	13.4	0.08861	NA	NA	NA	NA	NA	NA
1439	O00170	AIP	AH receptor-inte	sp|O00170|AIP_HUMAN A	NA	NA	NA	NA	NA	NA	NA	NA	6	26.1	-0.1379	0.62896	0.9991	0.43341	0.629	0.9991	NA
1440	O00469	PLOD2	Procollagen-lysin	sp|O00469|PLOD2_HUMAN	NA	NA	NA	NA	NA	NA	NA	NA	4	7.5	-0.3718	NA	NA	NA	NA	NA	NA
1441	O00499	BIN1	Myc box-depend	sp|O00499|BIN1_HUMAN M	NA	NA	NA	NA	NA	NA	NA	NA	13	32.5	-0.4086	0.68281	0.9991	1.68613	0.6828	0.9991	NA
1442	O43353	RIPK2	Receptor-interac	sp|O43353|RIPK2_HUMAN	NA	NA	NA	NA	NA	NA	NA	NA	3	9.1	0.28382	NA	NA	NA	NA	NA	NA
1443	O60493	SNX3	Sorting nexin-3	sp|O60493|SNX3_HUMAN	NA	NA	NA	NA	NA	NA	NA	NA	4	24.1	0.23072	0.34743	0.9991	1.42785	0.3474	0.9991	NA
1444	O60568	PLOD3	Multifunctional	sp|O60568|PLOD3_HUMAN	NA	NA	NA	NA	NA	NA	NA	NA	5	8.7	0.19517	0.32795	0.9991	0.36708	0.328	0.9991	NA
1445	O75116	ROCK2	Rho-associated p	sp|O75116|ROCK2_HUMAN	NA	NA	NA	NA	NA	NA	NA	NA	10	9.8	0.11257	NA	NA	NA	NA	NA	NA
1446	O75306	NDUFS2	NADH dehydrog	sp|O75306|NDUS2_HUMA	NA	NA	NA	NA	NA	NA	NA	NA	3	8.2	0.09839	NA	NA	NA	NA	NA	NA
1447	O75746	SLC25A12	Calcium-binding	sp|O75746|CMC1_HUMAN	NA	NA	NA	NA	NA	NA	NA	NA	5	8.4	-0.1334	NA	NA	NA	NA	NA	NA
1448	O94979	SEC31A	Protein transpor	sp|O94979|SC31A_HUMAN	NA	NA	NA	NA	NA	NA	NA	NA	9	7.4	0.64186	0.31406	0.9991	0.66722	0.3141	0.9991	NA
1449	O95817	BAG3	BAG family mole	sp|O95817|BAG3_HUMAN	NA	NA	NA	NA	NA	NA	NA	NA	10	23	-0.29	0.43604	0.9991	0.45584	0.436	0.9991	NA
1450	O95834	EML2	Echinoderm micr	sp|O95834|EMAL2_HUMA	NA	NA	NA	NA	NA	NA	NA	NA	4	5.7	0.89061	NA	NA	NA	NA	NA	NA
1451	P04083	ANXA1	Annexin A1	sp|P04083|ANXA1_HUMAN	NA	NA	NA	NA	NA	NA	NA	NA	4	13.6	0.0265	NA	NA	NA	NA	NA	NA
1452	P06703	S100A6	Protein S100-A6	sp|P06703|S10A6_HUMAN	NA	NA	NA	NA	NA	NA	NA	NA	3	35.6	-0.0846	NA	NA	NA	NA	NA	NA
1453	P07197	NEFM	Neurofilament m	sp|P07197|NFM_HUMAN N	NA	NA	NA	NA	NA	NA	NA	NA	30	34.8	0.74486	0.59248	0.9991	5.37379	0.5925	0.9991	NA
1454	P07305	H1F0	Histone H1.0	sp|P07305|H10_HUMAN H	NA	NA	NA	NA	NA	NA	NA	NA	2	11.9	NA	NA	NA	-1.1137	NA	NA	NA
1455	P08648	ITGA5	Integrin alpha-5	sp|P08648|ITA5_HUMAN I	NA	NA	NA	NA	NA	NA	NA	NA	2	2.8	-0.0338	NA	NA	NA	NA	NA	NA
1456	P09382	LGALS1	Galectin-1	sp|P09382|LEG1_HUMAN G	NA	NA	NA	NA	NA	NA	NA	NA	9	70.4	0.33314	0.36029	0.9991	0.83649	0.3603	0.9991	NA
1457	P09486	SPARC	SPARC	sp|P09486|SPRC_HUMAN S	NA	NA	NA	NA	NA	NA	NA	NA	5	16.8	0.58681	0.10505	0.9991	1.01351	0.1051	0.9991	NA
1458	P11234	RALB	Ras-related prot	sp|P11234|RALB_HUMAN R	NA	NA	NA	NA	NA	NA	NA	NA	4	19.9	0.61475	NA	NA	NA	NA	NA	NA
1459	P13591	NCAM1	Neural cell adhe	sp|P13591|NCAM1_HUMA	NA	NA	NA	NA	NA	NA	NA	NA	3	4.7	-0.2021	NA	NA	NA	NA	NA	NA
1460	P13929	ENO3	Beta-enolase	sp|P13929|ENOB_HUMAN	NA	NA	NA	NA	NA	NA	NA	NA	8	27	0.2686	NA	NA	NA	NA	NA	NA
1461	P15090	FABP4	Fatty acid-bindir	sp|P15090|FABP4_HUMAN	NA	NA	NA	NA	NA	NA	NA	NA	4	28.8	-0.0743	NA	NA	NA	NA	NA	NA
1462	P16070	CD44	CD44 antigen	sp|P16070|CD44_HUMAN C	NA	NA	NA	NA	NA	NA	NA	NA	2	2.4	-0.1162	NA	NA	NA	NA	NA	NA
1463	P20711	DDC	Aromatic-L-amin	sp|P20711|DDC_HUMAN A	NA	NA	NA	NA	NA	NA	NA	NA	5	12.5	NA	NA	NA	3.11209	NA	NA	NA
1464	P21266	GSTM3	Glutathione S-tr	sp|P21266|GSTM3_HUMA	NA	NA	NA	NA	NA	NA	NA	NA	7	36.9	0.37976	0.25106	0.9991	-0.2992	0.2511	0.9991	NA
1465	P35321	SPRR1A	Cornifin-A	sp|P35321|SPR1A_HUMAN	NA	NA	NA	NA	NA	NA	NA	NA	2	27	1.02267	NA	NA	NA	NA	NA	NA
1466	Q9NRH3	TUBG2	Tubulin gamma-	sp|Q9NRH3|TBG2_HUMAN	NA	NA	NA	NA	NA	NA	NA	NA	4	14	0.29631	0.57544	0.9991	0.55774	0.5754	0.9991	NA
1467	P29317	EPHA2	Ephrin type-A re	sp|P29317|EPHA2_HUMAN	NA	NA	NA	NA	NA	NA	NA	NA	2	2.3	-0.1675	NA	NA	NA	NA	NA	NA
1468	P30740	SERPINB1	Leukocyte elasta	sp|P30740|ILEU_HUMAN L	NA	NA	NA	NA	NA	NA	NA	NA	4	7.7	-0.3741	NA	NA	NA	NA	NA	NA
1469	Q9H0L4	CSTF2T	Cleavage stimula	sp|Q9H0L4|CSTFT_HUMAN	3	6.8	NA	NA	NA	NA	NA	NA	7	16.9	-0.0335	0.96074	0.9991	1.45532	0.9607	0.9991	NA
1470	Q96A05	ATP6V1E2	V-type proton A	sp|Q96A05|VATE2_HUMA	2	9.7	NA	NA	NA	NA	NA	NA	5	23	-0.3579	0.23738	0.9991	0.66514	0.2374	0.9991	NA
1471	P41219	PRPH	Peripherin	sp|P41219|PERI_HUMAN P	NA	NA	NA	NA	NA	NA	NA	NA	28	55.7	NA	NA	NA	4.54205	NA	NA	NA
1472	P46934	NEDD4	E3 ubiquitin-pro	sp|P46934|NEDD4_HUMAN	NA	NA	NA	NA	NA	NA	NA	NA	7	7.4	-0.0556	NA	NA	NA	NA	NA	NA
1473	P48507	GCLM	Glutamate--cyst	sp|P48507|GSH0_HUMAN	NA	NA	NA	NA	NA	NA	NA	NA	5	20.8	0.00373	0.9879	0.9991	-0.7496	0.9879	0.9991	NA
1474	P50993	ATP1A2	Sodium/potassiu	sp|P50993|AT1A2_HUMAN	NA	NA	NA	NA	NA	NA	NA	NA	13	16.4	-0.2	NA	NA	NA	NA	NA	NA
1475	P51965	UBE2E1	Ubiquitin-conjug	sp|P51965|UB2E1_HUMAN	NA	NA	NA	NA	NA	NA	NA	NA	2	15	0.1003	0.83473	0.9991	-0.4329	0.8347	0.9991	NA
1476	Q15366	PCBP2	Poly(rC)-binding	sp|Q15366|PCBP2_HUMAN	12	41.6	-0.651205	0.31484	0.9984	0.6534	0.31484	0.99836	10	40	NA	NA	NA	NA	NA	NA	NA
1477	P62987	UBA52	Ubiquitin-60S rib	sp|P62987|RL40_HUMAN U	6	43	-0.004137	0.98076	0.9991	0.2771	0.12011	0.2853	7	47.4	0.16015	0.35703	0.9973	-0.2451	0.1658	0.2429	NA
1478	P04899	GNAI2	Guanine nucleot	sp|P04899|GNAI2_HUMAN	8	26.2	0.1150815	0.52734	0.9984	0.6046	0.52734	0.99836	9	32.1	NA	NA	NA	NA	NA	NA	NA
1479	Q71DI3	HIST2H3A	Histone H3.2	sp|Q71DI3|H32_HUMAN H	8	53.7	0.2881617	0.50946	0.9984	0.7242	0.50946	0.99836	5	42.6	NA	NA	NA	NA	NA	NA	NA
1480	P98082	DAB2	Disabled homolo	sp|P98082|DAB2_HUMAN	NA	NA	NA	NA	NA	NA	NA	NA	5	7.4	0.058	NA	NA	NA	NA	NA	NA
1481	Q00403	GTF2B	Transcription ini	sp|Q00403|TF2B_HUMAN T	NA	NA	NA	NA	NA	NA	NA	NA	3	12	0.19911	NA	NA	NA	NA	NA	NA
1482	Q00534	CDK6	Cyclin-dependen	sp|Q00534|CDK6_HUMAN	NA	NA	NA	NA	NA	NA	NA	NA	8	31	0.29334	NA	NA	NA	NA	NA	NA
1483	Q00577	PURA	Transcriptional a	sp|Q00577|PURA_HUMAN	NA	NA	NA	NA	NA	NA	NA	NA	2	11.2	-0.2477	NA	NA	NA	NA	NA	NA
1484	Q02809	PLOD1	Procollagen-lysin	sp|Q02809|PLOD1_HUMAN	NA	NA	NA	NA	NA	NA	NA	NA	6	11	0.13884	NA	NA	NA	NA	NA	NA

<table>
<tr><td>1485</td><td>Q03135</td><td>CAV1</td><td>Caveolin-1</td><td>sp|Q03135|CAV1_HUMAN</td><td>NA</td><td>NA</td><td>NA</td><td>NA</td><td>NA</td><td>NA</td><td>NA</td><td>NA</td><td>4</td><td>22.5</td><td>0.03067</td><td>NA</td><td>NA</td><td>NA</td><td>NA</td><td>NA</td><td>NA</td></tr>
<tr><td>1486</td><td>Q09666</td><td>AHNAK</td><td>Neuroblast diffe</td><td>sp|Q09666|AHNK_HUMAN</td><td>NA</td><td>NA</td><td>NA</td><td>NA</td><td>NA</td><td>NA</td><td>NA</td><td>NA</td><td>123</td><td>45.3</td><td>0.25946</td><td>0.44341</td><td>0.9991</td><td>-0.8413</td><td>0.4434</td><td>0.9991</td><td>NA</td></tr>
<tr><td>1487</td><td>Q12792</td><td>TWF1</td><td>Twinfilin-1</td><td>sp|Q12792|TWF1_HUMAN</td><td>NA</td><td>NA</td><td>NA</td><td>NA</td><td>NA</td><td>NA</td><td>NA</td><td>NA</td><td>6</td><td>21.4</td><td>0.15341</td><td>0.56117</td><td>0.9991</td><td>-0.1504</td><td>0.5612</td><td>0.9991</td><td>NA</td></tr>
<tr><td>1488</td><td>Q13188</td><td>STK3</td><td>Serine/threonin</td><td>sp|Q13188|STK3_HUMAN</td><td>2</td><td>8.4</td><td>NA</td><td>NA</td><td>NA</td><td>NA</td><td>NA</td><td>NA</td><td>NA</td><td>NA</td><td>NA</td><td>NA</td><td>NA</td><td>NA</td><td>NA</td><td>NA</td><td>NA</td></tr>
<tr><td>1489</td><td>Q13155</td><td>AIMP2</td><td>Aminoacyl tRNA</td><td>sp|Q13155|AIMP2_HUMAN</td><td>NA</td><td>NA</td><td>NA</td><td>NA</td><td>NA</td><td>NA</td><td>NA</td><td>NA</td><td>6</td><td>27.5</td><td>NA</td><td>NA</td><td>NA</td><td>0.05273</td><td>NA</td><td>NA</td><td>NA</td></tr>
<tr><td>1490</td><td>Q13322</td><td>GRB10</td><td>Growth factor re</td><td>sp|Q13322|GRB10_HUMAN</td><td>NA</td><td>NA</td><td>NA</td><td>NA</td><td>NA</td><td>NA</td><td>NA</td><td>NA</td><td>2</td><td>5.1</td><td>-0.2496</td><td>NA</td><td>NA</td><td>NA</td><td>NA</td><td>NA</td><td>NA</td></tr>
<tr><td>1491</td><td>Q14562</td><td>DHX8</td><td>ATP-dependent</td><td>sp|Q14562|DHX8_HUMAN</td><td>NA</td><td>NA</td><td>NA</td><td>NA</td><td>NA</td><td>NA</td><td>NA</td><td>NA</td><td>5</td><td>5.2</td><td>-0.024</td><td>NA</td><td>NA</td><td>NA</td><td>NA</td><td>NA</td><td>NA</td></tr>
<tr><td>1492</td><td>Q14764</td><td>MVP</td><td>Major vault prot</td><td>sp|Q14764|MVP_HUMAN</td><td>NA</td><td>NA</td><td>NA</td><td>NA</td><td>NA</td><td>NA</td><td>NA</td><td>NA</td><td>5</td><td>7.4</td><td>0.31747</td><td>NA</td><td>NA</td><td>NA</td><td>NA</td><td>NA</td><td>NA</td></tr>
<tr><td>1493</td><td>Q15052</td><td>ARHGEF6</td><td>Rho guanine nuc</td><td>sp|Q15052|ARHG6_HUMA</td><td>NA</td><td>NA</td><td>NA</td><td>NA</td><td>NA</td><td>NA</td><td>NA</td><td>NA</td><td>2</td><td>3</td><td>NA</td><td>NA</td><td>NA</td><td>-0.8735</td><td>NA</td><td>NA</td><td>NA</td></tr>
<tr><td>1494</td><td>Q15102</td><td>PAFAH1B3</td><td>Platelet-activati</td><td>sp|Q15102|PA1B3_HUMAN</td><td>NA</td><td>NA</td><td>NA</td><td>NA</td><td>NA</td><td>NA</td><td>NA</td><td>NA</td><td>10</td><td>69.7</td><td>-0.0742</td><td>NA</td><td>NA</td><td>NA</td><td>NA</td><td>NA</td><td>NA</td></tr>
<tr><td>1495</td><td>P62491</td><td>RAB11A</td><td>Ras-related prot</td><td>sp|P62491|RB11A_HUMAN</td><td>8</td><td>37</td><td>-0.049649</td><td>0.81528</td><td>0.9991</td><td>-0.0755</td><td>0.72279</td><td>0.84844</td><td>10</td><td>49.1</td><td>-0.4276</td><td>0.05732</td><td>0.9973</td><td>0.37925</td><td>0.0881</td><td>0.1435</td><td>NA</td></tr>
<tr><td>1496</td><td>Q16222</td><td>UAP1</td><td>UDP-N-acetylhex</td><td>sp|Q16222|UAP1_HUMAN</td><td>NA</td><td>NA</td><td>NA</td><td>NA</td><td>NA</td><td>NA</td><td>NA</td><td>NA</td><td>6</td><td>16.3</td><td>0.00298</td><td>NA</td><td>NA</td><td>NA</td><td>NA</td><td>NA</td><td>NA</td></tr>
<tr><td>1497</td><td>Q16777</td><td>HIST2H2AC</td><td>Histone H2A typ</td><td>sp|Q16777|H2A2C_HUMA</td><td>NA</td><td>NA</td><td>NA</td><td>NA</td><td>NA</td><td>NA</td><td>NA</td><td>NA</td><td>6</td><td>55</td><td>0.08528</td><td>0.81574</td><td>0.9991</td><td>2.37984</td><td>0.8157</td><td>0.9991</td><td>NA</td></tr>
<tr><td>1498</td><td>Q16881</td><td>TXNRD1</td><td>Thioredoxin redu</td><td>sp|Q16881|TRXR1_HUMAN</td><td>NA</td><td>NA</td><td>NA</td><td>NA</td><td>NA</td><td>NA</td><td>NA</td><td>NA</td><td>10</td><td>19.7</td><td>-0.4534</td><td>0.14691</td><td>0.9991</td><td>-0.2543</td><td>0.1469</td><td>0.9991</td><td>NA</td></tr>
<tr><td>1499</td><td>Q4G0F5</td><td>VPS26B</td><td>Vacuolar protei</td><td>sp|Q4G0F5|VP26B_HUMA</td><td>NA</td><td>NA</td><td>NA</td><td>NA</td><td>NA</td><td>NA</td><td>NA</td><td>NA</td><td>4</td><td>15.8</td><td>0.33413</td><td>NA</td><td>NA</td><td>NA</td><td>NA</td><td>NA</td><td>NA</td></tr>
<tr><td>1500</td><td>P63092</td><td>GNAS</td><td>Guanine nucleot</td><td>sp|P63092|GNAS2_HUMA</td><td>2</td><td>6.1</td><td>NA</td><td>NA</td><td>NA</td><td>NA</td><td>NA</td><td>NA</td><td>12</td><td>16.5</td><td>-0.0191</td><td>0.93922</td><td>0.9991</td><td>2.08617</td><td>0.9392</td><td>0.9991</td><td>NA</td></tr>
<tr><td>1501</td><td>Q5VW32</td><td>BROX</td><td>BRO1 domain-co</td><td>sp|Q5VW32|BROX_HUMAN</td><td>NA</td><td>NA</td><td>NA</td><td>NA</td><td>NA</td><td>NA</td><td>NA</td><td>NA</td><td>3</td><td>11.2</td><td>0.23062</td><td>NA</td><td>NA</td><td>NA</td><td>NA</td><td>NA</td><td>NA</td></tr>
<tr><td>1502</td><td>Q6NZI2</td><td>CAVIN1</td><td>Caveolae-associ</td><td>sp|Q6NZI2|CAVN1_HUMA</td><td>NA</td><td>NA</td><td>NA</td><td>NA</td><td>NA</td><td>NA</td><td>NA</td><td>NA</td><td>9</td><td>25.6</td><td>0.12768</td><td>NA</td><td>NA</td><td>NA</td><td>NA</td><td>NA</td><td>NA</td></tr>
<tr><td>1503</td><td>Q86Y82</td><td>STX12</td><td>Syntaxin-12</td><td>sp|Q86Y82|STX12_HUMAN</td><td>NA</td><td>NA</td><td>NA</td><td>NA</td><td>NA</td><td>NA</td><td>NA</td><td>NA</td><td>4</td><td>19.9</td><td>-0.0389</td><td>NA</td><td>NA</td><td>NA</td><td>NA</td><td>NA</td><td>NA</td></tr>
<tr><td>1504</td><td>Q8IVD9</td><td>NUDCD3</td><td>NudC domain-co</td><td>sp|Q8IVD9|NUDC3_HUMA</td><td>NA</td><td>NA</td><td>NA</td><td>NA</td><td>NA</td><td>NA</td><td>NA</td><td>NA</td><td>7</td><td>26.3</td><td>NA</td><td>NA</td><td>NA</td><td>0.48942</td><td>NA</td><td>NA</td><td>NA</td></tr>
<tr><td>1505</td><td>Q8IWT0</td><td>ZBTB8OS</td><td>Protein archease</td><td>sp|Q8IWT0|ARCH_HUMAN</td><td>NA</td><td>NA</td><td>NA</td><td>NA</td><td>NA</td><td>NA</td><td>NA</td><td>NA</td><td>3</td><td>21</td><td>-0.074</td><td>NA</td><td>NA</td><td>NA</td><td>NA</td><td>NA</td><td>NA</td></tr>
<tr><td>1506</td><td>P15170</td><td>GSPT1</td><td>Eukaryotic pepti</td><td>sp|P15170|ERF3A_HUMAN</td><td>8</td><td>21.8</td><td>0.0908704</td><td>0.63465</td><td>0.9984</td><td>-0.2196</td><td>0.63465</td><td>0.99836</td><td>13</td><td>30.7</td><td>NA</td><td>NA</td><td>NA</td><td>NA</td><td>NA</td><td>NA</td><td>NA</td></tr>
<tr><td>1507</td><td>Q8N392</td><td>ARHGAP18</td><td>Rho GTPase-acti</td><td>sp|Q8N392|RHG18_HUMA</td><td>NA</td><td>NA</td><td>NA</td><td>NA</td><td>NA</td><td>NA</td><td>NA</td><td>NA</td><td>2</td><td>3.9</td><td>-0.0192</td><td>NA</td><td>NA</td><td>NA</td><td>NA</td><td>NA</td><td>NA</td></tr>
<tr><td>1508</td><td>Q8NBJ5</td><td>COLGALT1</td><td>Procollagen gala</td><td>sp|Q8NBJ5|GT251_HUMAN</td><td>NA</td><td>NA</td><td>NA</td><td>NA</td><td>NA</td><td>NA</td><td>NA</td><td>NA</td><td>5</td><td>9.3</td><td>0.1511</td><td>0.65626</td><td>0.9991</td><td>-0.048</td><td>0.6563</td><td>0.9991</td><td>NA</td></tr>
<tr><td>1509</td><td>P61019</td><td>RAB2A</td><td>Ras-related prot</td><td>sp|P61019|RAB2A_HUMAN</td><td>6</td><td>34.4</td><td>0.0548909</td><td>0.79733</td><td>0.9984</td><td>0.1367</td><td>0.79733</td><td>0.99836</td><td>6</td><td>34.4</td><td>NA</td><td>NA</td><td>NA</td><td>NA</td><td>NA</td><td>NA</td><td>NA</td></tr>
<tr><td>1510</td><td>O75886</td><td>STAM2</td><td>Signal transduci</td><td>sp|O75886|STAM2_HUMA</td><td>1</td><td>1.9</td><td>NA</td><td>NA</td><td>NA</td><td>NA</td><td>NA</td><td>NA</td><td>NA</td><td>NA</td><td>NA</td><td>NA</td><td>NA</td><td>NA</td><td>NA</td><td>NA</td><td>NA</td></tr>
<tr><td>1511</td><td>Q92882</td><td>OSTF1</td><td>Osteoclast-stimu</td><td>sp|Q92882|OSTF1_HUMAN</td><td>NA</td><td>NA</td><td>NA</td><td>NA</td><td>NA</td><td>NA</td><td>NA</td><td>NA</td><td>2</td><td>17.3</td><td>0.03689</td><td>NA</td><td>NA</td><td>NA</td><td>NA</td><td>NA</td><td>NA</td></tr>
<tr><td>1512</td><td>Q96C19</td><td>EFHD2</td><td>EF-hand domain-</td><td>sp|Q96C19|EFHD2_HUMA</td><td>NA</td><td>NA</td><td>NA</td><td>NA</td><td>NA</td><td>NA</td><td>NA</td><td>NA</td><td>7</td><td>34.6</td><td>0.18202</td><td>0.66183</td><td>0.9991</td><td>0.43719</td><td>0.6618</td><td>0.9991</td><td>NA</td></tr>
<tr><td>1513</td><td>Q96EY1</td><td>DNAJA3</td><td>DnaJ homolog su</td><td>sp|Q96EY1|DNJA3_HUMA</td><td>NA</td><td>NA</td><td>NA</td><td>NA</td><td>NA</td><td>NA</td><td>NA</td><td>NA</td><td>7</td><td>19.8</td><td>NA</td><td>NA</td><td>NA</td><td>-0.1678</td><td>NA</td><td>NA</td><td>NA</td></tr>
<tr><td>1514</td><td>Q96I99</td><td>SUCLG2</td><td>Succinate--CoA li</td><td>sp|Q96I99|SUCB2_HUMAN</td><td>NA</td><td>NA</td><td>NA</td><td>NA</td><td>NA</td><td>NA</td><td>NA</td><td>NA</td><td>10</td><td>25.9</td><td>-0.3506</td><td>NA</td><td>NA</td><td>NA</td><td>NA</td><td>NA</td><td>NA</td></tr>
<tr><td>1515</td><td>Q96K76</td><td>USP47</td><td>Ubiquitin carbox</td><td>sp|Q96K76|UBP47_HUMAN</td><td>NA</td><td>NA</td><td>NA</td><td>NA</td><td>NA</td><td>NA</td><td>NA</td><td>NA</td><td>3</td><td>2.8</td><td>0.06969</td><td>NA</td><td>NA</td><td>NA</td><td>NA</td><td>NA</td><td>NA</td></tr>
<tr><td>1516</td><td>Q96L92</td><td>SNX27</td><td>Sorting nexin-27</td><td>sp|Q96L92|SNX27_HUMAN</td><td>NA</td><td>NA</td><td>NA</td><td>NA</td><td>NA</td><td>NA</td><td>NA</td><td>NA</td><td>4</td><td>9.2</td><td>0.24786</td><td>NA</td><td>NA</td><td>NA</td><td>NA</td><td>NA</td><td>NA</td></tr>
<tr><td>1517</td><td>Q9BWM7</td><td>SFXN3</td><td>Sideroflexin-3</td><td>sp|Q9BWM7|SFXN3_HUMA</td><td>NA</td><td>NA</td><td>NA</td><td>NA</td><td>NA</td><td>NA</td><td>NA</td><td>NA</td><td>1</td><td>4.4</td><td>NA</td><td>NA</td><td>NA</td><td>NA</td><td>NA</td><td>NA</td><td>NA</td></tr>
<tr><td>1518</td><td>Q9H098</td><td>FAM107B</td><td>Protein FAM107</td><td>sp|Q9H098|F107B_HUMAN</td><td>NA</td><td>NA</td><td>NA</td><td>NA</td><td>NA</td><td>NA</td><td>NA</td><td>NA</td><td>5</td><td>37.4</td><td>0.06189</td><td>0.81031</td><td>0.9991</td><td>-1.191</td><td>0.8103</td><td>0.9991</td><td>NA</td></tr>
<tr><td>1519</td><td>Q9HB90</td><td>RRAGC</td><td>Ras-related GTP</td><td>sp|Q9HB90|RRAGC_HUMA</td><td>NA</td><td>NA</td><td>NA</td><td>NA</td><td>NA</td><td>NA</td><td>NA</td><td>NA</td><td>5</td><td>14.3</td><td>-0.0068</td><td>NA</td><td>NA</td><td>NA</td><td>NA</td><td>NA</td><td>NA</td></tr>
<tr><td>1520</td><td>Q9HCS7</td><td>XAB2</td><td>Pre-mRNA-splici</td><td>sp|Q9HCS7|SYF1_HUMAN</td><td>NA</td><td>NA</td><td>NA</td><td>NA</td><td>NA</td><td>NA</td><td>NA</td><td>NA</td><td>4</td><td>7.4</td><td>0.17127</td><td>NA</td><td>NA</td><td>NA</td><td>NA</td><td>NA</td><td>NA</td></tr>
<tr><td>1521</td><td>Q9NP77</td><td>SSU72</td><td>RNA polymerase</td><td>sp|Q9NP77|SSU72_HUMA</td><td>NA</td><td>NA</td><td>NA</td><td>NA</td><td>NA</td><td>NA</td><td>NA</td><td>NA</td><td>3</td><td>16</td><td>NA</td><td>NA</td><td>NA</td><td>0.54438</td><td>NA</td><td>NA</td><td>NA</td></tr>
<tr><td>1522</td><td>Q9NVD7</td><td>PARVA</td><td>Alpha-parvin</td><td>sp|Q9NVD7|PARVA_HUMA</td><td>NA</td><td>NA</td><td>NA</td><td>NA</td><td>NA</td><td>NA</td><td>NA</td><td>NA</td><td>3</td><td>11.8</td><td>-0.2541</td><td>NA</td><td>NA</td><td>NA</td><td>NA</td><td>NA</td><td>NA</td></tr>
<tr><td>1523</td><td>Q9NWB6</td><td>ARGLU1</td><td>Arginine and glu</td><td>sp|Q9NWB6|ARGL1_HUMA</td><td>NA</td><td>NA</td><td>NA</td><td>NA</td><td>NA</td><td>NA</td><td>NA</td><td>NA</td><td>3</td><td>8.4</td><td>NA</td><td>NA</td><td>NA</td><td>1.34851</td><td>NA</td><td>NA</td><td>NA</td></tr>
<tr><td>1524</td><td>Q9NZM1</td><td>MYOF</td><td>Myoferlin</td><td>sp|Q9NZM1|MYOF_HUMA</td><td>NA</td><td>NA</td><td>NA</td><td>NA</td><td>NA</td><td>NA</td><td>NA</td><td>NA</td><td>10</td><td>6.3</td><td>0.13595</td><td>NA</td><td>NA</td><td>NA</td><td>NA</td><td>NA</td><td>NA</td></tr>
<tr><td>1525</td><td>Q9P2B2</td><td>PTGFRN</td><td>Prostaglandin F2</td><td>sp|Q9P2B2|FPRP_HUMAN</td><td>NA</td><td>NA</td><td>NA</td><td>NA</td><td>NA</td><td>NA</td><td>NA</td><td>NA</td><td>3</td><td>3.9</td><td>-0.0521</td><td>NA</td><td>NA</td><td>NA</td><td>NA</td><td>NA</td><td>NA</td></tr>
<tr><td>1526</td><td>Q9UBR2</td><td>CTSZ</td><td>Cathepsin Z</td><td>sp|Q9UBR2|CATZ_HUMAN</td><td>NA</td><td>NA</td><td>NA</td><td>NA</td><td>NA</td><td>NA</td><td>NA</td><td>NA</td><td>2</td><td>5.6</td><td>-1.3035</td><td>NA</td><td>NA</td><td>NA</td><td>NA</td><td>NA</td><td>NA</td></tr>
<tr><td>1527</td><td>Q9UBX3</td><td>SLC25A10</td><td>Mitochondrial di</td><td>sp|Q9UBX3|DIC_HUMAN M</td><td>NA</td><td>NA</td><td>NA</td><td>NA</td><td>NA</td><td>NA</td><td>NA</td><td>NA</td><td>3</td><td>11.8</td><td>0.20947</td><td>NA</td><td>NA</td><td>NA</td><td>NA</td><td>NA</td><td>NA</td></tr>
<tr><td>1528</td><td>Q9UIJ7</td><td>AK3</td><td>GTP:AMP phosp</td><td>sp|Q9UIJ7|KAD3_HUMAN</td><td>NA</td><td>NA</td><td>NA</td><td>NA</td><td>NA</td><td>NA</td><td>NA</td><td>NA</td><td>10</td><td>52.9</td><td>-0.1773</td><td>0.56205</td><td>0.9991</td><td>-0.2374</td><td>0.5621</td><td>0.9991</td><td>NA</td></tr>
<tr><td>1529</td><td>Q9UN36</td><td>NDRG2</td><td>Protein NDRG2</td><td>sp|Q9UN36|NDRG2_HUMA</td><td>NA</td><td>NA</td><td>NA</td><td>NA</td><td>NA</td><td>NA</td><td>NA</td><td>NA</td><td>3</td><td>12.1</td><td>-0.1951</td><td>NA</td><td>NA</td><td>NA</td><td>NA</td><td>NA</td><td>NA</td></tr>
<tr><td>1530</td><td>Q9UNH7</td><td>SNX6</td><td>Sorting nexin-6</td><td>sp|Q9UNH7|SNX6_HUMAN</td><td>NA</td><td>NA</td><td>NA</td><td>NA</td><td>NA</td><td>NA</td><td>NA</td><td>NA</td><td>6</td><td>17.2</td><td>-0.1678</td><td>0.49211</td><td>0.9991</td><td>-1.8418</td><td>0.4921</td><td>0.9991</td><td>NA</td></tr>
<tr><td>1531</td><td>Q9UQB8</td><td>BAIAP2</td><td>Brain-specific an</td><td>sp|Q9UQB8|BAIP2_HUMAN</td><td>NA</td><td>NA</td><td>NA</td><td>NA</td><td>NA</td><td>NA</td><td>NA</td><td>NA</td><td>10</td><td>22.8</td><td>-0.1545</td><td>NA</td><td>NA</td><td>NA</td><td>NA</td><td>NA</td><td>NA</td></tr>
<tr><td>1532</td><td>Q9Y281</td><td>CFL2</td><td>Cofilin-2</td><td>sp|Q9Y281|COF2_HUMAN</td><td>NA</td><td>NA</td><td>NA</td><td>NA</td><td>NA</td><td>NA</td><td>NA</td><td>NA</td><td>8</td><td>50.6</td><td>0.18032</td><td>NA</td><td>NA</td><td>NA</td><td>NA</td><td>NA</td><td>NA</td></tr>
</table>

1533	Q9Y2Q9	MRPS28	28S ribosomal p	sp|Q9Y2Q9|RT28_HUMAN	NA	NA	NA	NA	NA	NA	NA	NA	2	10.2	NA	NA	NA	1.27664	NA	NA	NA
1534	Q9Y371	SH3GLB1	Endophilin-B1	sp|Q9Y371|SHLB1_HUMAN	NA	NA	NA	NA	NA	NA	NA	NA	4	11.2	-0.0003	0.99932	0.9999	-1.1099	0.9993	0.9999	NA
1535	Q9Y5X1	SNX9	Sorting nexin-9	sp|Q9Y5X1|SNX9_HUMAN	NA	NA	NA	NA	NA	NA	NA	NA	2	4.2	-0.126	NA	NA	NA	NA	NA	NA
1536	Q9NR31	SAR1A	GTP-binding pro	sp|Q9NR31|SAR1A_HUMA	4	26.8	0.1287896	0.50254	0.9984	-0.8872	0.50254	0.99836	6	42.9	NA	NA	NA	NA	NA	NA	NA
1537	P0DPI2	GATD3A	Glutamine amid	sp|P0DPI2|GAL3A_HUMAN	3	17.5	-0.01067	0.97378	0.999	0.5892	0.97378	0.99899	4	25	NA	NA	NA	NA	NA	NA	NA
1538	P68036	UBE2L3	Ubiquitin-conjug	sp|P68036|UB2L3_HUMAN	2	10.4	-0.072379	0.85246	0.9991	-0.7607	0.06409	0.20018	8	65.6	-0.0669	0.86359	0.9973	-0.221	0.5718	0.6636	NA
1539	A8MPP1	DDX11L8	Putative ATP-de	sp|A8MPP1|D11L8_HUMA	2	2.2	-0.103113	NA	NA	NA	NA	NA	1	1.3	NA	NA	NA	NA	NA	NA	NA
1540	Q99613	EIF3C	Eukaryotic trans	sp|Q99613|EIF3C_HUMAN	10	15.4	0.0305203	0.87519	0.9991	-0.2883	0.19671	0.37977	17	25.1	0.02772	0.88657	0.9973	-0.2989	0.1381	0.2057	NA
1541	E9PAV3	NACA	Nascent polypep	sp|E9PAV3|NACAM_HUMA	6	5.1	0.2180141	0.31103	0.9991	-0.3311	0.13152	0.29758	5	3.4	0.06699	0.7521	0.9973	-0.9268	0.0004	0.0015	4
1542	O00571	DDX3X	ATP-dependent	sp|O00571|DDX3X_HUMAN	10	19.5	-0.057922	0.78227	0.9991	-0.1701	0.42133	0.61339	18	30.7	-0.0516	0.80532	0.9973	-1.3786	######	######	4
1543	P47813	EIF1AX	Eukaryotic trans	sp|P47813|IF1AX_HUMAN	3	19.4	0.7805285	0.16742	0.9991	0.1354	0.82061	0.91051	4	31.2	0.22196	0.64999	0.9973	0.15072	0.7823	0.8217	NA
1544	O14818	PSMA7	Proteasome sub	sp|O14818|PSA7_HUMAN	7	37.5	0.2341068	0.26339	0.9991	-0.233	0.26563	0.46617	9	41.5	-0.075	0.71544	0.9973	0.70054	0.0031	0.0084	2
1545	P19105	MYL12A	Myosin regulato	sp|P19105|ML12A_HUMAN	3	22.8	-0.311614	0.55686	0.9991	-0.3831	0.5103	0.68136	9	59.1	0.25252	0.59413	0.9973	0.59523	0.2193	0.3042	NA
1546	O43865	AHCYL1	S-adenosylhomo	sp|O43865|SAHH2_HUMA	2	4.3	-0.293612	NA	NA	NA	NA	NA	5	13.6	NA	NA	NA	NA	NA	NA	NA
1547	O60684	KPNA6	Importin subunit	sp|O60684|IMA7_HUMAN	2	5.4	NA	NA	NA	NA	NA	NA	9	28.4	0.09468	0.62715	0.9991	0.08749	0.6272	0.9991	NA
1548	O75348	ATP6V1G1	V-type proton A	sp|O75348|VATG1_HUMA	2	22	NA	NA	NA	0.0998	NA	NA	3	28.8	NA	NA	NA	NA	NA	NA	NA
1549	O95239	KIF4A	Chromosome-ass	sp|O95239|KIF4A_HUMAN	4	3.4	-0.374034	0.38694	0.9984	-0.0854	0.38694	0.99836	7	5.7	0.10051	NA	NA	NA	NA	NA	NA
1550	O95819	MAP4K4	Mitogen-activat	sp|O95819|M4K4_HUMAN	3	3.6	NA	NA	NA	NA	NA	NA	6	6.5	-0.8129	NA	NA	NA	NA	NA	NA
1551	O96019	ACTL6A	Actin-like protei	sp|O96019|ACL6A_HUMAN	4	12.6	-0.222544	0.33233	0.9984	0.5604	0.33233	0.99836	8	29.4	NA	NA	NA	NA	NA	NA	NA
1552	P00367	GLUD1	Glutamate dehy	sp|P00367|DHE3_HUMAN	11	23.3	-0.026912	0.88027	0.9991	0.4967	0.01203	0.07061	14	31	-0.0394	0.82564	0.9973	-0.6715	0.0015	0.0044	5
1553	P01111	NRAS	GTPase NRas	sp|P01111|RASN_HUMAN	2	12.2	0.2233319	0.54478	0.9984	0.4146	0.54478	0.99836	2	12.2	NA	NA	NA	NA	NA	NA	NA
1554	P10316	HLA-A	HLA class I histo	sp|P10316|1A69_HUMAN	4	16.2	-0.17651	0.55716	0.9984	0.4492	0.55716	0.99836	NA	NA	NA	NA	NA	NA	NA	NA	NA
1555	Q99878	HIST1H2AJ	Histone H2A typ	sp|Q99878|H2A1J_HUMAN	7	38.3	-0.050322	0.92912	0.999	0.0309	0.92912	0.99899	NA	NA	NA	NA	NA	NA	NA	NA	NA
1556	P05388	RPLP0	60S acidic ribos	sp|P05388|RLA0_HUMAN 6	10	43.2	-0.095054	0.77673	0.9991	-0.5384	0.12152	0.2853	13	53.3	0.05038	0.88043	0.9973	-0.4318	0.2083	0.2937	NA
1557	P07355	ANXA2	Annexin A2	sp|P07355|ANXA2_HUMAN	13	46.3	-0.145036	0.4544	0.9991	-0.2334	0.235	0.43276	29	75.8	0.19826	0.31008	0.9973	-0.9284	0.0001	0.0006	4
1558	P09429	HMGB1	High mobility gr	sp|P09429|HMGB1_HUMA	8	32.6	0.1241136	0.68659	0.9991	-0.3275	0.29419	0.49544	12	50.2	-0.121	0.69392	0.9973	1.77134	######	0.0001	2
1559	P09651	HNRNPA1	Heterogeneous	sp|P09651|ROA1_HUMAN	17	40.6	-0.466346	0.23532	0.9991	-0.3883	0.3199	0.51783	19	48.1	0.06532	0.8651	0.9973	1.7231	0.0003	0.0012	2
1560	Q71UI9	H2AFV	Histone H2A.V	sp|Q71UI9|H2AV_HUMAN	5	31.2	0.5477235	0.22309	0.9991	0.3309	0.45506	0.64015	6	53.9	0.62535	0.16708	0.9973	2.42658	######	0.0002	1
1561	P0CG35	TMSB15B	Thymosin beta-1	sp|P0CG35|TB15B_HUMAN	2	40	0.6709544	0.01493	0.9984	-0.3646	0.01493	0.99836	NA	NA	NA	NA	NA	NA	NA	NA	NA
1562	P0DMV9	HSPA1B	Heat shock 70 k	sp|P0DMV9|HS71B_HUMA	17	35.1	-0.132933	0.47877	0.9984	0.2599	0.47877	0.99836	33	68.8	NA	NA	NA	NA	NA	NA	NA
1563	P0DN76	U2AF1L5	Splicing factor U	sp|P0DN76|U2AF5_HUMA	2	15	NA	NA	NA	NA	NA	NA	4	25.8	NA	NA	NA	-0.5205	NA	NA	NA
1564	P35520	CBS	Cystathionine be	sp|P35520|CBS_HUMAN Cy	9	20.7	0.1552582	0.45484	0.9984	-0.0986	0.45484	0.99836	3	6.2	NA	NA	NA	NA	NA	NA	NA
1565	P0DP25	CALM3	Calmodulin-3	sp|P0DP25|CALM3_HUMA	10	70.5	-0.02015	0.93831	0.9991	-0.1763	0.50133	0.67575	12	85.2	0.08331	0.74937	0.9973	0.4446	0.1017	0.1603	NA
1566	P11169	SLC2A3	Solute carrier fa	sp|P11169|GTR3_HUMAN S	6	13.1	-0.19015	0.31291	0.9984	0.8211	0.31291	0.99836	NA	NA	NA	NA	NA	NA	NA	NA	NA
1567	P12532	CKMT1A	Creatine kinase	sp|P12532|KCRU_HUMAN (	2	4.6	0.2043584	NA	NA	NA	NA	NA	12	45.6	NA	NA	NA	NA	NA	NA	NA
1568	P63000	RAC1	Ras-related C3 b	sp|P63000|RAC1_HUMAN	2	10.9	NA	NA	NA	NA	NA	NA	7	43.2	0.17321	0.43634	0.9991	0.87262	0.4363	0.9991	NA
1569	P16104	H2AFX	Histone H2AX	sp|P16104|H2AX_HUMAN	6	37.8	NA	NA	NA	NA	NA	NA	8	63.6	0.25704	0.234	0.9991	1.34736	0.234	0.9991	NA
1570	P20340	RAB6A	Ras-related prot	sp|P20340|RAB6A_HUMAN	5	28.8	-0.319335	0.2995	0.9991	-0.2546	0.40521	0.60515	5	28.8	-0.3117	0.31088	0.9973	-0.6011	0.0604	0.103	NA
1571	P22392	NME2	Nucleoside diph	sp|P22392|NDKB_HUMAN	10	66.4	-0.212451	0.55616	0.9991	-1.1536	0.00477	0.04078	11	74.3	-0.4416	0.22927	0.9973	1.22224	0.0032	0.0084	2
1572	Q16778	HIST2H2BE	Histone H2B typ	sp|Q16778|H2B2E_HUMAN	11	60.3	-0.226468	0.499	0.9984	-0.0579	0.499	0.99836	11	60.3	NA	NA	NA	NA	NA	NA	NA
1573	P38159	RBMX	RNA-binding mo	sp|P38159|RBMX_HUMAN	12	31.7	0.3731403	0.1306	0.9984	0.7748	0.1306	0.99836	9	22.8	NA	NA	NA	NA	NA	NA	NA
1574	P40429	RPL13A	60S ribosomal p	sp|P40429|RL13A_HUMAN	4	20.7	0.1120594	0.79	0.9991	0.4367	0.30676	0.50462	6	30	0.29026	0.49302	0.9973	-0.1563	0.7106	0.7775	NA
1575	P41091	EIF2S3	Eukaryotic trans	sp|P41091|IF2G_HUMAN E	8	23.5	-0.411396	0.15124	0.9991	-0.9594	0.00645	0.04968	16	48.1	-0.0096	0.97243	0.9973	1.26986	0.0003	0.0011	2
1576	P46783	RPS10	40S ribosomal p	sp|P46783|RS10_HUMAN 4	5	27.9	0.2101839	0.52486	0.9991	0.0553	0.86628	0.92126	8	43	0.47378	0.16197	0.9973	-0.9922	0.0072	0.0167	4
1577	P49585	PCYT1A	Choline-phospha	sp|P49585|PCY1A_HUMAN	2	8.4	0.2347976	NA	NA	NA	NA	NA	3	12.5	NA	NA	NA	NA	NA	NA	NA
1578	P50502	ST13	Hsc70-interactin	sp|P50502|F10A1_HUMAN	7	25.7	0.1339408	0.70569	0.9991	-1.2877	0.0019	0.0275	8	25.5	0.04495	0.89895	0.9973	2.02474	######	0.0002	2
1579	P55786	NPEPPS	Puromycin-sensit	sp|P55786|PSA_HUMAN P	13	15.3	-0.279507	0.18911	0.9991	-1.2238	1.69E-05	0.00239	9	11.5	-0.1065	0.60863	0.9973	-0.6372	0.0064	0.0152	3
1580	Q99879	HIST1H2BM	Histone H2B typ	sp|Q99879|H2B1M_HUMA	11	60.3	-0.091418	0.83714	0.9991	-0.04	0.92832	0.9536	11	60.3	0.40245	0.37121	0.9973	-0.3206	0.4743	0.5671	NA

1581	P61088	UBE2N	Ubiquitin-conjug	sp|P61088|UBE2N_HUMAN	4	36.8	-0.050143	0.85903	0.9991	-0.7906	0.01151	0.07028	10	80.9	0.27762	0.33235	0.9973	2.2958	######	######	2
1582	P61158	ACTR3	Actin-related pr	sp|P61158|ARP3_HUMAN	3	6.7	0.0635507	0.8875	0.9984	0.0884	0.8875	0.99836	11	36.1	NA	NA	NA	NA	NA	NA	NA
1583	P84077	ARF1	ADP-ribosylation	sp|P84077|ARF1_HUMAN	4	33.7	0.4305792	0.13536	0.9991	-0.6063	0.04152	0.15175	7	54.7	0.06183	0.82433	0.9973	-1.41	######	0.0004	4
1584	P61224	RAP1B	Ras-related prot	sp|P61224|RAP1B_HUMAN	3	19	-0.046205	0.85969	0.9991	-0.0657	0.85883	0.92074	8	42.9	-0.0873	0.73887	0.9973	-1.1712	0.0004	0.0016	4
1585	P61254	RPL26	60S ribosomal p	sp|P61254|RL26_HUMAN 6	6	37.2	0.1749386	0.53461	0.9991	0.5506	0.06279	0.19963	7	37.9	0.01738	0.9505	0.9973	-0.8586	0.0066	0.0154	5
1586	P61619	SEC61A1	Protein transpor	sp|P61619|S61A1_HUMAN	3	6.1	-0.111437	0.78045	0.9984	1.5568	0.78045	0.99836	3	6.7	NA	NA	NA	NA	NA	NA	NA
1587	P61956	SUMO2	Small ubiquitin-r	sp|P61956|SUMO2_HUMA	2	23.2	-0.525962	0.1171	0.9991	-0.0464	0.88564	0.9344	2	23.2	0.04382	0.89205	0.9973	-0.7126	0.0392	0.0705	4
1588	P63167	DYNLL1	Dynein light cha	sp|P63167|DYL1_HUMAN	2	37.1	-0.11552	0.86816	0.9984	0.1762	0.86816	0.99836	3	44.9	NA	NA	NA	NA	NA	NA	NA
1589	P63241	EIF5A	Eukaryotic trans	sp|P63241|IF5A1_HUMAN	7	69.5	0.2089738	0.3078	0.9991	-0.464	0.03245	0.12854	13	88.3	0.15636	0.44218	0.9973	0.15244	0.4535	0.545	3
1590	P68133	ACTA1	Actin, alpha ske	sp|P68133|ACTS_HUMAN	17	38.7	-0.463123	0.1921	0.9991	-0.1178	0.76036	0.87233	14	32.4	0.175	0.61349	0.9973	0.04704	0.8916	0.9082	NA
1591	P68104	EEF1A1	Elongation facto	sp|P68104|EF1A1_HUMAN	30	61.7	-0.152417	0.43746	0.9991	-0.9618	0.00012	0.00755	28	72.5	0.00427	0.98246	0.9973	-0.2412	0.2255	0.3087	3
1592	P68400	CSNK2A1	Casein kinase II	sp|P68400|CSK21_HUMAN	2	7.2	-0.70289	NA	NA	NA	NA	NA	7	27.4	0.11216	0.71479	0.9991	-0.1714	0.7148	0.9991	NA
1593	P69849	NOMO3	Nodal modulator	sp|P69849|NOMO3_HUMA	12	13.5	0.0806778	0.77697	0.9991	0.4178	0.15486	0.32873	9	9.3	-0.0468	0.86926	0.9973	-1.0974	0.0012	0.0037	5
1594	Q01105	SET	Protein SET	sp|Q01105|SET_HUMAN P	5	24.1	0.325981	0.18646	0.9991	-0.2684	0.27259	0.4737	7	33.8	-0.0521	0.82839	0.9973	1.16687	0.0001	0.0006	2
1595	Q14671	PUM1	Pumilio homolog	sp|Q14671|PUM1_HUMAN	4	3.9	-0.117559	NA	NA	NA	NA	NA	4	5.1	NA	NA	NA	NA	NA	NA	NA
1596	Q9H8S9	MOB1A	MOB kinase acti	sp|Q9H8S9|MOB1A_HUMA	2	11.6	0.101086	NA	NA	NA	NA	NA	3	16.7	0.20128	NA	NA	NA	NA	NA	NA
1597	Q8N0W4	NLGN4X	Neuroligin-4, X-l	sp|Q8N0W4|NLGNX_HUMA	3	5.6	-0.244938	0.45613	0.9984	0.5045	0.45613	0.99836	NA	NA	NA	NA	NA	NA	NA	NA	NA
1598	Q93008	USP9X	Probable ubiquit	sp|Q93008|USP9X_HUMAN	11	5.8	0.3550563	0.1272	0.9984	-0.112	0.1272	0.99836	5	2.8	NA	NA	NA	NA	NA	NA	NA
1599	Q99961	SH3GL1	Endophilin-A2	sp|Q99961|SH3G1_HUMAN	4	13.9	-0.203203	NA	NA	NA	NA	NA	12	42.9	NA	NA	NA	NA	NA	NA	NA
1600	Q9BWF3	RBM4	RNA-binding pro	sp|Q9BWF3|RBM4_HUMAN	7	25.8	-0.084428	0.75078	0.9984	0.6208	0.75078	0.99836	11	34.6	NA	NA	NA	2.5035	NA	NA	NA
1601	Q9H0U4	RAB1B	Ras-related prot	sp|Q9H0U4|RAB1B_HUMA	7	48.3	-0.017143	0.96045	0.999	0.4514	0.96045	0.99899	10	58.7	NA	NA	NA	NA	NA	NA	NA
1602	Q9NUU7	DDX19A	ATP-dependent	sp|Q9NUU7|DD19A_HUMA	7	16.7	0.403169	NA	NA	NA	NA	NA	8	20.3	-0.2489	0.3926	0.9991	-0.0358	0.3926	0.9991	NA
1603	Q9P289	STK26	Serine/threonine	sp|Q9P289|STK26_HUMAN	5	14.4	0.0275134	0.88793	0.9984	-0.294	0.88793	0.99836	4	12.7	NA	NA	NA	NA	NA	NA	NA
1604	Q9UQN3	CHMP2B	Charged multive	sp|Q9UQN3|CHM2B_HUM	2	7.5	0.1189147	0.80464	0.9984	0.133	0.80464	0.99836	1	0	NA	NA	NA	NA	NA	NA	NA
1605	Q9Y605	MRFAP1	MORF4 family-a	sp|Q9Y605|MOFA1_HUMA	2	28.3	0.1223033	0.76664	0.9984	0.3327	0.76664	0.99836	2	26.8	NA	NA	NA	NA	NA	NA	NA
1606	P11940	PABPC1	Polyadenylate-b	sp|P11940|PABP1_HUMAN	22	44	NA	NA	NA	NA	NA	NA	27	51.6	0.07752	0.70086	0.9991	-0.3842	0.7009	0.9991	NA
1607	P35321	SPRR1A	Cornifin-A	sp|P35321|SPR1A_HUMAN	NA	NA	NA	NA	NA	NA	NA	NA	2	27	1.02267	NA	NA	NA	NA	NA	NA
1608	Q9NRH3	TUBG2	Tubulin gamma-	sp|Q9NRH3|TBG2_HUMAN	NA	NA	NA	NA	NA	NA	NA	NA	4	14	0.29631	0.57544	0.9991	0.55774	0.5754	0.9991	NA
1609	P62701	RPS4X	40S ribosomal p	sp|P62701|RS4X_HUMAN 4	14	52.1	NA	NA	NA	NA	NA	NA	16	55.1	-0.0649	0.80303	0.9991	-0.7743	0.803	0.9991	NA
1610	P62854	RPS26	40S ribosomal p	sp|P62854|RS26_HUMAN 4	1	13	NA	NA	NA	NA	NA	NA	3	27	-0.9001	0.025	0.9991	-0.0055	0.025	0.9991	NA
1611	P62879	GNB2	Guanine nucleot	sp|P62879|GBB2_HUMAN	5	19.4	NA	NA	NA	NA	NA	NA	9	36.2	-0.0123	0.96571	0.9991	0.63996	0.9657	0.9991	NA
1612	P63165	SUMO1	Small ubiquitin-r	sp|P63165|SUMO1_HUMA	4	35.6	NA	NA	NA	NA	NA	NA	4	36.6	0.65667	0.39626	0.9991	3.03838	0.3963	0.9991	NA
1613	Q01130	SRSF2	Serine/arginine-	sp|Q01130|SRSF2_HUMAN	5	22.2	NA	NA	NA	NA	NA	NA	4	21.7	-0.1121	0.77105	0.9991	-0.6356	0.771	0.9991	NA
1614	Q15052	ARHGEF6	Rho guanine nuc	sp|Q15052|ARHG6_HUMA	NA	NA	NA	NA	NA	NA	NA	NA	2	3	NA	NA	NA	-0.8735	NA	NA	NA
1615	Q16777	HIST2H2AC	Histone H2A typ	sp|Q16777|H2A2C_HUMAN	NA	NA	NA	NA	NA	NA	NA	NA	6	55	0.08528	0.81574	0.9991	2.37984	0.8157	0.9991	NA
1616	Q9HB90	RRAGC	Ras-related GTP	sp|Q9HB90|RRAGC_HUMA	NA	NA	NA	NA	NA	NA	NA	NA	5	14.3	-0.0068	NA	NA	NA	NA	NA	NA
