# Supplementary material for: Hyperosmotic Stress Induces a Specific Pattern for Stress Granule Formation in Human-Induced Pluripotent Stem Cells
Source: Stem Cells Int. 2021 Oct 15;2021:8274936. doi: 10.1155/2021/8274936 (PMC8538399; doi:10.1155/2021/8274936)
Supplement: Supplementary 1 — Supplementary Tables—Table S1: LC-MS/MS parameters. Table 2: list of identified proteins—mass spectrometry-based protein quantitation. Table S3: data summary of all quantified proteins in hiPSCs/IMR90-1 and SH-SY5Y. Supplementary Table S4: data summary of overlapped proteins between hiPSCs/IMR90-1 and SH-SY5Y. Supplementary Table S5: top networks in hiPSCs/IMR90-01 and SH-SY5Y. [file 8274936.f1.zip › Supplementary Tables (2).docx]

**Supplementary Tables:**

**Supplementary Table S1: LC-MS/MS parameters**

| **Nano-liquid chromatography (nLC)** | |
| --- | --- |
| Instrument | EASY nLC-1200 (Thermo Scientific) |
| Analytical column | 18 cm Fused Silica Capillary Tubing of 75 µm inner diameter, packed with ReproSil-Pur 120 C18-AQ, 3 µm (Dr. Maisch GmbH, r13.aq) |
|  |  |
| Flow rate | 250 nL/min |
| Column oven temperature | 50°C |
| Chromatography conditions | Water with 0.5% acetic acid for mobile phase A;  Water with acetonitrile, 20:80 volume ratio, with 0.5% acetic acid for mobile phase B;  Flow-rate of 250 nL/min; |
| Gradient  Injection volume  Loading pressure | Gradient of buffer B:   - 5 min 5%, - 90 min 5% to 30%, - 5 min 30% to 60%, - 10 min 60 to 95%, - 10 min 95% to5%,   6.0 μL  280 bars |
|  | |
| **Mass spectrometry (MS)** | |
| Instrument | Q Exactive HF (Thermo Scientific) |
| Operation mode | Data-dependent, top 10 method |
| Electrospray | Nanospray Flex Ion Source |
| **Full MS** |  |
| MS scan resolution | 60,000 |
| AGC target | 3e6 |
| Maximum ion injection time for the MS scan | 20 ms |
| Scan range | 300 – 1,650 m/z |
| Spectra data type | Profile |
| **dd-MS2** |  |
| Resolution | 15,000 |
| MS/MS AGC target | 1e5 |
| Maximum ion injecting time for the MS/MS  **Scans** | 120 ms |
| Spectra data type | Profile |
| Selection for MS/MS | 10 most abundant isotope patterns with charge ≥2 from the survey scan |
| Isolation window | 3.0 |
| Dissociation mode | HCD |
| Normalized collision energy | 25% |
| Dynamic exclusion | 25 s |
| Charge exclusion  Fixed lower mass-to-charge  Charge Exclusion  Peptide Match | Unassigned, 1,  cut-off of 100  On  On |

**Supplementary Table S3. Data summary of all quantified proteins in hiPSCs/IMR90-1 and SH-SY5Y.**

| Cell type | Comparison | p<0.05 | FDR<0.05 | bonf<0.05 |
| --- | --- | --- | --- | --- |
| hiPSCs/IMR90-1 | 200mM vs NT | 29 | 0 | 0 |
|  | 400mM vs NT | 279 | 72 | 0 |
| SH-SY5Y | 200mM vs NT | 17 | 0 | 0 |
|  | 400mM vs NT | 455 | 395 | 82 |

**Supplementary Table S4. Data summary of overlapped proteins between hiPSCs/IMR90-1 and SH-SY5Y.**

| Cell type | Comparison | p<0.05 | FDR<0.05 | bonf<0.05 |
| --- | --- | --- | --- | --- |
| hiPSCs/IMR90-1 | 200mM vs NT | 12 | 0 | 0 |
|  | 400mM vs NT | 159 | 88 | 7 |
| SH-SY5Y | 200mM vs NT | 15 | 0 | 0 |
|  | 400mM vs NT | 372 | 356 | 138 |

**Supplementary Table S5: Top Networks in hiPSCs/IMR90-01 and SH-SY5Y.**

| Cell type | Molecules in Network | Score | Focus Molecules | Top Diseases and Functions |
| --- | --- | --- | --- | --- |
| hiPSCs/IMR90-1 | AARS1,Actin,AHCY,Alpha tubulin,BETA TUBULIN,CAP1,CCT3,CCT5,CCT6A,CCT8,CFL1,CLIC4,Cofilin,Cytoplasmic Dynein,DNA-PK,DYNC1H1,EIF2S3,F Actin,G-Actin,Hsp90,Immunoglobulin,LDHB,PFN1,PPIA,TKT,TPI1,TPR,TUBA1B,TUBB,TUBB4B,tubulin,tubulin (family),WDR1,YWHAQ,YWHAZ | 50 | 23 | [Cell-To-Cell Signaling and Interaction, Cellular Assembly and Organization, Reproductive System Development and Function] |
| SH-SY5Y | ATXN2L,CAPRIN1,DDX3X,DHX9,FUS,HNRNPA1,HNRNPDL,HNRNPH3,HNRNPK,HNRNPM,HNRNPR,ILF2,ILF3,MATR3,NOP56,nucleoside-triphosphatase,PCBP1,PCBP2,PYM1,RPL10A,RPL21,RPL22,RPL26,RPL3,RPL30,RPL4,RPL7,RPL9,RPLP1,RTRAF,Spliceosome,SRSF1,SRSF6,TARDBP,U2AF2 | 61 | 33 | [Protein Synthesis, RNA Damage and Repair, RNA Post-Transcriptional Modification] |
