# Supplementary material for: Hyperosmotic Stress Induces a Specific Pattern for Stress Granule Formation in Human-Induced Pluripotent Stem Cells
Source: Stem Cells Int. 2021 Oct 15;2021:8274936. doi: 10.1155/2021/8274936 (PMC8538399; doi:10.1155/2021/8274936)
Supplement: Supplementary 2 — Supplementary Figures—Supplementary Figure 1: pluripotency marker assessment of hiPSCs/IMR90-1 after hyperosmolarity treatment. Supplementary Figure 2: stress granules in iPSCs have the same molecular components as those in other types of cells. Supplementary Figure 3: G3BP stress granule marker in hiPSCs/IMR90-1 after sorbitol treatment. Supplementary Figure 4: two different stem cell lines were treated with NaCl hyperosmolarity treatment (no treatment (control), 200 mM, and 400 mM). Supplementary Figure 5: two different cell lines were treated with NaCl hyperosmolarity treatment (no treatment (control), 200 mM, and 400 mM). Supplementary Figure 6: phosphorylation of eIF2 alpha in both hiPSCs/IMR90-1 and SH-SY5Y after NaCl treatment. Supplementary Figure 7: effects of NaCl on caspase-3 activation by Western blot analysis. Supplementary Figure 8: graphical representation of proteomic data. Supplementary Figure 9: additional mitochondrial quantified proteins. [file 8274936.f2.docx]

**Supplementary Figures:**

**Supplementary Fig. 1.**

**Supplementary Fig 1: Pluripotency marker assessment of hiPSCs/IMR90-1 after hyperosmolarity treatment.** Images of immunofluorescent staining of pluripotency markers (Nanong, Oct4 and Sox2) on hiPSC colonies (green)). Nucleus is stained in blue (Hoechst).

**Supplementary Fig 2.**


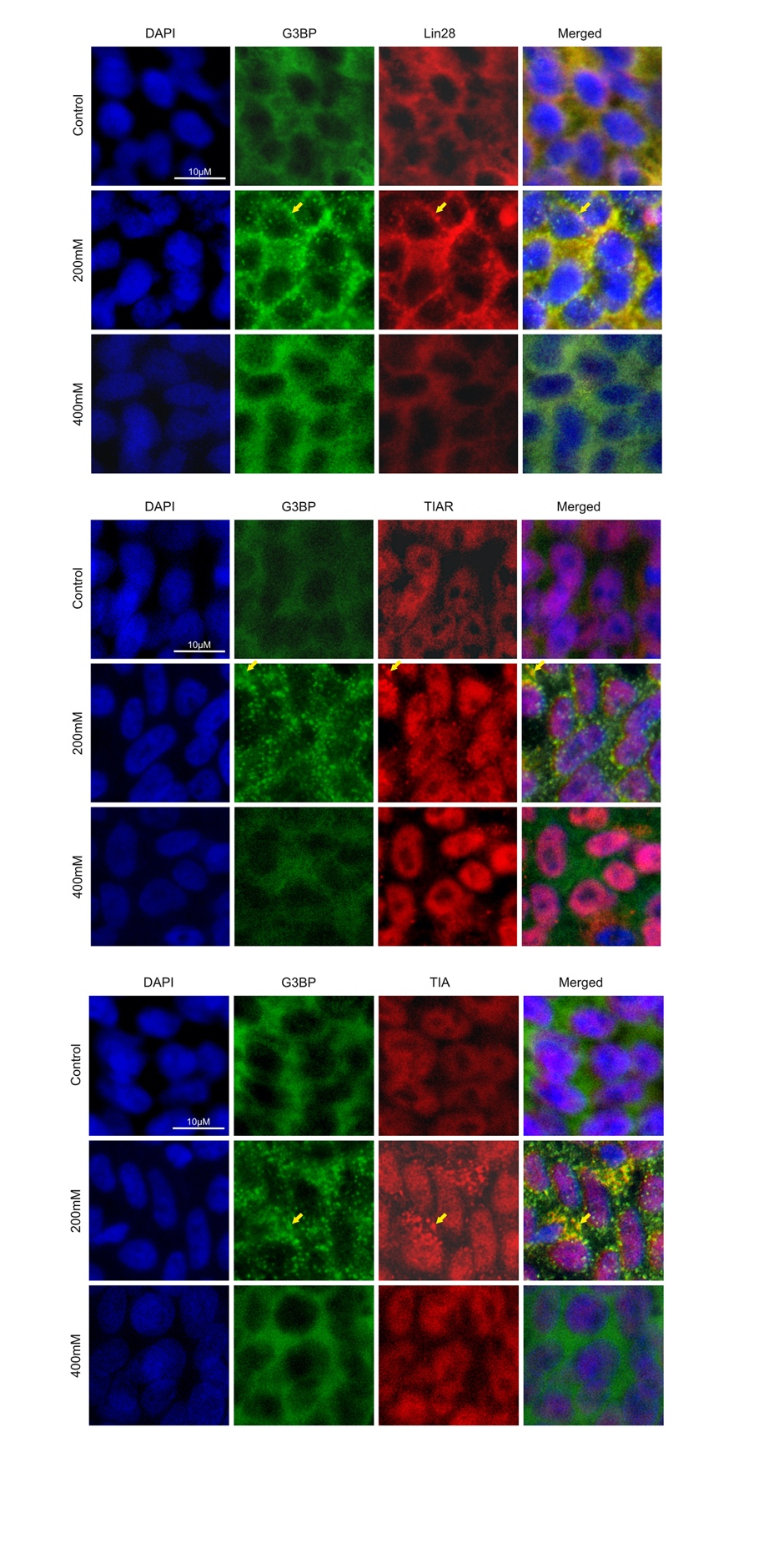


(A)

(B)

(C)

‘

**Supplementary Fig. 2. Stress granules in iPSCs have the same molecular components as those in other types of cells.** The stress granules in iPSCs containing G3BP and the known SG markers such as LIN28 (A), TIA-1 (B) and TIAR (C) are shown.

**Supplementary Fig. 3.**


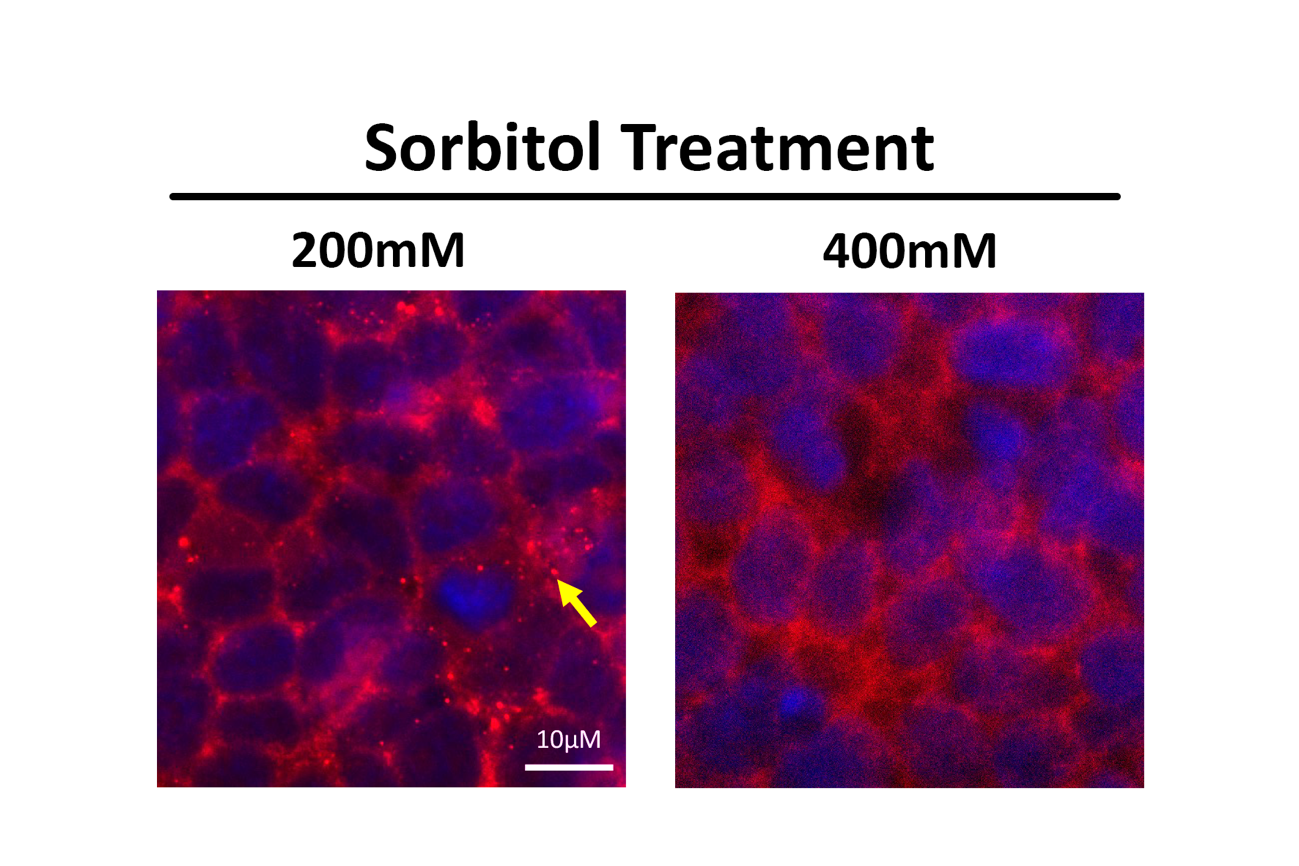


**Supplementary Fig. 3: G3BP Stress Granules marker in hiPSCs/IMR90-1 after sorbitol treatment.** Images of fluorescent hiPSCs/IMR90-1 under treatment with A) 200mM and B) 400mM of sorbitol stained with the robust SG marker (G3BP (red)). Nucleus is stained in blue (Hoechst). Yellow arrow indicates SGs. Scale bars indicates 10 μm.

**Supplementary Fig 4.**

**
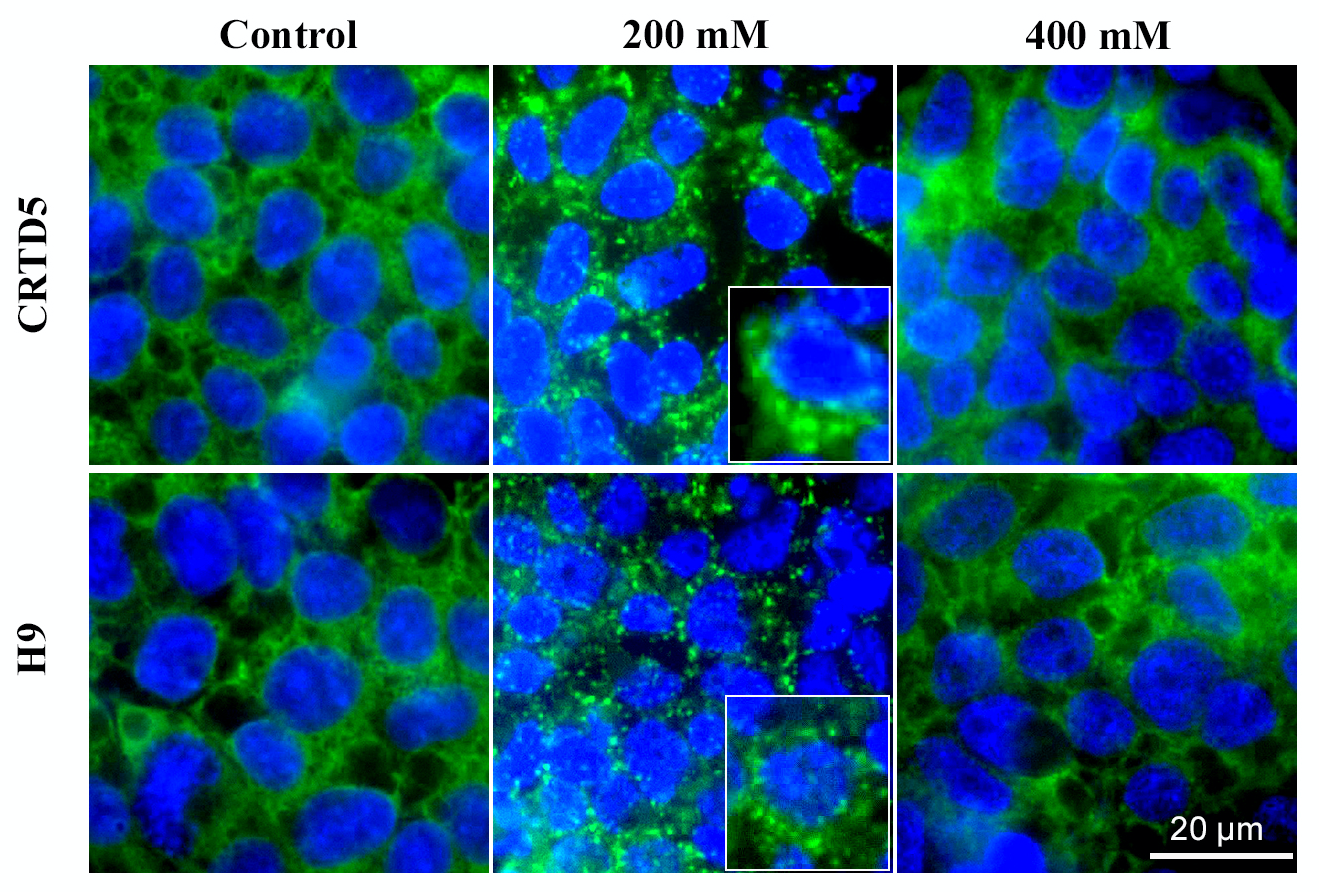
**

**Supplementary Fig 4: Two different stem cell lines were treated with NaCl hyperosmolarity treatment (No treat (control), 200mM and 400mM).** Images of immunofluorescent staining of SGs marker (G3BP) on cells (green). Nucleus is stained in blue (Hoechst). Yellow arrow indicates SGs. Scale bars indicates 20 μm.

**Supplementary Fig 5.**


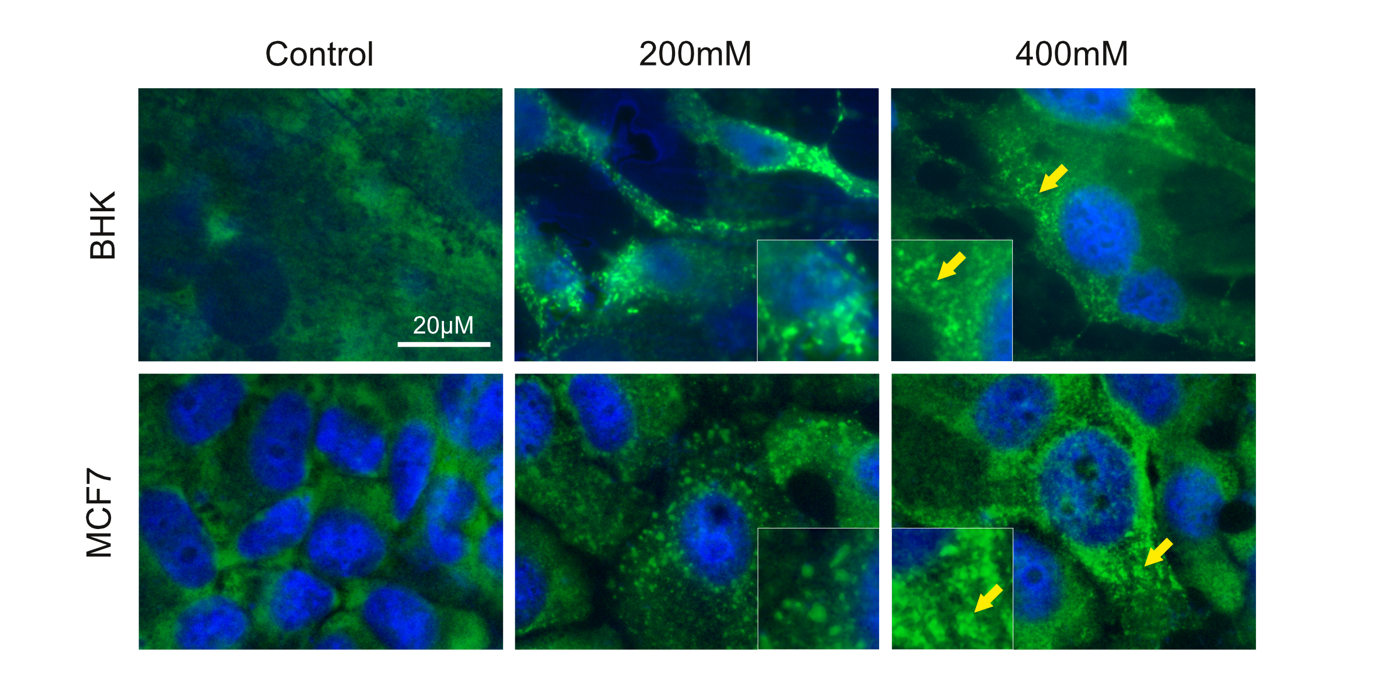


**Supplementary Fig 5: Two different cell lines were treated with NaCl hyperosmolarity treatment (No treat (control), 200mM and 400mM).** Images of immunofluorescent staining of SGs marker (G3BP) on cells (green). Nucleus is stained in blue (Hoechst). Yellow arrow indicates SGs. Scale bars indicates 20 μm.

**Supplementary Fig. 6.**

**hiPSCs/IMR90-1**


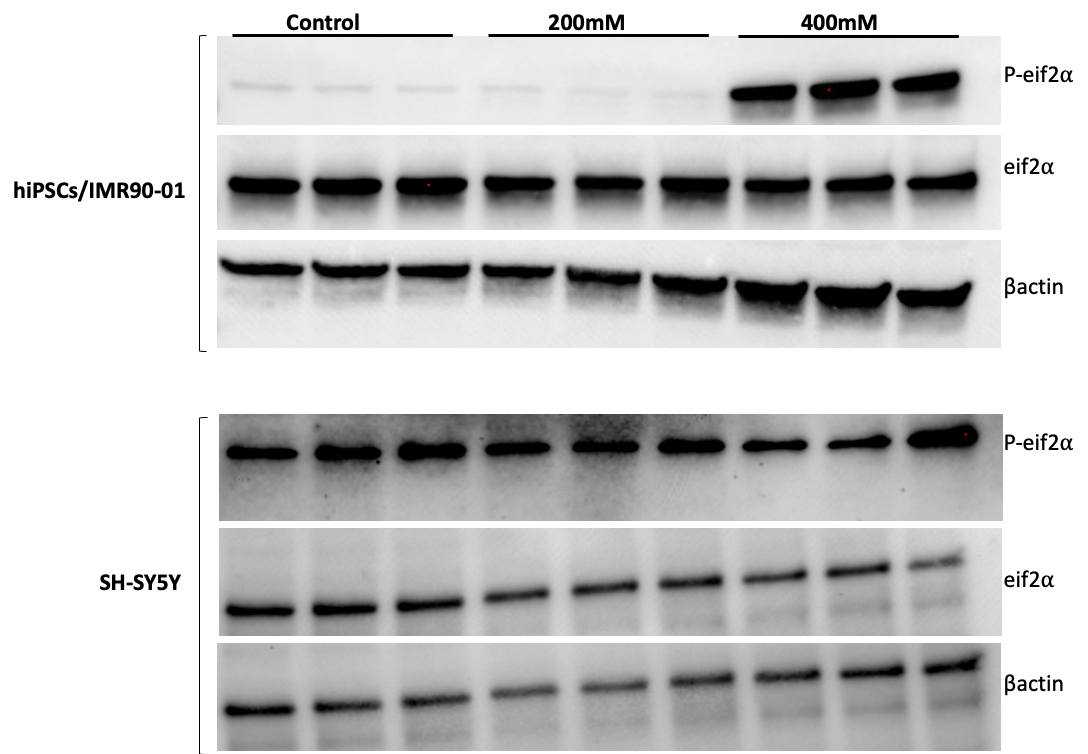

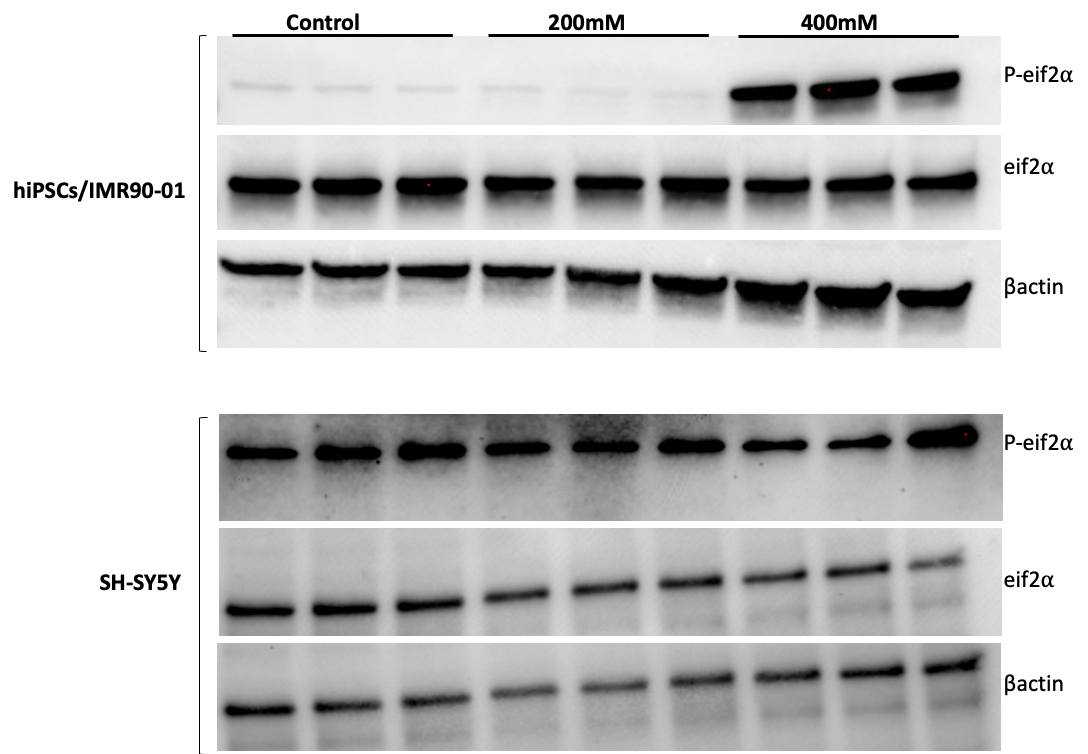


**Supplementary Fig 6. Phosphorylation of eIF2 alpha in both hiPSCs/IMR90-1 and SH-SY5Y after NaCl treatment.**

**Supplementary Fig. 7.**

**
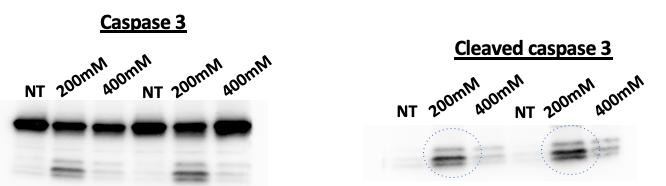
**

**Supplementary Fig. 7. Effects of NaCl on caspase-3 activation by western blot analysis.** hiPSCs/IMR90-1 cells were exposed to different concentrations of NaCl (0, 200 and 400mM) 1h. Cell lysates were subjected to western blot analysis with a specific antibody against caspase-3 or cleaved caspase-3. NT: No treat.

**Supplementary Fig. 8.**

**
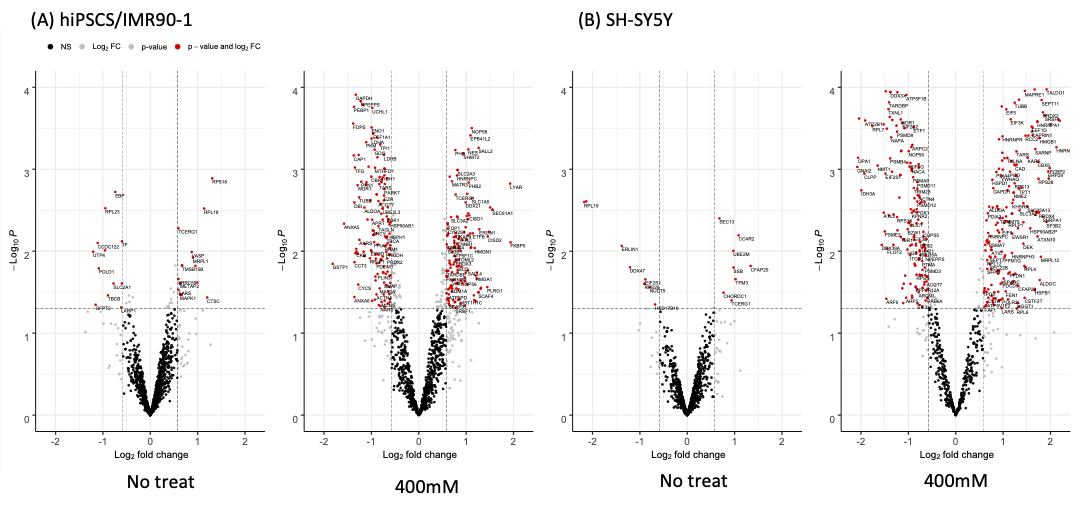
**

**Supplementary Fig. 8: Graphical representation of proteomic data.** (A) Quantitative proteomic data for hiPSCs/IMR90-1 cells and (B) Quantitative proteomic data for SHSy5y cells. Volcano plot showing the distribution of protein log2 Fold Change (FC) and the corresponding statistical -log10 p-value. Log FC shows the comparison of 200 mM and 400 mM NaCl treated cells with untreated cells. Horizontal dashed line indicates a significance level of 0.05, vertical dashed line indicates an arbitrary FC cut-off of 1.5 (log FC 0.58). Red dots indicate proteins which pass both criteria. NS: Non Significant.

**Supplementary Fig. 9.**

**Supplementary Fig. 9: Additional mitochondrial quantified proteins.** The total number of mitochondrial quantified proteins were three; COX7C, SHMT2, and ACO2 in both cell lines (hiPSCs/IMR90-1 and SH-SY5Y), under 200mM and 400mM of NaCl treatment compared to NT (no treated). The mitochondrial proteins, COX7C, SHMT2, ACO2 were statistically significant upregulated at 400mM of NaCl treatment in hiPSCs/IMR90-1 (Log_2_ larger that 0.58, which means higher than 1.50-fold change). COX7C was significantly lower in SH-SY5Y, Log_2_ smaller -0.58 which corresponds to smaller than 0.67 fold change. *FDR-adjusted p-value less than 0.05; ** FDR-adjusted p-value less than 0.01.
